# Comparative Analysis and Functional Mapping of *SACS* Mutations Reveal Novel Insights into Sacsin Repeated Architecture

**DOI:** 10.1002/humu.22269

**Published:** 2012-12-24

**Authors:** Alessandro Romano, Alessandra Tessa, Amilcare Barca, Fabiana Fattori, Maria Fulvia de Leva, Alessandra Terracciano, Carlo Storelli, Filippo Maria Santorelli, Tiziano Verri

**Affiliations:** 1 Department of Biological and Environmental Sciences and Technologies, University of SalentoLecce, Italy; 2 Neurogenetics and Molecular Medicine, IRCCS Fondazione Stella MarisPisa, Italy; 3 Neuromuscular Unit, IRCCS Bambino Gesù HospitalRome, Italy; 4 Department of Neurology, Federico II UniversityNaples, Italy

**Keywords:** autosomal recessive spastic ataxia of Charlevoix–Saguenay (ARSACS), comparative protein analysis, functional mapping of human mutations, neurodegeneration, protein domain architecture, repeated domains, *SACS*, sacsin

## Abstract

Autosomal recessive spastic ataxia of Charlevoix–Saguenay (ARSACS) is a neurological disease with mutations in *SACS*, encoding sacsin, a multidomain protein of 4,579 amino acids. The large size of *SACS* and its translated protein has hindered biochemical analysis of ARSACS, and how mutant sacsins lead to disease remains largely unknown. Three repeated sequences, called sacsin repeating region (SRR) supradomains, have been recognized, which contribute to sacsin chaperone-like activity. We found that the three SRRs are much larger (≥1,100 residues) than previously described, and organized in discrete subrepeats. We named the large repeated regions *Sacsin Internal RePeaTs* (*SIRPT1*, *SIRPT2*, and *SIRPT3*) and the subrepeats *sr1*, *sr2*, *sr3*, and *srX*. Comparative analysis of vertebrate sacsins in combination with fine positional mapping of a set of human mutations revealed that *sr1*, *sr2*, *sr3*, and *srX* are functional. Notably, the position of the pathogenic mutations in *sr1*, *sr2*, *sr3*, and *srX* appeared to be related to the severity of the clinical phenotype, as assessed by defining a severity scoring system. Our results suggest that the relative position of mutations in subrepeats will variably influence sacsin dysfunction. The characterization of the specific role of each repeated region will help in developing a comprehensive and integrated pathophysiological model of function for sacsin.

## Introduction

Autosomal recessive spastic ataxia of Charlevoix–Saguenay (ARSACS; MIM #270550) is an early-onset neurological disease presenting a founder effect in the Quebec regions of Charlevoix and Saguenay–Lac-St-Jean where the estimated carrier frequency is 1/22 [Bouchard et al., 1978; 1998]. The major clinical features of ARSACS include early-onset ataxia, later occurrence of spastic paraparesis, and brisk tendon reflexes, and an axonal sensory-motor peripheral neuropathy, with some instances of mental retardation or cognitive decline. Brain magnetic resonance imaging shows a distinct, tigroid appearance of the pons [Van Damme et al., 2009] and invariably an atrophied cerebellar vermis. Hypermyelination of the retinal nerve fibers [Bouchard et al., 1978, 1998] has long been considered a cardinal feature in Quebecois French–Canadian patients, and is not so obvious in cases from elsewhere [Criscuolo et al., 2004; Hara et al., 2005] or even absent. Several aspects including early appearance of abnormal pontocerebellar and retinal fibers seen at brain neuroimaging speak for a neurodevelopmental anomaly in ARSACS [Gazulla et al., 2012]. However, the progressive clinical course with involvement of the corticospinal tract and peripheral nerves in patients as well as studies in model mice questioned this hypothesis and suggested also the occurrence of a neurodegenerative process [Girard et al., 2012; Prodi et al., 2012].

The gene responsible for ARSACS (*SACS*) [Engert et al., 2000] is located on chromosome 13q12 and encodes sacsin, a protein whose canonic variant is described as a polypeptide of 4,579 amino acids (GenBank acc. no. NP_055178.3). The enormous size of the *SACS* gene and translated protein has considerably hindered biochemical studies to date, and currently much more is known about the genetics of ARSACS than about the function of sacsin in cells. Over the years, the number of ARSACS patients harboring mutations in the *SACS* gene has rapidly increased. They are distributed worldwide and are not limited to few ethnicities, and virtually any type of mutations has been discovered [Anheim et al., 2008].

How mutant sacsin leads to neurodegeneration remains largely unknown. Earlier work had indicated that sacsin might be involved in chaperone-mediated protein-folding activity [Engert et al., 2000] and play a role in regulating the Hsp70 chaperone machinery [Parfitt et al., 2009]. Recent biological and comparative genomic evidence suggested that sacsin is organized in a repetitive supradomain structure of ∼360 amino acids, named sacsin repeating region (SRR) [Anderson et al., 2010], which in turn might drive its function. Biochemical characterization demonstrated that such repetitive supradomain possesses ATPase activity, which appears to be a requirement for sacsin function, as a disease causing mutation leads to an alternate conformation incapable of hydrolyzing ATP [Anderson et al., 2010]. As well, this structure has been shown to enhance the refolding efficiency of a client protein, maintain it in soluble folding-competent states, and cooperate with members of the Hsp70 chaperone family to increase the yield of correctly folded client [Anderson et al., 2011]. Even more recently, sacsin has been shown to operate as a dimer and bind GTP at its C-terminus [Kozlov et al., 2011], with mutations in this region also resulting in loss of function. In addition, sacsin has been indicated as a potential substrate of the ubiquitin ligase Ube3A protein, which is responsible for Angelman syndrome (MIM #105830), a neurodevelopmental disorder with a motor component that shares same clinical aspects with ARSACS [Greer et al., 2010]. Such observations onto the function(s) of sacsin mainly arise from preliminary analysis on single putative domains that have been recognized along the sacsin sequence and are presently considered hallmarks of its structure. Finally, the generation of a sacsin knockout mouse is opening intriguing perspectives in the exploration of the pathophysiological basis of ARSACS, having shown that sacsin localizes to mitochondria and participates in regulation of mitochondrial dynamics via its interaction with dynamin-related protein 1 [Girard et al., 2012].

In the present work, we aimed at expanding our knowledge on the structure of sacsin. Three very large (≥1,100 amino acids) repeated regions were detected along the sacsin amino-acid sequence, each characterized by the occurrence of at least three subrepeats. A fourth subrepeat occurred in the first and third repeated region only. Such organization in domains is common to sacsin in all vertebrates including mammals, birds, reptiles, and fish. The comparative analysis of vertebrate sacsins architecture in combination with the fine positional mapping of a large set of disease causing mutations in human *SACS* well supported the concept of the functional nature of these novel domains. Furthermore, the location of a small selection of genetic variants detected in ARSACS was put in relation with the phenotype adopting a Spastic Ataxia (SPAX) rating system of clinical severity. Scoring mutations suggested original structure–function paradigms for sacsin, with hints on the relative relevance of novel and known domains in the activity of the protein.

## Materials and Methods

### Human *SACS* Gene, mRNA, and Protein Sequences and SNPs

The reference sequences for human (*Homo sapiens*) *SACS* gene (GenBank acc. no. NC_000013.10), mRNA (GenBank acc. no. NM_014363.4), and protein (GenBank acc. no. NP_055178.3) were as reported in *Entrez Gene* at the National Center for Biotechnology Information (NCBI) (http://www.ncbi.nlm.nih.gov/gene). The human *SACS* gene SNPs mapped in this study (missense and nonsense mutations only) were from *dbSNP* at NCBI (http://www.ncbi.nlm.nih.gov/snp) and from literature [Engert et al., 2000; Guernsey et al., 2010; Vermeer et al., 2009]. Throughout the manuscript, we systematically used names for both DNA and protein variations whenever appropriate, and adopted a mutation numbering system based on cDNA sequence as suggested by the internationally agreed mutation nomenclature (http://www.hgvs.org/).

### Pattern and Profile Searches

Putative domains were defined using the pattern and profile searches tools included in the ExPASy Proteomics Server (http://www.expasy.org/resources); in particular, the Simple Modular Architecture Research Tool 6 (SMART 6) (http://smart.embl-heidelberg.de/) [Letunic et al., 2009] and/or the ScanProsite tool (http://prosite.expasy.org/) [de Castro et al., 2006]. Internal repeats were detected by using the Prospero program, as included in SMART 6. Default parameters were always used for analyses and only domains above threshold were represented. SIM, an alignment tool for analysis of local similarity in nucleotide and amino-acid sequences (http://web.expasy.org/sim/) [Huang and Miller, 1991], served to generate pairwise alignments of sacsin versus internal repeats using default parameters. The computed alignments were viewed using the graphical viewer program LALNVIEW (http://pbil.univ-lyon1.fr/software/lalnview.html) [Duret et al., 1996]. Further details on the single computational tools and parameters used for analyses are reported in the legends to the figures as appropriate. Domains were drawn using the MyDomains image creator (http://prosite.expasy.org/mydomains).

### Protein Sequence Alignments and Phylogenesis

On the basis of the genomic analysis detailed in the Supporting Information, the deduced protein sequences of orangutan, dog, horse, mouse, rat, chicken, zebra finch, anole lizard, fugu, tetraodon, stickleback, medaka, and zebrafish were obtained and used for alignments. Pairwise alignments of human versus the other vertebrate sacsin proteins were obtained by using SIM, as detailed above. Multiple sequence alignment of vertebrate sacsin proteins was obtained by using ClustalW2 using default parameters (http://www.ebi.ac.uk/Tools/clustalw2/index.html) [Larkin et al., 2007]. The phylogenetic reconstruction was generated by the neighbor-joining method [Saitou and Nei, 1987], as implemented in the Molecular Evolutionary Genetics Analysis 4 (MEGA4) software (http://www.megasoftware.net/) [Tamura et al., 2007].

### Definition of a SPAX Scoring System (SPAX score) in ARSACS

Definition of a clinical score in ARSACS is lagging behind, although reliable and valid composite scores have been developed for the highly similar inherited ataxias [Trouillas et al., 1997] and the hereditary spastic paraplegias [Schüle et al., 2006]. To define a posteriori a measure of disease severity in ARSACS and to correlate scores with type and location of mutations in sacsin, we put together a measure of severity in SPAX score that takes into account the “core features” of ARSACS, including cerebellar ataxia, spastic paraplegia, and peripheral neuropathy. We are aware that SPAX scores are only an initial attempt to score disease severity, especially in the absence of functional tests, but the rating system has an intrinsic value in that it sums the gravity of the individual hallmarks of the disease through the use of validated scales. In particular, we used the parameters developed in the Scale for the Assessment and Rating of Ataxia [Schmitz-Hübsch et al., 2006] for cerebellar ataxia, the Spastic Paraplegia Rating Scale for motor symptoms and spasticity [Schüle et al., 2006], and the modified version of the Charcot–Marie–Tooth neuropathy score [Murphy et al., 2011] for peripheral neuropathy. In addition, cognitive impairment (0, absent to 3, if severe) and ocular findings (from 0, normal to 4, maximal abnormality) were assessed. When visual abnormalities were detected only at optical coherence tomography, a unit was subtracted from the subscore. The several items of the scales were reviewed by two independent investigators blind to the genotype, duplicated items removed, data on single items averaged, and then corrected for disease duration whenever possible (or for averaged disease duration in a family). A grade of functional severity in ARSACS varying from 0 to 2 (maximal severity) was then calculated.

## Results

### Identification of Novel Domains in Human *SACS*

**Figure 1:**
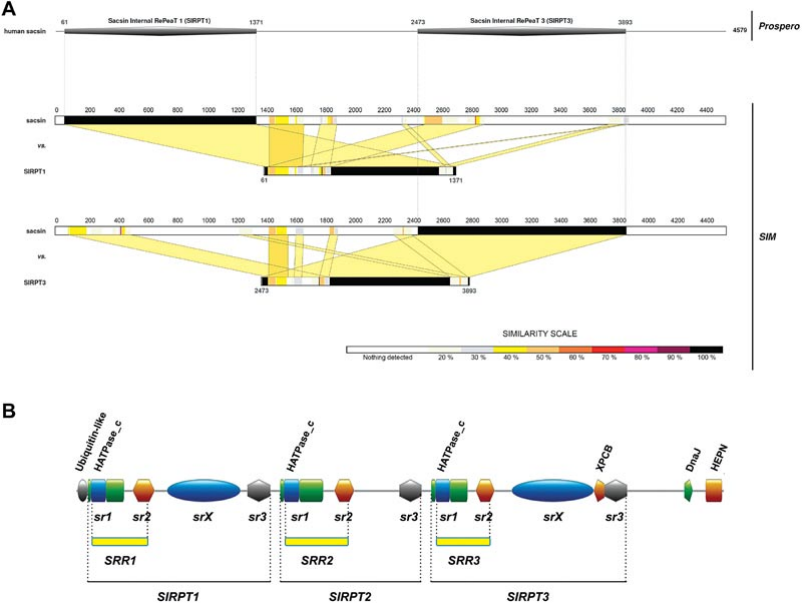
Identification of domains in human sacsin. **A:** (upper panel) Internal repeats above threshold were detected by Prospero. (Lower panel) Pairwise sequence alignments of human sacsin versus the first and the second Prospero repeat (corresponding to amino acids 61–1,371 and 2,473–3,893, respectively) were generated by SIM. The computed alignments were visualized by LALNVIEW. Percent identity is reported in the figure. Different colors indicate different degrees of similarity (amino-acid identity) along the aligned sequences (black: 100%; white: nothing detected). **B:** *Sacsin Internal RePeaTs* (*SIRPTs*) and relevant subrepeats 1 (*sr1*), 2 (*sr2*), 3 (*sr3*), and X (*srX*) within *SIRPTs* are indicated, spanning along the protein sequence as follows (amino-acid range in parentheses): *SIRPT1* (amino acids 84–1,374), *SIRPT2* (1,444–2,443), *SIRPT3* (2,512–3,896), *SIRPT1–sr1* (84–339), *SIRPT1–sr2* (400–557), *SIRPT1–sr3* (1,212–1,374), *SIRPT1–srX* (644–1,162), *SIRPT2–sr1* (1,444–1,747), *SIRPT2–sr2* (1,826–1,968), *SIRPT2–sr3* (2,287–2,443), *SIRPT3-sr1* (2,512–2,768), *SIRPT3–sr2* (2,826–2,960), *SIRPT3–sr3* (3,736–3,896), *SIRPT3–srX* (3,081–3,659). The sacsin repeating region (SRR) supradomains defined by Anderson et al. (2010, 2011) are indicated as SRR1 (amino acids 107–505), SRR2 (1,471–1,921) and SRR3 (2,539–2,922), with each supradomain composed of an *sr1*, an *sr2*, and an *sr1*–*sr2* connecting (linker) region. Please note that *sr1* starts 23–27 amino acids upstream the C-terminus of the SRR domain (with SRR virtually starting with the HATPase_c domain) and *sr2* ends 38–52 amino acids downstream the N-terminus of the SRR domain. Putative domains above threshold as detected by using SMART 6 and/or ScanProsite are also indicated: ubiquitin-like (ubiquitin; PFAM acc. no. PF00240), HATPase_c (histidine kinase-like ATPases; SMART acc. no. SM00387), DnaJ (DnaJ molecular chaperone homology domain; SMART acc. no. SM00271), HEPN (higher eukaryotes and prokaryotes nucleotide-binding domain; SMART acc. no. SM00748). For sake of clarity, the putative sacsin XPCB domain is also shown. Domains were drawn using the MyDomains image creator.

Along with the original description of human *SACS* [Engert et al., 2000], it was suggested that repeating regions, two of which containing the putative ATP-binding domain of Hsp90, might have occurred in the sacsin protein. At that time, human *SACS* was considered to consist of a single gigantic exon spanning 12,794 bp [Engert et al., 2000]. With the identification of nine (one noncoding and eight coding) additional exons upstream of this gigantic exon, the presence of conserved amino-acid sequences occurring in triplicate along the encoded protein started to be foreseen, and very recently the formal description of the SRR supradomain has been proposed [Anderson et al., 2010]. In this study, a systematic analysis of domains along the human sacsin amino-acid sequence was performed. In particular, besides the well-known ubiquitin-like (ubiquitin; PFAM acc. no. PF00240), DnaJ (DnaJ molecular chaperone homology domain; SMART acc. no. SM00271), and HEPN (Higher Eukaryotes and Prokaryotes Nucleotide-binding domain; SMART acc. no. SM00748) domains (see Fig. 1B), two large *Prospero* repeats, corresponding to the 61–1,371 and 2,473–3,893 protein fragments of the human sacsin, were detected along the polypeptide chain (Fig. 1A). Interestingly, both repeats shared similarity with a third homologous region in between them (along the 1,372–2,472 protein fragment), as detected by SIM analysis of sacsin protein versus each Prospero repeat (Fig. 1A). Similar results were also obtained by using the HHRepID program [Biegert and Söding, 2008] (data not shown). We named these three large homologous repeating regions Sacsin Internal RePeaTs (namely *SIRPT1*, *SIRPT2*, and *SIRPT3*; see Fig. 1). In spite of their low overall similarity (e.g., 16%–18% in human sacsin, with *SIRPT1* vs. *SIRPT2*: 17%, *SIRPT1* vs. *SIRPT3*: 16%, and *SIRPT2* vs. *SIRPT3*: 18%), each *SIRPT* displayed, based on the degree of local similarity, at least three subrepeats (Fig. 1B) that were distanced by regions of extremely low similarity. We named these: subrepeat 1 (*sr1*), 2 (*sr2*), and 3 (*sr3*) (for position of the subrepeats along the protein, see Fig. 1B). Noteworthy, each *sr1* contained a well-recognizable HATPase_c (Histidine kinase-like ATPases; SMART acc. no. SM00387) domain, which is adopted by the ATP-binding and catalytic domain of (among others) the members of the vast GHKL class of proteins (so-called after the founding members of the class: DNA Gyrase, Hsp90, bacterial histidine Kinases and MutL) [Dutta and Inouye, 2000] (Fig. 1B). Also, within the *SIRPT* architecture, *sr1*s and *sr2*s virtually corresponded to Anderson et al. SRR supradomain [Anderson et al., 2010] (Fig. 1B). On the other side, *sr3* revealed no obvious relationships to any of the so far acknowledged domains included in databases. Besides the *sr1*, *sr2*, and *sr3* domains described above, another repeated region could be identified in *SIRPT1* and *SIRPT3* in the area of very limited similarity. In fact, a long repeated region in *SIRPT1* shared similarity with a homologous region in *SIRPT3*. We named this region *srX* (Fig. 1B). The *srX* domain had no obvious counterpart in the significantly shorter *SIRPT2* (see Fig. 1A and B). Also, *srX* had no obvious similarity to any of the so far acknowledged domains in databases. Interestingly, in *SIRPT3*, the amino-acid sequence between *srX* and *sr3* corresponded to a sacsin region previously reported to share limited homology with the Xeroderma Pigmentosum complementation group C binding (XPCB) domain of hHR23A [Kamionka and Feigon, 2004] and recently implicated in interactions with the ubiquitin ligase Ube3A [Greer et al., 2010] (Fig. 1B).

### Conservation of Sacsin Structural Organization among Vertebrates

**Figure 2:**
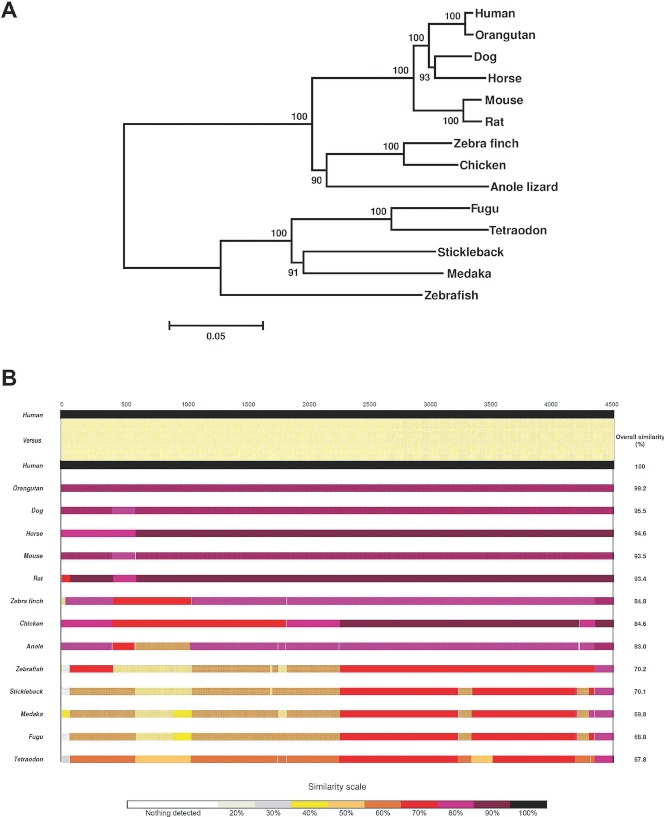
Comparative analysis of vertebrate sacsin proteins. **A:** Unrooted phylogenetic tree depicting the evolutionary relationship of mammalian (orangutan, dog, horse, mouse, and rat), bird (chicken and zebra finch), reptilian (anole lizard), and fish (zebrafish, stickleback, medaka, fugu, and tetraodon) sacsin proteins. The unrooted tree was constructed using the neighbor-joining (NJ) method based on the alignment of the amino-acid sequences of the vertebrate sacsins. Bootstrap values (1,000 replicates) indicating the occurrence of nodes are reported above each branch in the figure. **B:** Schematic alignment of human versus the above listed vertebrate sacsin proteins. Pairwise sequence alignments and scores were generated using SIM. The computed alignments were visualized by LALNVIEW. Species are aligned according to their overall similarity (amino-acid identity) with respect to the human protein (from the highest to the lowest degree of overall similarity). Percent identity is reported in the figure. Different colors along the sequences are indicative of different degrees of similarity along the aligned sequences (black: 100%; white: nothing detected).

Comparative analysis of homologous proteins across phylogenetically distant species represents a powerful method for detecting conserved structural elements in proteins. Comparison of human sequences with sequences of other mammals, avians, reptiles, and teleost fish is valuable; in particular, teleosts offer maximal stringency for sequence comparisons among vertebrates. On this conceptual basis, we compared amino-acid sequence of sacsins from human with fish, having verified that: (1) genes encoding sacsin proteins are found in all vertebrate genomes sequenced so far, (2) sacsin proteins may have similar functional role(s) in all vertebrates, as supported by the evidence of similar expression patterns in mammals [Engert et al., 2000; Parfitt et al., 2009] and fish (such as zebrafish; see Supp. Fig. S1 and Supp. Table S1). In particular, as a result of a comprehensive gene analysis among vertebrates, sacsin proteins were deduced from human and other 13 vertebrate species, namely five mammals (orangutan, mouse, rat, horse, and dog), two birds (chicken and zebra finch), one reptile (anole lizard), and five fish (zebrafish, tetraodon, fugu, stickleback, and medaka). Then, the protein sequences were compared (for details, see Supp. Fig. S2), and the phylogenetic relationships among them are summarized in Figure 2A. With respect to the human protein, the other mammalian sacsins exhibited an overall degree of similarity (amino-acid identity) that varied from ∼99% to ∼93%, whereas the bird sacsins revealed an overall similarity of ∼84% and the reptilian sacsin of 83% (for details, see Supp. Table S2). Fish proteins shared an overall degree of similarity with human sacsin that varied from ∼70% to ∼68% (Supp. Table S2). As expected, degrees of similarity locally varied along the protein sequence. The local degree of similarity is depicted in Figure 2B. In spite of these differences, all vertebrate sacsins conserved the same structural architecture as the human sacsin (see Supp. Fig. S3).

### Comparative Analysis of Vertebrate Protein Architecture and Positional Mapping of Human *SACS* Mutations Reveal the Functional Nature of the Sacsin Repeated Domains

On the basis of our *SIRPT*-centered protein architecture and sequence similarity data, the following intra/intersequence alignment strategy was played out to identify and typify unique conserved elements in the repeated domains of the vertebrate sacsin proteins. Namely, the amino-acid sequences corresponding to *SIRPT1-sr1*, *SIRPT2-sr1*, and *SIRPT3-sr1* from the human and other vertebrate sacsins were aligned against each other (see Supp. Fig. S4). The same procedure was applied to the sequences corresponding to *SIRPT1-sr2*, *SIRPT2-sr2*, and *SIRPT3-sr2* (see Supp. Fig. S5), to *SIRPT1-sr3*, *SIRPT2-sr3*, and *SIRPT3-sr3* (see Supp. Fig. S6), and to *SIRPT1-srX* and *SIRPT3-srX* (see Supp. Fig. S7) of the human and other vertebrate sacsins. Notably, in spite of the highly selective alignment procedure, a number of amino-acid residues still kept appearing conserved in the same position of mate repeated domains.

**Figure 3:**
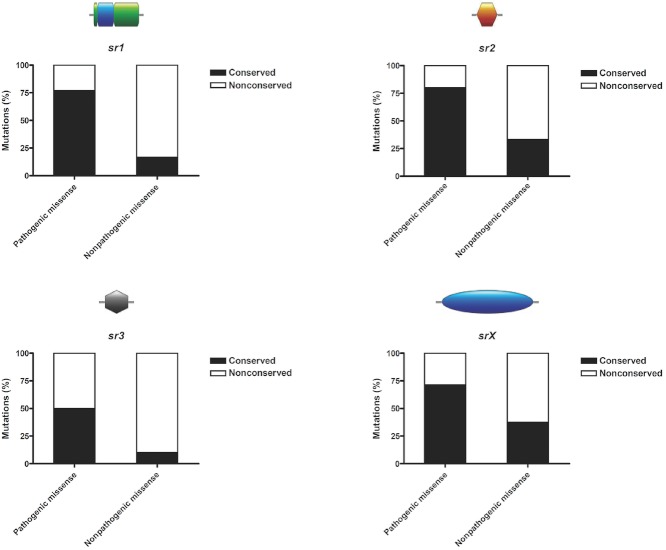
Relative amount of conserved versus nonconserved missense mutations in *SIRPT sr1*, *sr2*, *sr3*, and *srX* domains. When mapped on our multiple alignments (see Supp. Figs. S4–S7), in each domain “conserved” (i.e., identical, conserved and semi-conserved, as assessed by ClustalW) pathogenic missense mutations were invariably over-represented with respect to missense nonpathogenic mutations (for details, see Supp. Table S5). Unclear mutations (i.e., variants not yet clearly associated with disease; for details, see Supp. Tables S3–S5) were omitted from the analysis.

If the sacsin repeated domains are functional, the amino acids that are found in these conserved positions should then be considered critical for sacsin function. Accordingly, in such repeated and conserved positions, one should expect to find more missense mutations associated with disease (missense pathogenic) than missense mutations not associated with disease (missense nonpathogenic) and/or nonsense (protein truncating) pathogenic mutations [Miller and Kumar, 2001; Miller et al., 2003]. To test this hypothesis, we collected missense (pathogenic and nonpathogenic) and nonsense mutations that have been reported to occur in human sacsin. In particular, Table 1 represents the recent update (January 2012) of all the acknowledged missense and nonsense mutations that are clearly pathogenetic in ARSACS patients of different geographic origins (Supp. Appendix I lists pathogenic missense and nonsense mutations identified later than January 2012 and frameshift mutations not used in this study). Furthermore, Supp. Table S3 represents the list of all the missense mutations that have been described as SNPs in humans up to January 2012; for the most part, these mutations were recognized as undoubtedly nonpathogenic and were used for analysis (for details, see legend to Supp. Table S3) (Supp. Appendix II and Supp. Appendix III report a recent update of SNPs from dbSNP and NHLBI Exome Sequencing Project, respectively). Detailed positional information and distribution of the mutations in the various domains along the human protein are summarized in Supp. Figure S2 and Supp. Table S4. All the mutations falling in positions within the *sr1*, *sr2*, *sr3*, and *srX* domains have been represented in Supp. Figures S4–S7. The relative amounts of missense pathogenic mutations, on one hand, and missense nonpathogenic mutations, on the other—expressed as percent of conserved *vs*. non-conserved mutations—are reported in Figure 3. As expected, with respect to the group of missense nonpathogenic mutations, missense pathogenic mutations were invariably over-represented in conserved positions in *sr1*, *sr2*, *sr3*, and *srX* (for details, see Supp. Table S5), thus suggesting that the four repeated domains of the *SIRPT* regions identified in this work (that include, with *sr1* and *sr2*, and go beyond, with *sr3* and *srX*, the SRR design; see Fig. 1B) [Anderson et al., 2010] do play a functional role in the sacsin protein.

**Table 1:** Human *SACS* Gene (Missense, Nonsense, and Frameshift Leading to Immediate Premature Protein Truncation only) Pathogenic Mutations Mapped in this Study

		Mutation		Amino-acid change		
				
Origin	Exon	Original nucleotide position reported^a^ (see *Reference*, last column)	Newly assigned nucleotide position in CDS^b^	Original amino-acid position reported^c^ (see *Reference*, last column)	Newly assigned amino-acid position in protein^d^	Reference
Synthetic construct	3	sdm	sdm	R50A	p.R50A	Parfitt et al., 2009
Synthetic construct	3	sdm	sdm	R51A	p.R51A	Parfitt et al., 2009
Synthetic construct	4	sdm	sdm	L58A	p.L58A	Parfitt et al., 2009
Synthetic construct	4	sdm	sdm	D60A	p.D60A	Parfitt et al., 2009
The Netherlands	7	c.502G>T	c.502G>T	p.D168Y	p.D168Y	Vermeer et al. (2008, 2009)
Synthetic construct		sdm	sdm	D168Y	p.D168Y	Anderson et al. (2010)
Belgium	7	c.602C>A	c.602C>A	p.T201K	p.T201K	Baets et al. (2010)
Italy	8	c.815G>A	c.815G>A	p.R272H	p.R272H	In house database
Maritime Canada (Acadian descent)	8	c.814C>T	c.814C>T	p.R272C	p.R272C	Guernsey et al. (2010)
Italy	8	c.826C>T	c.826C>T	p.R276C	p.R276C	Prodi et al. (2012)
Aragona-Spain/Croatia	8	c.832C>T	c.832C>T	p.Q278X	p.Q278X	Gazulla et al. (2012)
Japan	8	922C>T	c.922C>T	L308F	p.L308F	Takado et al. (2007)
The Netherlands	8	c.961C>T	c.961C>T	p.R321X	p.R321X	Vermeer et al. (2008, 2009)
Italy	8	c.1373C>T	c.1373C>T	p.T458I	p.T458I	In house database
Italy	8	c.1420C>T	c.1420C>T	p.R474C	p.R474C	In house database
The Netherlands	8	c.1475G>A	c.1475G>A	p.W492X	p.W492X	Vermeer et al. (2008, 2009)
France	8	c.1607C>T	c.1607C>T	p.P536L	p.P536L	Anheim et al. (2008)
Morocco	8	c.1667T>C	c.1667T>C	p.L556P	p.L556P^e^	Baets et al. (2010)
Aragona-Spain	8	c.1894C>T	c.1894C>T	p.R632W	p.R632W	Gazulla et al. (2012)
The Netherlands	9	c.2182C>T	c.2182C>T	p.R728X	p.R728X	Vermeer et al. (2008, 2009)
UK	10	c.2224C>T	c.2224C>T	p.R742X	p.R742X	Terracciano et al. (2010)
Japan	10	g.2405T>C	c.2405T>C	L802P	p.L802P	Kamada et al. (2008)
Belgium	10	c.2971T>C	c.2971T>C	p.C991R	p.C991R	Baets et al. (2010)
Japan	10	987T>C	c.3161T>C	F304S	p.F1054S	Shimazaki et al. (2005)
Aragona-Spain	10	c.3198T>A	c.3198T>A	p.C1066X	p.C1066X	Gazulla et al. (2012)
Belgium	10	c.3491T>A	c.3932T>A	p.M1164K	p.M1311K	Ouyang et al. (2008)
Japan	10	3774C>T	c.4033C>T	Q1198X	p.Q1345X	Okawa et al. (2006)
Italy	10	1858C>T	c.4108C>T	Q620X	p.Q1370X	Grieco et al. (2004)
Turkey	10	g.2018T>C	c.4182T>C	C648R	p.C1398R	Richter et al. (2004)
Italy	10	c.4198T>A	c.4198T>A	p.Y1400N	p.Y1400N	In house database
Italy	10	c.4567T>C	c.4567T>C	p.W1523R	p.W1523R	In house database
Serbia	10	c.4724G>C	c.4724G>C	p.R1575P	p.R1575P	Baets et al. (2010)
Spain	10	c.4748C>G	c.4748C>G	p.P1583R	p.P1583R	In house database
Belgium	10	c.4760A>G	c.4760A>G	p.H1587R	p.H1587R	Baets et al. (2010)
Algeria	10	c.4934G>A	c.4934G>A	p.R1645Q	p.R1645Q	In house database
The Netherlands (Turkish descent)	10	c.4957G>T	c.4957G>T	p.E1653X	p.E1653X	Vermeer et al. (2008, 2009)
The Netherlands	10	c.5125C>T	c.5125C>T	p.Q1709X	p.Q1709X	Vermeer et al. (2008, 2009)
The Netherlands	10	c.5143A>T	c.5143A>T	p.K1715X	p.K1715X	Vermeer et al. (2008, 2009)
Italy	10	c.5629C>T	c.5629C>T	p.R1877X	p.R1877X	Anesi et al. (2011)
Italy	10	c.5639C>T	c.5639C>T	p.T1880I	p.T1880I	In house database
Algeria	10	c.5719C>T	c.5719C>T	p.R1907X	p.R1907X	Prodi et al. (2012)
Tunisia	10	3662T>C	c.5836T>C	W1196R	p.W1946R	El Euch-Fayache et al. (2003)
Japan	10	6355C>T	c.6355C>T	R2119X	p.R2119X	Hara et al. (2007)
Algeria	10	c.6409C>T	c.6409C>T	p.Q2137X	p.Q2137X	H'mida'Ben Brahim et al. (2011)
Italy	10	c.6680T>C	c.7121T>C	p.L2374S	p.L2374S	Terracciano et al. (2009)
Belgium	10	c.7276C>T	c.7276C>T	p.R2426X	p.R2426X	Baets et al. (2010)
Quebec	10	g.5254C>T	c.7504C>T	R1752X	p.R2502X	Engert et al. (2000)
France	10	c.7673C>T	c.7673C>T	p.A2558V	p.A2558V	Anheim et al. (2008)
Spain	10	7848C>T	c.8107C>T	R2556C	p.R2703C	Criscuolo et al. (2005)
France	10	c.8289_8291delTTC	c.8289_8291delTTC	p.Y2763X	p.Y2763X	In house database
Morocco	10	c.8393C>A	c.8393C>A	p.P2798Q	p.P2798Q^e^	Baets et al. (2010)
Aragona-Spain/Italy	10	c.8677A>T	c.8677A>T	p.R2893X	p.R2893X	In house database
Aragona-Spain	10	c.9670C>T	c.9670C>T	p.R3224X	p.R3224X	Gazulla et al. (2012)
Japan	10	7492T>C	c.9742T>C	W2498R	p.W3248R	Ogawa et al. (2004)
Tunisia	10	c.10290C>G	c.10290C>G	p.Y3430X	p.Y3430X	H'mida'Ben Brahim et al. (2011)
The Netherlands (English descent)	10	c.10442T>C	c.10442T>C	p.L3481P	p.L3481P	Vermeer et al. (2008, 2009)
The Netherlands	10	c.10906C>T	c.10906C>T	p.R3636X	p.R3636X	Vermeer et al. (2008, 2009)
Belgium	10	c.10907G>A	c.10907G>A	p.R3636Q	p.R3636Q^f^	Baets et al. (2010)
Belgium	10	c.10934T>C	c.10934T>C	p.L3645P	p.L3645P	Baets et al. (2010)
Belgium	10	c.10954C>A	c.10954C>A	p.P3652T	p.P3652T^f^	Baets et al. (2010)
Belgium	10	c.10517T>C	c.10958T>C	p.F3506S	p.F3653S	Breckpot et al. (2008)
Italy	10	c.10743C>T	c.11185C>T	p.Q3582X	p.Q3729X	Kamionka and Feigon (2004), Masciullo et al. (2008)
Tunisia	10	c.11374C>T	c.11374C>T	p.R3792X	p.R3792X	Bouhlal et al. (2008)
Maritime Canada (Acadian descent)	10	c.11707C>T	c.11707C>T	p.R3903X	p.R3903X	Guernsey et al. (2010)
The Netherlands	10	c.12160C>T	c.12160C>T	p.Q4054X	p.Q4054X	Vermeer et al. (2008, 2009)
Tunisia	10	c.10046G>C	c.12220G>C	p.A3324P	p.A4074P	El Euch-Fayache et al. (2003), H'mida-Ben Brahim et al. (2011)
Italy	10	c.12428_12429insA	c.12428_12429insA	p.Y4143X	p.Y4143X	Prodi et al. (2012)
Japan	10	10723C>T	c.12973C>T	R3575X	p.R4325X	Takiyama (2006), Yamamoto et al. (2006)
The Netherlands	10	c.12992G>A	c.12992G>A	p.R4331Q	p.R4331Q	Vermeer et al. (2008, 2009)
Synthetic construct		sdm	sdm	R4331Q	p.R4331Q	Parfitt et al., 2009
Italy		c.12991C>T	c.12991C>T	p.R4331W	p.R4331W	Prodi et al. (2012)
Synthetic construct	10	sdm	sdm	H4337Q	p.H4337Q	Parfitt et al., 2009
Belgium	10	c.13027G>A	c.13027G>A	p.E4343K	p.E4343K	Baets et al. (2010)
Italy	10	c.13132C>T	c.13132C>T	p.R4378X	p.R4378X	Anesi et al. (2011)
UK	10	c.13237C>T	c.13237C>T	p.Q4413X	p.Q4413X	Terracciano et al. (2010)
Aragona-Spain	10	c.13405G>C	c.13405G>C	p.A4469P	p.A4469P	Gazulla et al. (2012)
Belgium	10	c.13523A>C	c.13523A>C	p.K4508T	p.K4508T	Baets et al. (2010)
Turkey	10	g.11471A>G	c.13645A>G	N3799D	p.N4549D	Richter et al. (2004)
Synthetic construct		sdm	sdm	N4549D	p.N4549D	Kozlov et al. (2011)

CDS, coding sequence; sdm, site-directed mutagenesis.

This table represents the recent update (January 2012) of the acknowledged (41 missense, 28 nonsense, and two frameshift leading to premature protein truncation) mutations that associate to pathogenicity in ARSACS patients of different geographic origins. A total of 10 variants (eight missense, one nonsense, and one frameshift leading to premature protein truncation) are new and as yet unpublished, and were identified upon a large collaborative clinical-genetic work performed in the laboratory of one of us (Filippo M. Santorelli, in house database) on behalf of SPATAX, the Euro-Mediterranean clinical network on inherited ataxias and spastic paraplegias. Detailed information on the associated clinical and paraclinical features in patients as well as on mutation analyses will be presented elsewhere. This table also lists eight missense mutations artificially generated by molecular biology approaches in the ubiquitin-like domain (four variants) [Parfitt et al., 2009], HATPase_c domain (one variant) [Anderson et al., 2010], DnaJ domain (two variants) [Parfitt et al., 2009], and HEPN domain (one variant) [Kozlov et al., 2011] that helped defining the functional nature of such domains.

a Nucleotide positions and changes are indicated as reported in the original article and refer to different NCBI Reference Sequences (see *Reference*, last column). DNA mutation numbering system in use, based on cDNA sequence (with a “c.” symbol before the number) (http://www.hgvs.org), can be found for more recent mutations only. For elder mutations, numbering system based on genomic sequences can be found (with a “g.” symbol before the number).

b On the basis of the following NCBI Reference Sequence: GenBank acc. no. NM_014363.4. DNA mutation numbering system in use is based on cDNA sequence (with a “c.” symbol before the number). Nucleotide numbering reflects cDNA numbering with +1 corresponding to the A of the ATG translation initiation codon in the reference sequence, with the initiation codon being codon 1 (http://www.hgvs.org).

c Amino-acid positions and changes are indicated as reported in the original article and refer to different NCBI Reference Sequences (see *Reference*, last column). Amino-acid change numbering system in use, based on protein sequence (with a “p.” symbol before the letter) (http://www.hgvs.org), can be found for more recent variants only.

d On the basis of the following NCBI Reference Sequence: GenBank acc. n. NP_055178.3. Amino-acid change numbering system in use is based on protein sequence (with a “p.” symbol before the letter) (http://www.hgvs.org).

e Double mutant alleles.

f Double mutant alleles.

g New variants not mapped in this study are reported in Supp. Appendix I.

The functional nature of *sr1*, *sr2*, *sr3*, and *srX* is also sustained by the observation that in human sacsin pathogenic missense mutations were found to be over-represented in these domains with respect to the regions between domains (interdomains), as qualitatively assessed by calculating the likelihood of occurrence of missense pathogenic mutations, that is, the ratio of the percentage mutations on a given region and the percentage of amino acids of the protein on the same region (for details, see Table 2). In particular, the calculated likelihood was 1.97, 1.42, 0.79, and 0.51 for *sr1*, *sr2*, *srX*, and *sr3* domains, respectively, with respect to 0.35 for the interdomains.

**Table 2:** Percentage of the Amino Acids in a Given Region (% protein), Percentage of the Mutations in the Same Region (% mutation) and Likelihood of a Mutation Occurring in the Region (% Mutation/% Protein), Calculated as the Ratio of the Percentage Mutations on a Given Region and the Percentage of Amino Acids of the Protein on the Region

Region	Whole sacsin	Ubiquitin-like	sr1	sr2	sr3	*srX*	XPCB	DnaJ	HEPN	Interdomains
Protein (fragment) length (aa)	4,579	72	817	436	481	1098	76	60	117	1,422
% Protein	100	1.57	17.84	9.52	10.51	23.98	1.66	1.31	2.56	31.05
Missense mutations	37	0	13	5	2	7	0	3	3	4
% Mutation	100	0	35.13	13.51	5.41	18.92	0	8.11	8.11	10.81
Likelihood (% mutation/% protein)	1.00	0.00	1.97	1.42	0.51	0.79	0.00	6.19	3.17	0.35

aa, amino acids.

### Functional Relevance of *sr1*, *sr2*, *srX*, and *sr3* in Sacsin Protein Based on Composite SPAX Scores Analysis

To investigate on the putative functional relevance of the various repeated domains that result from the proposed new sacsin architecture, we analyzed the clinical phenotype in patients selected for having, in a given domain (i.e., *sr1*, *sr2*, *srX*, and *sr3*), a missense pathogenic mutation (1) in homozygosis or (2) in heterozygosis with a frameshift mutation, a stop mutation, or a macrodeletion (see Table 3). It is reasonable to think that in an autosomal recessive disorder, such as ARSACS, frameshift mutations, stop mutations, and macrodeletions can abolish sacsin function, although other mechanisms, such as dominant-negative effects, cannot totally be excluded until functional tests are performed. Under these conditions, we expect that differences in the clinical phenotypes observed in patients (1) are due to the effect(s) of the missense mutation on protein function and (2) provide (at least in part) information on the functional relevance of the protein domain where the missense mutation acts. In fact, although the nature of the substituted amino acid may contribute per se to the severity of the phenotype, it cannot be ignored that the effect of an amino-acid substitution depends on the protein domain where the substitution occurs. As a means to evaluate the pleomorphic clinical phenotype of ARSACS, we defined a composite SPAX score, which takes into account the major core features (cognitive, cerebellar, spasticity, peripheral nerve, and retinopathy) that are part of the disease. This scoring system is largely based on validated rating scales for spasticity, peripheral neuropathy, and cerebellar function, corrected for disease duration and used to evaluate the severity of the clinical phenotype (see Table 3).

**Table 3:** Composite SPAX Score Assigned to Selected ARSACS Patients

*SACS* mutation						Severity of clinical phenotype					
						
Allele 1^b^	Allele 2^b^	Reference^a^	Sacsin internal repeat (SIRPT) subregion (sr) or interdomain where the mutation is located	Duration (yrs)	Onset	Cognitive	Cerebellar	Spasticity	Peripheral neuropathy	Retinal	Composite SPAX score = total score corrected for years disease duration/100
c.4182T>C (p.C1398R)	c.4182T>C (p.C1398R)	Richter et al. (2004)	Interdomain between SIRPT1–sr3 and SIRP2–sr1	16	3	1	1	1	1	1	0.88
c.4198T>A (p.Y1400N)	c.5719C>T (p.R1907X)	In house database	Interdomain between SIRPT1–sr3 and SIRP2–sr1	11	2	0	1	2	2	1	0.55
c.4567T>C (p.W1523R)	c.11303insG (p.T3768fsX1)	In house database	SIRPT1–sr1	18	3	1	2	2	2	0	0.93
c.502G>T (p.D168Y)	c.502G>T (p.D168Y)	Vermeer et al. (2008)	SIRPT1–sr1	33	3	0	2	2	0	0	1.16
c.602C>A (p.T201K)	c.7276C>T (p.R2426X)	Baets et al. (2010)	SIRPT1–sr1	26	3	0	2	2	2	0	0.77
c.4748C>G (p.P1583R)	c.8677A>T (p.R2893X)	In house database	SIRPT1–sr1	15	3	1	3	2	1	1	1.24
c.4760A>G (p.H1587R)	c.3421_3422insAC (p.L1141fsX9)	Baets et al. (2010)	SIRPT1–sr1	15	1	1	3	1	3	3	1.34
c.815G>A (p.R272H)	c.815G>A (p.R272H)	In house database	SIRPT1–sr1	34	3	2	2	3	3	0	1.30
c.814C>T (p.R272C)	c.814C>T (p.R272C)	Guernsey et al. (2010)	SIRPT1–sr1	24	3	1	2	2	3	0	1.10
c.4934G>A (p.R1645Q)	c.2224C>T (p.R472X)	In house database	SIRPT1–sr1	21	3	2	3	3	2	2	1.20
c.826C>T (p.R276C)	c.826C>T (p.R276C)	Prodi et al. (2012)	SIRPT1–sr1	28	2	2	3	3	3	0	0.78
c.8107C>T (p.R2703C)	c.8107C>T (p.R2703C)	Criscuolo et al. (2005)	SIRPT2–sr1	29	1	0	3	3	3	0	0.80
c.922C>T (p.L308F)	c.922C>T (p.L308F)	Takado et al. (2007)	SIRPT1–sr1	32	2	0	2	3	3	0	1.07
c.1373C>T (p.T458I)	Δ (1.5 Mb macrodeletion)	In house database	SIRPT1–sr2	24	2	0	3	1	1	3	0.70
c.5639C>T (p.T1880I)	c.8289_8291delTTC (p.Y2763X)	In house database	SIRPT2–sr2	24	2	0	3	2	1	2	0.70
c.1420C>T (p.R474C)	c.5719C>T (p.R1907X)	In house database	SIRPT1–sr2	13	3	1	3	3	2	1	1.69
c.5836T>C (p.W1946R)	c.5836T>C (p.W1946R)	El Euch-Fayache et al. (2003)	SIRPT2–sr2	34	2	0	3	3	1	1	1.30
c.1894C>T (p.R632W)	c.12973C>T (p.R4325X)	Gazulla et al. (2012)	Interdomain between SIRPT1–sr2 and SIRPT1–srX	17	2	1	1	2	1	0	0.67
c.9742T>C (p.W3248R)	c.9742T>C (p.W3248R)	Ogawa et al. (2004)	SIRPT3–srX	22	2	2	1	2	1	3	0.84
c.2405T>C (p.L802P)	c.482delA (p.N161fsX14)	Kamada et al. (2008)	SIRPT1–srX	17	1	2	3	1	2	0	0.88
c.10442T>C (p.L3481P)	c.9910insT (p.L3304fsX14)	Vermeer et al. (2008)	SIRPT3–srX	40	2	0	1	2	1	0	0.76
c.3161T>C (p.F1054S)	c.3161T>C (p.F1054S)	Shimazaki et al. (2005)	SIRPT1–srX	36	3	0	3	0	2	0	0.81
c.10934T>C (p.L3645P)	c.7374delT (p.L2458fsX16)	Baets et al. (2010)	SIRPT1–srX	26	1	0	3	3	3	0	0.83
c.10958T>C (p.F3653S)	Δ (1.5 Mb macrodeletion)	Breckpot et al. (2008)	SIRPT3–srX	15	2	1	2	2	1	0	0.87
c.7121T>C (p.L2374S)	Δ (1.5 Mb macrodeletion)	Terracciano et al. (2009)	SIRPT2–sr3	26	1	1	2	2	1	0	0.73
c.3932T>A (p.M1311K)	c.3932T>A (p.M1311K)	Ouyang et al. (2008)	SIRPT2–sr3	17	2	1	1	2	1	0	0.69
c.12220G>C (p.A4074P)	c.12220G>C (p.A4074)	El Euch-Fayache et al. (2003), H'mida-Ben Brahim et al. (2011)	Interdomain between SIRPT3–sr3 and DnaJ	39	3	0	3	2	3	0	0.46
c.12992G>A (p.R4331Q)	c.5143A>T (p.K1715X)	Vermeer et al. (2008)	DnaJ	37	2	0	3	2	1	0	1.36
c.12991C>T (p.R4331W)	c.5719C>T (p.R1907X)	Prodi et al. (2012)	DnaJ	13	3	2	2	3	3	1	1.72
c.13405G>C (p.A4469P)	Δ (1.5 Mb macrodeletion)	Gazulla et al. (2012)	HEPN	18	2	1	3	3	1	0	0.94
c.13645A>G (p.N4549D)	c.13645A>G (p.N4549D)	Richter et al. (2004)	HEPN	11	3	0	3	3	2	1	1.77

del, base deletion (microdeletion); Δ, deletion (macrodeletion); fs, frameshift; ins, insertion; yrs, years.

a See also Table 1.

b Numbering based on the following NCBI Reference Sequences: GenBank acc. no. NM_014363.4, for nucleotide, and GenBank acc. n. NP_055178.3, for protein. DNA mutation numbering system in use is based on cDNA sequence (with a “c.” symbol before the number). Nucleotide numbering reflects cDNA numbering with +1 corresponding to the A of the ATG translation initiation codon in the reference sequence, with the initiation codon being codon 1. Amino-acid change numbering system in use is based on protein sequence (with a “p.” symbol before the letter) (http://www.hgvs.org). Original nucleotide and amino-acid positions and changes can be found in the original articles (see *Reference*).(*Continued*)

**Figure 4:**
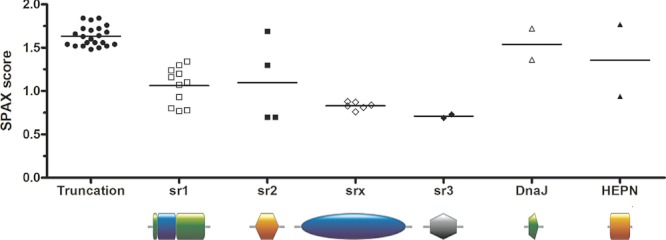
Composite SPAX (Spastic Ataxia) scores versus sacsin repeated domains. This scatter dot plot shows the assortment of SPAX scores from patients carrying a pathogenic missense mutation in *sr1*, *sr2*, *srX*, or *sr3* in homozygosis or heterozygosis with a frameshift mutation, a stop mutation or a macrodeletion (for details, see Table 3). SPAX scores from patients in which both alleles were predicted to generate truncated proteins (for the presence on both alleles of either a frameshift mutation or a stop mutation or a macrodeletion) were also represented (for details, see Supp. Table S6). For comparison, SPAX scores from patients carrying a pathogenic missense mutation in DnaJ or HEPN in homozygosis or heterozygosis with a frameshift mutation, a stop mutation or a macrodeletion were also drawn (for details, see Table 3). Within each category, the horizontal line indicates the calculated mean value.

As a way to define the maximal severity of the disease, in our analysis, we initially calculated SPAX scores from patients in which both alleles were predicted to generate truncated sacsins (due to presence on both alleles of either a frameshift mutation or a stop mutation or a macrodeletion; for a description of various combinations of alleles, please see Supp. Table S6). As it results from the analysis of Figure 4, these patients formed a homogeneous group that ranked at the highest SPAX scores among those calculated in this study (for comparison, see also Table 3), with values varying from 1.48 to 1.84. Conversely, when SPAX scores were calculated from patients carrying a pathogenic missense mutation in *sr1*, *sr2*, *srX*, or *sr3* (in homozygosis or heterozygosis with a frameshift mutation, a stop mutation or a macrodeletion, as described above), it was evident that the severity of the clinical phenotype largely varied (see Fig. 4 and Table 3) from values similar to those observed in patients carrying (a) truncated protein(s), for example, 1.69 for the c.1420C>T (p.R474C)/c.5719C>T (p.R1907X), which suggests nearly complete abolition of protein function, to significantly lower values, for example, 0.69 for c.3932T>A (p.M1311K)/c.3932T>A (M1311K), which suggests subsistence of partial or residual protein activity. Overall, the presence of the missense pathogenic mutations in *sr1*, *sr2*, *srX*, or *sr3* established on average a set of phenotypes (i.e., SPAX scores) significantly less severe (i.e., lower) than those observed for mutations that generated truncated proteins (ANOVA; *P* < 0.0001). We assumed that such a behavior correlated to the relevance that the domain in which the mutation falls had for sacsin activity. In particular, a trend to lower SPAX scores passing from *sr1* and *sr2* to *srX* and *sr3* could be observed, suggesting (1) that alterations in *srX* and *sr3* do cause less harmful, although measurable, effects on the function of the protein with respect to *sr1* and *sr2*, and thus (2) that *srX* and *sr3* play a “minority” role in the operational mechanism of the protein with respect to *sr1* and *sr2*.

## Discussion

In this study, a systematic inspection of vertebrate sacsins has been carried out to identify repeated domains along the protein. By using a combination of standard databank consulting tools and bioinformatics methods, three large (≥1,100 amino acids) repeated regions have been identified. Such internal repeats, named *SIRPT1*, *SIRPT2*, and *SIRPT3*, cover ∼84% of the protein sequence, and each contains three subrepeats, named *sr1*, *sr2*, and *sr3*, with *sr1* and *sr2* falling into Anderson et al. SRR supradomain [Anderson et al., 2010]. In addition, a fourth subrepeat, named *srX*, occurs in the first and the third internal repeat only, in a region between *sr2* and *sr3*. Our *SIRPT*-based architectural structure is invariably conserved in all vertebrate sacsins. This is not unexpected, as vertebrate sacsins share a high degree of similarity at both global and local level (this study), and most probably exert similar functional roles, a notion also supported by the observation that similar expression patterns can be found in both mammals [Engert et al., 2000; Parfitt et al., 2009] and fish (this study).

All the different subrepeats identified within the *SIRPT* architecture most likely represent regions involved in sacsin function. To answer this question, we have developed a strategy that combines very stringent alignments of the vertebrate sacsin domains with positional mapping of the human *SACS* mutations (for details, see *Results*). As a matter of fact, at least two pieces of evidence come out from our analyses indicating that the different subrepeats identified do represent functional regions. First, in *sr1*, *sr2*, *srX*, and *sr3*, missense pathogenic mutations are invariably over-represented in conserved positions with respect to missense nonpathogenic mutations. Second, missense pathogenic mutations are over-represented in *sr1*, *sr2*, *sr3*, and *srX* with respect to the regions between domains [Miller et al., 2003], this scheme being fully applicable also to the well-known DnaJ and HEPN domains. All together, these findings indicate that there is a strong tendency in the sacsin protein to gather the missense mutations associated with disease within the newly identified or the already known domains.

Sacsin is considered to operate in a chaperone-like manner, but very limited information is available on its activity, mainly due to the technical difficulties of managing with such an unusually long protein by means of standard biochemical, cellular, or molecular biology assays [Anderson et al., 2010; Kozlov et al., 2011; Parfitt et al., 2009). Under such circumstances, achieving information on the functional role(s) of our *sr1*, *sr2*, *srX*, and *sr3* domains represents a difficult task. In the effort to obtain new hints on the impact of the newly identified domains in the activity of the protein, we have developed a procedure that allows evaluation of the functional relevance of the domains by measuring the severity of the clinical phenotype, quantified in terms of SPAX score, in patients selected for carrying missense pathogenic mutations in *sr1*, *sr2*, *srX*, and *sr3* in homozygosity or heterozygosity with a null allele (for details, see *Results*). In spite of the limits of this experimental approach, essentially because of the so far limited number of patients composing each group, from our analysis it is evident that: (1) patients carrying missense pathogenic mutations in homozygosity or heterozygosity with a null allele exhibit significantly milder phenotypes, that is, lower SPAX scores, than patients carrying a null mutation on each allele (a condition that is predicted to fully abolish protein function; for details, see *Results*); (2) mean SPAX scores decrease passing from *sr1* to *sr3*, with *sr1* (1.06) = *sr2* (1.10) > *srX* (0.83) > *sr3* (0.71), which suggests that alterations in *srX* and *sr3* are less damaging in patients than those in *sr1* and *sr2*, and thus that *srX* and *sr3* play a less determinant role in the operational mechanism of the protein with respect to *sr1* and *sr2*. Nonetheless, we recognize that our data should be weighted cautiously and that additional determinants of severity might come out from future functional tests. In this context, it has to be underlined that our simplified approach cannot take into account in a simple way the yet possible contribution of the nature of the amino-acid substitution on the severity of the phenotype. Thus, we considered that the effect of an amino-acid substitution depends on the protein domain where the substitution falls and comes to operate rather independently of the nature of the mutation. That this may hold true comes from the observation that the same type of amino-acid change (see, e.g., R-to-C, that occurs thrice in *sr1* and once in *sr2*) may result in either high (in *sr2*) or medium-to-low (in *sr1*) SPAX scores (for details, see Table 3).

**Table:** Table 3. *Continued* Individual Items to Score Disease Severity

Score	Onset	Cognitive	Cerebellar^a^	Spasticity^b^	Peripheral neuropathy^c^	Retinal
0	Adult	Absent	Absent	Absent	Absent	Absent
1	Juvenile	Mild decline	Mild	Mild	Mild	No functional impairment but aware of worsened acuities
2	Teen	IQ lower than peers	Moderate	Moderate	Moderate	Reduced night vision
3	Early-onset	Marked mental retardation	Severe	Severe	Severe	Abnormal fundoscopy or ERG

IQ, intelligence quotient; ERG, electroretinogram.

*Note*: Total scoring is corrected for time of disease (yrs) under the assumption that disease severity worsen with disease duration, and it is expressed as percent.

a On the basis of SARA: Scale for the Assessment and Rating of Ataxia and IACRS (Inherited Ataxia Clinical Rating Scale).

b On the basis of SPRS: Spastic Paraplegia Rating Scale.

c On the basis of CMT (Charcot–Marie–Tooth) neuropathy score (second version).

Our results extend and refine the current knowledge on the organization of some sacsin domains. In particular, the *sr1* and *sr2* domains identified in this work substantially form the SRR supradomain recently defined by others [Anderson et al., 2010]. This supradomain is composed of an N-terminal portion (∼160 residues), which is homologous to the HATPase_c domain of Hsp90, and a C-terminal portion (∼200 residues), which consists of a novel sequence invariably connected to the HATPase_c domain [Anderson et al., 2010]. Our bioinformatics approach divides this SRR supradomain in two well-defined repeated domains, that is, *sr1* and *sr2*, which are separated by an evident nonrepeated linker segment. This organization is coherent with a system that works as an Hsp90-like protein. In fact, in Hsp90-type chaperones, the ATP binding domain is connected to the middle domain via a divergent linker region. In particular, in our sacsin organization, *sr1* represents the ATP binding domain and *sr2* the middle domain. Notably, in Hsp90 the middle domain invariably contains an arginine residue accepting phosphate after ATP hydrolysis [Pearl and Prodromou, 2006]. This phosphoacceptor arginine, already observed by Anderson et al. (2010) as invariably conserved in each C-terminal region of their SRR supradomains, does occur in each *sr2* domain. Interestingly, our study clearly demonstrates the crucial role of this arginine in the operational mechanism of sacsin. In fact, a mutation occurring on one of such conserved arginines, namely c.1420C>T (p.R474C) in *SIRPT1-sr2*, associates to one of the highest SPAX scores (1.69) found in this survey.

In this article, we report for the first time the occurrence of two novel repeated domains, namely *srX* and *sr3*, downstream the Hsp90-type regions discussed above. Such domains share no similarity to any domains reported so far in databanks, and no obvious role can be assigned to them. However, in the context of an Hsp90-like scheme of function, *srX* and/or *sr3*, located near the *sr1*/*sr2* “biochemical clamp” that allows ATP binding and hydrolysis, may participate (via dimerization, client binding, cochaperone interaction, regulation, etc.) to sacsin chaperone activity. In this respect, there has been recent demonstration that a large sacsin region (RegA), virtually corresponding to our *SIRPT1*, do exhibit a chaperone-like activity that can be detected in vitro by standard biochemical approaches [Anderson et al., 2011]. Such protein module is composed of the Hsp90-like region and of a large undefined downstream region. However, our study identifies *srX* and *sr3* as functional elements in that large undefined region and likely involved in the chaperone activity of the whole module.

In conclusion, we used a functional comparative genomics approach that combines bioinformatics sequence examination tools to mapping and phenotypical analysis of human mutations, to provide novel information on the organization in repeated domains of sacsin. In particular, our results establish that large portions of the protein can be arranged in a few and well-defined repeated domains. The demonstration of the functional nature of *sr1*, *sr2*, *srX*, and *sr3* suggests that these regions contribute to the activity of the protein. Further studies are needed to define the specific role(s) of such domains, in the perspective of developing a comprehensive and integrated model of function for sacsin in the context of cell pathophysiology. In a larger perspective, our approach that combines comparative analysis of vertebrate protein sequences/architecture, positional mapping of human mutations, and severity of clinical phenotype can be tentatively applied in the biomedical field to shed light on the functional nature of other proteins associated to disease but of yet unknown function.

## Supplementary material

Disclaimer: Supplementary materials have been peer-reviewed but not copyedited.
