# Supplementary material for: Comparative Analysis and Functional Mapping of SACS Mutations Reveal Novel Insights into Sacsin Repeated Architecture
Source: Hum Mutat. 2012 Dec 24;34(3):525–37. doi: 10.1002/humu.22269 (PMC3629688; doi:10.1002/humu.22269)
Supplement: Supplementary file 1 [file humu0034-0525-SD1.pdf]

Supporting Information for the article:

Comparative Analysis and Functional Mapping of *SACS* Mutations Reveal Novel Insights into Sacsin Repeated Architecture

Alessandro Romano, Alessandra Tessa, Amilcare Barca, Fabiana Fattori, Maria Fulvia de Leva, Alessandra Terracciano, Carlo Storelli, Filippo Maria Santorelli, Tiziano Verri

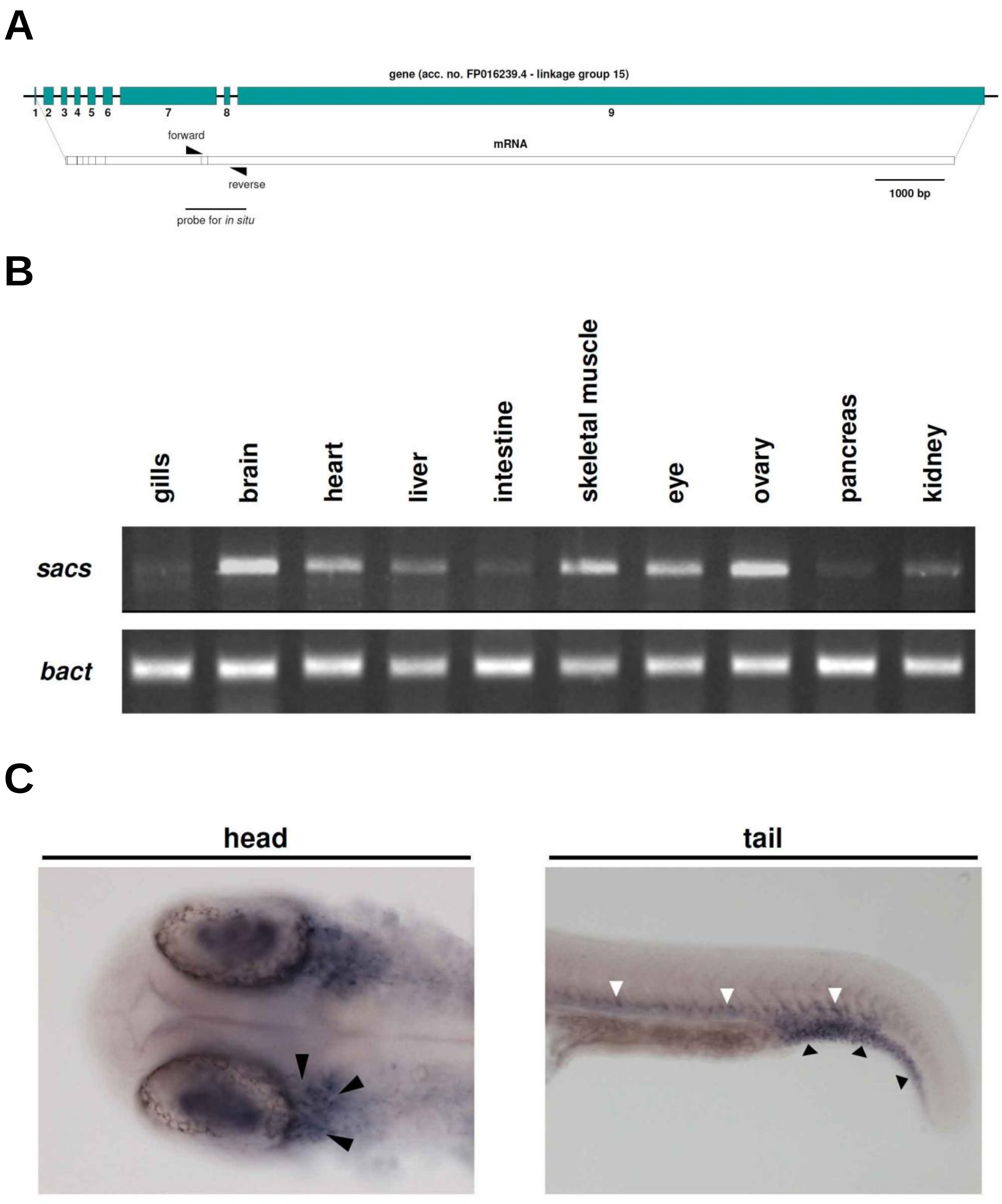

Supp. Figure S1. Basic characterization of zebrafish (*Danio rerio*) *sacs* gene and expression. **A:** Structure of the zebrafish *sacs* gene and its relationships to the related mRNA. Only coding exons are represented and numbered from 1 to 9 [Please note that the current description of the human *SACS* gene contemplates one additional non-coding exon upstream the 9 coding exons, so that the total number of exons for human *SACS* is 10. Non-coding exons have not yet been characterized in zebrafish *sacs*]. Exons are depicted as green vertical bars, widths drawn to scale. Introns are depicted as black lines, widths not drawn to scale. For details on the zebrafish *sacs* gene intron/exon

boundaries see Supp. Table S1. The relative contribution of each exon to the spliced mRNA transcript is shown. **B:** Sacsin mRNA expression pattern in zebrafish adult fish tissues as assessed by RT-PCR. Forward primer in putative exon 7 and reverse primer in putative exon 9 (see *sacs* gene structure in A); PCR product sizes were 890 bp for sacsin (PCR fragment cloned and sequenced) and 440 bp for β-actin (*bact*). **C:** Sacsin mRNA-targeted *in situ* hybridization in zebrafish embryos (24 hpf). Arrowheads in the head magnification show antisense probe hybridization in the head mesoderm/neural crest and in the midbrain regions; arrowheads in the tail magnification show hybridization in the myotomes (white) and in the ventral tail (black).

Essential notes on Methods used and Results shown in Supp. Figure S1

Sacsin is expressed in all vertebrates. To ascertain whether sacsin operates in all vertebrates, we chose to study the expression of the zebrafish *sacs* gene (for zebrafish *sacs* gene organization see Supp. Fig. S1A and Supp. Table S1). Zebrafish *sacs* mRNA was expressed in zebrafish tissues, both at the adult stage and during the embryonic development. In particular, as observed by RT-PCR, zebrafish *sacs* mRNA was found in the adult virtually in all tested tissues (i.e. gills, brain, heart, liver, intestine, muscle, eye, ovary, pancreas, kidney), although the highest expression levels were in brain, muscle, eye and ovary (Supp. Fig. S1B). Moreover, in 24 hours post-fertilization (24 hpf) embryos, both brain, eye, muscle (myotomes) and ventral tail exhibited detectable zebrafish *sacs* mRNA expression, as observed by whole-mount *in situ* hybridization analysis (Supp. Fig. S1C).

Zebrafish maintenance, breeding and embryo collection. Adult zebrafish were kept in captivity under optimal conditions for normal growth and breeding, and for the generation of viable embryos capable of carrying out development without morphogenetic alterations. Fish were bred by natural crosses in a male to female ratio of 2:1 [Westerfield, 1995]. Immediately after spawning, fertilized eggs were collected, washed and placed in Petri dishes in 0.6 mg/l Instant Ocean sea salts (Aquarium Systems, Sarrebourg, France). The developing embryos were incubated at 28.5 °C until use. Developmental stages of zebrafish embryos were expressed as hours post-fertilization (hpf) at 28.5 °C [Kimmel et al., 1995]. All experimental procedures were conducted according to the European Communities Council Directives, with all efforts to minimize animal sufferings and the number of animals necessary to collect reliable scientific data.

RT-PCR. Total RNA was extracted from tissues of adult animals (TRIzol Reagent; Invitrogen). RT was performed (50 °C/50 min) on 5 µg total RNA using the SuperScript III First-Strand Synthesis System for RT-PCR (Invitrogen) and oligo(dT)12-18. PCR was performed on 2 µl of the resulting cDNA using Platinum Taq DNA polymerase (Invitrogen) and zebrafish sacsin-specific primers (designed on the zebrafish DNA sequence from clone CH73-346G24, GenBank acc. no. FP016239.4; forward: 5'-AAGAGTGCTTGGAGAAGACTGG-3', starting at nucleotide 46,338; reverse: 5'-CGACTTGGACTCAAGCATGGAC-3', starting at nucleotide 53,294). After a denaturing step at 94 °C for 2 min, amplification was performed for 35 cycles (94 °C denaturation/40 s, 54 °C annealing/40 s, 72 °C extension/1 min; final synthesis: 72 °C/7 min) to obtain a predicted 890 bp long PCR product. Primers for zebrafish β-actin (GenBank acc. no. NM_131031.1; forward: 5'-CGTGACATCAAGGAGAAGCT-3', starting at nucleotide 681; reverse: 5'-ATCCACATCTGCTGGAAGGT-3', starting at nucleotide 1123; amplicon: 440 bp) were used as control to assess RNA quality and efficiency of the RT step. PCR products were separated on a 1.5% agarose gel and stained by ethidium bromide. Gel images were captured using the Gel-Doc 2000 gel documentation system equipped with a CCD camera (Bio-Rad Laboratories, Hercules, CA). PCR product identity was confirmed by cloning and sequencing.

Whole-mount in situ hybridization. A 890 bp fragment corresponding to a portion of zebrafish sacsin mRNA covering the final part of exon 7, exon 8 and the initial part of exon 9 was amplified (RT-PCR) from brain RNA using the following the above indicated sacsin-specific primers. The amplification product was subcloned into the pCRII-TOPO vector (Invitrogen) and the recombinant clone (zf_Sacs_probe-pCRII-TOPO) was sequenced to confirm the identity of the insert. The 890 bp digoxigenin (DIG)-11-UTP(Roche, Mannheim, Germany)-labeled antisense riboprobe was synthesized *in vitro* with T7 RNA polymerase (Roche) using the linearized (*Kpn I*-cleaved) zf_Sacs_probe-pCRII-TOPO as template. Whole-mount *in situ* hybridization was performed as described [Thisse et al., 1993] on 24 hpf zebrafish embryos. Stained embryos were mounted in glycerol and observed on an AZ100 Multizoom stereo microscope microscope equipped with Nomarski optics and a DS-U2 digital camera (Nikon Instruments, Calenzano, Italy).

Supp. Figure S2

```
                                                                    AA      A
human             -METKENRWVPVTVLPGCVGCRTVAALASWTVRDVKERIFAETGFPVSEQRLWRGGRELS 59
orangutan         -METKENRWVPVTVLPGCVGCRTVAALASWNVRDVKERIFAETGFPVSEQRLWRGGRELS 59
mouse             -METEETRWVRVTVLRGCVGCRTVAVPATATGRDLKERIFAETSFPVAEQRLWRGDREIP 59
rat               -MGTEETRWVRVTVLRGCVGCRTVAVRATATGRDLKERIFAETGFPVAEQRLWRGDREIP 59
dog               -METTDNRWLRVTVLPGCVGCRTVAARASWTVRDLKERILAETGFPASEQRLWLGGRELS 59
horse             -METKDNRYAEATVLHSLAGCSTVAVAASWTVRDVKERICAETGFPVSEQRLWLGDRELS 59
chicken           -------RLARVTILHGCLGCRTFELPPSADVGDVKARIDAEAGFPAARQRLWHCGRELS 53
zebra finch       MEIKDKNRLARVTVLHDCLGCRTFELPPSAAVRDVKR-IEAEAGLPAAEQRLWHRGRELS 59
anole lizard      -------RSATVTVLHDYMGCRTFEVPSSVSVGAIKELIYPETGFPVAEQRLCYQGRELS 53
fugu              MAGSPVPWLPWVMVHHDYIGLRSYQVSPFTSVHCVKQLLYEETHLPVEEQRLSHNGRRLD 60
tetraodon         MASLPAPWLPRVTVHHQYLGLRSYRLSPFTSVQRVKQLLYEETKLPVREQRLSHNGRQLD 60
stickleback       MTSCTDPWLPRVTVRHEYIGLRSYRVSPFTSVEDIKELLYEETNLPVKEQRLTHNGRLLD 60
medaka            MANCIDPWFPWVTVRHECIGPRSYRVSPVTSVQDVKQLLYEETNLPVKEQRLLHNGRILD 60
zebrafish         MATGQDPWLPWVIVSHDCLSTRVFQVPSSTVIRSIKTLIYEETDYPVSEQQLFHNGKMVH 60
                             . :     .       .      :*  :  *:  *. .*:*   .: :

                  A
human             DWIKIGDLT-SKNCHLFVNLQSKGLKGGGRFGQTTPPLVDFLKDILRRYPEGGQILKELI 118
orangutan         DWIKIGDLT-SKNCHLFVNLQSKGLKGGGRFGQTTPPLVDFLKDILRRYPEGGQILKELI 118
mouse             DWIKIGDLT-SKTCHLFVNLQSKGLKGGGRFGQTTPPLVDFLKDILRRYPEGGQILKELI 118
rat               DWIKIGDLT-SKTC-LFVNLQSKGLKGGGRFGQTTPPLVDFLKDILRRYPEGGQILKELI 117
dog               DWIKIGDLT-LKNHHLFVNLQSRGLKGGGRFGQTTPPLVDFLKDILRRYPEGGQILKELI 118
horse             DWIRIGNLT-SKDRHLFVNLQSKGLKGGGRFGQTTPPLVDFLKDILRRYPEGGQILKELI 118
chicken           DDTKIVDLQ-KSQNQIFLKLQSEALKGGGRFGQTTPPLVDFLKDILRRYPEGGQILKELI 112
zebra finch       DAIKIGDLQ-KSQNQVFLELQSKGLKGGGRFGQTTPPLVDFLKDILRRYPEGGQILKELI 118
anole lizard      DSVKVEDLQ-TSQNHVFLHLLSKGLKGGGRFGQTTPPLVDFLKDILRRYPEGGQILKELI 112
fugu              DRVQIGTLVPPGTPEVSFTLAGGGLKGGGRFGQTTPPLVEFLKDILRRYPEGGQILKELI 120
tetraodon         DGVQIGSLVPSGAAEVSLTLAGGGLRGGGRFGQTTPPLVEFLKDILRRYPEGGQILKELI 120
stickleback       DSVQIRTLVPAGSPEVSVALEGSGLKGGGRFGQTTPPLVDFLKDILRRYPEGGQILKELI 120
medaka            DSIQIGTLAPAGSSHVCVTLEGRGLKGGGRFGQTTPPLVEFLKDILRRYPEGGQILKELI 120
zebrafish         DGVEIGNLVPHGDHEIIMTLHGRGPRGGGRFGQTTPPLVDFLKDILRRYPEGGQILKELI 120
                  *  .:  *       : . * . . :*************:********************

                                                                   Y
                                                             T     Y
human             QNAEDAGATEVKFLYDETQYGTETLWSKDMAPYQGPALYVVYNNAVFTPEDWHGIQEIARS 178
orangutan         QNAEDAGATEVKFLYDETQYGTETLWSKDMAPYQGPALYVVYNNAVFTPEDWHGIQEIARS 178
mouse             QNAEDAGATEVKFLYDETQYGTETLWSKDMAQYQGSALYVVYNNAVFTPEDWHGIQEIARS 178
rat               QNAEDAGATEVKFLYDETQYGTETLWSKDMAQYQGSALYVVYNNAVFTPEDWHGIQEIARS 177
dog               QNAEDAGATEVKFLYDETQYGTETLWSKDMAQYQGPALYVVYNNAVFTPEDWHGIQEIARS 178
horse             QNAEDAGATEVKFLYDETQYGTETLWSKDMAQYQGSALYVVYNNAVFTPEDWHGIQEIARS 178
chicken           QNAEDAGATEVRFLYDETQYGTETLWSKDMAQYQGPAFYAYNDAIFTPEDWHGIQEIARS 172
zebra finch       QNAEDAGATEVRFLYDETQYGTETLWSKDMAQYQGPAFYAYNDAVFTPEDWHGIQEIARS 178
anole lizard      QNAEDAGATEVRFLYDETQYGNESLWSKDMAQYQGSALYVVYNNAIFTPEDWHGIQEIARS 172
fugu              QNAEDAGATEVKFLFDETEYGVESLWSPDMEQHQGTALYAYNDAVFTVEDWNGIQEIARS 180
tetraodon         QNAEDAGATEVKFMYDETEHGVESLWSPDLEQHQGTALYAYNDAVFTLEDWSGIQEIARS 180
stickleback       QNAEDAGATEVKFMYDETEYGVESLWSPDMAQYQGTALYVVYNDAVFTEEDWNGIQEIARS 180
medaka            QNAEDAGATEVRFMYDETEYGVESLWSPDMAQHQGAAMYVVYNNAVFTGEDWNGIQEIARS 180
zebrafish         QNAEDAGATEVKFLYDETEYGVESLWSHDMAQYQGTALYVVYNDAVFTTEDWNGIQEIARS 180
                  ***********:*::***::* *:*** *:  :**.*:*.**:*:** *** ********

                                        K                              K
human             RKKDDPLKVGRFGIGFNSVYHITDVPCIFSGDQIGMLDPHQTLFGPHESGQCWNLKDDSK 238
orangutan         RKKDDPLKVGRFGIGFNSVYHITDVPCIFSGDQIGMLDPHQTLFGPHESGQCWNLKDDSK 238
mouse             RKKDDPLKVGRFGIGFNSVYHITDVPCIFSGDQIGMLDPHQTLFGPHESGQCWNLKDDIK 238
rat               RKKDDPLKVGRFGIGFNSVYHITDVPCIFSGDQIGMLDPHQTLFGPHESGQCWNLKDDIK 237
dog               RKKDDPLKVGRFGIGFNSVYHITDVPCIFSGDQIGMLDPHQTLFGPHESGQCWNLKDDSK 238
horse             RKKDDPLKVGRFGIGFNSVYHITDVPCIFSGDQIGMLDPHQTLFGPHESGQCWNLKDDSK 238
chicken           RKKDDPLKVGRFGIGFNSVYHITDVPSIFSGDQIGMLDPHQTLFGPHESGQCWNLKEDSK 232
zebra finch       RKKDDPLKVGRFGIGFNSVYHITDVPSIFSGDQIGMLDPHQTLFGPHESGQCWNLKEDSK 238
anole lizard      RKKDDPLKVGRFGIGFNSVYHITDVPSIFSGDQIGMLDPHQTLFGPHESGQCWNLKDDFK 232
fugu              RKRDDPLKVGRFGIGFNSVYHVTDVPSIFSGDQIAMLDPHQTLFGVHESGQCWNMKTDIK 240
tetraodon         RKRDDPLKVGRFGIGFNSVYHVTDVPSIFSGDQIAMLDPHQTLFGPHESGQCWNLKTDTK 240
stickleback       RKREDPLKVGRFGIGFNSVYHITDAPSIFSGDQIAMLDPHQTLFGVHESGQCWNLKTDMK 240
medaka            RKRDDPLKVGRFGIGFNSVYHITDVPSIFSGNQIAMLDPHQTLFGVNESGQCWNLSTDRK 240
zebrafish         RKREDPLKVGRFGIGFNSVYHITDVPSIFSGDQIAMLDPHQMLFGVHESGQCWNLKSDIK 240
                  **::*****************:**.*.****:**.****** *** :*******:. * *

                                                   C
                                                   H   C X
human             EISELSDQFAPFVGIFGSTKETFINGNFPGTFFRFPLRLQPSQLSSNLYNKQKVLELFES 298
orangutan         EISELSDQFAPFVGIFGSTKETFINGNFPGTFFRFPLRLQPSQLSSNLYNKQKVLELFES 298
mouse             EINELPDQFAPFIGVFGSTKETFTNGSFPGTFFRFPLRLQPSQLSSNLYTKQKVLELFDS 298
rat               EINELPDQFAPFIGVFGSTKETFTNGSFPGTFFRFPLRLQPSQLSSNLYTKQKVLELFDS 297
dog               EISELSDQFAPFIGIFGSTKETFVNANFPGTFFRFPLRLQPSQLSSNLYNKQKVLELFDS 298
horse             EISELSDQFAPFIGIFGSTKEMFVNGNFPGTFFRFPLRLQPSQLSSNLYNKQKVLELFES 298
chicken           EINELTDQFAPFIGVFGSTKETFKNGNFPGTFFRFPLRLQPSQLSSNVYDKQKVLELFES 292
zebra finch       EINELTDQFAPFIGVFGSTKETFKNGNFPGTFFRFPLRLQPSQLSSNVYDKQKVLELFES 298
anole lizard      EMNELTDQFAPFIGVFGSTKETFQNGHFPGTFFRFPLREQPSQLSSNIYNKEKVLELFDS 292
fugu              EITELSDQFAPYFGIFGILEKNIKEANFPGTLFRFPLRLKPSQLSSNIYNKEKVLELFES 300
tetraodon         EIAELSDQFAPYFGIFGISEKHMKDASFPGTLFRFPLRLRPSQLSSNVYNKEKVLELFES 300
stickleback       EITELADQFTPYFGIFGSSEKTVKDGSFPGTLFRFPLRLKPSQLSGNIYNKEKVLELFES 300
medaka            EITELADQFSPYMGMFGISEKTIKDGSFLGTLFRFPLRMKPSQLSGNIYNKEKVLELFES 300
zebrafish         EITELSDQFSPYFGLLGSSEKTIKDGNFPGTLFRFPLRMKPSQLSNNIYNKEKVLELFES 300
                  *: **.***:*:.*::*  :: . :. * **:****** :*****.*:* *:******:*
```

```
                                 F           X   R
human            FRADADTVLLFLKSVQDVSLYVREADGTEKLVFRVTSSESKALKHERPNSIKILGTAISN 358
orangutan        FRADADTVLLFLKSVQDVSLYVREGDGTEKLVFRVTSSESKALKHERPNSIKILGTAISN 358
mouse            FRADADTVLLFLKSVQAVSLHVREADGTEKLVFRVTASENKALKHERPNSIKILGTAISN 358
rat              FRADADTVLLFLKSVQAVSLHVREADGTEKLVFRVTASENKTLKHERPNSIKILGTAISN 357
dog              FRADADTVLLFLKSVQDVSLHVREADGTEKLVFRVTASENKALKHERPNSVKILETAISN 358
horse            FRADADTVLLFLKSVQDVSLHVREADGTEKLMFRVTASESTALKQERLNSVKILGTAISN 358
chicken          FRADADTVLLFLKSVQDVSLHVREADGTEKLIFRVTASENKALKHERPNSIKILGTAINQ 352
zebra finch      FRTDADTVLLFLKSVQDVSLHVREADGTERLVFRVTASENKALKHERPNSIKILGTAINQ 358
anole lizard     FRADADTVLLFLKSVQDVSLHVREADGTERLIFRVTASENKALKHEKPNSIKILGSSINQ 352
fugu             FKTDADTVLLFLKSVNKISLHVRESDGTERMLFQVTSAGNAEDKLERPNALKMLGQAVDS 360
tetraodon        FKADADTVLLFLKSVNRISLHVRESDGTERMLFQVTASGNAEDELERPNALKTLGQAVDG 360
stickleback      FKADADTVLLFLKSVQKISLHVRECDGTERMLFQVTATENTEDKEERPNALNTLGEAIDG 360
medaka           FKADANTVLLFLKSVQKISLHVREPDGTERMLFQATATENPDNKLERPNALETLGQAIDN 360
zebrafish        FKVDADTVLLFLKSVQKVSLHIRESDGTERMLFQVTASDSQEDRMERPNSLKTLGQAIDS 360
                 *:.**:*********: :**::** ****:::*:.*:: .   . *: *::: *  ::.

                                                          D
human            YCKKTPSNNITCVTYHVNIVLEEESTKDAQKTSWLVCNSVGGRGISSKLDSLADELKFVP 418
orangutan        YCKKTPSSDITCVTYHVNIVLEEESTKDAQKTSWLVCNSVGGRGISGKLDSLADELKFVP 418
mouse            YCKKIPSNSVTCVTYHINIVLEDESTKDAQKTSWLVCNSVGGRGISSKLDSLADELKFVP 418
rat              YCKKIPSNSVTCVTYHINIALEDESTKDAQKTSWLVCNSVGGRGISSKLDSLADELKFVP 417
dog              YCKKIPSNSITCVTYHINIVLEDESTKDAQKTSWLVCNSVGGRGISGKLDSLADELKFVP 418
horse            YCKKIPSDSVTCVTYHINIVLEDESTKDTQKTSWLVCNSVGGRGVSSALDSLADELKFVP 418
chicken          YCKGVPSNSITCVTYHVNIALEDESVKDAQKTSWLVCNCVGGRGMCTELDCLADDLKFVP 412
zebra finch      YCKGVPSNSITCVTYHVNIVVEDESVKDAQKTSWLVCNCVGGRGMCTELDCLADDLKFVP 418
anole lizard     YCKGIPSNSVTCVTYHINIVLEDESIKDAQKTSWLVCNSVGGRGMCSDLDCLADDLKYIP 412
fugu             YNNGVPSSTITCVTYQVNIDTQDKTAKETQRTTWLVTNGVGGRGMCAELDSLADDLKFMP 420
tetraodon        YSNGVPSSTVTCVTYQLNVDTQDGAAKETQRTAWLVSNGVGGRGLCAELDALADDLKFTP 420
stickleback      YGNGVPSSTITCVTYQVSIETQDETAKETQRMTWLVCNGVGGRGMCPELDSLADVLKFMP 420
medaka           YSNRVPSSTITCVTYQVNIETQDETAKEANRTMWLVCSGVGGRGMCAELDSLADNLKFMP 420
zebrafish        YSNGVPSSSVTCATYQLSIEARDETAKETQKTTWLVCNSVGGRGMCGELDSLADDLKFIP 420
                 * :   **. :**.**::.:  .: : *::::  *** . *****:.  **.*** **: *

                                                             I
                                                             R
                                                             K               C
human            IIGIAMPLSSRDDEAKGATSDFSGKAFCFLPLPPGEESSTGLPVHISGFFGLTDNRRSIK 478
orangutan        IIGIAMPLSSRDDEAKGATSDFSGKAFCFLPLPPGEESSTGLPVHISGFFGLTDNRRSIK 478
mouse            IIGLAMPLSGKDEE-NGAISDFSGKAFCFLPLPPGEESRTGLPVHISGFFGLTDNRRSIK 477
rat              IIGLAMSLSGRDEE-NGATSDFSGKAFCFLPLPPGEESRTGLPVHISGFFGLTDNRRSIK 476
dog              IIGIAMSLS-RDDEEKGATADFSGKAFCFLPLPPGEESKTGLPVHISGFFGLTDNRRSIK 477
horse            IIGIAMPLSSRDDEEKGATSDFSGKAFCFLPLPPGEESKTGLPVHISGFFGLTDNRRSIK 478
chicken          TIGIAMPLS-YNEKDKGAVADFSGRAFCFLPLPPGEESKTGLPVHVSGFFGLTDNRRSIK 471
zebra finch      TIGIAMSLA-ANEEENGAVADFSGRAFCFLPLPPGEESKTGLPVHVSGFFGLTDNRRSIK 477
anole lizard     TIGIAMSLS-SDGEGKGAAAEFSGRAFCFLPLPPGEESKTGLPVHVSGFFGLTDNRRSIK 471
fugu             TIGIALPLTVINKDDEGATSGFSGRAFCFLPLPPGEESETGLPVHVSGFFGLTDNRRSIK 480
tetraodon        TIGIALPLAVTGKDEQGATSGFSGRAFCFLPLPPGEESQTGLPVHVSGFFGLTDNRRSIK 480
stickleback      TIGIALPLTVVNKGDTGATSGFSGRAFCFLPLPPGEESETGLPVHLSGFFGLTDNRRSIK 480
medaka           IIGIALPLAANNQEDKGAASGFSGRAFCFLPLPPGEESETGLPVHVSGFFGLTDNRRSIK 480
zebrafish        TIGIALPLTLID-EDKGATSSFSGRAFCFLPLPLGEESMTGLPVHVSGFFGLTDNRRSIK 479
                  **:*:.*:   .     ** : ***:******** **** ******:**************

                                 X                      M                   L
human            WRELDQWRDPAALWNEFLVMNVVPKAYATLILDSIKRLEMEKSSDFPLSVDVIYKLWPEA 538
orangutan        WRELDQWRDPAALWNEFLVMNVVPKAYATLILDSIKRLETEKSSDFPLSVDVIYKLWPEA 538
mouse            WRELDQWRDPAALWNEYLIVNVVPKTYATLILDSIKRLETEKSSDFPLSVDTIYKLWPEA 537
rat              WRELDQWRDPAALWNQFLIVNVVPKAYATLILDSIKRLETEKSSDFPLSVDAIYKLWPEA 536
dog              WRELDQWRDPAALWNEFLVMTVVPKAYATLILDSIKRLETERSSDFPLSVDLIYKLWPDV 537
horse            WRELDQWRDPAALWNEFLVVNVVPKAYATLILDSIKRLETERSSDFPFSVDVIYRLWPDV 538
chicken          WRELDQWRDPAALWNDLLVVNIVPKAYSTLILEAIKRMETEENSDFPLSAERIYGLWPDE 531
zebra finch      WRELDQWRDPAALWNDLLVVNIVPKAYTTLILEAIKRMETEKNSDFPLSPERIYRLWPDE 537
anole lizard     WRELDQWRDPAALWNELLVTNMVPKAYATLILEAIKRMETDENSDFPLSADRIYRLWPQK 531
fugu             WREVDQWRDPAALWNELLLITVIPRAYLMLITEYVQRVQTKKDQDFPLTPTGTYGAWPNP 540
tetraodon        WREVDQWRDPAAVWNQLLLVTVIPRAYLVLITEAIQRVQRRKDQDFPLSPMGTYGAWPNP 540
stickleback      WREVDQWRDPAALWNELLIITVIPWAYFTLITEAIRRVQTQQDQDFPLSPAGTYGAWPDP 540
medaka           WREVDQWRDLAALWNELLIVIVIPRAYFTLIIDAIKRVQTQKDQDFPLSPAGTYRAWPDP 540
zebrafish        WREVDQWRDPAALWNELLIVTVIPRAYFTLIMETIQRIQTKKDQDFPLSPRGIYGAWPDP 539
                 ***:***** **:**: *:   ::* :*  ** : ::*::  ...***::    *  **:

                                       P
human            SKVKVHWQPVLEPLFSELLQNAVIYSISCDWVRLEQVYFSELDENLEYTKTVLNYLQSSG 598
orangutan        SKVKVHWQPVLEPLFSELLQHAVIYSISHEWVRLEQVYFSELDENLEYTKTVLNYLQSSG 598
mouse            SKVKAHWHPVLGPLFSELFQHAVIYSIGGEWVKLEQVHFSELDGSLESTRSVLNYLQSSG 597
rat              SKVKAHWHPVLGPLFSELFQHAVIYSVGGEWVKLEQVHFSELDESLESTQSVLNYLQRSG 596
dog              SKVRVHWQPVLEPLFNELFQNAVMYSLSNQWVKLEQAYFSELDESWECTKSVLSYLQSSG 597
horse            NKVRVHWQRVLEPLFNELFQNAVLYSISNHWVQLEQAYFSELDDSLEYTETVLNYLQSSG 598
chicken          NKIRVPWKPIVVPLFKELLQHTVIYSVSNQWMKVEQVHFSEMDESLEYTQSVLNYLQNSG 591
zebra finch      NKIRVPWKPIVVPLFKDLLQHPVIYSVSNQWIKVEQVYFSEMDESLEYTQSVLNYLQNSG 597
anole lizard     NKIKVHWESIIEPLLKELFQNEVIYSISGKWIKVEEVYFSEMDESLEYTQAVLNYLQDSG 591
fugu             NLVKSRWKPILQPLFHELLQLPVIYSLCEKWVQADEVVFSELDMDEDISKIVINYLQNSG 600
tetraodon        GQVRSRWKPILQPLFQELLRQPVIFSLCESWVQADQAVFSELDPDQDTSEAVIAYLQKSG 600
stickleback      KRVKSRWKPILQPLFHDLLQQQVIHSLSESWIRVDQAVFSELNTDEDISETVISYLQSSG 600
medaka           KQVKSRWMPILQPLFQELLQQKVIYSLSESWVGMDHAVFSELDMDENLSETVINYLQSTG 600
zebrafish        NRIRPRWKPILEPLLNDLLQHPVIYSLSGSWVQVDETVFSDLDSSMDTAGTIIKYLQTSG 599
                   ::  *  :: **: *:*::  *:.*:   *:  :.. **::: . : :  :: *** :*
```

```
                                      P                 W
human               KQIAKVPGNVDAAVQLTAAS---GTTPVRKVTPAWVRQVLRKCAHLGCAEEKLHLLEFVL 655
orangutan           KQIAKVPGNVDAAVQLTAAS---GTTPVRKVTPAWVRQVLRKCAHLGCAEEKLHLLEFVL 655
mouse               KQIAKVPGNLAAAVQLSAASATSSASPVRKVTPAWVRQVLRKCAHLGSAEEKLHLLEFVL 657
rat                 KQIARVPGNLAAAVQLSAASATSTASPVRKVTPVWVRQVLRKCAHLGSAEEKLHLLEFVL 656
dog                 KHIVKVPPNLAAAVQLAAS----SAKPVRKVTPAWVRQVLRKSAHPGGAQDKLHLLEFVL 653
horse               KQIVKVPANLAAAVELAAP----GAA--RKVTPAWVRQVLRKSAPEGGARERLHLLEFVL 652
chicken             KQIAKVPANIASAVHFTTCT----VKAVKKVTPAVVRQVLRKSGHSGPAEEKLHLLQFVL 647
zebra finch         KQIAKVPANIANAVHLTIST----SKAVKKVTPAVVRQVLRKSGHSGPAEEKLNLLEFVL 653
anole lizard        KEVAKVPDNIAKAIDLIVYS----TKPVKKVTPGTVRQVLRKSGHKGPADAKLHILDFVL 647
fugu                VQVAKVPISVDVVLASYMAE----ASEVKKVRPPLLRQVIRKNKHKGSSQEKLLLLEFVL 656
tetraodon           VQVAQVPASVDAVLATYMT-------EVKKVTPSLLRQAMRRSKHKGSSQEKLSLLEFVL 653
stickleback         TQVAKVPAATDFVFAACTTE----SIEVRKVTPSLLRQVIRKCKHKGPSQEKLLLLEFAL 656
medaka              IQVAKVPAAVHSVLASYTTE----PFEVRTVTPSLLRQTVRKYTHKGPAQEKLLLLEFAL 656
zebrafish           MMLAQVPAYIDEALTMFVTK----PGSLRRVTPSFVRQTLRKCRHKGPSTEKLLLLEFIL 655
                      :.:**     ..              : * *  :**.:*:    * :  :* :*:* *

                                                          T
human               SDQAYSELLGLELLPLQNGNFVPFSSSVSDQDVIYITSAEYPRSLFPSLEGRFILDNLKP 715
orangutan           SDQAYSELLGLELLPLQNGNFVPFSSSVSDQDVIYITSAEYPRSLFPSLEGRFILDNLKP 715
mouse               SDQAYSELLGLELLPLQSGAFVPFSSSVSDQDVVYITSEEFPRSLFPGLEARLILENLKP 717
rat                 SDQAYSELLGLELLPLQSGAFVPFSSSVSDQDVVYITSEEFPRSLFPGLEARLILENLKP 716
dog                 SDQAYSELLGLELLPLQNGNFVPFSSSVSDQDVIYITSEDYPRSLFPGLEGRFILDSLKP 713
horse               SDEAYSELLGLELLPLQNGHFVPFSSSVSDQDVVYITSEDYPRSLFPGLEGRFILDNLKP 712
chicken             SDGVYSELIGLELLPLQNGNFISFSSSVSEQDIVYITSEDFPRSLFPGLEGRLLSDDLKP 707
zebra finch         SDGVYSELIGLELLPLQNGSFIPFSSSVSEQDVVYITSEEYPRSLFPGLEGRLLSDDLKP 713
anole lizard        SDGNYNELIGLELLPLQNGSFTLFSSSASDQDAVYITSESYPRSLFPGFEGRLLADDVKP 707
fugu                SDANYSDLIGLELLPLQDETFTSFSSPVSEKDAIYITSEEYPRFLYPGLEGRFILESIKS 716
tetraodon           ADANYSDLIGLELLPLQDETFASFASPVSDKDSVYMASEEYPRCLYPGLEARFVLESIKP 713
stickleback         SDGNYSDLIGLELLPLQDGTFTTFSSSFSDKDSIYIASEQYPKSLYPGLEGRFLLESIKP 716
medaka              SDANYSDLIGLELLPLQDKTFATFSSSVSEKDAVYIPSEDYPRVLYPGLEHRFILESIKP 716
zebrafish           SDSCYNDLIGLELLPLQDETFAVFSSSVNDKDAVYIASQEYPRSLYPGLEGRFVLESIPP 715
                    :*  *.:*:********.  *  *:*. .::* :*:.* .:*: *:*.:*  *:: :.: .

                                SX            X
human               HLVAALKEAAQTRGRPCTQLQLLNPERFARLIKEVMNTFWPGRELIVQWYPFDENRNHPS 775
orangutan           HLVAALKEAAQTRGRPCTQLQFLNPERFARLVKEVMNTFWPGRELIVQWYPFDENRNHPS 775
mouse               HLLAALKEAAQTRGRPCTQLQLLNPERFARLIKEVMNTFWPGRELVVQWYPFSEDKRHPS 777
rat                 HLLAALKEAAQTRGRPCTQLQLLNPERFARLIKEVMNTFWPGRELVVQWYPLSEDKHHPS 776
dog                 HLIAALKEAAQTRGRPCTQLQLLNPERFARLIKEVMNTFWPGRELIVQWYPFDEDKNHPS 773
horse               HLLAALKEAAQTRGRPCTQLQLLNPERFARLIKEAMNTFWPGRELIVQWYPCDKDKRHPS 772
chicken             EVLAALKEAAKSRGRPCTQLQLLNPERFTRLIKEVMNSVWPGRDMVVQWYPGLEDKNHPS 767
zebra finch         EVLAALKEAAKSRGRPCTQLQLLNPERFARLVKEVMNSVWPGRDVVVQWYPGLEEKKHPS 773
anole lizard        HILAALKEAAKSRGRPCTQLQLLNPERFARLIKEVMNALWPRREIVVQWFPLSEDKSQPP 767
fugu                SVMDSLKKAAKSRGRPCTQLQMLSPERSARLIKDILSSVWPTRDFTVEWEPGNRELKHPT 776
tetraodon           SVKDGLMKAAKSRGRPCTQLQVLSPERSARLIKDVLSSAWPTRDFAVEWEPGNRELRHPT 773
stickleback         LVMDALKEAAKSRGRPCTQLQMLNSERSARLIKEILSTAWLSRDFIVRWEPGNKELKHPT 776
medaka              SVMDSLKEAANSRGRPCTQLQELNPERSARLIKEILSTAWTTREFSVKWEPGNGELKHPT 776
zebrafish           HVMTSLKEAAKSRGRPCTQVQVLTPERSARLIKEVLTTAWPSRDFAVQWNPGDQEKKHPS 775
                     :  .* :**::*******:* *..** :**:*: :.: *  *:. *.* *   :  :*.

                                          P
human               VSWLKMVWKNLYIHFSEDLTLFDEMPLIPRTILEEGQTCVELIRLRIPSLVILDDESEAQ 835
orangutan           VSWLKMVWKNLYIHFSEDLTLFDEMPLIPRTILEEGQTCVELIRLRIPSLVILDDESEAQ 835
mouse               LSWLKMVWKNLYIHFSEDLTLFDEMPLIPRTLLNEDQTCVELIRLRIPSVVILDDETEAQ 837
rat                 ISWLKMVWKNLYIHFSEDLTLFDEMPLIPRTPLDADQTCVELIRLRVPSVVILDDETEAQ 836
dog                 VSWLKMVWKNLYIHFSEDLTLFDEMPLIPRTTLEEGQTCMELIRLRIPSLVILDDESEAQ 833
horse               VSWLKMVWKNLYIHFSEDLTLFDEMPLIPRTALEEGQMCVELIRLRIPSLVILDDESEAQ 832
chicken             ISWLKMVWKNLYMHFSDDLSAFDDMPLIPKTLLEENQTSLELVRFRNPSPVILEDESETQ 827
zebra finch         VSWLKMVWKNLYIHFSDDLSVFDEMPLIPRTLLEENQTSVELVRFKNPSPIILEDESETQ 833
anole lizard        VSWLKMVWKNLYLHFSGDLSMFDDMPLLPKTLLDEGEEIVELIRLRTPSPIILDDGLETQ 827
fugu                ISWLRMIWKHLYIHFSDDLSTFEDMPLIPLVPLEESMNSVHLLRLRTPSAIILVEGEETT 836
tetraodon           LSWLRMVWKHLYIHFAEDLSTFEDMPLIPLVPLEENMTRVHLLRLRTPSPIIFADAEEAA 833
stickleback         VSWLKRIWKHLYIHFADDLSTFEDMPLIPLVPLEESMEVAQLLRLKTPSPVILLDEEESS 836
medaka              VSWLKMIWRHLYIHFADDLYTFEDMPLIPLVPLDESMNNITLLRLKSPSPIILIDDKQAP 836
zebrafish           ASWLRMIWKHLYINFADDLSVFEDLPLIPNVPLADTLDCLELLRLKSPSPIVLVNEEEGP 835
                     ***: :*::**::*: **  *:::**:* . *        *:*:: ** ::: :  :

                                        K
human               LPEFLADIVQKLGGFVLKKLDASIQHPLIKKYIHSPLPSAVLQIMEKMPLQKLCNQITSL 895
orangutan           LPEFLADVVQKLGGFVLKKLDASIQHPLIKKYIHSPLPSAVLQIMEKMPLQKLCNQITSL 895
mouse               LPEFLADIVQKLGGIVLKRLDTSIQHPLVKKYIHSPLPSAILQIMEKIPLQKLCNQIASL 897
rat                 LPEFLADIVQKLGGIVLKRLDTSLQHPLVKKYIHSPLPSAILQIMEKIPLQKLCNQIASL 896
dog                 LPEFLADIVQKLGGIVLKKLDASIQHPLIKKYIHSPLPSAVLQIMEKMPLQKLCNQIASL 893
horse               LPEFLADIVQKLGGIILKKLDASIQHPLIKKYINSPLPSAVLQIMEKMPLQKLCNQIASL 892
chicken             LPDYIADIIEKVGGVVLKKLDVSIQHPLIKKYVHPPLPSAVLQIMEKVSLQKLCNQVSSL 887
zebra finch         LPEYLADIIQKIGGVVLKKLDISIQHPLIKKYVHPPLPSAVLQIMEKMSLQKLCNQVASF 893
anole lizard        LPEFLPDIIEQLGGIVIKKLDPAIQHPLLKKYIHAPLPSVVLQIVEKLSLQKLTSQITSF 887
fugu                SSGILLDIMEKLGGTVMKKLDSCLQHPLLKNYIHPSSPAVLLQIMDRLSKQRLSSQVTSL 896
tetraodon           PSGNLLDIMEKLGGTVMTKLDSCLQHPLLKNYIHPSSPAALLQIMDRLSKQRLASQVSSF 893
stickleback         LPENLVDIMEKLGGVVIKKLDSSLQHPQLKNFVHPSSPGLLLQIMDRLPTQRLSGLVSSF 896
medaka              FSENLLEVVEKLGGVAMKKMDPCLQHPLLKKYIHPCSPSILLQIMGRQSTQRLSSQVSSL 896
zebrafish           PSEDLLEVMKKLGCVILKQIDPCLHHPLLKNYIHQSSPSTLLQIMDRSTSQRLASQVSSL 895
                     .  : ::::::*   :.::* .::** :*:::: *. :***: : . *:* . ::*:
```

```
                                                          E
                                                          K
human             LPTHKDALRKFLASLTDSSEKEKRIIQELAIFKRINHSSDQGISSYTKLKGCKVLHHTAK 955
orangutan         LPTHKDALRKFLASLTDSSEKEKRIIQELAIFKRINHSSDQGISSYTKLKGCKVLHHTAK 955
mouse             LPTHKDALRKFLASLTDTSEKEKRIIQELTIFKRINHSSDQGISSYTKLKGCKVLDHTAK 957
rat               LPTHKDALRKFLASLTDTSEKEKRIIQELTIFKRINHSSDQGISSYTKLKGCKVLDHTAK 956
dog               LPTHKDALRKFLASLTDSSEKEKRIIQELPIFKRINHLSDQGISSYTKLKGCKVLHHTAK 953
horse             LPTHKDALRKFLAGLTDSSEKEKRIIQELTIFKRVNHSSGQGLSSYTKLKGCKVLHHTAK 952
chicken           PSTHKDALRAFLASLNDVSEKEGRIIQELLIFKKMEKSSDDGVPVYAGLNGSKVLHHTAK 947
zebra finch       PPTHKDALRAFLASLTDANEKEKRIIQELLMFKKVEKSSDESVPVYTGLNGSKVLHHTAK 953
anole lizard      STSYKNALRSYLASLTDITEKEKKVIHELVIFRRISQSLDDDIAFTS-LKSCKVLHHTAK 946
fugu              SITEKIALRKYLAGLSDVTEREKHTLLELSIFEKFG-TSCEGTSKFTSLRGARALHHRAK 955
tetraodon         SLTEKIALRKYLAGLSDVTEREKHTLLELSVFEKVG-SSCEGSSRFTSLRGARALHHRAK 952
stickleback       SVKEKVALRNFLAGLSDITAREKHTLLELSIFEKVG-TCSEGTSAFTSLRGARALHHRAK 955
medaka            SDKEKTALRNYLAGLPDITEKEKHTLLDLSIFDKVG-GFREGSPVFTSLRGARALHHRAK 955
zebrafish         TVKQRLALRSFLAGLTDVTEKEKRIIQELPIFDKVGQKSKTDTSPLTLLKGARVLHHTAK 955
                    . : *** :**.* * . :* : : :* :* :.      . .  : *...:.*.* **

                                                     R   F
human             LPADLRLSISVIDSSDEATIRLANMLKIEQLKTTSCLKLVLKDIENAFYSHEEVTQLMLW 1015
orangutan         LPADLRLSISVIDSSDEATIRLANMLKIEQLKTTSCLKLVLKDIENAFYSHEEVTQLMLW 1015
mouse             LPTDLRLSVSVIDSSDEATIRLANMLKIEKLKTTSCLKFVLKDIGNAFYTQEEVTQLMLW 1017
rat               LPTELRLSVSVIDSSDEATIRLANMLKIEKLKTTSCLKFVLKDIENAFYTQEEVTRLMLW 1016
dog               LAPDLRLSISVIDSSDEATIRLANMLKIEKLKTTHCIKLILRDIENAFYSHDEVTHLMLW 1013
horse             LPPGLRLSVPVIDSSDEATIRLANMLKIETLKTTSCLQLVLKDMENAFYSHEEITHLMLW 1012
chicken           IPPGLRFSVPVIDSSDEATIRLANLLKIEQLKSTDCLKFIIQDIRSCFYSYDETTQIMQW 1007
zebra finch       IPPGLRFSVPIIDSSDEATIRLANLLKIEQLKSTDCLKFVIEDIRSDFYSYDETAQIMQW 1013
anole lizard      IPPDIKLSISLVDSSDEATIRLVKILKAEQLRSTDCIKFILNDIQSDFYCNEEATKIMLW 1006
fugu              YPPNVKLSINLVGYCDEESLRLIKMLNIEQITTTECLKFIVHDIERGFYTTDEITQIMLW 1015
tetraodon         YPPDVKLSINLVGYCDEESIRLIKMLNIEQLTTTECLKFIVHDIERGFYTTEEMAHIMLW 1012
stickleback       YPPDVKLSINLVGCCDEESIRLIKMLNVQQLKTTECLKMIIQDIERGFYTTDEVTQVMLW 1015
medaka            YPPDVKLSQSLVACSDEESIRLFKMLNVEQLKTTECLKMIIQDMETGFYTKDETTKIMLW 1015
zebrafish         HPLDVKLSINVVDCSDEATIRLVKLLNIEQVKSTECLKVIIQDIEKGFYTKEEVTKIMLW 1015
                   .  :::*  ::  .** ::** ::*: : : :* *::.::.*:   **  :* :::* *

                                                        S           X
human             VLENLSSLKNENPNVLEWLTPLKFIQISQEQMVSAGELFDPDIEVLKDLFCNEEGTYFPP 1075
orangutan         VLENLSSLKNENPNVLEWLTPLKFIQISQEQMVSAGELFDPDIEVLKDLFCNEEGTYFPP 1075
mouse             ILENLSSLKNENSNVLDWLMPLKFIHMSQGHVVAAGDLFDPDIEVLRDLFYNEEEACFPP 1077
rat               ILENLSSLKNENPSVLDWLMPLKFIHMSQEHVVAASDLFDPDIEVLRDLFYNEEEACFPP 1076
dog               ILENLSSLKNENPNVLDWLMPLKFIQISQEQMVSAGELFDPDIEVLKDLFYNEEETCFPP 1073
horse             ILENLSSLKNENPNVLDWLMPLKFIQISQEQIVSASELFDPDIEILKDLFYDEEETCFPP 1072
chicken           VLENLTFLKNENTDVIDWLAALRFIRISEEKLMTANELFDPEVELLQNLFYSEEEISFPP 1067
zebra finch       VLENLTFLKNENTDVIDWLTPLKFIQISPEKIVSANELFDPEVELLQHLFYAEEESCFPP 1073
anole lizard      ILENLTFLKNENAEVLDWLAPLKFIQISEGKLVSASELFDPEVEVLQNLFYGEEEHCFPP 1066
fugu              ALQNMAFLKNENSSVLGWLSPIKFIQLPCGKLAKASDLFDPELEILQNLFYMEEKTRFPT 1075
tetraodon         ALQHMAFLKNENPSVLSWLSPIKFIQLPCGRLVKASDLFDPELEILQNLFYMEEKSRFPT 1072
stickleback       ALKHLAFLKNENPTVIRWLSALTFIQLPCGKSVKASDLFDSELEILQNLFYMEEKSRFPP 1075
medaka            ALKHLAFLKNENSCVIGWLSTLKFIHMPCGKSVKASDLFDPELEILQHLFYMEEKTRFPT 1075
zebrafish         ALTYLSFLKNENKAVISWLSSLKFIHTASEKLHSPTDFFDPELEILQNLFFMEEKVRFPP 1075
                   *  :: *****  *: ** .: **: .  :   . ::**.::*:*:.**  **   **.

human             SVFTSP-DILHSLRQIGLKNEASLKEKDVVQVAKKIEALQVGACPDQDVLLKKAKTLLLV 1134
orangutan         SVFTSP-DILHSLRQIGLKNEASLKEKDFVQVAKKIEALQVGACPDQDVLLKKAKTLLLV 1134
mouse             TIFTSP-DILHSLRQIGLKNESSLKEKDVVQVARKIEALQVSSCQNQDVLMKKAKTLLLV 1136
rat               AVFTSP-DILHSLRQIGLKNEASLKEKDVVQVAKKIEALQVSSCQNQDVLLKKAKTLLLV 1135
dog               SVFTSP-DILHSLRQIGLKNEASLKENDVVQVAKKIEDLQVSSCPNQDILLKKAKTLLMV 1132
horse             SVFTSP-DILHSLRQIGLKNEASLKEKDVVQVAKKIEGLQVSSCPNQDVLLKKAKTLLLV 1131
chicken           AIFTSS-DILHSLRQIGLKSEANLEESDIMRVANKIESLHADSNTDCDLLVRKARTLLMI 1126
zebra finch       VIFTS--DILHSLRQIGLKNEANLEENDILRVAKKIESLHADSNTNHDLLLRKARTLLTI 1131
anole lizard      LTFRASSDILHSLRLIGLKSEGSLEEKDILHLAHKIENLRDCTGTNHDALKRKARTLLMI 1126
fugu              SEFTFSSDVLHSLRQLGLRNEVQLNEKDVVTVAKKIEELQHSKDTNEDLVIKKAKMLLQI 1135
tetraodon         SELASSPDVLHSLRQLGLRNEVQLSERDVVTVAKRIEELQRSQDAKEDLVIKKAKTLLQI 1132
stickleback       GLFKSSADILHSLRQLGLRNEVELTEKDVLKVAMKIEELQSSREPEMELIVKKAKTLLQI 1135
medaka            NVFTSSPDILHSLRQLGLKNEVQLNEKDALKVAKKIEELQSSEDPDWDSITKKAKTLLQI 1135
zebrafish         DDFMSSPDVLHSLRQLGLKNEVQLNEKDVLQVARKIEELQGNSKPEWDPILKKAKTLLTI 1135
                    :    *:***** :**:.* .* * * : :* :** *:     . : : :**: ** :

human             LNKNHTLLQSSEGKMTLKKIKWVPACKERPPNYPGSLVWKGDLCNLCAPPDMCDVGHAIL 1194
orangutan         LNKNHTLLQSSEGKMTLKKIKWVPACKERPPNYPGSLVWKGDVCNLCAPPDMCDVGHAIL 1194
mouse             LNKNQTLLQSSEGKMALKKIKWVPACKERPPNYPGSLVWKGDLCNLCAPPDMCDAAHAVL 1196
rat               LNKNHTLLQSSEGKMALKKIKWVPACKERPPNYPGSLVWKGDLCNLCAPPDMCDAAHAVL 1195
dog               LNKNHMLLQSSEGKMTLKKIKWVPACKERPPNYPGSLVWKGDVCNLCAPPDMCDGAHAIL 1192
horse             LNKNHALLQSAEGKMTLKKIKWVPACKERPPNYPGSLVWKGDICDLCAPPDMCDAMHAIL 1191
chicken           LNKNHKLLQSSETKTALKKIKWIPACKERPPNYPGSLVWKGDHCNLCLPPEMCDISHAIL 1186
zebra finch       LNKNHMLLQSSETKATMKKIKWIPASKERPPNYPGSLVWKGDHCNLCSPPEMCDISHAIL 1191
anole lizard      LNKSCSLLRSPETKASLKKIKWIPACKERPPNYPNSLTWKGDHCNLCSPPEMCDLSQATL 1186
fugu              LNRQTKLVKSADAQTALLKLQWVPVCKERPLTYPKSLSWVGDAATICSLSEMCDISHAVL 1195
tetraodon         LNRQTKLVKSADAQSALLKLQWVPVCKERPLTYPKSLSWVGDVATICSLSEMCDIAHAVL 1192
stickleback       LNRQTKLVKSADAQMSLLKLKWVPVCKERPLTYPKSLAWVGDDLNICSLSEMCDISHAVL 1195
medaka            LNKQTKLIKTADTQASLLKLKWVPVCKERPPTYPKSLAWVGDTLNISSLSEMCELSHAVL 1195
zebrafish         LNKQTKLVKSSEVQTNLQKLKWVPVCKDRPLNYPKSLAWRGDSCNISSLSEMCDISYAVL 1195
                  **:.  *:::.: :  : *::*:*..*:** .** ** * **   :.  .:**:   * *
```

```
                                                                    C     T
human             IGSSLPLVESIHVNLEKALGIFTKPSLSAVLKHFKIVVDWYSSKTFSDEDYYQFQHILLE 1254
orangutan         IGSSLPLVESIHVNLEKALGIFTKPSISAVLKHFKIVVDWYTSKTFSDEDYYQFQHILLE 1254
mouse             VGSSLPLVESVHVNLEQALSIFTKPTINAVLKHFKTVVDWYTSKTFSDEDYYQFQHILLE 1256
rat               VGSSLPLVESAHVSLEKTLGIFTKPTISAVLKHFKIVVDWYTSKTFSDEDYYQFQHILLE 1255
dog               VGSSLPLVESVHINLEKALGIFTKPSISAVLKHFKIVVDWYTSKTFSDEDYYQFQHILLE 1252
horse             VGSSLPLVESVHINLEKALGIVTKPSISAVLKHFKIVVDWYTSKTFSDEDYYQFQHILLE 1251
chicken           VGSSVPLVENVQFDIEKALGISTKPSIKAVLKHFKVVVDWHSSKTFSDEDYYQFQHILLE 1246
zebra finch       VGSSVPLVENVVLDIEKALGIPTKPSIKAVLKHFKVVVDWHGSKTFSDEDYYQFQHILLE 1251
anole lizard      VGSSVPLVEGVQPSVEKALGISIKPSIKSVLKHFKIVVDWHSSKKFSDEDYYQFQHILLE 1246
fugu              VGSAVALVEHTSAGMKKALKLSLEPQADQVVQHLRAVIDWHKSKAFTTEDWYQFQQILFE 1255
tetraodon         VGSAVALVEHTSAGMRKALKLNVEPQVDQVIQHLKAVIEWHKSQAFTTEDWYQFQQILFE 1252
stickleback       VGSSVALVEHTSAGMKKALKLTTEPQVDQVLQQLKAVNDWHKSQAFTTEDWYQFQQILFE 1255
medaka            VGSSVAVVEHTSAGLKKSLKLNIEPQVDQVLQHLKAVNDWHKSQAFTTEDWYQFQQILFE 1255
zebrafish         VGSSVSLVERTSAGMKKALKLSLEPQVDQVLQHLKAVNDWHQSQAFTTEDWYQFQQILCE 1255
                  :**::.:**    .:.::* :  :*  . *::::: * :*: *: *: **:****:** *

                                                                          K
human             IYGFMHDHLNEGKDSFRALKFPWVWTGKKFCPLAQAVIKPIHDLDLQPYLHNVPKTMAKF 1314
orangutan         IYGFMHDHLNEGKDSFKALKFPWVWTGKKFCPLAQAVIKPIHDLDLQPYLHNVPKTMAKF 1314
mouse             IYGFMHDHLSEGKDSFKALKFPWVWTGKNFCPLAQAVIKPTHDLDLQPYLYNVPKTMAKF 1316
rat               IYGFMHDHLSEGKDSFKALKYPWVWTGKNFCPLAQAVIKPIHDLDLQPYLYNVPKTMAKF 1315
dog               IYGFMHDHLSEGKDAFRALKFPWVWTGKKFCPLAQAVIKPFHDLDLQPYLHSVPKTMAKF 1312
horse             IYGFMHDHLNEGKDAFRALKFPWVWTGKKFCPLSQAVIKPFHDLDLQPYLHSVPKTMAKF 1311
chicken           IYGFMHDHLEEGKEAFKALKFPWVWTGKTFCSLTQAVIKTVPDLDLQPYLHYVPKTMAKF 1306
zebra finch       IYGFMHDHLEEGKEAFKSLKFPWVWTGKTFCSLAQAVIKSVPDLDLQPYLHYVPKTMAKF 1311
anole lizard      IYGFMHDHLDEGKEAFKTLKFPWVWTGKTFCSLSQAVIKSVHDLDLQPYLHNVPKTMAKF 1306
fugu              IYGFMQTHIQDAKEAMKSLPFDWVWTGKTFSSPGRTVLKPLPDLDLQPYLYSLPKTIRKF 1315
tetraodon         MYGFMQTHLQDAKEAMKSLPFDWVWTGKTFSSPDRTVLKPLPDLDLQPYLYSLPKTIRKF 1312
stickleback       IYGFMQAHLEDAREAMKSLPFDWVWTGKTFSSPGHTALKPLPDLDLQPYLYTLPKTIRKF 1315
medaka            IYSFMQAHLEDAREAMKSLSFDWVWTGKTFSSPSQTVLKPLADLDLQPYLYSLPKTMRKF 1315
zebrafish         IYEFMQAHLEDAREALKSLSFDWVWTGKTFASPGCTVLKPIPDLDLQPYLYSLPKTIAKF 1315
                  :* **: *:.:.::::::* : ******.*..   :.:*.  ********: :***: **

                                                                            V
                                 E               X                        X  P
human             HQLFKVCGSIEELTSDHISMVIQKIYLKSDQDLSEQESKQNLHLMLNIIRWLYSNQIPAS 1374
orangutan         HQLFKVCGSIEELTSDHISMVIQKIYLKSDQDLSEQESKQNLHLMLNIIRWLYSNQIPAS 1374
mouse             HQLFKACGSIEELTSDHISMVIQKVYLKSDQELSEEESKQNLHLMLNIMRWLYSNQIPAS 1376
rat               HQLFKACGSIEELTSDHISMVIQKVYLKSDQELSEEESKQNLHLMLNIMRWLYSNQIPAS 1375
dog               HQLFKVCGSIEELTSDHISMVIQKVYLKSDQDLSDQESKQNLHLMLNIIRWLYSNQIPAS 1372
horse             HQLFKVCGSIEELTSDHISMVIQKVYLKSDQDLSEQESKQNLHLMLNIIRWLYSNQIPAS 1371
chicken           HQLFKCCGSIEQLTPDHVSMVIQKIYLKSEQSLSEQESKQNLHIMLSIIRWLYSNQIPAS 1366
zebra finch       HQLFKCCGSIEELTPDHVSMVIQKIYLKSEQSLSEQESKQSLHIMLSIIRWLYSNQIPAS 1371
anole lizard      HQLFKCCGSIEQLTPDHVSMVIQKIYLRSEQPLTEDESKQNLHIMLNIIRWLYRCQIPAN 1366
fugu              NKLFKSCGSIEEVVPSQVLEVINTIQQRCTGELTKDDSKHNILLLLNILRWLHNNQIPVD 1375
tetraodon         NKLFKSCGSIEEVDPSQVLEVVNTIQQRCTGEMTKDDSKHNILLLINILRWLYNNQIPVA 1372
stickleback       HKLFKFCGSVEEVVSSHVFEVISTIKQRCDGEITKEESKHDILLLVNILRWLNNNQIEVD 1375
medaka            QKLFKFCGSVEEVVPNHVFDVIATIRQRCEEEMTKEESKHDILLLINILRWLYNTQTPVD 1375
zebrafish         NKLFKFCGSIEEVVPSHVFEVVKTIRQRCEEEMTKQESKRNVLLLINILRWLYSSQIPVD 1375
                  ::*** ***:*:: ..::  *: .:  :.   ::.::**:.: :::.*:***   *  .

                                         R N
human             PNTPVPIHHSKNPSKLIMKPIHECCYCDIKVDDLNDLLEDSVEPIILVHEDIPMKTAEWL 1434
orangutan         PNTPVPIHHSKNPSKLIMKPIHECCYCDIKVDDLNDLLEDSVEPIILVHEDIPMKTAEWL 1434
mouse             PNTPVPIYHSRNPSKLVMKPIHECCYCDIKVDDLNDLLEDSVEPIILVHEDIPMKTAEWL 1436
rat               PNTPVPIYHSKNPSKLVMKPIHECCYCDIKVDDLNDLLEDSVEPIILVHEDIPMKTAEWL 1435
dog               PNTPVPIHHSKNPSKLIMKPIHECCYCDIKVDDLNDLLEDSVEPIILVHEDIPMKTAEWL 1432
horse             PNTPVPIHHSKNTSKLIMKPIHECCYCDIKVDDLNDLLEDSVEPIILVHEDIPMKTAEWL 1431
chicken           LHTPMPLYSGKLPYKLVMRPIHECCYCDIKVDDLNDLLEDSVEPIILVHEDIPMKTAEWL 1426
zebra finch       LHTPMPLYSGKLPYKLAMKPIHECCYCDIKVDDLNDLLEDSVEPIILVHEDIPMKTAEWL 1431
anole lizard      LNTPVPIHCSKKPYRFTMKPIHDCCYCDIKVDDLNDLLEDSVEPIILVHEDIPMKTAEWL 1426
fugu              TDMHVPILFHKDPSKLAMKPIHECTYCDIKVDDLIDLLEDTAEPIILVHDDIPMKTAEWL 1435
tetraodon         TDMHVPILFHKDPSRLAMRPIHECTYCDIKVDDLIDLLEDTSEPIVLVHDDIPMRTAEWL 1432
stickleback       TDMHVPILCYKDPSKLALKPIHECTYCDIKVDDLNDLLEDASEPIILVHDDIPMKTAEWL 1435
medaka            TSMHVPILCYKDPSKLAMKPIHECTYCDIKVDDLNDLLEDGSEPIILVHDEIPMKTAEWL 1435
zebrafish         ADMHVPILYHKDPSKLVMKPIHECTYCDIKVEDLNDLLDDASEPIILVHDDIPMKTAEWL 1435
                      :*:   : . :: ::***:* ******:** ***:*  ***:***::***:*****

                                                                        S
human             KVPCLSTRLINPENMGFEQSGQREPLTVRIKNILEEYPSVSDIFKELLQNADDANATECS 1494
orangutan         KVPCLSTRLINPENMGFEQSGQREPLTVRIKNILEEYPSVSDIFKELLQNADDANATECS 1494
mouse             KVPCLSTRLINPENMGFEQSGQREPLTVRIKNILEEYPSVSDIFKELLQNADDANATECS 1496
rat               KVPCLSTRLINPENMGFEQSGQREPLTVRIKNILEEYPSVSDIFKELLQNADDANATECS 1495
dog               KVPCLSTRLINPENMGFEQSGQREPLTVRIKNILEEYPSVSDIFKELLQNADDANATECS 1492
horse             KVPCLSTRLINPENMGFEQSGQREPLTVRIKNILEEYPSVSDIFKELLQNADDANATECS 1491
chicken           NVPCLSTRLINPENMGFEQSGQREPLTVRIKNILEEYPSISDIFKELLQNADDANASECN 1486
zebra finch       NVPCLSTRLINPENMGFEQSGQREPLTVRIKNILEEYPSISDIFKELLQNADDANATECN 1491
anole lizard      NVPCLSTRLINPENMGFEQSGQREPLTVRIKNILEEYPSVSDIFKELLQNADDANATDCN 1486
fugu              KVPCLSTRLVNPENLGFEQSGQREPLTVRIKNILEEYPSVADIFKELLQNADDASATECN 1495
tetraodon         KVPCLSTRLVNPENLGFEQSGQREPLTVRIKNILEEYPSVADIFKELLQNADDASATECN 1492
stickleback       KVPCLSTRLINPENLGFEQSGQREPLTVRIKNILEEYPSVADIFKELLQNADDASATECS 1495
medaka            KVPCLSTRLINPENLGFEQSGQQEPLTVRIKNILEEYPSVADIFKELLQNADDASATECS 1495
zebrafish         KVPCLSTRLINPENLGFEQSGQREPLTVRIKNILEEYPSVADIFKELLQNADDANATECS 1495
                  :********:****:*******:****************::*************.*::*.
```

```
                                              R
human             FLIDMRRNMDIRENLLDPGMAACHGPALWSFNNSQFSDSDFVNITRLGESLKRGEVDKVG 1554
orangutan         FLIDMRRNMDIRENLLDPGMAACHGPALWSFNNSQFSDSDFVNITRLGESLKRGEVDKVG 1554
mouse             FMIDMRRNMDIRENLLDPGMAACHGPALWSFNNSEFSDSDFLNITRLGESLKRGEVDKVG 1556
rat               FMIDMRRNMDIRENLLDPGMAACHGPALWSFNNSEFSDSDFINITRLGESLKRGEVDKVG 1555
dog               FMIDMRRNMDIRENLLDPGMAACHGPALWSFNNSQFSDSDFVNITRLGESLKREEVDKVG 1552
horse             FMIDMRRNMDIRENLLDPGMAACHGPALWSFNNSEFSDSDFVNITRLGESLKRAEVDKVG 1551
chicken           FLIDMRRNMDIRENLLDPGMAACHGPALWSFNNSEFSDSDFLNITRLGESLKRSEVDKVG 1546
zebra finch       FLIDMRRNMDIRENLLDPGMAACHGPALWSFNNSEFSDSDFLNITRLGESLKRSEVDKVG 1551
anole lizard      FLIDMRRNNDIRENLLDPGMAASHGPALWSFNNSEFSDNDFLNITRLGESLKRAEVDKVG 1546
fugu              FLIDMRKNNEIRENLLDPGMIVCHGPSLWSFNNAVFSDTDFLNITRLGGSVKRSEADKVG 1555
tetraodon         FLIDMRKNNDIRENLLDPGMIACHGPSLWSFNNAVFSDTDFLNITRLGGSVKRSEADKVG 1552
stickleback       FLIDMRKNTNIRENLLDPGMIVCHGPSLWSFNSSVFSDADFLNITRLGGSVKRCEADKVG 1555
medaka            FLIDMRKNLDIRENLLDPGMIVCHGPSLWSFNNAVFTDTDFLNITRLGGSIKRCEADKVG 1555
zebrafish         FLIDMRKNLEIRENLLDPGMIICHGPALWSFNNSVFSDTDFLNITRLGGSMKRCEADKVG 1555
                  *:****:* :**********  .***:*****.: *:* **:****** *:** *.****

                                      P       R   R
human             KFGLGFNSVYHITDIPIIMSREFMIMFDPNINHISKHIKDKSNPGIKINWSKQQKRLRKF 1614
orangutan         KFGLGFNSVYHITDIPIIMSREFMIMFDPNINHISKHIKDKSNPGIKINWSKQQKRLRKF 1614
mouse             KFGLGFNSVYHITDIPIIMSREFMIMFDPNINHISKHIKDRSNPGIKINWSKQQKRLRKF 1616
rat               KFGLGFNSVYHITDIPIIMSREFMIMFDPNINHISKHIKDRSNPGIKINWSKQQKRLRKF 1615
dog               KFGLGFNSVYHITDIPIIMSREFMIMFDPNINHISKHIKDKSNPGIKINWSKQQKRLRKF 1612
horse             KFGLGFNSVYHITDIPIIMSREFMIMFDPNINHISKHIKDKSNPGIKINWSKQQKRLRKF 1611
chicken           KFGLGFNSVYHITDVPIILSREFMIMFDPNVNHIGKHIRDKSNPGIKINWSKQQKRLRKF 1606
zebra finch       KFGLGFNSVYHITDVPIILSREFMIMFDPNVNHISKHIRDKSNPGIKINWSKQQKRLRKF 1611
anole lizard      KFGLGFNSVYHITDIPIIMSREFMIMFDPNINHLSKHIRDKSNPGIKINWSKQQKRLRKF 1606
fugu              KFGLGFNSVYHITDIPIIMSREFMIIFDPNINHIRKHIRDRSNPGIKINWSKQQKRLRKF 1615
tetraodon         KFGLGFNSVYHITDIPIIMSREFMIIFDPNINHISKHIRDRSNPGIKINWSKQQKRLRKF 1612
stickleback       KFGLGFNSVYHITDIPIIMSREFMIMFDPNINHISKHIRDSSNPGIKINWSKQQKRLRKF 1615
medaka            KFGLGFNSVYHITDIPIIMSREFMIMFDPNINHISKHIRDRSNPGIKINWSKQQKRLRKF 1615
zebrafish         KFGLGFNSVYHVTDIPIIMSREFMIMFDPNINHIGKHIRDKSNPGIKINWSKQQKRLRKF 1615
                  ***********:**:***:******:****:**: ***:* *******************

                                                Q       X
human             PNQFKPFIDVFGCQLPLTVEAPYSYNGTLFRLSFRTQQEAKVSEVSSTCYNTADIYSLVD 1674
orangutan         PNQFKPFIDVFGCQLPLTVEAPYSYNGTLFRLSFRTQQEAKVSEVSSTCYNTADIYSLVD 1674
mouse             PNQFKPFIDVFGCQLPLAVEAPYSYNGTLFRLSFRTQQEAKVSEVSSTCYNTADIYSLVD 1676
rat               PNQFKPFIDVFGCQLPLAVEAPYSYNGTLFRLSFRTQQEAKVSEVSSTCYNTADIYSLVD 1675
dog               PNQFKPFIDVFGCQLPLTVEAPYSYSGTLFRLSFRTQQEAKVSEVSSTCYNTADIYSLVD 1672
horse             PNQFKPFINVFGCQLPLTVEAPYSYNGTIFRLSFRTQQEAKVSEVSSTCYNTADIYSLVD 1671
chicken           PNQFKPFIGVFGCQLPLTVESPYSYKGTLFRLSFRTQQEAKVSEVSNTCYNTADIYSLVD 1666
zebra finch       PNQFKPFIGVFGCQLPLTVESPYSYKGTLFRLSFRTQQEAKVSEVSSASYNTADIYSLVD 1671
anole lizard      PNQFKPFIGVFGCQLPLTMEAPYSYKGTLFRLSFRTQQEAKVSEVSSTSYNTADIYSLVD 1666
fugu              PNQFKPFINVFGCELPLAQDSPYKYNGTLFRLPFRTEQEASQSEISSLYYNTTDIYSLVD 1675
tetraodon         PNQFKPFINVFGCQLPLAQDSPYKFNGTLFRLPFRTEQEASQSEISSLYYNTTDIYSLVD 1672
stickleback       PNQFKPFINVFNCQLPLAEDSPYKYNGTLFRLPFRTEQEASASEISSLYYNTTDIYSLVD 1675
medaka            PNQFKPFINVFNCQLPLAQDSPYKFNGTLFRLPFRTEQEAAVSEISSLYYNTTDIYSLVD 1675
zebrafish         PNQFRPFINVFNCQLPLSLEAPYKYDGTLFRLPFRTEQEASASEISGIYYNTTDIYSLVD 1675
                  ****:***.**.*:***: ::**.:.**:***.***:***  **:*.  ***:*******

                                                    X     X
human             EFSLCGHRLIIFTQSVKSMYLKYLKIEETNPSLAQDTVIIKKKSCSSKALNTPVLSVLKE 1734
orangutan         EFSLCGHRLIIFTQSVKSMYLKYLKIEETNPSLAQDTVIIKKKSCSSKALNTPVLSVLKE 1734
mouse             EFSLCGHRLIIFTQSVNSMYLKYLKIEETNPSLAQDTIIIKKKVCPSKALNAPVLSVLKE 1736
rat               EFSLCGHRLIIFTQSVNSMYLKYLKIEETNPTLAQDTIIIKKKACPSKALNAPVLSVLKE 1735
dog               EFSLCGHRLIIFTQSVTSMYLKYLKIEETNPSLAQDTVIIKKKPCPSKALSAPVLSVLKE 1732
horse             EFSLCGHRLIIFTQSVTSMYLKYLKIEEANPSLAQDTVIIKKKSCSSKALHAPVLSVLQE 1731
chicken           EFSICGHRLIIFAQSVNSMVLKYLKIEAEDPGAAQDVVTIKKRVCSSKALTGPNVSVLKE 1726
zebra finch       EFSICGHRLIIFAQSVNSMVLKYLKIEADDPGVAQDVVTIRKNLCSSKALTAPNVSVLKE 1731
anole lizard      EFSICGHRLIIFTQCVNSMVLSYLKTEETNPDAAQETVTIKKNLCVSKAITTPVTSILKE 1726
fugu              EFSICGHRFILFTQHVGCMLLKYLKYEEPDPVAAQDVVSINKSVWSSKASYGP-LSILKA 1734
tetraodon         EFSICGHRFILFTQHVGGMVLKYLKYEDPDPAAAQDVVTINKSVWSSKASYGP-LSILKA 1731
stickleback       EFSICGHRFILFTQHVGSMVLKYLKYEEPNPAGAQDVVTINKSVWSSKSSYGP-LSILKS 1734
medaka            EFSICGHRFILFTQHVGCMILKYLKYEEPNPAGAQDVVTINKSVWSSKASYGP-LSILKS 1734
zebrafish         EFSICGHRLILFTQHVGTMVLKYLKCEEPNPAAAQDVVTINKSVWSSKAAYGP-LSILKS 1734
                  ***:****:*:*:* *  * *.*** *  :*  **:.: *.*    **:   *  *:*:

human             AAKLMKTCSSSNKKLPSDEPKSSCILQITVEEFHHVFRRIADLQSPLFRGPDDDPAALFE 1794
orangutan         AAKLMKTCSSSNKKLPSDEPKSSCILQITVEEFHHVFRRIADLQSPLFRGPDDDPAALFE 1794
mouse             AAKLMKTCSSSNKKLPTDVPKSSCILQITVEEFHHVFRRIADLQSPLFRGPDDDPATLFE 1796
rat               AAKLMKTCSSSNKKLPTDVPKSSCILQITVEEFHHVFRRIADLQSPLFRGPDDDPATLFE 1795
dog               AARLMKTCSSSNKKLPADAPKSSCILQITVEEFHHVFRRIADLQSPLFRGPEDDLTALFE 1792
horse             AAKLMKTCSSSNKKPPADAPKSSCILQITVEEFHHVFRRIADLQSPLFRGPDDDPAALFE 1791
chicken           AAKLMKVCSSSNKKLPTETPKSSCILQIVVEEFHHVFRRIADLQSPLFRGAEDDPAAFFE 1786
zebra finch       AAKLMKVCSSSTKKLPTEAPKSSCILQIVVEEFHHVFRRIADLQSPLFRGTEDDPTAFFE 1791
anole lizard      AAKVMKTCSSSTKKLPAESPKSSCILQITIEEFHHVFRRIADLHSPVFRGPEDDTVSFFE 1786
fugu              AAKVMKKVASTN-KVPTDIPKSGCIIRILVEEFHNVFKRIVDLHSPLFRGSEEDPNQYFE 1793
tetraodon         AAKVMKKVASTN-KVPADIPKSGCIIRILVEEFHNVFKRIVDLHSPLFRGSEEDPNQYFE 1790
stickleback       AAKVMKKVASTN-RVPADVPKSGCIIRVLVEEFHNVFKRIVDLHSPLFRGSEEDPNQYFE 1793
medaka            AAKLMKKVASTN-KIPADFPKSGCIIRVVVEEFHNVFKRIVDLHSPLFRGSEEDPNQYFE 1793
zebrafish         AAKVMKKVAATS-KVPTEVPKSGCIIRIIVEEYHNVFRRIVDLQSPLFRGSDEDPSSYFE 1793
                  **::**  :::. : *:: ***.**::: :**:*:**:**.**:**:***.::*    **
```

```
                                         R
human              MAKSG-QSKKPSDELSQKTVECTTWLLCTCMDTGEALKFSLSESGRRLGLVPCGAVGVQL 1853
orangutan          MAKSG-QSKKPSDELSQKTVECTTWLLCTCMDTGEALKFSLSESGRRLGLVPCGAVGVQL 1853
mouse              MAKSG-QSKKPSDELPQKTVDCTTWLICTCMDTGEALKFSLNESGRRLGLVPCGAVGVLL 1855
rat                MAKSG-QSKKPSDELPQKTVDCTTWLICTCMDTGEALKFSLNESGRRLGLVPCGAVGVLL 1854
dog                MAKSG-QSKKPSDELPQKTVECTTWLICTCMDTGEALKFSLNENGRRLGLVPCGAVGVLL 1851
horse              MAKLG-QSKKPSDELPQKTVECTTWLLCSCMDTGEALKFSLSESGRRLGLVPCGAVGVLL 1850
chicken            MAKSG-QVKRSADELPQKTVDSTTWLICSCMDTGDALKFSLHESGRRLGLVPCGGVAVLL 1845
zebra finch        MAKSG-QTKRSGDELPQKTVDSTTWLICSCMDVGDALKFSLHESGRRLGLVPCGGVGVLL 1850
anole lizard       MAKSG-QTKRP-DELPQKTVDTTTWLICSCMDTGEALKFSLQESGRRLGLVPCGSVGVIL 1844
fugu               MAAKGIQTRRLTDEIPQKAVEVTNWLICSCMDANEALKFSLSDSGKRLGLVPCGGVAVLL 1853
tetraodon          MAAKGVQTRRLTDEIPQKALEVTNWLICSCMDANEALKFSLSDSGKRLGLVPCGGVAVLL 1850
stickleback        MAAKGIQTRRLTDEMSAKAVEVTNWLICSCMDVTEALKFSLSDSGKRLGLVPCGGVAVLL 1853
medaka             MAAKGVQTRRLTDEMPQRAVEVTSWLICSCMDGSEALKFSLGDSGKRLGLVPCGGVAVLL 1853
zebrafish          MAAKGTQTKRLTDDMPQKGVENTNWLICSCMDVTEALKFSLNDSGKRLGLVPCGGIAVLL 1853
                   **  * * ::  *::. : :: *.**:*:***  :****** :.*:********.:.* *

                                              X  I                          X
human              SEIQDQKWTVKP---HIGEVFCYLPLRIKTGLPVHINGCFAVTSNRKEIWKTDTKGRWNT 1910
orangutan          SEIQDQKWTVKP---HIGEVFCYLPLRIKTGLPVHINGCFAVTSNRKEIWKTDTKGRWNT 1910
mouse              HETQEQKWTVKP---HIGEVFCYLPLRIKTGLPIHINGCFAVTSNRKEIWKTDTKGRWNT 1912
rat                HETQEQKWTVKP---HIGEVFCYLPLRIKTGLPIHINGCFAVTSNRKEIWKTDTKGRWNT 1911
dog                SEIQDQKWAVKP---HVGEVFCYLPLRIKTGLPVHINGCFAVTSNRKEIWKTDTKGRWNT 1908
horse              SEIQDQKWAVKP---HVGEVFCYLPLRIKTGLPVHINGCFAVTSNRKEIWKTDTKGRWNT 1907
chicken            TETQDQKWIVKPNCNNIGEVFCYLPLRIKTGLPLHINGCFAVTSNRKEIWKTDTKGRWNT 1905
zebra finch        SETQDQKWIVKPNCNNIGEVFCYLPLRIKTGLPLHINGCFAVTSNRKEIWKTDTKGRWNT 1910
anole lizard       SEKQDGKWTVKPNCSNLGEVFCYLPLRIKTGLPVHINGSFAVTSNRKEIWKTDTKGRWNT 1904
fugu               SEEENHKWTVKMNATPTGEVFCYLPLRIKTGLPVHINGCFAVTSNRKEIWKTDTKGQWNS 1913
tetraodon          SEEENHKWTVKTNATPSGEVFCYLPLRIKTGLPVHINGCFAVTSNRKEIWKTDTKGQWNS 1910
stickleback        SEEENRKWTVKTNATPIGEVFCYLPLRIKTGLPVHINGCFAVTSNRKEIWKTDTKGQWNS 1913
medaka             SEEENRKWTVKTNPNPIGEVFCYLPLRIKTGLPVHINGCFAVTSNRKEIWKTDTKGQWNS 1913
zebrafish          TEEDNRKWTVKTSATPIGEVFCYLPLRIKTGLPVHINGCFAVTSNRKEIWKTDTKGQWNS 1913
                    * :: ** **      ****************:****.*****************:**:

                                                      R
human              TFMRHVIVKAYLQVLSVLRDLATSGELMDYTYYAVWPDPDLVHDDFSVICQGFYEDIAHG 1970
orangutan          TFMRHVIVKAYLQALSVLRDLATSGELMDYTYYAVWPDPDLVHDDFSVICQGFYEDIAHG 1970
mouse              TFMRHVIVKAYLQALSVLRDLAIGGELTDYTYYAVWPDPDLVHDDFSVICKGFYEDIAHG 1972
rat                TFMRHVIVKAYLQALSVLRDLAISGELIDYTYYAVWPDPDVVHDDFSVICQGFYEDIAHG 1971
dog                TFMRHVIVKAYLEALSVLRDLATNGELMDYTYYSVWPDPELVHDDFSVICQGFYEDIAHG 1968
horse              TFMRHVVVKAYLEALSVLRDLATSGELVDYTYYAVWPDPDSVHDDFSVICQGFYEDIAHG 1967
chicken            VFMRHVIVKAYIEALCVLRDMAISGELVDYSYCAVWPDPDSVHDDFSVICQGFYEDIAHM 1965
zebra finch        VFMRHVIVKAYIEALCVLRDMAINGELVDYSYCAVWPDPDSVHDDFSVICQGFYEDIAHM 1970
anole lizard       VFMRHVIVKAYLEALVVLRDMAVCGELEDYCYCAVWPDPDLVHDDFSVICQGFYEEIAHM 1964
fugu               VFMRHVIVQAYLAALAMLRSMAENGELLDYSYYAVWPDPSQVHDDFALISQGVYQEIVKG 1973
tetraodon          VFMRHVIVQAYLAALAMLRSMAENGELLDYTYYAAWPDPSQVHDDFALISQGVYQEIAKG 1970
stickleback        VFMRHVIVQGYLAALTMLRSMAESGELLDYSYYAAWPDPSQVHDDFTLVSQGVYQEIAKG 1973
medaka             VFMRHVIVQAYLAALSMLRSMAESGELLEYNYYGAWPDPGQVHDDFTLVSQGVYQEISKG 1973
zebrafish          IFMRHVIVQAYLAALNMLRSMAESGELLDYSYYAAWPDPGVVHDDFNLICQGVYQEIAKG 1973
                    *****:*:.*: .* :**.:*  *** :* * ..****  ***** ::.:*.*::* :

                                                                 N
human              KGKELTKVFSDGSTWVSMKNVRFLDDSILKRRDVGSAAFKIFLKYLKKTGSKNLCAVELP 2030
orangutan          KGKELTKVFSDGSTWVSMKNVRFLDDSILKRRDVGSAAFKIFLKYLKKTGSKNLCAVELP 2030
mouse              KGKELTRVFSDGSMWVSMKNVRFLDDSILQRKDVGSAAFKIFLKYLKKTGSKNLCAVELP 2032
rat                KGKDLTRVFSDGSMWVSMKNVRFLDDSILQRKDVGSAAFKIFLKYLKKTGSKNLCAVELP 2031
dog                KGKELTRVFSDGSMWVSMKNVRFLDDSILKRKDVGPAAFKIFLKYLKKTGSKNLCAVELP 2028
horse              KGKELTRVFSDGSTWVSMKNVRFLDDSILKRRDVGPAAFKIFLKYLKKTGSKNLCAVELP 2027
chicken            KSKEGIKVFSDGISWVSMKNVRFLDDSILKRPDVGPSAFKIFLKYLKKTGSKNLCAVDLP 2025
zebra finch        KSKEGIKVFSDGSSWVSMKNVRFLDDSILKRPDVGPSAFKIFLKYLKKTGSKNLCAVDLP 2030
anole lizard       KGKEGIRVFSDGLSWVSMKNVRFLDDSLLKRPDIGTSAFQIFLKYLKKTGSKNLCAVDLP 2024
fugu               GDSEHAKVFSDGKTWVSIKYVRFLDDTLHSRIDIGPAAFKIFLKYLKKSGSQNLCAVELP 2033
tetraodon          GDSEHARVFSDGKTWVSIRYVRFLDDALHCRPDIGPAAFNIFLKYLKKSGSQNLYAVELP 2030
stickleback        GDGEHANVFSDGKTWVSIKYVRFLDDALLCRPDIGPAAFKIFLKYLKKSGSQNLCAVELP 2033
medaka             GDSEQSRFFSDGKTWVSIKYVRFLDDSLLCRPDIGPAAFKIFLKYLKKTGSQNLCAVELP 2033
zebrafish          ADDDRAKVFSDGKTWVSIKNVRFLDDSLLCRPDIGSAAFKIFLKYLRKCGSQDLCAVELP 2033
                    . :  ..****  ***:: ******::  * *:*.:**:******:* **::* **:**

human              SSVKLGFEEAGCKQILLENTFSEKQFFSEVFFPNIQEIEAELRDPLMIFVLNEKVDEFSG 2090
orangutan          SSVKLGFEEAGCKQILLENTFSEKQFFSEVFFPNIQEIEAELRDPLMIFVLNEKVDEFSG 2090
mouse              SSVKAGFEEAGCKQILLENTFSEKQFFSEVFFPNIQEIEAELRDPLMNFVLNEKLDEFSG 2092
rat                SSVKVGFEEAGCKQILLENTFSEKQFFSEVFFPNIQEIEAELRDPLMNFVLNEKLDEFSG 2091
dog                SSVKSGFEEAGCKQILLENTFSEKQFFYEVFFPNIQEIEAELRDPLMNFVLNEKIDEFSG 2088
horse              SSVKLGFEEAGCKQILLENTFSEKQFFSEVFFPNIQEIEAELRDPLMNFVLNEKVDEYSG 2087
chicken            SWVKTGFEEAGCKHILLENTFSEKQFFSEVFFPNVQEIAAELRDPLMRYVLNEKLEEFSG 2085
zebra finch        SWVKTGFEEAGCKYILLENTFSEKQFFSEVFFPNIQEIDAELRDPLMRYVLNEKVEEFSG 2090
anole lizard       SWVKEGFEEAGCKQVLIENTFSERQFFFEVFFPNIQEIEAELRDPLMHYVLSEKVDEFSE 2084
fugu               DWVKEGFEDAGCKGKLLENTLTEKQFFAQVFFPHIQEIDKQHRDPLIHYVLNEKLEEFSS 2093
tetraodon          DWVKEGFEDAGCKAKLLENTLTEKQFFAEVFFPHIQEIDKEHRDPLIHYVLNEKLEEFSS 2090
stickleback        DWVKEGFDDAGCKGKLMENTLTEKQFFSDVFFPHIEEIDRELRDPLVHYVLNEKLEDFAS 2093
medaka             DWVKEGFEDAGCKGKLLEKTLSERKFFSQVFFPHIQEIDTDLRDPLMHYVLNEKLEDFAS 2093
zebrafish          EWVKEGFDDAGCKGKLMENTLTEKQFFSDVFFPHIQDIDKDLRDPLMYYVLNEKLEDFAS 2093
                   . ** **::****  *:*:*::*::** :****::::*  : ****: :**.**:::::
```

```
                                       S  P   X                 X
human             VLRVTPCIPCSLEGHPLVLPSRLIHPEGRVAKLFDIKDGRFPYGSTQDYLNPIILIKLVQ 2150
orangutan         ILRVTPCIPCSLEGHPLVLPSRLIHPEGRVAKLFDIKDGRFPYGSTQDYLNPIILIKLVQ 2150
mouse             ILRVTPCVPCSLEGHPLVLPSRLIHPEGRVAKLFDTKDGRFPYGSTQDYLNPIILIKLVQ 2152
rat               ILRVTPCVPCSLEGHPLVLPSRLIHPEGRVAKLFDIKDGRFPYGSTQDYLNPIILIKLVQ 2151
dog               ILRVTPCIPCSLEGHPLVLPSRLIHPEGRVAKLFDIKDGRFPYGSTQDYLNPIILIKLVQ 2148
horse             ILRVTPCIPCSLEGHPLVLPSRLIHPEGRVAKLFDTKDGRFPYGSTQDYLNPIILIKLVQ 2147
chicken           IHRITPCIPCSLDGHPLVTPSRLIHPEGRVAKLYDAEDGRFPYGTTQDYLNPVILVKLVQ 2145
zebra finch       ILRATPCIPCSLDGHSLVTPSRLIHPEGRVAKLYDAEDGRFPYGTTQDYLNPVILVKLVQ 2150
anole lizard      ILCITPCIPCSLTDHPLVEPSRLIHPEGRVAKLYDKEDGRFPHGTSHDYLNPVILVKLVQ 2144
fugu              ILRVTPCIPCCGPNSTLVLPSRLIHPEGRVAKLYNADDGRFPEGTSKDYLNPVCLVKLVQ 2153
tetraodon         ILRVTPCVPCCGPRNTLVLPSRLIHPEGRVAKLYNTDDGRFPKGTSKDYLNPVCLVKLVQ 2150
stickleback       ILRVTPCIPCCGPKKELVLPSRLIHPEGRVAKLYHTDDGRFPEGTTKDYLNPVCLVKLVQ 2153
medaka            ILRVTPCIPCSGPTKELVLPCKLIHPEGRVAKLYNPDDGRFPDESSKDYVNPVCLVKLVQ 2153
zebrafish         ILKETPCIPSSGPSKMLVLPSRLIHPEGRVAKLYNSEDGRFPEGSLRDYVNPVCLVKLLQ 2153
                  :   ***:*..     ** *.:***********:. .*****  : :**:**: *:**:*

human             LGMAKDDILWDDMLERAVSVAEINKSDHVAACLRSSILLSLIDEKLKIRDPRAKDFAAKY 2210
orangutan         LGMAKDDILWDDMLERAVSVTEINKSDHVAACLRSSILLSLIDEKLKIRDPRAKDFATKY 2210
mouse             LGMAKDDILWDDMLERAESVAEINKSDHAAACLRSSILLSLIDEKLKIKDPRAKDFAAKY 2212
rat               LGMAKDDILWDDMLERAESVAEINKSDHAAACLRSSILLSLIDEKLKIRDPRAKDFAAKY 2211
dog               LGMAKDDILWDDMLERAQSVAEINKSDHAAACLRSSILLSLIDEKLKIRDPRAKDFAARY 2208
horse             LGMAKDDILWDDMLERAESVAEVNKTDHAAACLRSSILLSLIDEKLKIRDPRARDFAAKY 2207
chicken           LGMAKDDILWEDLIERAESVAEINKVDHAAACLRSSIILSLIDEKLKCRDPRAKEFAEKC 2205
zebra finch       LGMAKDDILWEDLIERAESVAEINKTDHAAACLRSSIILSLIDEKLKSRDPRAKEFAAKC 2210
anole lizard      LGMAKDDILWEDLVERAESVEEINTTDHAAACLRSSILLNLIDEKLKCRDPKAKEFSAKF 2204
fugu              LGMVKDDLSWEDLLERAHSVTELNENDHSAACFRSSLLLSLIDEKLKSRDSEADESLEKL 2213
tetraodon         LGMVKDDLSWEDLIERAESVVELNENDHMAACFRSSLLLSLTDEKLKARDPGAEESLERL 2210
stickleback       LGMVKDDLSWEDLIERAQSVIDLNEKDHTAACFRSSIVLSLIDEKLKMSDPGPAELLEKL 2213
medaka            LGMVKDDLSWEDLIERAHSVIELNESDHAAACFRSSVLLSLVDEKLKMKDPEASEHLETL 2213
zebrafish         LGMVKDDLSWEDLIERAESVLALNENDHTIACHRSSIILSLIDEKLKMRDPAANQYLDIF 2213
                  ***.***: *:*::*** **  :*  **  ** ***::*.* *****  *. . :

                                                                    I
human             QTIRFLPFLTKPAGFSLDWKGNSFKPETMFAATDLYTAEHQDIVCLLQPILNENSHSFRG 2270
orangutan         QTIPFLPFLTKPAGFSLDWKGNSFKPETMFAATDLYTAEYQDIVCLLQPILNENSHSFRG 2270
mouse             QTIPFLPFLTKPAGFSLEWKGNSFKPETMFAATDIYTAEYQDIVCLLQPILNENSHSFRG 2272
rat               QTIPFLPFLTKPAGFSLEWKGNSFKPETMFAATDLYTAEYQDIVCLLQPILNENSHSFRG 2271
dog               QTIPFLPFLTKPAGFSLDWKGNSFKPETMFAAIDLYTAEHQDIVCLLQPILNENSHSFRG 2268
horse             QTIPFLPFLTKPAGFSLDWKGNSFKPETMFAATDLYTAEHQDIVCLLQPILNENSHSFRG 2267
chicken           QTIPFLPFLSKPAGFSLHWKGSDFPPDAMFSATDLFTADHQDIVCLIQPILNENSHSFKG 2265
zebra finch       QNIPFLPFLSKPAGFSLHWKGSDFQPEAMFSANDLFTADHQDIVCLIQPILNENSHSFKG 2270
anole lizard      QTIPFLPFLTKPAGFSLHWKGNDFESETMFSAVDLFTADYQDIVCLLKPILNENSHSFKG 2264
fugu              QAIKFLPFLTRPAGFSLPWHGNNFSPTTMFSARELLTIEHQDTVCLMKPILNENSPSFRG 2273
tetraodon         QDIKFLPFLTRPAGFSLPWHGNNFSPATLFSARELFTSEHQDTVCLMKPILNENSPSFRG 2270
stickleback       HDTKFLPFLTRPAGFSLPWHGNNFPPTKMFSAKDLFTTEHQETVCLMMPILNENSPSFKG 2273
medaka            QDIKFLPFLTRPAGFSLPWHGNSFSPATMFSPREVFTTEHQDTVCLMKPILNENSPSFKG 2273
zebrafish         QNIKFLPFLTKPAGFSLPWHGNNFPQLTMFSAKELFTTEHQDTVCLMNPILNENSPSFKG 2273
                  :   *****::****** *:*..*    :*:. :: * ::*: ***: ******* **:*

                         G                                     A
human             CGSVSLAVKEFLGLLKKPTVDLVINQLKEVAKSVDDGITLYQENITNACYKYLHEALMQN 2330
orangutan         CGSVSLAVKEFLGLLKKPTVDLVINQLKEVAKSVHDGITLYQENITNACYKYLHEALMQN 2330
mouse             CGSVSLAVKEFLGLLKKPTVDLVINQLKQVAKSVDDGITLYQENITNACYKYLHEAVLQN 2332
rat               CGSVSLAVKEFLGLLKKPTVDLVINQLKQVAKSVDDGITLYQENITNACYKYLHEAILQN 2331
dog               CGSVSLAVKEFLGLLRKPTVDLVVNQLKEVAKSVDDGVTLYQENITNACYKYLHDAMMQN 2328
horse             CGSVPLAVKEFLGLLRKPTVDLVVNQLKEVARSVDDGVTLYQENITNACYKYLHDAMMQN 2327
chicken           CGALSLAVKEFLGLLKKPAVNLVINQLEEVAKSFDG-ITLYQENITNACYKHLHEAMLEN 2324
zebra finch       CGTLSLAVKEFLGLLKKPAVNLVINQLEEVAKSFDG-ITLYQENITNACYKYLHEAMLES 2329
anole lizard      CGNISLAVKDFLGLLKKPTVIMVINQLKVVAKHFDG-ITLYQENITNACYKYLHEALLQN 2323
fugu              CGTMSLAVKDGLGLIRKPSVELVISQLKKLSQSFDG-ITLYQENITNACYKFLHEEMLQD 2332
tetraodon         CGTVSLAVKDGLGLIRTPSVELVISQLKKLSQSFDG-VTLYQENITNACYKFLHEEMLQD 2329
stickleback       CGAMSLAVKDCLGLIRKPSVGLVISQLKKLSQSFDG-VTLYQENITNACYKFLHEEMLQD 2332
medaka            CGAMSLAVKDGLGLIRKPSVALVISQLKKLSQSFDG-VTLYQENITNACYKYLHEEMFQD 2332
zebrafish         CGPISLAVKDFLGLIRKPTVSLVISQLKELSKSFDG-VTLYQENITNACYKYLHEELLQS 2332
                  ** :.****: ***::.*:* :*:.**: ::: ... :*************.**: :::.

                                                             S     K
human             EITKMSIIDKLKPFSFILVENAYVDSEKVSFHLNFEAAPYLYQLPNKYKNNFRELFETVG 2390
orangutan         EITKISIIDKLKPFSFILVENAYVDSEKVSFHLNFEAAPYLYQLPNKYKNNFRELFESVG 2390
mouse             EMAKATIIEKLKPFCFILVENVYVESEKVSFHLNFEAAPYLYQLPNKYKNNFRELFESVG 2392
rat               EMAKATIIEKLKPFCFILVENVYVESEKVSFHLNFEAAPYLYQLPNKYKNNFRELFESVG 2391
dog               EMIKISIIEKLKPFSFILVENAYVDSEKVSFHLNFEAAPYLYQLPNKYKNNFRELFESVG 2388
horse             EVAKMSIVEKLKLFNFILVENAYVDSERVSFHLNFEAAPYLYQLPNKYKNNFRELFESVG 2387
chicken           ESTKAMIIEQLTNCSFILVENVYVDPTKVSFHLNFEAAPYLYQLPNKYKNSFRELFESMG 2384
zebra finch       ESTKAMIIEQLTNSSFILVENVYADPSKVSFHLNFEAAPYLYQLPNKYKNSFRELFESVG 2389
anole lizard      GATKIVIVEELKNYSFILVEDGYVDPTRVSFHLNFEAAPYLHQLPNKYRNSFRELFESVG 2383
fugu              ECAKEQIAEELTAFNSILVENTYVSPAKIAFHLNFDASPHLYQLPNKYRSSCRELFENVG 2392
tetraodon         ERAKQQMAEELTAFNSILVENTYVSPAKIAFHLNFDASPHLYQLPNKYRNSCRELFESVG 2389
stickleback       KKATEQISEELKEFNSILVENTYVNPSKVAFHLNFDAAPHLYQLPNKYRNSCRELFENVG 2392
medaka            ENAKDLITEELKAFNSILVENTYVSPSKVAFHLNFDVAPHLYQLPNKYRNSCRELFENVG 2392
zebrafish         NAAKEEIMEELKTFSSILVENTYVEPLKVAFNLNFDAAPYLYQLPNKYRNSCRELFENVG 2392
                     .  : ::*.    ****: *... :::*:***:.:*:*:******:.. *****.:*
```

```
                        R A                            X       G
human               VRQSCTVEDFALVLESIDQERGTKQITEENFQLCRRIISEGIWSLIREKKQEFCEKNYGK 2450
orangutan           VRQSFTVEDFALVLESIDQERGTKQITEENFQLCRRIISEGIWSLIREKKQEFCEKNYGK 2450
mouse               VRQSFTVEDFALVLESIDQERGKKQITEENFQLCRRIISEGIWSLIREKRQEFCEKNYGK 2452
rat                 VRQSFTVEDFALVLESIDQERGKKQITEENFQLCRRIISEGIWSLIREKRQEFCEKNYGK 2451
dog                 VRQSFTVEDFALVLESIDQEKGTKQITEENFQLCRRIISEGIWSLIREKKQEFCEKNYGK 2448
horse               VRQSFTVEDFALVLESIDQEKGTKQITEENFQLCRRIISEGIWSLIREKKQEFCEKNYGK 2447
chicken             VRQSFTVEDFALVLELINQERGTKQLTEENFQLCRRIISEGIWSLIREKKQEFCVKKYGE 2444
zebra finch         VRQAFTVEDFAVVLELINQERGTKQLTEDNFQLCRRIISEGIWGLIREKKQEFCEKKYGE 2449
anole lizard        VHQAFTVEDFAQVLGSVNQETENKSLTEENFQLCRRIISEGIWSLIREKKQELCEKKYGE 2443
fugu                VKSSFSVDDFSGILETVKQGCGRRALSEDNFQLCRRVISEGIWSLIRDKSQEYCQENHGG 2452
tetraodon           VKSSFSVEDFSGILETVKQGCGRRALSEDNFQLCRRVISEGIWSLIRDKSQEHCKEHHSG 2449
stickleback         VQASFTVEDFATVLETVKEECGRRVLTEENFQLCRRIISEGIWSLIRDKNQEYCQAHFGG 2452
medaka              VQPNFTVEDFSTVLETVRQECGRRALTEENFQLCRRIISEGIWSLIRDKNQEYCQANYGG 2452
zebrafish           VHASFTVETFSGVLQLIKNECGRKPLTEENFQLSRRIISEGIWSLIRDKSQEFCLSNYGE 2452
                    *:   :*: *: :*  : :    : ::*:****.**:******.***:* ** *  :..

                               S                                       X       T
human               ILLPDTNLMLLPAKSLCYNDCPWIKVKDTTVKYCHADIPREVAVKLGAVPKRHKALERYA 2510
orangutan           ILLPDANLMLLPAKSLCYNDCPWIKVKDTTVKYCHADIPREVAVKLGAVPKRHKALERYA 2510
mouse               ILLPDTNLLLLPAKSLCYNDCPWIKVKDSTVKYCHADIPREVAVKLGAIPKRHKALERYA 2512
rat                 ILLPDTNLLLLPAKSLCYNDCPWIKVKDSSVKYCHADIPREVAVKLGAIPKRHKALERYA 2511
dog                 ILLPDTNLKLLPAKSLCYNDCPWIKVKDTTVKYCHADIPREVAVKLGAVPKRHKALERYA 2508
horse               ILLPDSNLKLLPAKSLCYNDCPWIKVRDTTVKYCHADIPREVAVKLGAVPKRHKALERYA 2507
chicken             ILLPDTRFALLPAKSLCYNDCPWIKVKDTTVKYCHADIPREVAVKLGAIPKRHKALERYA 2504
zebra finch         ILLPDTRLALLPAKSLCYNDCPWIKVKDTTVKYCHGDIPREVAVKLGAIPKRHKALERYA 2509
anole lizard        ILLPDTCLALLPANSLCYNDCPWIKVKDTSVKYCHPDIPREVAVKLGAIPKRHKALERYA 2503
fugu                ILLPDSNLTLQPSKSLCYNDCPWIKVRDTTVKYCHGDIPREVAVKLGAVPKRHKALERYA 2512
tetraodon           ILLPDSNLTLQPSKSLCYNDCPWIKVRDTTVKYCHGDIPREVAVKLGAVPKRHKALERYA 2509
stickleback         ILLPDFNLTLQPSKSLCYNDCPWIKVRDSSVKYCHGDIPREVAVKLGAVPKRHKALERYA 2512
medaka              ILLPDSNLTLQPSKSLCYNDCPWIKVRDSSVKYCHSDIPREVAVKLGTIPKRHKALERYA 2512
zebrafish           ILLPDSNFTLQPSKSLCYNDCPWIKVRDTTVKYCHGDIPREVAVKLGAIPKRHKALERYA 2512
                    *****  : * *::************:*::***** ***********::***********

                                                                   V
human               SNVCFTTLGTEFGQKEKLTSRIKSILNAYPSEKEMLKELLQNADDAKATEICFVFDPRQH 2570
orangutan           SNVCFTTLGTEFGQKEKLTSRIKSILNAYPSEKEMLKELLQNADDAKATEICFVFDPRQH 2570
mouse               SNICFTALGTEFGQKEKLTSRIKSILNAYPSEKEMLKELLQNADDAKATEICFVFDPRQH 2572
rat                 SNICFTAPGTEFGQKEKLTSRIKSILNAYPSEKEMLKELLQNADDAKATEICFVFDPRQH 2571
dog                 SNICFTTLGTEFGQKEKLTSRIKSILNAYPSEKEMLKELLQNADDAKATEICFVFDPRQH 2568
horse               SNICFTTLGTEFGQKEKLTSRIKSILNAYPSEKEMLKELLQNADDAKATEICFVFDPRQH 2567
chicken             SNICFTTLGTEFGQKEKLTSRIKSILNAYPSEKEMLKELLQNADDAKATEICFVFDPRQH 2564
zebra finch         SNICFTTLGTEFGQKEKLTSRIKSILNAYPSEKEMLKELLQNADDAKATEICFVFDPRQH 2569
anole lizard        SNICFTTLGTEFGQKEKLTGRIKSILNAYPSEKEMLKELLQNADDAKATEICFVFDPRQH 2563
fugu                SNICFTPPGSEFGQKEKLTSRIKSILSAYPSEKEMLKELLQNADDAKATEIFFIYDPRTH 2572
tetraodon           SNICFTPPGSEFGQKETLTSRIKSILSAYPSEKEMLKELLQNADDAKATEIYFIYDPRTH 2569
stickleback         SNVCFTTLGTEFGQKEKLTSRIKSILNAYPSEKEMLKELLQNADDAKATEIYFVFDPRTH 2572
medaka              SNVCFTTLGSEFGQKEKLTSRIKSILKAYPSEKEMLKELLQNADDAKATEIFFVFDSRTH 2572
zebrafish           SNICFTTLGSEFGQKEKLTSRIKSILNAYPSEKEMLKELLQNADDAKATEIYFVFDPRTH 2572
                    **:***. *:******.**.******.************************ *::*.* *

                                                                            S
human               PVDRIFDDKWAPLQGPALCVYNNQPFTEDDVRGIQNLGKGTKEGNPYKTGQYGIGFNSVY 2630
orangutan           PVDRIFDDKWAPLQGPALCVYNNQPFTEDDVRGIQNLGKGTKEGNPYKTGQYGIGFNSVY 2630
mouse               PVDRIFDDKWAPLQGPALCVYNNQPFTEDDVRGIQNLGKGTKEGNPCKTGQYGIGFNSVY 2632
rat                 PVDRIFDDKWAPLQGPALCVYNNQPFTEDDVRGIQNLGKGTKEGNPCKTGQYGIGFNSVY 2631
dog                 PVDRIFDDKWAPLQGPALCVYNNQPFTEDDVRGIQNLGKGTKEGNPCKTGQYGIGFNSVY 2628
horse               PVDRIFDDKWAPLQGPALCVYNNQPFTEDDVRGIQNLGKGTKEGNPCKTGQYGIGFNSVY 2627
chicken             PADRIFDEKWAPLQGPALCVYNNQPFTEDDIRGIQNLGKGTKVGNPCKTGQYGIGFNSVY 2624
zebra finch         PADRIFDEKWAPLQGPALCVYNNQPFTEDDIRGIQNLGKGTKVGNPCKTGQYGIGFNSVY 2629
anole lizard        STDRIFDDKWAPLQGPALCVYNNQPFTEDDIRGIQNLGKGTKEGNPCKTGQYGIGFNSVY 2623
fugu                PTDRVFDDKWIPMQGPALCVYNNQPFTEDDVRGIQNLGRGTKEANPGKTGQYGIGFNSVY 2632
tetraodon           PTDRVFDDKWIPMQGPALCVYNNQPFTEDDVRGIQNLGRGTKEANPGKTGQYGIGFNSVY 2629
stickleback         PTDRIFDDKWIPMQGPALCVYNNQPFTEDDVRGIQNLGRGTKEANPGKTGQYGIGFNSVY 2632
medaka              PTDRIFDDKWVPMQGPALCVYNNQPFTEDDVRGIQNLGRGTKEANPGKTGQYGIGFNSVY 2632
zebrafish           STDRIFDDKWTPLQGPSLCVYNNQPFTEDDIRGIQNLGRGTKEANPGKTGQYGIGFNSVY 2632
                    ..**:**:** *:***:*************:*******:*** .** *************

human               HITDCPSFISGNDILCIFDPHARYAPGATSISPGRMFRDLDADFRTQFSDVLDLYLGTHF 2690
orangutan           HITDCPSFISGNDILCIFDPHARYAPGATSISPGRMFRDLDADFRTQFSDVLDLYLGTHF 2690
mouse               HITDCPSFISGNDILCIFDPHARYAPGATSVSPGRMFRDLDADFRTQFSDVLDLYLGNHF 2692
rat                 HITDCPSFISGNDILCIFDPHARYAPGATSVSPGRMFRDLDADFRTQFSDVLDLYLGNHF 2691
dog                 HITDCPSFISGNDILCIFDPHARYAPGATSVSPGRMFRDLDADFRTQFSDVLDLYLGTHF 2688
horse               HITDCPSFISGNDILCIFDPHARYAPGATSISPGRMFRDLDADFRTQFSDVLDLYLGSHF 2687
chicken             HITDCPSFISGNDILCIFDPHARYAPGATSTSPGRMFRDLDADFRTQFSDVLDLYLGDHF 2684
zebra finch         HITDCPSFLSGNDILCIFDPHARYAPGSTSTSPGRMFRDLDADFRTQFSDVLDLYLGNHF 2689
anole lizard        HLTDCPSFVSGNDILCIFDPHARYAPGATSISPGRMFRDLDADFRTQFSDVLDLYLGDHF 2683
fugu                YITDCPSFISNSDILCIFDPHAQYAPGATSASPGRMFRDLDSDFRSQFSDVLNLYLGAHF 2692
tetraodon           HITDCPSFISNNDILCIFDPHAQYAPGATSASPGRMFRDIDSDFRSQFSDVLNLYLGANF 2689
stickleback         HITDCPSFISNNDILCIFDPHAQYSPGATSTSPGRMFRDLDSDFRSQFSDVLSLYLGVHF 2692
medaka              HITDCPSFVSNNDILCIFDPHAQYAPGATSTSPGRMFRDLDSDFRSQFSDVLNLYLGDHF 2692
zebrafish           HITDCPSFISNNDILCIFDPHARYAPGATSVSPGRMFRDLDSDFRSQFSDVLNLYLGNHF 2692
                    ::******:*..**********:*:**:** ********:*:***:******.**** :*
```

```
                              C     N                                       V
human             KLDNCTMFRFPLRNAEMAKVSEISSVPASDRMVQNLLDKLRSDGAELLMFLNHMEKISIC 2750
orangutan         KLDNCTMFRFPLRNAEMAKVSEISSVPASDRMVQNLLDKLRSDGAELLMFLNHMEKISIC 2750
mouse             KLDNCTMFRFPLRNAEMAQVSEISSVPSSDRMVQNLLDKLRSDGAELLMFLNHMEKISIC 2752
rat               KLDNCTMFRFPLRNAEMAQVSEISSVPSSDRMVQNLLDKLRSDGAELLMFLNHMEKISIC 2751
dog               KLDNCTMFRFPLRNAEMAKVSEISSVPSSDRMVQNLLDKLRSDGAELLMFLNHMEKISIC 2748
horse             KLDNCTMFRFPLRSAEMAKVSEISSVPSSDRMVQNLLDKLRSDGAELLMFLNHMEKISIC 2747
chicken           KLDNCTMFRFPLRNGEMAKVSEISTVPCSDRMVQNLLDKLRTDGAELLMFLNHMEKISIC 2744
zebra finch       KLDNCTMFRFPLRNGEMAKVSEISSVPCSDRMVQNLLDKLRTDGAELLMFLNHMEKISIC 2749
anole lizard      KLENCTMFRFPLRNGEMAKVSEISPVPCSDRMVQNLLDKLRSDGAELLMFLNHMEKISIC 2743
fugu              KLERSTMFRFPIRSTEMAKTSEISSLPASDRMVQNLLDKLKIDGAELLMFLNHMEKISIC 2752
tetraodon         KLERSTMFRFPIRSTEMAKTSEISSVPASDRMVQNLLDKLKSDGAELLMFLNHMEKISIC 2749
stickleback       KLERSTMFRFPIRSKGMAKISEISSVPASDRMVQNLLDKLKTDGAELLMFLNHMEKISIC 2752
medaka            KLERSTMFRFPIRTVEMAKTSEISSVPASDRMVHNLLDKLKTDGAELLMFLNHMEKISIC 2752
zebrafish         KLERSTMFRFPIRNVEMARISEISSAPASDRMVQNFLDKIRMDGAELLMFLNHMEKISIC 2752
                  **:..******:*.  **: ****. *.*****:*:***:: ******************

                                                 V
                                                 T
                                                 G
                                                 E
                                                 S
                              X                C P               Q
human             EIDKSTGALNVLYSVKGKITDGDRLKRKQFHASVIDSVTKKRQLKDIPVQQITYTMDTED 2810
orangutan         EIDKSTGALNVLYSVKGKITDGDRLKRKQFHASVIDSVTKKRQLKDIPVQQITYTMDTED 2810
mouse             EIDKATGGLNVLYSVKGKITDGDRLKRKQFHASVIDSVTKKRQLKDIPVQQITYTMDTED 2812
rat               EIDKATGGLNVLYSVKGKITDGDRLKRKQFHASVIDSVTKKRQLKDIPVQQITYTMDTED 2811
dog               EIDKGTGALNVLYSVKGKITDGDRLKRKQFHASVIDSVTKKRQLKDIPVQQITYTMDTED 2808
horse             EIDKASGALNVLYSVKGKITDGDRLKRKQFHASVIDSVTKKRQLKDIPVQQITYTMDTED 2807
chicken           EIEKTTGALNVLYSVQGKITDGDRLKRKQFHASVIDSVTKKKQLSEIPVQQITYTMDTED 2804
zebra finch       EVEKTTGALNVLYSVQGKITDGDRLKRKQFHASVIDSVTKKKQLSEIPVQQITYTMDTED 2809
anole lizard      EIEKATGTLNVLYSVTGKITDGDRQKRKQFHASVIDSVTKKKQLNEIPVQQITYTMDIED 2803
fugu              EIDQSSGQPKVIYSVTARISDGDRLKRKQFHASVIDSVIKKKQLSAIPVQQITYSMDILD 2812
tetraodon         EIDQSSGQPRVIYSVTARISDGDRLKRKQFHASVIDSVMKKKPLSAIPVQQITYSMDILD 2809
stickleback       EIDSASGALKVLYSVTAKITDGDRLKRKQFHASVIDSVTKKKQLTAIPVQQITYTMDIED 2812
medaka            EIDKSSGDLKVLYSVTAKITDGDRLKRKQFHASVLDSVTKKKQLTAIPVQQISYSMDIED 2812
zebrafish         EIENGTGDLKTLYSVTAKITDGDRLKRKQFHASVLDSVYKKRQLTQIPVQQITYTMDIED 2812
                  *::. :*  ..:*** .:*:**** *********:*** **: *. ******:*:**  *

                                   G
human             SEGNLTTWLICNRSGFSSMEKVSKSVISAHKNQDITLFPRGGVAACITHNYKKPHRAFCF 2870
orangutan         SEGNLTTWLICNRSGFSSMEKVSKSVISAHKNQDITLFPRGGVAACITHNYKKPHRAFCF 2870
mouse             SEGNLTTWLICNRSGFSSMEKVSKSVISAHKNQDITLFPRGGVAACITHNYKKPHRAFCF 2872
rat               SEGNLTTWLICNRSGFSSMDKVSKSVISAHRNQDITLFPRGGVAACITHNYKKPHRAFCF 2871
dog               SEGNLTTWLICNRSGFSSMEKVSKSVISAHKNQDITLFPRGGVAACITHNYKKPHRAFCF 2868
horse             SEGNLTTWLICNRSGFSSMEKVSKSVISAHKNQDITLFPRGGVAACITHNYKKPHRAFCF 2867
chicken           SEGNLTTWLICNRSGFSAMEKVSKSVVSAHKNEDITLFPRGGVAACITHNYKKPHRAFCF 2864
zebra finch       SEGNLTTWLICNRSGFSAMEKVSKSVVSAHKNEDITLFPRGGVAACITHNYKKPHRAFCF 2869
anole lizard      SEGNLTSWLICNRSGFSSIDKVSKSVISAHKNKDITLFPRGGVAACITHNYKKPHRAFCF 2863
fugu              TDGNSTTWMICNRSGFPNMENVSKGLVSAHKNEDITLFPRGGVAACVSHNYKKPHRAFCF 2872
tetraodon         TDGNSTTWMICNRSGFPNIENVSKALVSAHKNEDITLFPRGGVAACVSHNYKKPHRAFCF 2869
stickleback       NDGNLTSWMICNRSGFPDIENVSKSVISAHKNEDITLFPRGGVAACVSHNYKKPHRAFCF 2872
medaka            TDGNLTTWVICNRSGFSDMETVSKSIISAHKNDDITLFPRGGVAACLSHNYKKPHRAFCF 2872
zebrafish         TDGNSTTWLICNRSGFSDMERVSKSVISAHKNEDITLFPRGGVAACTSHNYKKTHRAFCF 2872
                  .:** *:*:*******. :: ***.::***:*.************* :*****.******

                                        X
human             LPLSLETGLPFHVNGHFALDSARRNLWRDDNGVGVRSDWNNSLMTALIAPAYVELLIQLK 2930
orangutan         LPLSLETGLPFHVNGHFALDSARRNLWRDDNGVGVRSDWNNSLMTALIAPAYVELLIQLK 2930
mouse             LPLSLETGLPFHVNGHFALDSARRNLWRDDNGVGVRSDWNNSLMTALIAPAYVELLIQLK 2932
rat               LPLSLETGLPFHVNGHFALDSARRNLWRDDNGVGVRSDWNNSLMTALIAPAYVELLIQLK 2931
dog               LPLSLETGLPFHVNGHFALDSARRNLWRDDNGVGVRSDWNNSLMTALIAPAYVELLIQLK 2928
horse             LPLSLETGLPFHVNGHFALDSARRNLWRDDNGVGVRSDWNNSLMTALIAPAYVELLIQLK 2927
chicken           LPLSLETGLPFHVNGHFALDSARRNLWRDDNGVGVRSDWNNSLMTALIAPAYVELLIQLK 2924
zebra finch       LPLSLETGLPFHVNGHFALDSARRNLWRDDNGVGVRSDWNSSLMTALIAPAYVELLIQLK 2929
anole lizard      LPLSLETGLPFHVNGHFALDSARRNLWRDDNGVGVRSDWNNSLMTALIAPAYVELLIQLK 2923
fugu              LPLSLETGLPFHVNGHFALDSARRNLWRDDNGVGVRSDWNNNLMTLLIAPACVELLIQLK 2932
tetraodon         LPLSLETGLPFHVNGHFALDSARRNLWRDDNGVGVRSDWNTNLMTLLIAPACVELLIQLK 2929
stickleback       LPLSLETGLPFHVNGHFALDSARRNLWRDDNGVGVRSDWNNNLMTSLIAPAIVELLIQLK 2932
medaka            LPLSLETGLPFHVNGHFALDSARRNLWRDDNGVGVRSDWNNTLMTSLIAPAYVELLIQLK 2932
zebrafish         LPLSLETGLPFHVNGHFALDSARRNLWRDDNGVGVRSDWNNNLMKVLIAPACVELLIQLK 2932
                  ****************************************..**. ***** ********

                                             R
human             KRYFPGSDPTLSVLQNTPIHVVKDTLKKFLSFFPVNRLDLQPDLYCLVKALYNCIHEDMK 2990
orangutan         KRYFPGSDPTLSVLQNTPIHVVKDTLKKFLSFFPVNRLDLQPDLYCLVKALYNCIHEDMK 2990
mouse             KRYFPGSDPTLSVLQNTPIHVVKDTLKKFLSFFPVNRLDLQPDLYCLVKALYSCIHEDMK 2992
rat               KRYFPGSDPTLSVLQNTPIHVVKDTLKKFLSFFPVNRLDLQPDLYCLVKALYSCIHEDMK 2991
dog               KRYFPGSDPTLSVLQNTPIHVVKDTLKKFLSFFPVNRLDLQPDLYCLVKALYSCIHEDMK 2988
horse             KRYFPGSDPTLSVLQNTPIHVVKDTLKKFLSFFPVNRLDLQPDLYCLVKALYSCIHEDMK 2987
chicken           KRYFPGTDPTVSVLQNTPVHAVKDTLKKFLSFFPVNRLDVQPDWYCLVKAVYSCIYEDLK 2984
zebra finch       KRYFPGTDPTVSVLQNTPIHAVKDTLKKFLSFFPVNRLDIQPDWYCLVKAVYSCIYEDLK 2989
anole lizard      KRYFQGTDPTVSVLQNTSMHAVKDTLKKFLSFFPVNRLDLQPDWYCLVKAVYSCIHEESK 2983
fugu              RRYFPGPDPTMTILQGTPLHMVKDTLRKYLFFFPANRLDIQPDWYCLVKAVYSCIHSDLK 2992
tetraodon         RRYFPGPDPTMTILQGTPLHAVKDTLRKYLFFFPANRLDIQPDWYCLVKAVYHCVHADLK 2989
stickleback       RRSFQGPDPTITVLQGTPLHVVKDTLRKYLFFFPVNRVDIQPDWYCLVKAVYNCIHADMK 2992
medaka            RRYFPGPDPTMTMLQGTPLHAVKDTLRKYLFFFPANRLETQPDWYCLVKAIYSCIHADLK 2992
zebrafish         RRLFPGPDPTMTIFQGTPIHVVRDILRKYLSFFPVNRLDIQPDWYCLVKAIYLCIHADLK 2992
                  :* * *.***::::*.*.:* *:* *:*:* ***.**:: *** ******:* *:: : *
```

```
human               RLLPVVRAPNIDGSDLHSAVIITWINMSTSNKTRPFFDNLLQDELQHLKNADYNITTRKT 3050
orangutan           RLLPVVRAPNIDGSDLHSAVIITWINMSTSNKTRPFFDNLLQDELQHLKNADYNITTRKT 3050
mouse               RLLPVVRAPNIDGSDLHSAVIITWINMSTSNKTRPFFDNLLQDELQHLKNADYNITTRKT 3052
rat                 RLLPVVRAPNIDGSDLHSAVIITWINMSTSNKTKPFFDNLLQDELQHLKNADYNITTRKT 3051
dog                 RLLPVVRAPNIDGSDLHSAVIITWINMSTSNKTRPFFDNLLQDELQHLKNADYNITTRKT 3048
horse               RLLPVVRAPNIDGSDLHSAVIITWINMSTSNKTRPFFDNLLQDELQHLKNADYNITTRKT 3047
chicken             RLLPVMRAPNVDGSDLHSAVIITWVNMSTVNKGRPFFDNLLQDELQHLKNTEYNITTRKT 3044
zebra finch         RLLPVMRAPNVDGSDLHSAVIITWVNMCAVNKGRPFFDNLLQDELQHLKNTEYNITTRKT 3049
anole lizard        RLLPVVRAPNVDGSDLHSAVIITWINMSTTNKTRPFFDNLLQDELHQLKNVEYNITTRKT 3043
fugu                RLLPVVRAAHSENSDMHSVIYILWVNTSTAKKSRAFFDNLLQDELQHLKNAEYNITSRKS 3052
tetraodon           RLLPVLRAAPSDNSDMHSVIYILWVNTCTANKGRAFFDNLLQDELQHLKNTEYNVTSRKS 3049
stickleback         RLLPVVRAVHMENSDMHSVIYISWVNTSTANKGRAFFDNLLQDELEHLKNTEYNITSRKS 3052
medaka              RLLPVVRTTQLDNSEMHSVVYISWVNTSTANKGRAFFDNLLQDELQHLKNTEYNITSRKS 3052
zebrafish           RLLPVVRAPHLDNSDMHSAVYISWVNTSTSTKGKAFFDNLLQDELQHLKNTEYNITTRKS 3052
                    *****:*:   :.*::**.: * *:* .: .* :.**********.:***.:**:*:**:

human               VAENVYRLKHLLLEIGFNLVYNCDETANLYHCLIDADIPVSYVTPADIRSFLMTFSSPDT 3110
orangutan           VAENVYRLKHLLLEIGFNLVYNCDETANLYHCLIDADIPVSYVTPADIRSFLMTFSSPDT 3110
mouse               VAENVYRLKHLLLEIGFNLVYNCDETANLYHCLVDADIPVSYVTPADVRSFLMTFSSPDT 3112
rat                 VAENVYRLKHLLLEIGFNLVYNCDETANLYHCLVDAAIPVSYVTPADVRSFLMTFSSPDT 3111
dog                 VAENVYRLKHLLLEIGFNLVYNCDETANLYHCLIDADIPVSYVTPADVRSFLMTFSSPDT 3108
horse               VAENVYRLKHLLLEIGFNLVYNCDETANLYHCLIDADIPVSYVTPADVRSFLMTFSSPDT 3107
chicken             VAENVYRLKHLLLEIGFNLVYNCDETANLYHCLVDADIPVTYVTPADVRLFLMTFSFPDS 3104
zebra finch         VAENVYRLKHLLLEIGFNLVYNCDETANLYHCLVDADIPVTYVTPADVRLFLMTFSFPDS 3109
anole lizard        VAENVYRLKHLLLEIGFNLIYSCDETANLYHCLVDADIPVSYVTPADVRSFLLTFSSPDS 3103
fugu                VAENVYRLKTLLLDIGFNLIHSCEETANLYTCLEATGIPVSYVTPKDVRNFLYTFSSPDT 3112
tetraodon           VAENVYRLKTLLLDVGFNLIYSCDETANLYACLEAAGIPVSYVTPRDVRNFLYTFSSPDT 3109
stickleback         VAENVYRLKTLLLDIGFNLVHSCDETANLYFCLEDAGIPVSYVTPDDVRHFLHTFLSPDT 3112
medaka              VAENVYRLKTLLLDIGFNLIHSCDETSNIYFCLEDAGIPVSYVTPTDVRNFLQTFSSPDT 3112
zebrafish           VAENVFRLKAMLLDVGFNLIHSCDETASLYFCLEDAGIPVNYVTPEDVRSFLLTFSSPDT 3112
                    *****:*** :**::****::.*:**:.:* **  : ***.**** *:* ** **  **:

human               NCHIGKLPCRLQQTNLKLFHSLKLLVDYCFKDAEENEIEVEGLPLLITLDSVLQTFDAKR 3170
orangutan           NCHIGKLPCRLQQTNLKLFHSLKLLVDYCFKDAEENEIEVEGLPLLITLDSVLQTFDAKR 3170
mouse               NCHIGKLPCRLQQTNLKLFHSLKLLVDYCFKDAEESEFEVEGLPLLITLDSVLQIFDGKR 3172
rat                 NCHIGKLPCRLQQTNLKLFHSLKLLVDYCFKDAEESEFEVEGLPLLITLDSVLQIFDGKR 3171
dog                 NCHIGKLPCRLQQTNLKLFHSLKLLVDYCFKDAEENEIEVEGLPLLITLDSVLQTFDAKR 3168
horse               NCHIGKLPCRLQQTNLKLFHSLKLLVDYCFKDAEENEIEVEGLPLLITLDSVLQTFDAKR 3167
chicken             NCHIGKLPCRLQQTNLKLFHSLKLLVDYCFKDAEENEIQIEGLPLLITLDNVLQIFDSKR 3164
zebra finch         NCHIGKLPCRLQQTNLKLFHSLKLLVDYCFKDAEENEIQIEGLPLLITLDNVLQVFDSKR 3169
anole lizard        NCHIGKLPCRLQQTNLKLFHSLKLLVDYCFKDAEENEIHIEGLPLLITLDSVLQVFDSKR 3163
fugu                TCHIGKLPCRIQHSHYKLLHNLKLLVDYCFKDVDVDEVKIEGLPLLITIDNMLQVFDSKR 3172
tetraodon           TCHVGKLPCRIQHSHYKLLHNLKLLVDYCFKDMEVDEVKLEGLPLLITMDNMLQVFDSKR 3169
stickleback         SCHVGKLPCRIQQSNFKLVHNLKLLVDYCFKDREAGDLTIEGLPLLITMDNMLQVFDSKR 3172
medaka              SCHVGKLPCRLQQSNYRLLHSLKLLVDYCFTDIEEGEVKIEGLPLLITMDGMLQVFDSNR 3172
zebrafish           SCQVGKLPCRLQQSNFKLFHGLKLLVDYCFKDVEENDINIEGLPLLMTMDNILQVFDSRH 3172
                    .*::******:*::: :*.*.*********.* : .:. :******:*:*.:** **..:

                                                                 X
human               PKFLTTYHELIPSRKDLFMNTLYLKYSNILLNCKVAKVFDISSFADLLSSVLPREYKTKS 3230
orangutan           PKFLTTYHELIPSRKDLFMNTLYLKYSNILLNCKVAKVFDISSFADLLSSVLPREYKTKS 3230
mouse               PKFLTTYHELIPSRKDLFMNTLYLKYSSVLLNCKVAKVFDISSFADLLSSVLPREYKTKN 3232
rat                 PKFLTTYHELIPSRKDLFMNTLYLKYSSVLLNCKVAKVFDISSFADLLSSVLPREYKTKN 3231
dog                 PKFLTTYHELIPSRKDLFMNTLYLKYSNILLNCKVAKVFDISSFADLLSSVLPREYKTKN 3228
horse               PKFLTTYHELIPSRKDLFMNTLFLKYSNILLNCKVAKVFDISSFADLLSSVLPREYKTKN 3227
chicken             PKFLTTYHELIPSRKDLFMNTLYLRYNNILLSSDVAKVFDIASFAELLSSVLPREYKTKS 3224
zebra finch         PKFLTTYHELIPSRKDLFMNTLYLRYNNILLSSEVAKVFDIRSFAELLSSVLPREYKTRN 3229
anole lizard        PKFLTTYHDLIPSRKDLFMNTLYLRYSSILLKCSVAKVFDISSFADLLSSVLPREYKARG 3223
fugu                PKFLTLHHELISSRKELFINTLFMRYSNILLKEGIAKNFDISSFGDLLGSVLPREYQTKV 3232
tetraodon           QKFLTVHHELVSSRKDMFINTLFMRYSSILMKQGIAKHFDISSFGDLLGSVLPREYRTKV 3229
stickleback         PKFLTTHHELISSRKEMFMNTLYIRYSNILLMAGAAKTFDISCFADLLGSVLPREYRTRI 3232
medaka              PKFLTTHHELISSRKEMFMNTLYLKYCNVLLKAQVAKNFDISSFGDLLGSVLPREYHTRV 3232
zebrafish           PKFLTTYHELIATRKELFMHTLYIRYSNILIKAGVAKTFDINSFGDLLGSVLPRECKARS 3232
                     **** :*:*:.:**::*::**:::* .:*:    ** *** .*.:**.****** :::

                                     R
human               CTKWKDNFASESWLKNAWHFISESVSVKEDQEETKPTFDIVVDTLKDWALLPGTKFTVSA 3290
orangutan           CTKWKDNFASESWLKNAWHFISESVSVKEDQEETKPTFDIVVDTLKDWALLPGTKFTVSA 3290
mouse               CAKWKDNFASESWLKNAWHFISESVSVTDDQEEPKPAFDVIVDILKDWALLPGTKFTVST 3292
rat                 CAKWKDNFASESWLKNAWHFISESVSVTDDQEEPKPAFDVIVDILKDWALLPGTKFTVSA 3291
dog                 CTKWKDNFASESWLKNAWHFISESVNVKEDQEETQPIFDIVVETLKDWALLPGTKFTVSA 3288
horse               CTKWKDNFASESWLKNTWHFISESVSVKEEQEETKPVFDVVVDTLKDWALLPGTKFTVSA 3287
chicken             CMKWKENFASESWLKNAWHFISESIAVKEEQEDIQAKFDGVVETLKDWTLLPGVKFTVSA 3284
zebra finch         CMKWKENFASESWLKSAWHFISESLNIKEEQEDTQAKFDGVIETLKDWTLLPGVKFTVSA 3289
anole lizard        CIRWKENFASESWLKNAWHFISESLHAKDDQDDVKPTFEDVVDTLKDWALIPGAKFTVSS 3283
fugu                PVKWRDTFASESWLKNVWNFISENIAIKEDQLERTLGFDTVADILKDWALLPGTKFMAR- 3291
tetraodon           PVKWRDTFASESWLKNVWNFISENVAAKGDQLEHTPGFDTAMDILKDWALLPGTKFLAK- 3288
stickleback         PVKWRDTFASESWLKSVWNFISENIAVKEDQVDIKPSFETLLDILKDWALLPGMKFMAR- 3291
medaka              PVKWRDTFANDSWLKNVWNFLSENIAVKEDQVDIKPSFDTMVDILKDWALLPGLKFIAR- 3291
zebrafish           SVKWKENFPTESWLKSSWHFISEQLANKDDQAENKLKFETILEILKDWALLPGIKFLVSG 3292
                      :*::.*..:****. *:*:**.:  . :* :    *:   : ****:*:** ** .
```

```
human             NQLVVPEGDVLLPLSLMHIAVFPNAQSDKVFHALMKAGCIQLALNKICSKDSAFVPLLSC 3350
orangutan         NQLVVPEGDVLLPLSLMHIAVFPNAQSDKVFHALMKAGCIQLALNKICSKDSAFVPLLSC 3350
mouse             SQLVVPEGDVLIPLSLMHIAVFPNAQSDKVFHALMKAGCIQLALNKICSKDSALVPLLSC 3352
rat               SQLVVPEGDVLIPLSLMHIAVFPNAQSDKVFHALMKAGCIQLALNKICSKDSALVPLLSC 3351
dog               NQLVVPEGDVLLPLSLMHIAVFPNAQSDKVFHALMKAGCIQLALNKICSKDSAFVPLLSC 3348
horse             NQLVVPEGDVLLPLSLMHIAVFPNAQSDKVFHALMKAGCIQLALNKICSKDSAFVPLLSC 3347
chicken           NHLVVPECDVLLPLSIMHIAVFPNAQSDKVFHALMKTGCIQLALNKICSKDSALVPLLSA 3344
zebra finch       NHLVVPESDVLLPLSIMHIAVFPNAQSDKVFHALMKTGCIQLALNKICSKDSALVPLLSG 3349
anole lizard      NQLVVPDCDVLLPLSLMHIALFPNSQTDKVFHALMKTGCIQLALNKICSKDNALVPLMSA 3343
fugu              DKIVVPEHDVLLPLSLMHLAIFPQGQNDKVFHTLMKSGCIQLALNKISVKENPIMPILAQ 3351
tetraodon         EKIVVPEHDVLLPLSLMHLAIFPQGQNDKVFHTLMKSGCIQLAVNKISVKENPIMPILAQ 3348
stickleback       DKIVVPEHDVLLPLSLMSIAIFPHGQNDKVFHTLMKAGCIQLAVTKICLKDNPIMPFLAQ 3351
medaka            DKIVVPDHDMLLPLSLMNIAIFPQGQNDKVFHTLMKAGCIQLAVNKICVKDNQIMHFLTQ 3351
zebrafish         NKLVVPEHDVLLPLSLIFAAIFPNGQNDKVFHTLMKGGCFQLAVNKICAKENPMLPLLAQ 3352
                  .::***: *:*:***::  *:**:.*.*****:*** **:***:.**. *:. :: :::

                                    A
human             HTANIESPTSILKALHYMVQTSTFRAEKLVENDFEALLMYFNCNLNHLMSQDDIKILKSL 3410
orangutan         HTANIESPTSILKALHYMVQTSTFRTEKLVENDFEALLMYFNCNLNHLMSQDDIKILKSL 3410
mouse             HTANIDSPASILKAVHYMVQTSTFRTEKLMENDFEALLMYFNCNLSHLMSQDDIKILKSL 3412
rat               HTANIDSPASILKAVHYMVQTSTFRTEKLVENDFEALLMYFNCNLSHLMSQDDLKILKSL 3411
dog               HTANIESPTSILKAVHYMVQTSTFRAEKLVENDFEALLMYFNCNLSHLMSQDDIKILKSL 3408
horse             HTANIESPASILKALHYMVQTSTFRTEKLVENDFEALLMYFNCNLSHLMSQDDIKILKSL 3407
chicken           STANIDNPSSILKAIQYMVQTSSFRTEKLAESDFEALLMYFNCNLCHLTSQDDIKILKSL 3404
zebra finch       RTANIDNPSSILKAIQYMVQTSTFKTEKLAESDFEALLMYFNCNLCHLTSQDDIKILKSL 3409
anole lizard      HTANIDNPSSILRAIHYMVQTSTFKTEKLAENDFEALLMYFNCNLNHLTSEDDIKILRSL 3403
fugu              HTANIENPQSILRAVEYMIQASAFKAASLTDKDFEALLLYFNCNLAGLT-QQNAQSLKLL 3410
tetraodon         HTANIENPQSILRAVEYMIQASAFKAASLSDRDFEALLSYFSCNLASLT-QQNAQSLKLL 3407
stickleback       HTANIENPSSVLKAVEYIIQTATFKTSNLTEKDFEALLLYFNCNLANLT-KQDAQSLKLL 3410
medaka            HTANIENPSSILKATEYMIQMSAFKTANLTDKDFEALLLYFNCNLVSLT-QQDAQSLKLL 3410
zebrafish         HTANIDNPSSILRALEYMIQISAFKTASLTDKDFDALLLYFNCNLSNLA-QDDIQCLKLL 3411
                   ****:.* *:*:* .*::* ::*:: .* : **:*** **.***  *  ::: : *: *

                                     X
human             PCYKSISGRYVSIGKFGTCYVLTKSIPSAEVEKWTQSSSSAFLEEKIHLKELYEVIGCVP 3470
orangutan         PCYKSISGRYVSIAKFGTCYVLTKSIPSAEVEKWTQSSSSAFLEEKIHLKELYEVIGCVP 3470
mouse             PCYKSISGRYMSIAKFGTCYVLTKSIPSAEVEKWTQSSSSAFLEEKVHLKELYEVLGCVP 3472
rat               PCYKSISGRYMSIAKFGTCYVLTKSIPSAEVEKWTQSSSSAFLEEKVHLKELYEVLGCVP 3471
dog               PCYKSISGRYMSIAKFGTCYVLTKSIPSAEVEKWTQSSLSAFLEEKIHLKELYEVLGCVP 3468
horse             PCYKSISGRYMSIAKFGACYVLTKSIPSAEVEKWTQSSPSAFLEEKIHLKELYEVLGCVP 3467
chicken           PCYKSISGRYISISKYGTCYVLTKSIPSVEVDRWTQSTSSAFLEEKIHLKDLYTVLGCVS 3464
zebra finch       PCYKSISGRYISIAKYGTCYVLTKSIPSVEVDRWTQSTSSAFLEEKVHLKDLYTMLGCVS 3469
anole lizard      PCYKSISGRYMSISKCGSCYVLTRSIPSVEVDRWTQSASAAFLEEKIHLKDLYTLLGCVP 3463
fugu              PCFKSIGGKYVSIANHGANYVLVRNIPTADMDRWAHNGSFAFLADCPQLKELYTFLGCMP 3470
tetraodon         PCFRSVGGKFMSIANYGASYVLVRNIPTADMDKWAHNGSSAFLADCPQLKELYSFLGCAP 3467
stickleback       PCFKSVSGRYISIANNGTNYVLVKSIPTADIDKWAHTAPLGFLADNQQLKDLYTFLGCVP 3470
medaka            PCFKSVSGRYISIASYGANYVLEKSPPTADIEKWAHTPSSSFLADIPQLKELYTFLGCMP 3470
zebrafish         PCYKAVSGRYICISNFGACYVLTKSIPSADIEKWAHNTSSAFLADNPQLKELYGFLGITP 3471
                  **::::.*:::.*.. *: *** :. *:.::::*::.   .** :  :**:** .:*  .

                            P
human             VDDLEVYLKHLLPKIENLSYDAKLEHLIYLKNRLSSAEELSEIKEQLFEKLESLLIIHDA 3530
orangutan         VDDLEVYLKHLLPKIENLSYDAKLEHLIYLKNRLSSAEELSEIKEQLFEKLESLLIIHDA 3530
mouse             VDDLEVYLKHLLPKIENLSYDAKLEHLIYLKNRLASIEEPSEIKEQLFEKLESLLIIHDA 3532
rat               VDDLEVYLKHLLPKIENLSYDAKLEHLIYLKNRLASIEELSETKEQLFEKLESLLIIHDA 3531
dog               VDDLEVYLKHLLPKIENLSYDAKLEHLIYLKNRLSSIEELSEIKEQLFEKLESLLIIHDA 3528
horse             VDDLEVYLKHLLPKIENLSYDAKLEHLIYLKNRLSSIEELSEIKEQLFEKLESLLIIYDA 3527
chicken           VDDLEVYLKHLLPKIESLSYDAKLEHLIYLKNRLSSIEETSEMKEQLFEKLESILIIHDA 3524
zebra finch       VDDLEVYLKHLLPKIESLSYDAKLEHLIYLKNRLTSIEETSEMKEQLFEKLESILIIHDA 3529
anole lizard      VDDLEVYLKHLLPKIESLSYDAKLEHLIYLKNRLCSIEESSEIKEQLFEKLESFLIIHDS 3523
fugu              IDDLEIYHHHVLPKFESFCYDAKVEHIVYLKERLLSIEEPCDLVDQLYEKLEGLMFIFDY 3530
tetraodon         IDDLEVYLHHVLPKFESFCYDAKVEHVAYLKERLVSMEEPSDFTDQLYEKLEGLMFIFDC 3527
stickleback       IDDLEIYLQYLLPKFESLSYDAKVEHIVYLKERLLSMEESCEMKNQLYEKLEGLLFIYDY 3530
medaka            IDDIEIYLHHLLPKFDSLSSDAKVEHIVYLKERLLSMEESCAMKEQLYEKLEGLSFIHDH 3530
zebrafish         IDDLEVYLNHVLPKFDILSHDAKIQHVIYLKERLLAMEEQVSIKDKLFEKLEGLSFIYDS 3531
                  :**:*:* :::***:: :. ***::*: ***:** : **     ::*:****.: :*.*

human             NSRLKQAKHFYDRTVRVFEVMLPEKLFIPNDFFKKLEQLIKPKNHVTF-MTSWVEFLRNI 3589
orangutan         NSRLKQAKHFYDRTVRVFEVMLPEKLFIPNDFFKKLEQLIKPKNHVTF-MTSWVEFLRNI 3589
mouse             NNRLKQAKHFYDRTVRVFEVMLPEKLFIPKEFFKKLEQVIKPKNQAAF-MTSWVEFLRNI 3591
rat               NNRLKQAKHFYDRTVRVFEVMLPEKLFIPKEFFKKLEQVIKPKNQVVF-MTSWVEFLRNI 3590
dog               NSRLKQAKHFYDRTVRVFEVMLPEKLFIPKDFFKKLEQLIKPKNQATF-MTSWVEFLRNI 3587
horse             NSRLKQAKHFYDRTVRVFEVMLPEKLFIPKDFFKKLEQLIKPKNQVAF-MTSWVEFLRNI 3586
chicken           SNRLKPAKYFYDRTVKVFEVMLPEKLFIPQDFFKKLEQITKPKSQAAF-LPSWVTFLRHI 3583
zebra finch       SNRLKPAKYFYDRTVKVFEVMLPEKFFIPQDFFKKLEQLTKPKNQAAF-LPSWVTFLRHI 3588
anole lizard      CNRLKPAKHFYDRTVRVFEVMLPEKLFIPQDFFKKLEQLTKPKNRAAF-LTSWVAFLRNM 3582
fugu              SNRLRPAKFFYDETVKVFQVMLPSKSFITSDFFKKVEQVSKPKNGALS-LASWITFLRNI 3589
tetraodon         SNRLRPAKFFFDETVRVFQVMLPAKSFITSDFFKKVEQVSRPKNGIASSLASWITFLRHI 3587
stickleback       SNRLRPTKFFYDETVKVFKVMLPTKSFIQSDFFKKVEQVSKPRNGTLL-LTSWITFLRNI 3589
medaka            SNRLRPVKFFFDQTVKVFEVMLPSKSFIPSEFFKKIEQLTKPKNGTLL-MTSWIIFLRNI 3589
zebrafish         TNRLKPAKAFFDQTVKVFEVMLPQKAFIPNDFFRKIEQVSKPKSGTSY-VPSWTTFLRNI 3590
                   .**: .* *:*.**:**:**** * ** .:**:*:**: :*:.     :.**  ***::
```

```
                                                               Q
                                                           M   X        P
human            GLKYILSQQQLLQFAKEISVRANTENWSKETLQNTVDILLHHIFQERMDLLSGNFLKELS 3649
orangutan        GLKYILSQQQLLQFAKEISVRANTENWSKETLQNTVDILLHHIFQERMDLLSGNFLKELS 3649
mouse            GLKYALSQQQLLQFAKEISVRANTENWSKETLQSTVDILLHHIFQERMDLLSGNFLKELS 3651
rat              GLKHVLSQQQLLQFAKEISVRANTENWSKETLQNTVDILLHHIFQERTDLLSGNFLKELS 3650
dog              GLKYILSQQQLLQFAKEISVRANTENWSKETLQNTVDILLHHIFQERMDLLSGNFLKELS 3647
horse            GLKYILSQQQLLQFAKEISVRATTENWSKETLQNTVDILLHHIFQERMDLLSGNFLKELS 3646
chicken          GLKYIISQQQLLQFAKEISMRANTENWSKETLQNAVDVLLHHIFQERTDLLSGNFLKELS 3643
zebra finch      GLKFIISQQQLLQFAKEISMRANTENWSKETLQNTVDVLLHHIFQERADLFSGNFLKELS 3648
anole lizard     GLKYIISQQQLLQFAKEISMRANTENWTKDTLQNTVDVLLHHIFQERTDLFSGNFLKELS 3642
fugu             GLKHQVSQQQVLQFAKEVSIKAQTEGWTKEKVQTTADVLLNHIFNERTDLFQGSFLKELS 3649
tetraodon        GLKHQVSQQQVLQFAKEVSIKAQTEGWTKEKVQTAVDVLLSHVFNERTDLFQGSFLKELS 3647
stickleback      GLKHKVSQQQVLQFAKEVSIKAQTESWSQEKVQTIVDVLLNHIFNDRTDLLEGTFLKELS 3649
medaka           GLKHDVTQQQVLQFAKEVSIKAQTEGWSKEKVQNIVEVLLNHIFNDRKDLFQGTFLKELS 3649
zebrafish        GLKYVVSQQQVLQFAKEISIKAHTENWSKEKVQTIVDVLLNHIFNDRTDLFDGTFLKELS 3650
                 ***. ::***:******:*::* **.*:::.:*. .::** *:*::* **:.*.******

                   TS    W  V                A
human            LIPFLCPERAPAEFIRFHPQYQEVNGTLPLIKFNGAQVNPKFKQCDVLQLLWTSCPILPE 3709
orangutan        LIPFLCPERAPAEFIRFHPQYQEVNGTLPLIKFNGAQVNPKFKQCDVLQLLWTSCPILPE 3709
mouse            LIPFLCPERAPAEYIRFHPQYQEVNGTLPLIKFNGAQVNPKFKQCDVLQLLWTSCPILPE 3711
rat              LIPFLCPERAPAEYIRFHPQYQEVNGTLPLIKFNGAQVNPKFKQCDVLQLLWTSCPILPE 3710
dog              LIPFLCPERAPAEFIRFHPQYQEVNGTLPLIKFNGAQVNPKFKQCDVLQLLWTSCPILPE 3707
horse            LIPFLCPERAPAEFIRFHPQYQEVNGTLPLIKFNGAQVNPKFKQCDVLQLLWTSCPILPE 3706
chicken          LIPFLCPERAPAEFVRFHPQYQEVNGTLPLIRFNGAQVNPKFKQTDVLQLLWTSCPILPE 3703
zebra finch      LIPFLCPERAPAEFVRFHPQYQEVNGTLPLIRFNGAQVNPKFKQTDVLQLLWTSCPILPE 3708
anole lizard     SIAFLCPERAPAEIVRFHPQYQEVNGTLPLIKFSGAQVNPKFKQTDVLQLLWTSCPILPE 3702
fugu             MIPFLYPERAPKELIKLHSQYQEMSGSLPLIRFSGSQVNPKFKQTDIIHLLWTSCPILPE 3709
tetraodon        MVQFLYPERAPKELVKLHSQYQEVSGSLPLIRFSGSQVNPKFKQTDAIHLLWTSCPVLPE 3707
stickleback      MIPFLCPERAPNELIKLHSQYQEMSGTIPLIRFSGSQVNPKFKQTDVIHLLWTSCPILPE 3709
medaka           MIPFLCPERAPRELLKLHTQYQEMSSTLPLIRFSGSQVNPKFKQTDIIHLLWTSCPILPE 3709
zebrafish        MISFLCPERAPAELVHLHAQYQEMNDTVPLIRFSGSQVNPKFKQTDIMQLLWTSCPILPE 3710
                  : ** ***** * :::*.****:..::***:*.*:******** * ::*******:***

                                    X
human            KATPLSIKEQEGSDLGPQEQLEQVLNMLNVNLDPPLDKVINNCRNICNITTLDEEMVKTR 3769
orangutan        KATPLSIKEQEGSDLGPQEQLEQVLNMLNVNLDPPLDKVINNCRNICNITTLDEEMVKTR 3769
mouse            KATPLSIKEQEGSDLAPQEQLEQVLNMLNVNLDPPLDKVINNCRNICNITTLDEEMVKTR 3771
rat              KATPLSIKEQEGSSLAPQEQLEQVLNMLNVNLDPPLDKVINNCRNICNITTLDEEMVKTR 3770
dog              KATPLSIREQEGSDLGPQEQLEQVLSMLNVNLDPPLDKVINNCRNICNITTLDEEMVKTR 3767
horse            KATPLSIKEQEGSDLGPQEQLEQVLSMLNVNLDPPLDKVINNCRNICNITTLDEEMVKTR 3766
chicken          KATPLSIKEQEGSTLDMQEQLEQVLTMLSVNLDPPLDKVINNCRNICNITTVDEEMVKTR 3763
zebra finch      KATPLSIKEQEGSSLGAQEQLEQVLAMLNVNLDPPLDKVINNCRNICNITTLDEDMVKTR 3768
anole lizard     KATPLSIKEQEGSSLGPQEQLEHVLTMLSVNLDPPLDKVINNCRNICNITTLDEDMVKTR 3762
fugu             KATPSSIKEQDGTTLTGQEQLDQVLNTLAVTLDPPLEKVISNCKNICNISSPDDEMVKTR 3769
tetraodon        QATPSSIREQDGTSLRGQELLHQVLHLLGVNLDPPLEKVISNCKNICNISSPDDEMVKTR 3767
stickleback      KATPSSIKDQEGNTLTGQEQLDHVLNMLGVNLDPPLEKVLSNCKNICNISNPDDEMVKTR 3769
medaka           KATPSSIKDQEGSTLTGQEQLDQVLSMLGVNLDPPLDKVISNCKNICNIANPDDEMVKTR 3769
zebrafish        RATPASIKDQEGNTLPSQEQLDLILTMLNVNVEPPLEKVISNCKNICSVTNLDDDTVKTR 3770
                 :*** **::*:*. *  ** *. :*  * *.::***:**:.**:***.::. *:: ****

                                       X
human            AKVLRSIYEFLSAEKREFRFQLRGVAFVMVEDGWKLLKPEEVVINLEYESDFKPYLYKLP 3829
orangutan        AKVLRSIYEFLSAEKREFRFQLRGVAFVMVEDGWKLLKPEEVVINLEYESDFKPYLYKLP 3829
mouse            AKVLRSIYEFLSAEKREFRFQLRGVAFVMVEDGWKLLKPEEVVINLEYEADFKPYLYKLP 3831
rat              AKVLRSIYEFLSAEKREFRFQLRGVAFVMVEDGWKLLKPEEVVINLEYEADFKPYLYKLP 3830
dog              AKVLRSIYEFLSAEKREFRFQLRGVAFVMVEDGWKLLKPEEVVINLEYESDFKPYLYKLP 3827
horse            AKVLRSIYEFLSAEKREFRFQLRGVAFVMVEDGWKLLKPEEVVINLEYESDFKPYLYKLP 3826
chicken          AKILRSIYEFLSTEKREFRFQLRGVSFVMVEEGWKLLKPEEVVINLEYESDFKPYLYKLP 3823
zebra finch      AKVLRSIYEFLSTEKREFRFQLRGVSFVMVEEGWKLLKPEEVVINLEYESDFKPYLYKLP 3828
anole lizard     VKVLRSIYEFLSAEKREFRFQLRGVAFVMVEEGWKLLKPEEVVINLEYESDFKPYLYKLP 3822
fugu             NKVLRSTYEFLNGDKRDFRYHLRGVAFVIVEDGWKLLKSEEVVINLDNESDFKPYLYKLP 3829
tetraodon        NKVLRSTYEFLNGDKRDFRYHLRGVAFVIVEDGWKLLKSEEVVINLDNESDFKPYLYKLP 3827
stickleback      NKVLRSTYEFLNADKRDFRYQLRGVAVVMVEDGWKLLKPEEVVINLENESDFKPYLYKLP 3829
medaka           NKVLRSTYEFLTADKRDFSHQLRGIAFVMVEDGWKLLKPEQVVINLDNESDFKPYLYKLP 3829
zebrafish        NKVLRSIYEFLNADKNDFRFQLRGVAFVMVEEGWKLLKPEEVVINLDNESDFKPYLYKLP 3830
                  *:*** ****. :*.:* .:***::.*:**:******.*:*****: *:**********

human            LELGTFHQLFKHLGTEDIISTKQYVEVLSRIFKNSEGKQLDPNEMRTVKRVVSGLFRSLQ 3889
orangutan        LELGTFHQLFKHLGTEDIISTKQYAEVLSRIFKNSEGKQLDPNEMRTVKRVVSGLFRSLQ 3889
mouse            LELGTFHQLFKHLGTEDIISTKQYVEVLSRIFKSSEGKQLDPNEMRTVKRVVSGLFKSLQ 3891
rat              LELGTFHQLFKHLGTEDIISTKQYVEVLSRIFKNSEGKQLDPNEMRTVKRVVSGLFKSLQ 3890
dog              LELGTFHQLFKHLGTEDIISTKQYVEVLSRIFKNSEGKQLDPNEMRTVKRVVSGLFKSLQ 3887
horse            LELGTFHQLFKHLGTEDIISTKQYVEVLSRIFKNSEGKQLDPNEMRTVKRVVSGLFRSLQ 3886
chicken          LELGTFHQLFKHLGTEDVISTKQYVEVLGRIFKNSEGKQLDPNEMRTVKRVVSGLFKSLQ 3883
zebra finch      LELGTFHQLFKHLGTEDIISTKQYVEVLGRIYKSSEGKQLDPNEMRTVKRVVSGLFKSLQ 3888
anole lizard     LELGTFHQLFKHLGTEDVISTKQYVEVLSRIFKNSEGKQLDPNEMRTVKRVVSGLFKSLQ 3882
fugu             LELGTFHQLFKLLGAEDIVSTKQYTEVLWRIYRNSEGKQLDPNEMRTVKRVVSGFFKSLH 3889
tetraodon        LELGTFHQLFKLLGTEDVVSTKQYTEVLWRIYKNSEGKQLDPNEMRTVKRVVSGFFKSLH 3887
stickleback      LELGTFHQLFKLLGTEDVVSTKQYVEVLWRIYKSSDGKQLDPNEMRTVKRVVSGLFKSLQ 3889
medaka           LELGTFHQLFKLLGTEDVVSTKQYVEVLWRIFRNSEGKQLDPNEMRSVKRVVSGLFKSLQ 3889
zebrafish        LELGTFHQLFKLLGTEDIVSTKQYVEVLCRIFRISEGKQLDPNEMRTVKRTVSGLFKSLQ 3890
                 *********** **:**::*****.*** **:: *:**********:***.***:*:**:
```

```
                                 X                       V
human               NDSVKVRSDLENVRDLALYLPSQDGRLVKSSILVFDDAPHYKSRIQGNIGVQMLVDLSQC 3949
orangutan           NDSVKVRSDLENVRDLALYLPSQDGRLVKSSILVFDDAPHYKSRIQGNIGVQMLVDLSQC 3949
mouse               NDSVKVRSDLENARDLALYLPSQDGKLVKSSILVFDDAPHYKSRIQGNIGVQMLVDLSQC 3951
rat                 NDSVKVRSDLENARDLALYLPSQDGRLVKSSILVFDDAPHYKSRIQGNIGVQMLVDLSQC 3950
dog                 NDSVKVRSDLENVRDLALYLPSQDGRLVKSSILVFDDAPHYKSRIQGNIGVQMLVDLSQC 3947
horse               NDSVKVRNDLENVRDLALYLPSQDGRLVKSSILVFDDAPHYKSRIQGNIGVQMLVDLSQC 3946
chicken             NDSVKVRNDLENMRDFALYLPSQDGRLVKSSILVFDDAPHYKSRIQGNIGVQMLVDLSQC 3943
zebra finch         NDSVKVRNDLENMRDFALYLPSQDGRLVKSSILVFDDAPHYKSRIQGNIGVQMLVDLSQC 3948
anole lizard        NDSVKVRSELENVRDLALYLPSQNGRLVKSSILVFDDAPHYKSRIQGNIGVQMLVDLSQC 3942
fugu                NDPVEIRKDLENLRDLTFYLPSHDGKLAKSSSLVFDDAPHYKSRIQGNIGVQMLVDMSQC 3949
tetraodon           NDPVEIRKDLENLRDLTFYLPSHDGKLAKSSSLVFDDAPHYKSRIQGNIGVQMLVDMGQC 3947
stickleback         NDPVAIRNDLESLKDLLFYMPSHDGRLAKSSSLVFDDAPHYKSRIQGNVGVQMLVDMSQC 3949
medaka              NDPVEIRKDLENLRDLIFYLPSHDGRLAKSSGLVFDDAPHYKSRIQGNVGVQMLVDMSQC 3949
zebrafish           NDAVQVRKDLENLRDLIFYLPSHDGRLAKSNTLVFDDAPHYKSRIQGNVGVQMLVDMSQC 3950
                    **.* :*.:**. :*: :*:**::*:*.**. ****************:*******:.**

human               YLGKDHGFHTKLIMLFPQKLRPRLLSSILEEQLDEETPKVCQFGALCSLQGRLQLLLSSE 4009
orangutan           YLGKDHGFHTKLIMLFPQKLRPRLLSSILEEQLDEETPKVCQFGALCSLQGRLQLLLSSE 4009
mouse               YLGKDHGFHTKLIMLFPQKLRPRLLSSILEEQLDEETPKVCQFGALCSLQGRLQLLLSSE 4011
rat                 YLGKDHGFHTKLIMLFPQKLRPRLLSSILEEQLDEETPKVCQFGALCSLQGRLQLLLSSE 4010
dog                 YLGKDHGFHTKLIMLFPQKLRPRLLSSILEEQLDEETPKVCQFGALCSLQGRLQLLLSSE 4007
horse               YLGKDHGFHTKLIMLFPQKLRPRLLSSILEEQLDEEAPKVCQFGALCSLQGRLQLLLSSE 4006
chicken             YLGKDHGFHTKLIMLFPQKLRPRLLSSILEEQLDEESPRTCQFGALCSLQGRLQLLLSSE 4003
zebra finch         YLGKDHGFHTKLIMLFPQKLRPRLLSSILEEQLDEESPKICQFGALCSLQGRLQLLLSSE 4008
anole lizard        YLGKDHGFHTKLIMLFPQKLRPRLLSSILEEQLDEESPKICQFGALCSLQGRLQLLLSSE 4002
fugu                YLGKDHAFHTKLIMLLPQKLRPRLLSSILEEHLDEDSPKMCQFGALCSLQGRLQLLLSSE 4009
tetraodon           YLGKDHAFHTKLIMLLPQKLRPRLLSSILEEHLDEDSPKMCQFGALCSLQGRLQLLLSSE 4007
stickleback         YLGKDHAFHTKLIMLLPQKLRPRLLSSILEEQLDEDSPRMCQFGALCSLQGRLQLLLSSE 4009
medaka              YLGKDHSFHTKLIMLLPQKLRPRLLSSILEEQLDEDAPKMCQFGALCSLQGRLQLLLSSE 4009
zebrafish           YLGKDHAFHTKMIMLLPQKLRPRLLSSILEEQLDEESPKMCQFGALCSLQGRLQLLLSSE 4010
                    ******.****:***:***************:***::*: ********************

                                                                X
human               QFITGLIRIMKHENDNAFLANEEKAIRLCKALREGLKVSCFEKLQTTLRVKGFNPIPHSR 4069
orangutan           QFITGLIRIMKHENDNAFLANEEKAIRLCKALREGLKVSCFEKLQTTLRVKGFNPIPHSR 4069
mouse               QFITGLIRIMKHENDNAFLANEEKAIRLCKALREGLKVSCFEKLQTTLRVKGFNPIPHSR 4071
rat                 QFITGLIRIMKHENDNAFLANEEKAIRLCKALREGLKVSCFEKLQTTLRVKGFNPIPHSR 4070
dog                 QFITGLIRIMKHENDNAFLANEEKAIRLCKALREGLKVSCFEKLQTTLRVKGFNPIPHSR 4067
horse               QFITGLIRIMKHENDNAFLANEEKAIRLCKALREGLKVSCFEKLQTTLRVKGFNPIPHSR 4066
chicken             QFITGLIRIMKHENDNAFLANEEKAIRLCKALREGLKVSCFEKLQTTLRVKGFAPIPHSK 4063
zebra finch         QFITGLIRIMKHENDNAFLANEEKAIRLCKALREGLKVSCFEKLQTTLRVKGFAPIPHSK 4068
anole lizard        QFITGLIRIMKHENDNAFLANEEKAIKLCKTLREGLKVSCFEKLQTTLRVKGFAPIPHSK 4062
fugu                QFITGLMRIMKHENENAYIVNEEKSIRLCKALCEGLKVSCFEKLQTTLRIKGCTPIPNSC 4069
tetraodon           QFITGLIRIMKHENENAYLVNEEKAIRLCKALCEGLKVSCFEKLQTTLRIKGCTPIPHSC 4067
stickleback         QFITGLIRIMKHENDNAYLVNEEKAIRLCKALCEGLKVSCFEKLQTTLRVKGCSPIPHSR 4069
medaka              QFITGLIRIMKHENDNAYLVNEEKAIRLCKALCEGLKVSCFEKLQTTLRVKGCSPIPNSR 4069
zebrafish           QFITGLIRIMKHENENAYMVNEEKAIRLCKALCEGLKVSCFEKLQTTLRVKGCSPIPHSR 4070
                    ******:*******:**::.****:*:***:* ****************:**  ***:*

                        P                                                    S
human               SETFAFLKRFGNAVILLYIQHSDSKDINFLLALAMTLKSATDNLISDTSYLIAMLGCNDI 4129
orangutan           SETFAFLKRFGNAVILLYIQHSDSKDINFLLALAMTLKSATDNLISDTSYLIAMLGCNDI 4129
mouse               SETFAFLKRFGNAVILLYIQHSDSKDINFLLALAMTLKSATDNLISDTSYLIAMLGCNDI 4131
rat                 SETFAFLKRFGNAVILLYIQHSDSKDINFLLALAMTLKSATDNLISDTSYLIAMLGCNDI 4130
dog                 SETFAFLKRFGNAVILLYIQHSDSKDINFLLALAMTLKSATDNLISDTSYLIAMLGCNDI 4127
horse               SETFAFLKRFGNAVILLYIQHSDSKDINFLLALAMTLKSATDNLISDTSYLIAMLGCNDI 4126
chicken             SETFAFLKRYGNAVILLYIQHSDSKDINFLLALAMSLKSATDNLISDTSYLIAMLGCNDI 4123
zebra finch         SETFAFLKRYGNAVILLYIQHSDSKDINFLLALAMTLKSATDNLISDTSYLIAMLGCNDI 4128
anole lizard        SETFAFLKRYGNAVILLYIQHSDSKDINFLLALAMTLKSATDNLISDTSYLIAMLGCNDI 4122
fugu                SETLAFLKRYGTAVIHLYIQHSDSKDINFLLALAMTLKSATDNLISDTSYLIAMLGCNDI 4129
tetraodon           SETPAFLKRYGTAVIHLYIQHSDSKDINFLLALALTLKSATDNLISDTSYLIAMLGCSDI 4127
stickleback         SETLAFLKRYGTAVIHLYIQHSDSKDINFLLALAMTLKSATDNLISDTSYLIAMLGCNDI 4129
medaka              SETLAFLKRYGTSVIHLYIQHSDSRDINFLLALAMTLKSATDNLISDTSYLIAMLGCNDI 4129
zebrafish           SETLAFLKRYGSAVIHLYIQHSDSKDINFLLALAMTLKSATDNLISDTSYLIAMLGCNDI 4130
                    *** *****:*.:** ********:*********::*********************.**

                                 X
human               YRIGEKLDSLGVKYDSSEPSKLELPMPGTPIPAEIHYTLLMDPMNVFYPGEYVGYLVDAE 4189
orangutan           YRIGEKLDSLGVKYDSSEPSKLELPMPGTPIPAEIHYTLLMDPMNVFYPGEYVGYLVDAE 4189
mouse               YRISEKLDSLGVKYDSSEPSKLELPMPGTPIPAEIHYTLLMDPMNVFYPGEYVGYLVDAE 4191
rat                 YRISEKLDSLGVKYDSSEPSKLELPMPGTPIPAEIHYTLLMDPMNVFYPGEYVGYLVDAE 4190
dog                 YRISEKLDSLGVKYDSSEPSKLELPMPGTPIPAEIHYTLLMDPMNVFYPGEYVGYLVDAE 4187
horse               YRISEKLDSLGVKYDSSEPSKLELPLPGTPIPAEIHYTLLMDPMNVFYPGEYVGYLVDAE 4186
chicken             YRISEKLDSLGVKYDSSEPSKLELPTPGTPIPAEIHYTLLMDPMNVFYPGEYVGYLVDAE 4183
zebra finch         YRISEKLDSLGVKYDSSEPSKLELPTPGTPIPAEIHYTLLMDPMNVFYPGEYVGYLVDSE 4188
anole lizard        YRISEKLDSLGVKYDSSEPSKLELPNPGTPIPPEIHYTLLMDPMNVFYPGEYVGYLVDAE 4182
fugu                YRITEKLDNLGVKYDSAEPSKLELPLPGTPIPAEIHHTLLMDPMNVFYPGEYVGYLVDSE 4189
tetraodon           YRITEKLDNLGVKYDSTEPSKLELPLPGTPIPAEIHHTLLMDPMNVFYPGEYVGYLVDSE 4187
stickleback         YRITEKLDNLGVKYDSTEPSKLELPLPGTPIPAEIHHTLLMDPMNVFYPGEYVGYLVDSE 4189
medaka              YRITEKLDNLGVKYDSTEPSKLELPLPGTPIPAEIHHTLLMDPMNVFYPGEYVGYLVDSE 4189
zebrafish           YRITEKLDNLGVKYDTTEPSKLELPLPGTPIPAEIHHLLLMDPMNVFYPGEYVGYLVDSE 4190
                    *** ****.******::******** ******.***: ********************:*
```

```
                                           H
                                           D
human           GGDIYGSYQPTYTYAIIVQEVEREDADNSSFLGKIYQIDIGYSEYKIVSSLDLYKFSRPE 4249
orangutan       GGDIYGSYQPTYTYAIIVQEVEREDADNSSFLGKIYQIDIGYSEYKIVSSLDLYKFSRPE 4249
mouse           GGDIYGSYQPTYTYAIIVQEVEREDADNTSFLGKIYQIDIGYSEYKIVSSLDLYKFSRPD 4251
rat             GGDIYGSYQPTYTYAIIVQEVEREDADNASFLGKIYQIDIGYSEYKIVSSLDLYKFSRPD 4250
dog             GGDIYGSYQPTYTYAIIVQEVEREDADNSSFLGKIYQIDIGYSEYKIVSSLDLYKFSRPE 4247
horse           GGDIYGSYQPTYTYAIIVQEVEREDADNSSFLGKIYQIDIGYSEYKIVSSLDLYKFSRPE 4246
chicken         GGDIYGSYQPTYTYAIIVQEVEREDGESPSFLGKIYQIDIGYSEYKIVSSLDLYRFSRPE 4243
zebra finch     GGDIYGSYQPTYTYAIIVQEVEREDDESPSFLGKIYQIDIGYSEYKIVSSLDLYKFSRPE 4248
anole lizard    GSDIYGSYHPTYTYAIIVQEVEREDEENSSFLGKIYQIDIGYSEYKIVSSLDLYKFSRPD 4242
fugu            GGDMYGSYQPTYTYAIIVQEVEREEEENSGFLGKCFQIDIGYSEYKIVSSLDLYKFSRQD 4249
tetraodon       GGDIYGSYQPTYTYAIIVQEVEREEEENSGFLGKSFQIDIGYSEYKVVSSLDLYKFSRQD 4247
stickleback     GGDIYGSYQPTYTYAIIVQEVEREEEENKSFLGKCFQIDIGYSEYKIVSSLDLYKFSRQD 4249
medaka          GGDIYGSYQPTYTYAIIVQEVEREEEENASFLGKCFQIDIGYSEYKIVSSLDLYKFSRQE 4249
zebrafish       GGDILGTYQPTYTYAIIVQEVEREEDDSPSVLGKCFQIDIGYSEYKIVSSLDLYKFSRQD 4250
                *.*: *:*:***************: :. ..*** :*********:*******:*** :

human           ESSQSRDSAPSTPTSPTEFLTPGLRSIPPLFSGRESHKTSS--------KHQSPKKLKVN 4301
orangutan       ESSQSRDSAPSTPTSPTEFLAPGLRSIPPLFSGKESHKTSS--------KHQSPKKLKVN 4301
mouse           ESSQNRDSAPTTPTSPTEFLTPGLRSIPPLFSGKESHKSPST-------KHHSPRKLKVN 4304
rat             ESSQSRDSAPTTPTSPTEFLTPGLRSIPPLFSGKESHKPASS-------KHHSPRKLKVN 4303
dog             ESSQSRDSAPSTPTSPTEFLAPGLRSIPPLFSGRESQKTSSS-------KHHSPKKLKVN 4300
horse           ESSQSRDSAPSTPTSPTEFLAPGLRSVPPLFPGRESHKASSS-------KHPSPKKLKVN 4299
chicken         ESSQGRDSTPSTPTSPTEFPAHGLRTIPPLFTGRDSHKTTS-------SKHHSPKKLKSS 4296
zebra finch     ESSQGRDSTPSTPTSPTEFPAHGPRAIPPIFTGRESHKTMS-------SKHHSPKKIKSN 4301
anole lizard    ESSQSRDSAPSTPTSPTEFPAHGTRTVPPIFPGRESHKSSSPSLPSSSSKHHSPKKIKAN 4302
fugu            ESSHVRDTSAPSTPSSPNIHSSGMRLIPPIFSSKENLRPPSQ--------KQSPNKIKLN 4301
tetraodon       ESSHVRDAGAPTTPSSPNSRSSGMRLVPPLFSSKENLRAPTQ--------KQSPNKIKLN 4299
stickleback     ESNHVRETSAPSTPTSPNSRSSGPRMASAAFSGKENLRPPAQ--------KQSPKKIKLH 4301
medaka          ESTPMRNASTPSTPTSPDSRSSGLHMMPPIFTGKENLRPPLQ--------KQSPRKLKLH 4301
zebrafish       DGTHIRDG-TPTPTSPLEGHSPRARMVPPLFPGKENRRAPPT--------EASPKKVKFN 4301
                :..  *:  ..:..:. :  :   :  .. *..::. :.          . **.*:*

                                             Q
                                             W
                                       X     Q  F  Q     K
human           SLPEILKEVTSVVEQAWKLPESERKKIIRRLYLKWHPDKNPENHDIANEVFKHLQNEINR 4361
orangutan       SLPEILKEVTSVVEQAWKLPESERKKIIRRLYLKWHPDKNPENHDIANEVFKHLQNEINR 4361
mouse           ALPEILKEVTSVVEQAWKLPESERKKIIRRLYLKWHPDKNPENHDIANEVFKHLQNEINR 4364
rat             ALPEILKEVTSVVEQAWKLPESERKKIIRRLYLKWHPDKNPENHDIANEVFKHLQNEINR 4363
dog             SLPEILKEVTSVVEQAWKLPESERKKIIRRLYLKWHPDKNPENHDIANEVFKHLQNEINR 4360
horse           SLPDILKEVTSVVEQAWKLPESERKKIIRRLYLKWHPDKNPENHDIANEVFKHLQNEINR 4359
chicken         SLPEILREVTSVIEQAWKLPESERKKIIRRLYLKWHPDKNADNLDIANEVFKHLQNEINR 4356
zebra finch     SLPEILKEVTSVIEQAWKLPESERKKIIRRLYLKWHPDKNAENLDIANEVFKHLQNEINR 4361
anole lizard    SLIEILREVTSVLEQAWKLPESERKKIIRRLYLKWHPDKNAENLDLANEVFKHLQNEINR 4362
fugu            ALPDILKEVTSVVEQAWKRPESERKKIIRRLYLKWHPDKNADNLDIATEVFKHLQNEICR 4361
tetraodon       ALPEILKEVTSVVEQAWKRPETERKKIIRRLYLKWHPDKKGDNLDVATEVFKHLQNEISR 4359
stickleback     TLPEILKEVTLVVEQAWKLSETERKKIIRRLYLKWHPDKNAENLDIATEVFKHLQNEINR 4361
medaka          ALPEILKEVTLVIEQAWKLPETERKKIIRRLYLKWHPDKNAENLDIATEVFKHLQKEISR 4361
zebrafish       ALPEILKEVSSVVEQAWKLSETERKKIIRRLYLKWHPDKNAENLDIATEVFKHLQNEINR 4361
                :*  :**:**: *:***** .*:****************: :* *:*.*******:** *

                                 X                                   X
human           LEKQAFLDQN-ADRASRRTFSTSAS-RFQSDKYSFQRFYTSWNQEATSHKSERQQQNKEK 4419
orangutan       LEKQAFLDQN-ADRASRRTFSSSAS-RFQSDKYSFQRFYTSWNQEATSHKSERQQQNKEK 4419
mouse           LEKQAFLDQN-ADRASRRTFSTSAS-RFQSDKYSFQRFYTSWNQEATSHKSERQQQSKEK 4422
rat             LEKQAFLDQN-ADRASRRTFSTSAS-RFQSDKYSFQRFYTSWNQEATSHKSERQQQSKEK 4421
dog             LEKQAFLDQN-ADRASRRTFSTSAS-RFQSDKYSFQRFYTSWNQEATSHKSERQQQNKEK 4418
horse           LEKQAFLDQN-ADRASRRTFSTSAS-RFQSDKFSFQRFYTSWNQEATSHKSERQQQNKEK 4417
chicken         LEKQAFMDQN-TDRASRRTFSSSAS-RFQSDKFSFQRFYTSWNQEATSHKSERQQ-YKEK 4413
zebra finch     MEKQAFMDQN-MDRASRRPFSSSAS-RFQSDKFSFQRFYTSWNQEATSHKSERQQ-YKEK 4418
anole lizard    LEKQSFIDQN-VDRASRRTFSTSSS-RFQSDKFSFQRFYTSWNQEATSHKSERQQ-SKEK 4419
fugu            MEKQSLSDLQNTDRSTRRPYSTSSS-RFQSEKSSFQRFYSSWNQEATTHKTERQH-FKEH 4419
tetraodon       MEKQSLSDLQNTDRSSRRPYSTSSSSRFQSEKFSFQRFYSSWNQEATTHKTERQQ-FREQ 4418
stickleback     MEKQTLADQQNTERASRRSFSTSSS-RFQSEKFSYQRFYSSWNQEATNHKSERQQ-FREH 4419
medaka          MEKQSLSDQQNADRSSRRSFSTSST-RFQSEKSSFQRFYSSWNQEATSHKSEKQQ-FREH 4419
zebrafish       MEKQTQAEQT-TDRASRRPFSSSST-RFQSEKFSFQRFYTSWNQEATSHKSEKQQ-FREQ 4418
                :***:  :    :*::**.:*:*:: ****:* *:****:*******.**:*:*:  :*:

                                                                 P
human           CPPSAGQTYSQRFFVPPTFKSVGNPVEARRWLRQARANFSAARNDLHKNANEWVCFKCYL 4479
orangutan       CPPSAGQTYSQRFFVPPTFKSVGNPVEARRWLRQARANFSAARNDLHKNANEWVCFKCYL 4479
mouse           CPPSAGQTYSQRFFVPPTFKSVGNPVEARRWLRQARANFSAARNDLHKNANEWVCFKCYL 4482
rat             CPPSAGQTYSQRFFVPPTFKSVGNPVEARRWLRQARANFSAARNDLHKNANEWVCFKCYL 4481
dog             STPAAGQTYSQRFFVPPTFKSVGNPVEARRWLRQARANFSAARNDLHKNANEWVCFKCHL 4478
horse           CPPSAGQTYSQRFFVPPTFKSAGNPVEARRWLRQARANFSAARNDLHKNANEWVCFKCFL 4477
chicken         CPSSTGPSFSQRFFVPPTFRSVGNPVEARRWLRQARANFSAARNDLHKNANEWVCFKCYL 4473
zebra finch     CPSSTGPSYSQRFFVPPTFRSVGNPVEARRWLRQARANFSAARNDLHKNANEWVCFKCYL 4478
anole lizard    CPSSTGPSYSQRFFVPPTFKSVGNPVEARRWLRQARSNFSAARNDLHKNANEWVCFKCYL 4479
fugu            HTSYARSSHSPRFFVPPTFKTVGNPVEARRWLRQARANYSAARNDLHKNANEWVCFKCYL 4479
tetraodon       RASYAGSSHSPRFFAPPTFKTVGNPVEARRWLRQARANYSAARNDLHKNANEWVCFKCYL 4478
stickleback     YTSYAGSSHSHRFFVPPTFKTVGNPVEARRWLRQARANYSAARNDLHKNANEWVCFKCYL 4479
medaka          FTGYAGSSHSHRFFVPPTFKTVGNPVEARRWLRQARANYSAARNDLHKNANEWVCFKCYL 4479
zebrafish       YNNYAGTSHSNRFFVPPSFKSVGNPVEARRWLRQARANFSAARNDLHKNANEWVCFKCYL 4478
                    :  :.* ***.**:*::.**************:*:*******************.*
```

```
                                            T
                                            Q
human           STKLALIAADYAVRGKSDKDVKPTALAQKIEEYSQQLEGLTNDVHTLEAYGVDSLKTRYP 4539
orangutan       STKLALIAADYAVRGKSDKDVKPTALAQKIEEYSQQLEGLTNDVHTLEAYGVDSLKTRYP 4539
mouse           STKLALIAADYAVRGKSDKDVKPTALAQKIEEYSQQLEGLTNDVHTLEAYGVDSLKTRYP 4542
rat             STKLALIAADYAVRGKSDKDVKPTALAQKIEEYSQQLEGLTNDVHTLEAYGVDSLKTRYP 4541
dog             STKLALIAADYAVRGKSDKDVKPTALAQKIEEYSQQLEGLTNDVHTLEAYGVDSLKTRYP 4538
horse           STKLALIAADYAVRGKSDKDVKPTALAQKIEEYSQQLEGLTNDVHTLEAYGVDSLKTRYP 4537
chicken         STKLALIAADYAVKGKSDKDVKPAALAQKIEEYSQQLEGLANDVQTLEAYGVDSLKTRYP 4533
zebra finch     SAKLALIAADYAVKGKSDKDIKPAALAQKIEEYSQQLEGLSNDVQTLEAYGVDSLKTRYP 4538
anole lizard    STKLALIAADYAVRGKSDKDVKPAALAQKIEEYSQQLEGLTNDVHTLEAYGVDSLKTRYP 4539
fugu            ATKLALIAADYAVKGKSDKDVKPTALAQKVEEYSTHLIGLSNDVHILEGYGVDGLRTRYP 4539
tetraodon       ATKLALIAADYAVRGKSDKDVKPTALAQKVEEYSTHLIGLSNDVHILEGYGVDSLRTRYP 4538
stickleback     ATKLALIAADYAVRGKSDKDVKPTALAQKVEEYNPQLKGLSKDVQILEGYGVDSLRTRYP 4539
medaka          ATKLALIAADYAVRGKSDKDVKPTALAQKVEEYSSQLTGLARDVQILEGYGVDSLRTRYP 4539
zebrafish       ATKLALIAADYAVRGKSDKDVKPTALAQKVEEYNTQLTGLAKDVHILEGYGVDSLRTRYP 4538
                ::***********:******:**:*****:***. :* **:.**: **.****.*:****

                         D
                         D                       H
human           DLLPFPQIPNDRFTSEVAMRVMECTACIIIKLENFMQQKV 4579
orangutan       DLLPFPQIPNDRFTSEVAMRVMECTACIIIKLENFIQQKV 4579
mouse           DLLPFPQIPNDRFTSEVAMRVMECTACIIIKLENFIQQKV 4582
rat             DLLPFPQIPNDRFTSEVAMRVMECTACIIIKLENFIQQKV 4581
dog             DLLPFPQIPNDRFTSEVAMRVMECTACIIIKLENFIQQKV 4578
horse           DLLPFPQIPNDRFTSEVAMRVMECTACIIIKLENFIQQKV 4577
chicken         DLLPFPQIPNDRFTSEVAMRVMECTACIIIKLENFIQQKV 4573
zebra finch     DLLPFPQIPNDRFTSEVAMRVMECTACIIIKLENFIQQKV 4578
anole lizard    DLLPFPQIPNDRFTSEVAMRVMECTACIIIKLENFIQQKV 4579
fugu            DLLPFPQIPNDRYTSEVAMRVMECTARIIIKLETFVQQKI 4579
tetraodon       DLLPFPQIPNDRYTSEVAVRVMECTARIIIKLETFVQQKV 4578
stickleback     DLLPFPQIPNDRYTSEVAMRVMECTARIIIKLETFVQQKI 4579
medaka          DLLPFPQIPNDRYTSEVAMRVMECTARIIIKLETFVQHKM 4579
zebrafish       DLLPFPQIPNDRYTSEVAMRVMECTARIIIKLEAFVQQKI 4578
                ************:*****:******* ****** *:*:*:
```

Supp. Figure S2. Amino acid sequence alignment of mammalian (human, orangutan, rat, mouse, dog, horse), bird (chicken, zebra finch), reptilian (anole lizard) and fish (fugu, tetraodon, stickleback, medaka and zebrafish) sacsin proteins. Multiple sequence alignment was generated using ClustalW2 (CLUSTAL 2.0.12) at http://www.ebi.ac.uk/Tools/clustalw2/index.html using default parameters. Amino acids from coding exons (exons 2 to 10 if one refers to human *SACS*) are alternately indicated by black and blue letters. Residue overlap splice sites are indicated by red letters. This multiple alignment has been used to generate the phylogenetic tree reported in Fig. 2A. In addition, in this figure amino acid changes due to mutations in the human *SACS* gene are represented: a) amino acid changes due to missense mutations that are known to be non-pathogenic are highlighted in green (see Supp. Table S3 for reference); b) amino acid changes due to missense mutations not clearly associated to disease are highlighted in yellow (see Supp. Table S3 for reference); c) amino acid changes due to missense or nonsense pathogenic mutations are highlighted in red and purple, respectively (see Table 1 for reference); d) amino acid changes obtained by molecular biology approaches are highlighted in black (see Table 1 for reference).

Essential notes on Methods used and Results shown in Supp. Figure S2

Comparative analysis of vertebrate sacsin genes and deduced mRNA and protein sequences. The VISTA family of tools provides investigators with a unified framework for the alignment of long genomic sequences and whole-genome assemblies, interactive visual analysis of alignments along with functional annotation, and many other comparative genomics capabilities [Ratnere and Dubchak, 2009]. The VISTA Precomputed Alignments tool (*VISTA Browser*) (http://genome.lbl.gov/vista/index.shtml) was used to examine the genomic sequences corresponding to the orangutan (*Pongo pygmaeus abelii*), dog (*Canis lupus familiaris*), horse (*Equus caballus*), mouse (*Mus musculus*), rat (*Rattus norvegicus*) and chicken (*Gallus gallus*) sacsin genes with the reference genomic sequence corresponding to the fully annotated human *SACS* gene [Reference (Base) genome: Human Feb. 2009, chr13:23,902,965-24,007,841; size 104,877 bp].

Information on the nucleotide sequence corresponding to the orangutan *SACS* gene was obtained by direct alignment of the segment of the human genome containing the reference gene and the whole-genome Orangutan Jul. 2007 assembly using the PROLAGAN alignment program. Orangutan *SACS* covered a region on chromosome 13 (Orangutan Jul. 2007, chr13:22,441,062-22,555,954; size 114,893).

Information on the nucleotide sequence corresponding to the mouse *Sacs* gene was obtained by direct alignment of the reference gene and the whole-genome Mouse Jul. 2007 assembly using PROLAGAN. Mouse *Sacs* covered a region on chromosome 14 (Mouse Jul. 2007, chr14:61,757,311-61,834,338; size 77,028).

The nucleotide sequence corresponding to the rat *Sacs* gene was obtained by direct alignment of the reference gene and the whole-genome Rat Nov. 2004 assembly using PROLAGAN. Rat *Sacs* covered a region on chromosome 15 (Rat Nov. 2004, chr15:40,259,278-40,344,609; size 85,332).
The sequence corresponding to the dog *SACS* gene was obtained by direct alignment of the reference gene and the whole-genome Dog May 2005 assembly using the SLAGAN alignment program. Dog *SACS* covered a region on chromosome 25 (Dog May 2005, chr25:18,194,103-18,283,594; size 89,492).
The sequence corresponding to the horse *SACS* gene was obtained by direct alignment of the reference gene and the whole-genome Horse Jan. 2007 assembly using PROLAGAN. Horse *SACS* covered a region on chromosome 17 (Horse Jan. 2007, chr17:4,058,612-4,141,492; size 82,881).
The sequence corresponding to the chicken *SACS* gene was obtained by direct alignment of the reference gene and the whole-genome Chicken May 2006 assembly using PROLAGAN and SLAGAN. Chicken *SACS* covered a region on chromosome 1 between position 181,913,899 and position 181,946,870 (Chicken May 2006, chr1:181,913,899-181,946,870; size 32,972). In this region, the nucleotide sequence of the chicken *SACS* gene included the 2nd to the 9th coding exons. Upstream this region, chicken chromosome 1 lacked of aligned contigs. Thus, the nucleotide sequence corresponding to the first coding exon of the chicken *SACS* gene could not be identified by direct alignment. The first coding exon was not identified even after chicken Expressed Sequence Tag (EST) and mRNA data bank consulting.
The nucleotide sequence corresponding to the zebra finch (*Taeniopygia guttata*) *SACS* gene (*Genome Browser Gataway* at UCSC Genome Bioinformatics) (http://genome.ucsc.edu/) was initially defined by direct alignment of a large region of zebra finch chromosome 1 (whole-genome Zebra finch Jul. 2008 assembly) between position 47,455,299 and position 47,707,701 (Zebra finch Jul. 2008, chr1:47,455,299-47,707,701; size 252,403 bp) and the human genome using the AVID alignment program. This syntenic region included the *SACS* gene, the *SGCG* (γ-sarcoglycan) gene (upstream *SACS*) and the *TNFRSF19* (tumor necrosis factor receptor superfamily, member 19) gene (downstream *SACS*). In this genomic area, the nucleotide sequence of the zebra finch *SACS* gene containing from the 2nd to the 9th coding exons was detected by simple alignment, in the region between position 47,534,232 and position 47,567,206 (Zebra finch Jul. 2008, chr1:47,534,232-47,567,206; size 32,975 bp). Then, the nucleotide sequence containing the putative first coding exon was identified by prediction analysis using *GENSCAN* (http://genes.mit.edu/GENSCAN.html) in the region between position 47,567,207 and 47,634,577, with the putative ATG starting at position 47,573,973.
The nucleotide sequence corresponding to the anole lizard (*Anolis carolinensis*) *SACS* gene (*Genome Browser Gataway* at UCSC Genome Bioinformatics) was initially defined by direct alignment of a large region of anole lizard scaffold 38 (whole-genome Lizard Feb. 2007 assembly) between position 4,653,980 and position 4,902,544 (Lizard Feb. 2007, scaffold_38:4,653,980-4,902,544; size 248,565 bp) and the human genome using AVID. The syntenic region included the *SACS* gene, with the *SGCG* gene upstream and the *TNFRSF19* gene downstream *SACS*. Within this genomic area, the nucleotide sequence of the anole lizard *SACS* gene containing from the 2nd to the 9th coding exons was detected by simple alignment, in a region between position 4,722,981 and position 4,752,056 (Lizard Feb. 2007, scaffold_38:4,722,981-4,752,056; size 29,076 bp). Upstream this region, the first coding exon could not be identified by either *GENSCAN* analysis or anole lizard EST and mRNA data bank consulting.
The nucleotide sequence corresponding to the fugu (*Takifugu rubripes*) *sacs* gene was initially defined by direct alignment of a large region of fugu scaffold 165 (whole-genome Fugu Oct. 2004 assembly) between position 225,316 and position 262,280 (Fugu Oct. 2004, scaffold_165:225,316-262,280), which is located on the single virtual unordered chromosome Un (Fugu Oct. 2004, chrUn:227,141,621-227,178,585; size 36,965 bp), and the human genome using SLAGAN in the VISTA Precomputed Alignments tool. This region kept maintaining the syntenic organization observed in mammals, birds and reptiles, including *sacs*, *sgcg* (upstream) and *tnfrsf19* (downstream *sacs*). Within this region, the nucleotide sequence of the fugu *sacs* gene covering from the 1st to the 9th coding exons was detected by simple alignment, in a region on scaffold 165 between position 229,636 and position 252,320 (Fugu Oct. 2004, scaffold_165:229,636-252,320), which corresponds to position 227,145,941 and 227,168,625 on chromosome Un (Fugu Oct. 2004, chrUn:227,145,941-227,168,625; size 22,685 bp). The putative ATG started at position 227,165,489, as assessed by *GENSCAN*, *GenomeScan* (http://exon.mit.edu/genomescan.html) and by direct comparative analysis to the other fish *sacs* genes examined in this study (see below) using the MLAGAN alignment program. However, based on this analysis, alternative starting codons could not be excluded.
The nucleotide sequence corresponding to the tetraodon (*Tetraodon nigroviridis*) *sacs* gene was defined by direct alignment of the segment of the fugu genome containing the *sacs* (reference) gene and the whole-genome Tetraodon Mar. 2007 assembly using the SLAGAN alignment program. Tetraodon *sacs* covered a region of the virtual chromosome Un_random between position 13,532,193 and position 13,553,333 (Tetraodon Mar. 2007, chrUn_random:13,532,193-13,553,333; size 21,141 bp). Again, *sacs* was flanked by *sgcg* (upstream) and *tnfrsf19* (downstream). Within this region, the coding exons from the 1st to the 9th were detected, with the putative ATG starting at position 13,535,645, as assessed by *GENSCAN*, *GenomeScan* and by direct comparative analysis to the other fish *sacs* genes using MLAGAN. Also in this case, the presence of alternative starting codons could not be excluded.
The nucleotide sequence corresponding to the stickleback (*Gasterosteus aculeatus*) *sacs* gene was defined by direct conversion of the segment of the fugu genome containing the *sacs* gene into the stickleback *sacs* genomic sequence in the whole-genome Stickleback Feb. 2006 assembly using the “Convert” new assembly tool. Stickleback *sacs* covered a region of chromosome I between position 4,138,790 and position 4,160,955 (Stickleback Feb. 2006, chrI:4,138,790-4,160,955; size 22,166 bp). Within this region, the coding exons from the 1st to the 9th were detected, with the putative

ATG starting at position 4,159,754, as assessed by *GENSCAN*, *GenomeScan* and by comparative analysis to the other fish *sacs* genes using MLAGAN. Again, alternative starting codons could not be excluded.
The nucleotide sequence corresponding to the medaka (*Oryzias latipes*) *sacs* gene was also defined by direct conversion of the segment of the fugu genome containing the *sacs* gene into the medaka *sacs* genomic sequence in the whole-genome Medaka Oct. 2005 assembly using the "Convert" tool. Medaka *sacs* covered a region of chromosome 13 between position 25,020,228 and position 25,052,438 (Medaka Oct. 2005, chr13:25,020,228-25,052,438; size 32,211 bp). Within this region, the coding exons from the 1st to the 9th were detected, with the putative ATG starting at position 25,022,384, as assessed by *GENSCAN*, *GenomeScan* and by direct comparative analysis to the other fish *sacs* genes using MLAGAN. Alternative starting codons could not be excluded.
The nucleotide sequence corresponding to the zebrafish (*Danio rerio*) *sacs* gene was initially defined by direct conversion of the segment of the fugu genome containing the *sacs* gene into the zebrafish *sacs* genomic sequence in the whole-genome Zebrafish Mar. 2006 assembly using the "Convert" tool. Zebrafish *sacs* covered a region of chromosome 15 between position 20,067,250 and position 20,109,523 (Zebrafish Oct. 2005, chr15:20,067,250-20,109,523; size 42,274 bp). Within this region, only the coding exons from the 2nd to the 9th could be detected. Conversion was also performed in the whole-genome Zebrafish Jun. 2007. In this assembly, zebrafish *sacs* covered a region of chromosome 15 between position 10,264,995 and position 10,293,271 (Zebrafish Oct. 2005, chr15:10,264,995-10,293,271; size 28,277 bp). In this region, only the coding exons from the 3rd to the 9th could be detected. Therefore, it was obvious that the region of zebrafish chromosome 15 including the *sacs* gene is not well defined in such assemblies and that such approach is not suitable of our purposes. Thus, the nucleotide sequence corresponding to the zebrafish *sacs* gene was searched back on genomic clones by using BLASTN search and the human SACS protein as query sequence and found on the zebrafish genomic clone CH73-346G24 in linkage group 15 (GenBank acc. no. FP016239.4). On clone CH73-346G24, zebrafish *sacs* coding exons from the 1st to the 9th encompassed a region between position 35,232 (the position of the last coding codon) and position 83,318 (the position of the putative starting codon), as assessed by *GENSCAN*, *GenomeScan* and by direct comparative analysis to the other fish *sacs* genes using MLAGAN. Alternative starting codons could not be excluded. Clone CH73-346G24 was used as reference sequence for primer annotation (see Supp. Fig. S1). Details on zebrafish *sacs* gene organization are reported in Supp. Fig. S1A and Supp. Table S1. Based on the genomic analysis detailed above, conceptual translation of human, orangutan, dog, horse, mouse, rat, chicken, zebra finch, anole lizard, fugu, tetraodon, stickleback, medaka and zebrafish coding regions was directly obtained and the deduced protein sequences used for further analyses.

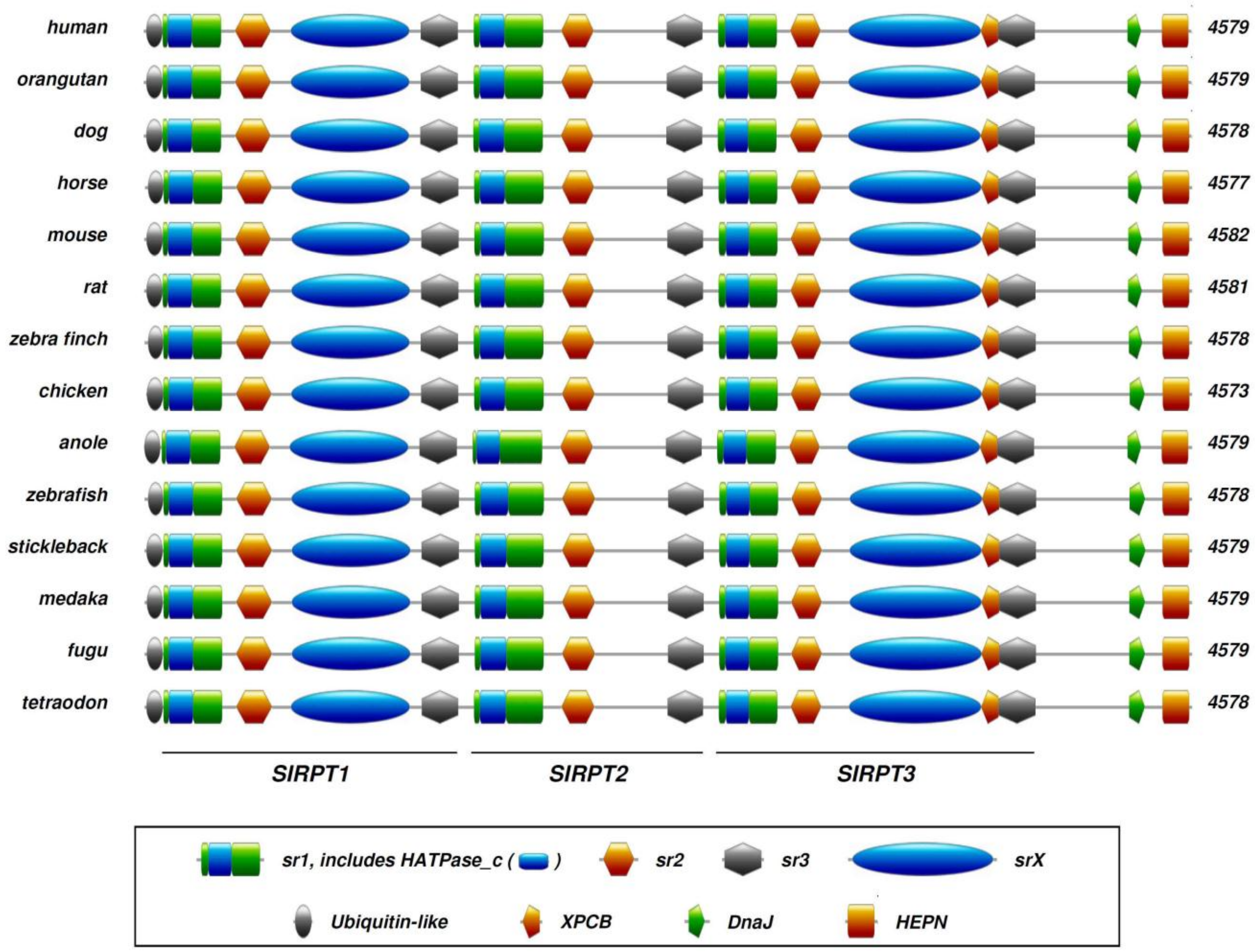

Supp. Figure S3. Conservation of sacsin architecture along the vertebrates scale. The putative fully conserved domains of human, orangutan, dog, horse, mouse, rat, chicken, zebra finch, anole lizard, zebrafish, stickleback, medaka, fugu and tetraodon sacsins are drawn. Repeated domains in the *Sacsin Internal RePeaTs* (*SIRPTs*) detected by using the *Prospero*/*SIM*/*LALNVIEW*-based strategy: *sr1*, sub-repeats 1; *sr2*, sub-repeat 2; *sr3*, sub-repeat 3; *srX*, sub-repeat X. Putative domains detected by using *SMART 6* and/or *ScanProsite*: Ubiquitin-like, HATPase_c, DnaJ, HEPN. For sake of clarity, the putative sacsin XPCB domain is also shown. Domains were drawn using the *MyDomains* image creator. Please note that the chicken and anole sacsins are depicted as lacking the extreme N-terminus because their respective exons one could not be identified in the databases.

```
human SIRPT1-sr1           ---LKGGGRFGQTTPPLVDFLKDILRRYPEGGQILKELIQNAEDAGATEVKFLYDETQYG--TETLWSKDMAPYQGPALYVYNNAVFTPEDWHGIQEIARSRKKDDPLKVGRFGIGFNSVYHITDVPCIFSG-DQIGMLDPHQTLFGPHE-----  226
orangutan SIRPT1-sr1       ---LKGGGRFGQTTPPLVDFLKDILRRYPEGGQILKELIQNAEDAGATEVKFLYDETQYG--TETLWSKDMAPYQGPALYVYNNAVFTPEDWHGIQEIARSRKKDDPLKVGRFGIGFNSVYHITDVPCIFSG-DQIGMLDPHQTLFGPHE-----  226
dog SIRPT-sr1              ---LKGGGRFGQTTPPLVDFLKDILRRYPEGGQILKELIQNAEDAGATEVKFLYDETQYG--TETLWSKDMAQYQGPALYVYNNAVFTPEDWHGIQEIARSRKKDDPLKVGRFGIGFNSVYHITDVPCIFSG-DQIGMLDPHQTLFGPHE-----  226
horse SIRPT1-sr1           ---LKGGGRFGQTTPPLVDFLKDILRRYPEGGQILKELIQNAEDAGATEVKFLYDETQYG--TETLWSKDMAQYQGSALYVYNNAVFTPEDWHGIQEIARSRKKDDPLKVGRFGIGFNSVYHITDVPCIFSG-DQIGMLDPHQTLFGPHE-----  226
mouse SIRPT1-sr1           ---LKGGGRFGQTTPPLVDFLKDILRRYPEGGQILKELIQNAEDAGATEVKFLYDETQYG--TETLWSKDMAQYQGSALYVYNNAVFTPEDWHGIQEIARSRKKDDPLKVGRFGIGFNSVYHITDVPCIFSG-DQIGMLDPHQTLFGPHE-----  226
rat SIRPT1-sr1             ---LKGGGRFGQTTPPLVDFLKDILRRYPEGGQILKELIQNAEDAGATEVKFLYDETQYG--TETLWSKDMAQYQGSALYVYNNAVFTPEDWHGIQEIARSRKKDDPLKVGRFGIGFNSVYHITDVPCIFSG-DQIGMLDPHQTLFGPHE-----  225
zebrafinch SIRPT1-sr1      ---LKGGGRFGQTTPPLVDFLKDILRRYPEGGQILKELIQNAEDAGATEVRFLYDETQYG--TETLWSKDMAQYQGPAFYAYNDAVFTPEDWHGIQEIARSRKKDDPLKVGRFGIGFNSVYHITDVPSIFSG-DQIGMLDPHQTLFGPHE-----  226
chicken SIRPT1-sr1         ---LKGGGRFGQTTPPLVDFLKDILRRYPEGGQILKELIQNAEDAGATEVRFLYDETQYG--TETLWSKDMAQYQGPAFYAYNDAIFTPEDWHGIQEIARSRKKDDPLKVGRFGIGFNSVYHITDVPSIFSG-DQIGMLDPHQTLFGPHE-----  220
anole SIRPT1-sr1           ---LKGGGRFGQTTPPLVDFLKDILRRYPEGGQILKELIQNAEDAGATEVRFLYDETQYG--NESLWSKDMAQYQGSALYVYNNAIFTPEDWHGIQEIARSRKKDDPLKVGRFGIGFNSVYHITDVPSIFSG-DQIGMLDPHQTLFGPHE-----  220
fugu SIRPT1-sr1            ---LKGGGRFGQTTPPLVEFLKDILRRYPEGGQILKELIQNAEDAGATEVKFLFDETEYG--VESLWSPDMEQHQGTALYAYNDAVFTVEDWNGIQEIARSRKRDDPLKVGRFGIGFNSVYHVTDVPSIFSG-DQIAMLDPHQTLFGVHE-----  228
tetraodon SIRPT1-sr1       ---LRGGGRFGQTTPPLVEFLKDILRRYPEGGQILKELIQNAEDAGATEVKFMYDETEHG--VESLWSPDLEQHQGTALYAYNDAVFTLEDWSGIQEIARSRKRDDPLKVGRFGIGFNSVYHVTDVPSIFSG-DQIAMLDPHQTLFGPHE-----  228
stickleback SIRPT1-sr1     ---LKGGGRFGQTTPPLVDFLKDILRRYPEGGQILKELIQNAEDAGATEVKFMYDETEYG--VESLWSPDMAQYQGTALYVYNDAVFTEEDWNGIQEIARSRKREDPLKVGRFGIGFNSVYHITDAPSIFSG-DQIAMLDPHQTLFGVHE-----  228
medaka SIRPT1-sr1          ---LKGGGRFGQTTPPLVEFLKDILRRYPEGGQILKELIQNAEDAGATEVRFMYDETEYG--VESLWSPDMAQHQGAAMYVYNNAVFTGEDWNGIQEIARSRKRDDPLKVGRFGIGFNSVYHITDVPSIFSG-NQIAMLDPHQTLFGVNE-----  228
zebrafish SIRPT1-sr1       ---PRGGGRFGQTTPPLVDFLKDILRRYPEGGQILKELIQNAEDAGATEVKFLYDETEYG--VESLWSHDMAQYQGTALYVYNDAVFTTEDWNGIQEIARSRKREDPLKVGRFGIGFNSVYHITDVPSIFSG-DQIAMLDPHQMLFGVHE-----  228
human SIRPT2-sr1           INPENMGFEQSGQREPLTVRIKNILEEYPSVSDIFKELLQNADDANATECSFLIDMRRNMDIRENLLDPGMAACHGPALWSFNNSQFSDSDFVNITRLGESLKRGEVDKVGKFGLGFNSVYHITDIPIIMSR-EFMIMFDPNINHISKHIKDKSN 1597
orangutan SIRPT2-sr1       INPENMGFEQSGQREPLTVRIKNILEEYPSVSDIFKELLQNADDANATECSFLIDMRRNMDIRENLLDPGMAACHGPALWSFNNSQFSDSDFVNITRLGESLKRGEVDKVGKFGLGFNSVYHITDIPIIMSR-EFMIMFDPNINHISKHIKDKSN 1597
dog SIRPT2-sr1             INPENMGFEQSGQREPLTVRIKNILEEYPSVSDIFKELLQNADDANATECSFMIDMRRNMDIRENLLDPGMAACHGPALWSFNNSQFSDSDFVNITRLGESLKREEVDKVGKFGLGFNSVYHITDIPIIMSR-EFMIMFDPNINHISKHIKDKSN 1595
horse SIRPT2-sr1           INPENMGFEQSGQREPLTVRIKNILEEYPSVSDIFKELLQNADDANATECSFMIDMRRNMDIRENLLDPGMAACHGPALWSFNNSEFSDSDFVNITRLGESLKRAEVDKVGKFGLGFNSVYHITDIPIIMSR-EFMIMFDPNINHISKHIKDKSN 1594
mouse SIRPT2-sr1           INPENMGFEQSGQREPLTVRIKNILEEYPSVSDIFKELLQNADDANATECSFMIDMRRNMDIRENLLDPGMAACHGPALWSFNNSEFSDSDFLNITRLGESLKRGEVDKVGKFGLGFNSVYHITDIPIIMSR-EFMIMFDPNINHISKHIKDRSN 1599
rat SIRPT2-sr1             INPENMGFEQSGQREPLTVRIKNILEEYPSVSDIFKELLQNADDANATECSFMIDMRRNMDIRENLLDPGMAACHGPALWSFNNSEFSDSDFINITRLGESLKRGEVDKVGKFGLGFNSVYHITDIPIIMSR-EFMIMFDPNINHISKHIKDRSN 1598
zebrafinch SIRPT2-sr1      INPENMGFEQSGQREPLTVRIKNILEEYPSISDIFKELLQNADDANATECNFLIDMRRNMDIRENLLDPGMAACHGPALWSFNNSEFSDSDFLNITRLGESLKRSEVDKVGKFGLGFNSVYHITDVPIILSR-EFMIMFDPNVNHISKHIRDKSN 1594
chicken SIRPT2-sr1         INPENMGFEQSGQREPLTVRIKNILEEYPSISDIFKELLQNADDANASECNFLIDMRRNMDIRENLLDPGMAACHGPALWSFNNSEFSDSDFLNITRLGESLKRSEVDKVGKFGLGFNSVYHITDVPIILSR-EFMIMFDPNVNHIGKHIRDKSN 1589
anole SIRPT2-sr1           INPENMGFEQSGQREPLTVRIKNILEEYPSVSDIFKELLQNADDANATDCNFLIDMRRNNDIRENLLDPGMAASHGPALWSFNNSEFSDNDFLNITRLGESLKRAEVDKVGKFGLGFNSVYHITDIPIIMSR-EFMIMFDPNINHLSKHIRDKSN 1589
fugu SIRPT2-sr1            VNPENLGFEQSGQREPLTVRIKNILEEYPSVADIFKELLQNADDASATECNFLIDMRKNNEIRENLLDPGMIVCHGPSLWSFNNAVFSDTDFLNITRLGGSVKRSEADKVGKFGLGFNSVYHITDIPIIMSR-EFMIIFDPNINHIRKHIRDRSN 1598
tetraodon SIRPT2-sr1       VNPENLGFEQSGQREPLTVRIKNILEEYPSVADIFKELLQNADDASATECNFLIDMRKNNDIRENLLDPGMIACHGPSLWSFNNAVFSDTDFLNITRLGGSVKRSEADKVGKFGLGFNSVYHITDIPIIMSR-EFMIIFDPNINHISKHIRDRSN 1595
stickleback SIRPT2-sr1     INPENLGFEQSGQREPLTVRIKNILEEYPSVADIFKELLQNADDASATECSFLIDMRKNTNIRENLLDPGMIVCHGPSLWSFNSSVFSDADFLNITRLGGSVKRCEADKVGKFGLGFNSVYHITDIPIIMSR-EFMIMFDPNINHISKHIRDSSN 1598
medaka SIRPT2-sr1          INPENLGFEQSGQQEPLTVRIKNILEEYPSVADIFKELLQNADDASATECSFLIDMRKNLDIRENLLDPGMIVCHGPSLWSFNNAVFTDTDFLNITRLGGSIKRCEADKVGKFGLGFNSVYHITDIPIIMSR-EFMIMFDPNINHISKHIRDRSN 1598
zebrafish SIRPT2-sr1       INPENLGFEQSGQREPLTVRIKNILEEYPSVADIFKELLQNADDANATECSFLIDMRKNLEIRENLLDPGMIICHGPALWSFNNSVFSDTDFLNITRLGGSMKRCEADKVGKFGLGFNSVYHVTDIPIIMSR-EFMIMFDPNINHIGKHIRDKSN 1598
human SIRPT3-sr1           NVCFTTLGTEFGQKEKLTSRIKSILNAYPSEKEMLKELLQNADDAKATEICFVFDPRQHP--VDRIFDDKWAPLQGPALCVYNNQPFTEDDVRGIQNLGKGTKEGNPYKTGQYGIGFNSVYHITDCPSFISGNDILCIFDPHARYAPGAT----- 2659
orangutan SIRPT3-sr1       NVCFTTLGTEFGQKEKLTSRIKSILNAYPSEKEMLKELLQNADDAKATEICFVFDPRQHP--VDRIFDDKWAPLQGPALCVYNNQPFTEDDVRGIQNLGKGTKEGNPYKTGQYGIGFNSVYHITDCPSFISGNDILCIFDPHARYAPGAT----- 2659
dog SIRPT3-sr1             NICFTTLGTEFGQKEKLTSRIKSILNAYPSEKEMLKELLQNADDAKATEICFVFDPRQHP--VDRIFDDKWAPLQGPALCVYNNQPFTEDDVRGIQNLGKGTKEGNPCKTGQYGIGFNSVYHITDCPSFISGNDILCIFDPHARYAPGAT----- 2657
horse SIRPT3-sr1           NICFTTLGTEFGQKEKLTSRIKSILNAYPSEKEMLKELLQNADDAKATEICFVFDPRQHP--VDRIFDDKWAPLQGPALCVYNNQPFTEDDVRGIQNLGKGTKEGNPCKTGQYGIGFNSVYHITDCPSFISGNDILCIFDPHARYAPGAT----- 2656
mouse SIRPT3-sr1           NICFTALGTEFGQKEKLTSRIKSILNAYPSEKEMLKELLQNADDAKATEICFVFDPRQHP--VDRIFDDKWAPLQGPALCVYNNQPFTEDDVRGIQNLGKGTKEGNPCKTGQYGIGFNSVYHITDCPSFISGNDILCIFDPHARYAPGAT----- 2661
rat SIRPT3-sr1             NICFTAPGTEFGQKEKLTSRIKSILNAYPSEKEMLKELLQNADDAKATEICFVFDPRQHP--VDRIFDDKWAPLQGPALCVYNNQPFTEDDVRGIQNLGKGTKEGNPCKTGQYGIGFNSVYHITDCPSFISGNDILCIFDPHARYAPGAT----- 2660
zebrafinch SIRPT3-sr1      NICFTTLGTEFGQKEKLTSRIKSILNAYPSEKEMLKELLQNADDAKATEICFVFDPRQHP--ADRIFDEKWAPLQGPALCVYNNQPFTEDDIRGIQNLGKGTKVGNPCKTGQYGIGFNSVYHITDCPSFLSGNDILCIFDPHARYAPGST----- 2658
chicken SIRPT3-sr1         NICFTTLGTEFGQKEKLTSRIKSILNAYPSEKEMLKELLQNADDAKATEICFVFDPRQHP--ADRIFDEKWAPLQGPALCVYNNQPFTEDDIRGIQNLGKGTKVGNPCKTGQYGIGFNSVYHITDCPSFISGNDILCIFDPHARYAPGAT----- 2653
anole SIRPT3-sr1           NICFTTLGTEFGQKEKLTGRIKSILNAYPSEKEMLKELLQNADDAKATEICFVFDPRQHS--TDRIFDDKWAPLQGPALCVYNNQPFTEDDIRGIQNLGKGTKEGNPCKTGQYGIGFNSVYHLTDCPSFVSGNDILCIFDPHARYAPGAT----- 2652
fugu SIRPT3-sr1            NICFTPPGSEFGQKEKLTSRIKSILSAYPSEKEMLKELLQNADDAKATEIFFIYDPRTHP--TDRVFDDKWIPMQGPALCVYNNQPFTEDDVRGIQNLGRGTKEANPGKTGQYGIGFNSVYYITDCPSFISNSDILCIFDPHAQYAPGAT----- 2661
tetraodon SIRPT3-sr1       NICFTPPGSEFGQKETLTSRIKSILSAYPSEKEMLKELLQNADDAKATEIYFIYDPRTHP--TDRVFDDKWIPMQGPALCVYNNQPFTEDDVRGIQNLGRGTKEANPGKTGQYGIGFNSVYHITDCPSFISNNDILCIFDPHAQYAPGAT----- 2658
stickleback SIRPT3-sr1     NVCFTTLGTEFGQKEKLTSRIKSILNAYPSEKEMLKELLQNADDAKATEIYFVFDPRTHP--TDRIFDDKWIPMQGPALCVYNNQPFTEDDVRGIQNLGRGTKEANPGKTGQYGIGFNSVYHITDCPSFISNNDILCIFDPHAQYSPGAT----- 2661
medaka SIRPT3-sr1          NVCFTTLGSEFGQKEKLTSRIKSILKAYPSEKEMLKELLQNADDAKATEIFFVFDSRTHP--TDRIFDDKWVPMQGPALCVYNNQPFTEDDVRGIQNLGRGTKEANPGKTGQYGIGFNSVYHITDCPSFVSNNDILCIFDPHAQYAPGAT----- 2661
zebrafish SIRPT3-sr1       NICFTTLGSEFGQKEKLTSRIKSILNAYPSEKEMLKELLQNADDAKATEIYFVFDPRTHS--TDRIFDDKWTPLQGPSLCVYNNQPFTEDDIRGIQNLGRGTKEANPGKTGQYGIGFNSVYHITDCPSFISNNDILCIFDPHARYAPGAT----- 2661
                                            *.  :*.**  **.  :::***:***:** *::  *: *        : : .       :*.::  :*.  *:  *  .*  .:. . *  :  *.*::*:******::** *  :.*  :  :  ::**:
                                                                                   SV                                R    T     Y                           S     K       P        R   R
```

(continue)

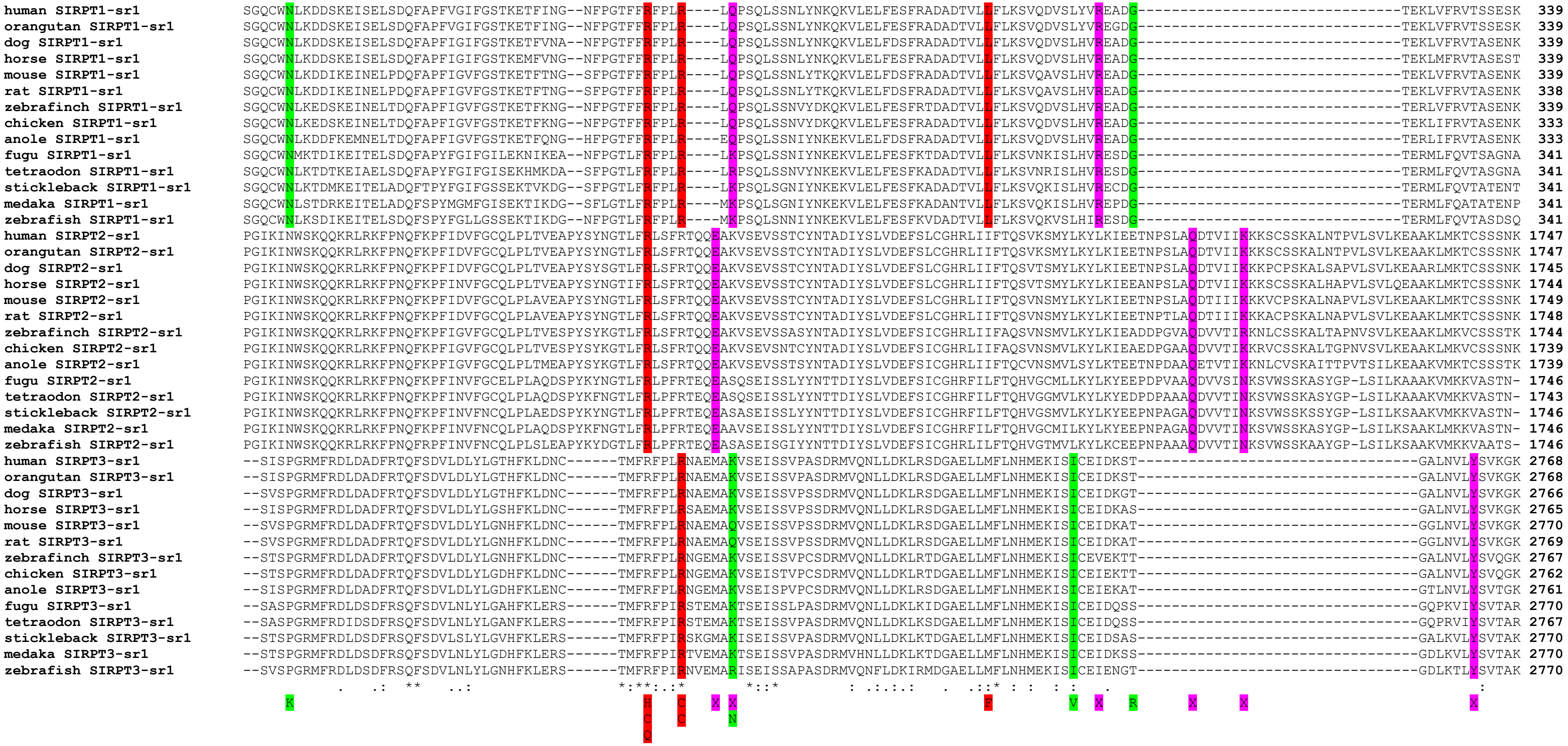

Supp. Figure S4. Multiple alignment of the *SIRPT1-sr1*, *SIRPT2-sr1* and *SIRPT3-sr1* amino acid sequences of vertebrate sacsin proteins. Sequences (human, orangutan, dog, horse, mouse, rat, mouse, zebrafinch, chicken, anole, fugu, tetraodon, stickleback, medaka and zebrafish) were aligned by *ClustalW2* using default parameters. Then missense and nonsense mutations were mapped on the generated alignment. Amino acid changes due to mutations known to be pathogenic are highlighted in red (missense mutations) and purple (nonsense mutations) (see Table 1 for reference). Amino acid changes due to missense mutations not clearly associated with disease are highlighted in yellow (see Supp. Table S3 for reference). Amino acid changes that are known to be non-pathogenic are highlighted in green (Supp. Table S3). (*upper*) Alignment of the N-terminal regions of *SIRPT1-sr1*, *SIRPT2-sr1* and *SIRPT3-sr1* that correspond to HATPase_c domain of sacsins. The following mutations are mapped: p.D168Y, p.T201K, p.W1523R, p.R1575P, p.P1583R, p.H1587R, p.A2558V, p.A162T, p.N1489S, p.N2627S. (*lower*) Alignment of the regions of *SIRPT1-sr1*, *SIRPT2-sr1* and *SIRPT3-sr1* located immediately downstream the HATPase_c domain of sacsins. The following mutations are mapped: p.R272H, p.R272C, p.R276C, p.Q278X, p.L308F, p.R321X, p.R1645Q, p.E1653X, p.Q1709X, p.K1715X, p.R2703C, p.Y2763X, p.N232K, p.G325R, p.K2709N, p.I2749V.

```
human SIRPT1-sr2              -GRGISSKLDSLADELKFVPIIGIAMPLSSRDDEAKGATSD--FSGKAFCFLPLPPGEESSTGLPVHISGFFGLTDNRRSIKWRELDQWRDPAALWNEFLVMNVVPKAYATLILDSIKRLEMEKSSDFPLSVDVIYKLWPEASKVKVHWQPVLEPLFSELL  557
orangutan SIRPT1-sr2          -GRGISGKLDSLADELKFVPIIGIAMPLSSRDDEAKGATSD--FSGKAFCFLPLPPGEESSTGLPVHISGFFGLTDNRRSIKWRELDQWRDPAALWNEFLVMNVVPKAYATLILDSIKRLETEKSSDFPLSVDVIYKLWPEASKVKVHWQPVLEPLFSELL  557
dog SIRPT1-sr2                -GRGISGKLDSLADELKFVPIIGIAMSLS-RDDEEKGATAD--FSGKAFCFLPLPPGEESKTGLPVHISGFFGLTDNRRSIKWRELDQWRDPAALWNEFLVMTVVPKAYATLILDSIKRLETERSSDFPLSVDLIYKLWPDVSKVRVHWQPVLEPLFNELF  556
horse SIRPT1-sr2              -GRGVSSALDSLADELKFVPIIGIAMPLSSRDDEEKGATSD--FSGKAFCFLPLPPGEESKTGLPVHISGFFGLTDNRRSIKWRELDQWRDPAALWNEFLVVNVVPKAYATLILDSIKRLETERSSDFPFSVDVIYRLWPDVNKVRVHWQRVLEPLFNELF  557
mouse SIRPT1-sr2              -GRGISSKLDSLADELKFVPIIGLAMPLSGKDEE-NGAISD--FSGKAFCFLPLPPGEESRTGLPVHISGFFGLTDNRRSIKWRELDQWRDPAALWNEYLIVNVVPKTYATLILDSIKRLETEKSSDFPLSVDTIYKLWPEASKVKAHWHPVLGPLFSELF  556
rat SIRPT1-sr2                -GRGISSKLDSLADELKFVPIIGLAMSLSGRDEE-NGATSD--FSGKAFCFLPLPPGEESRTGLPVHISGFFGLTDNRRSIKWRELDQWRDPAALWNQFLIVNVVPKAYATLILDSIKRLETEKSSDFPLSVDAIYKLWPEASKVKAHWHPVLGPLFSELF  555
zebrafinch SIRPT1-sr2         -GRGMCTELDCLADDLKFVPTIGIAMSLA-ANEEENGAVAD--FSGRAFCFLPLPPGEESKTGLPVHVSGFFGLTDNRRSIKWRELDQWRDPAALWNDLLVVNIVPKAYTTLILEAIKRMETEKNSDFPLSPERIYRLWPDENKIRVPWKPIVVPLFKDLL  556
chicken SIRPT1-sr2            -GRGMCTELDCLADDLKFVPTIGIAMPLS-YNEKDKGAVAD--FSGRAFCFLPLPPGEESKTGLPVHVSGFFGLTDNRRSIKWRELDQWRDPAALWNDLLVVNIVPKAYSTLILEAIKRMETEENSDFPLSAERIYGLWPDENKIRVPWKPIVVPLFKELL  550
anole SIRPT1-sr2              -GRGMCSDLDCLADDLKYIPTIGIAMSLS-SDGEGKGAAAE--FSGRAFCFLPLPPGEESKTGLPVHVSGFFGLTDNRRSIKWRELDQWRDPAALWNELLVTNMVPKAYATLILEAIKRMETDENSDFPLSADRIYRLWPQKNKIKVHWESIIEPLLKELF  550
fugu SIRPT1-sr2               -GRGMCAELDSLADDLKFMPTIGIALPLTVINKDDEGATSG--FSGRAFCFLPLPPGEESETGLPVHVSGFFGLTDNRRSIKWREVDQWRDPAALWNELLLITVIPRAYLMLITEYVQRVQTKKDQDFPLTPTGTYGAWPNPNLVKSRWKPILQPLFHELL  559
tetraodon SIPRT1-sr2          -GRGLCAELDALADDLKFTPTIGIALPLAVTGKDEQGATSG--FSGRAFCFLPLPPGEESQTGLPVHVSGFFGLTDNRRSIKWREVDQWRDPAAVWNQLLLVTVIPRAYLVLITEAIQRVQRRKDQDFPLSPMGTYGAWPNPGQVRSRWKPILQPLFQELL  559
stickleback SIRPT1-sr2        -GRGMCPELDSLADVLKFMPTIGIALPLTVVNKGDTGATSG--FSGRAFCFLPLPPGEESETGLPVHLSGFFGLTDNRRSIKWREVDQWRDPAALWNELLIITVIPWAYFTLITEAIRRVQTQQDQDFPLSPAGTYGAWPDPKRVKSRWKPILQPLFHDLL  559
medaka SIRPT1-sr2             -GRGMCAELDSLADNLKFMPIIGIALPLAANNQEDKGAASG--FSGRAFCFLPLPPGEESETGLPVHVSGFFGLTDNRRSIKWREVDQWRDLAALWNELLIVIVIPRAYFTLIIDAIKRVQTQKDQDFPLSPAGTYRAWPDPKQVKSRWMPILQPLFQELL  559
zebrafish SIRPT1-sr2          -GRGMCGELDSLADDLKFIPTIGIALPLTLID-EDKGATSS--FSGRAFCFLPLPLGEESMTGLPVHVSGFFGLTDNRRSIKWREVDQWRDPAALWNELLIVTVIPRAYFTLIMETIQRIQTKKDQDFPLSPRGIYGAWPDPNRIRPRWKPILEPLLNDLL  558
human SIRPT2-sr2              TGEALKFSLSESGRRLGLVPCGAVGVQLSEIQDQKWTVKP---HIGEVFCYLPLR----IKTGLPVHINGCFAVTSNRKEIWK------TDTKGRWNTTFMRHVIVKAYLQVLSVLRDLATSGELMDYTY-----YAVWPDPDLVHDDFSVICQGFYEDIA 1968
orangutan SIRPT2-sr2          TGEALKFSLSESGRRLGLVPCGAVGVQLSEIQDQKWTVKP---HIGEVFCYLPLR----IKTGLPVHINGCFAVTSNRKEIWK------TDTKGRWNTTFMRHVIVKAYLQALSVLRDLATSGELMDYTY-----YAVWPDPDLVHDDFSVICQGFYEDIA 1968
dog SIRPT2-sr2                TGEALKFSLNENGRRLGLVPCGAVGVLLSEIQDQKWAVKP---HVGEVFCYLPLR----IKTGLPVHINGCFAVTSNRKEIWK------TDTKGRWNTTFMRHVIVKAYLEALSVLRDLATNGELMDYTY-----YSVWPDPELVHDDFSVICQGFYEDIA 1966
horse SIRPT2-sr2              TGEALKFSLSESGRRLGLVPCGAVGVLLSEIQDQKWAVKP---HVGEVFCYLPLR----IKTGLPVHINGCFAVTSNRKEIWK------TDTKGRWNTTFMRHVVVKAYLEALSVLRDLATSGELVDYTY-----YAVWPDPDSVHDDFSVICQGFYEDIA 1965
mouse SIRPT2-sr2              TGEALKFSLNESGRRLGLVPCGAVGVLLHETQEQKWTVKP---HIGEVFCYLPLR----IKTGLPIHINGCFAVTSNRKEIWK------TDTKGRWNTTFMRHVIVKAYLQALSVLRDLAIGGELTDYTY-----YAVWPDPDLVHDDFSVICKGFYEDIA 1970
rat SIRPT2-sr2                TGEALKFSLNESGRRLGLVPCGAVGVLLHETQEQKWTVKP---HIGEVFCYLPLR----IKTGLPIHINGCFAVTSNRKEIWK------TDTKGRWNTTFMRHVIVKAYLQALSVLRDLAISGELIDYTY-----YAVWPDPDVVHDDFSVICQGFYEDIA 1969
zebrafinch SIRPT2-sr2         VGDALKFSLHESGRRLGLVPCGGVGVLLSETQDQKWIVKPNCNNIGEVFCYLPLR----IKTGLPLHINGCFAVTSNRKEIWK------TDTKGRWNTVFMRHVIVKAYIEALCVLRDMAINGELVDYSY-----CAVWPDPDSVHDDFSVICQGFYEDIA 1968
chicken SIRPT2-sr2            TGDALKFSLHESGRRLGLVPCGGVAVLLTETQDQKWIVKPNCNNIGEVFCYLPLR----IKTGLPLHINGCFAVTSNRKEIWK------TDTKGRWNTVFMRHVIVKAYIEALCVLRDMAISGELVDYSY-----CAVWPDPDSVHDDFSVICQGFYEDIA 1963
anole SIRPT2-sr2              TGEALKFSLQESGRRLGLVPCGSVGVILSEKQDGKWTVKPNCSNLGEVFCYLPLR----IKTGLPVHINGSFAVTSNRKEIWK------TDTKGRWNTVFMRHVIVKAYLEALVVLRDMAVCGELEDYCY-----CAVWPDPDLVHDDFSVICQGFYEEIA 1962
fugu SIRPT2-sr2               ANEALKFSLSDSGKRLGLVPCGGVAVLLSEEENHKWTVKMNATPTGEVFCYLPLR----IKTGLPVHINGCFAVTSNRKEIWK------TDTKGQWNSVFMRHVIVQAYLAALAMLRSMAENGELLDYSY-----YAVWPDPSQVHDDFALISQGVYQEIV 1971
tetraodon SIRPT2-sr2          ANEALKFSLSDSGKRLGLVPCGGVAVLLSEEENHKWTVKTNATPSGEVFCYLPLR----IKTGLPVHINGCFAVTSNRKEIWK------TDTKGQWNSVFMRHVIVQAYLAALAMLRSMAENGELLDYTY-----YAAWPDPSQVHDDFALISQGVYQEIA 1968
stickleback SIRPT2-sr2        VTEALKFSLSDSGKRLGLVPCGGVAVLLSEEENRKWTVKTNATPIGEVFCYLPLR----IKTGLPVHINGCFAVTSNRKEIWK------TDTKGQWNSVFMRHVIVQGYLAALTMLRSMAESGELLDYSY-----YAAWPDPSQVHDDFTLVSQGVYQEIA 1971
medaka SIRPT2-sr2             GSEALKFSLGDSGKRLGLVPCGGVAVLLSEEENRKWTVKTNPNPIGEVFCYLPLR----IKTGLPVHINGCFAVTSNRKEIWK------TDTKGQWNSVFMRHVIVQAYLAALSMLRSMAESGELLEYNY-----YGAWPDPGQVHDDFTLVSQGVYQEIS 1971
zebrafish SIRPT2-sr2          VTEALKFSLNDSGKRLGLVPCGGIAVLLTEEDNRKWTVKTSATPIGEVFCYLPLR----IKTGLPVHINGCFAVTSNRKEIWK------TDTKGQWNSIFMRHVIVQAYLAALNMLRSMAESGELLDYSY-----YAAWPDPGVVHDDFNLICQGVYQEIA 1971
human SIRPT3-sr2              ------FSSMEKVSKSVISAHKNQDITLFPRGGVAACITHNYKKPHRAFCFLPLS----LETGLPFHVNGHFALDSARRNLWRD--DNGVGVRSDWNNSLMTALIAPAYVELLIQLKKRYFPGSD--------------PTLSVLQNTPIHVVKDTLKKFL 2960
orangutan SIRPT3-sr2          ------FSSMEKVSKSVISAHKNQDITLFPRGGVAACITHNYKKPHRAFCFLPLS----LETGLPFHVNGHFALDSARRNLWRD--DNGVGVRSDWNNSLMTALIAPAYVELLIQLKKRYFPGSD--------------PTLSVLQNTPIHVVKDTLKKFL 2960
dog SIRPT3-sr2                ------FSSMEKVSKSVISAHKNQDITLFPRGGVAACITHNYKKPHRAFCFLPLS----LETGLPFHVNGHFALDSARRNLWRD--DNGVGVRSDWNNSLMTALIAPAYVELLIQLKKRYFPGSD--------------PTLSVLQNTPIHVVKDTLKKFL 2968
horse SIRPT3-sr2              ------FSSMEKVSKSVISAHKNQDITLFPRGGVAACITHNYKKPHRAFCFLPLS----LETGLPFHVNGHFALDSARRNLWRD--DNGVGVRSDWNNSLMTALIAPAYVELLIQLKKRYFPGSD--------------PTLSVLQNTPIHVVKDTLKKFL 2957
mouse SIRPT3-sr2              ------FSSMEKVSKSVISAHKNQDITLFPRGGVAACITHNYKKPHRAFCFLPLS----LETGLPFHVNGHFALDSARRNLWRD--DNGVGVRSDWNNSLMTALIAPAYVELLIQLKKRYFPGSD--------------PTLSVLQNTPIHVVKDTLKKFL 2962
rat SIRPT3-sr2                ------FSSMDKVSKSVISAHRNQDITLFPRGGVAACITHNYKKPHRAFCFLPLS----LETGLPFHVNGHFALDSARRNLWRD--DNGVGVRSDWNNSLMTALIAPAYVELLIQLKKRYFPGSD--------------PTLSVLQNTPIHVVKDTLKKFL 2961
zebrafinch SIRPT3-sr2         ------FSAMEKVSKSVVSAHKNEDITLFPRGGVAACITHNYKKPHRAFCFLPLS----LETGLPFHVNGHFALDSARRNLWRD--DNGVGVRSDWNSSLMTALIAPAYVELLIQLKKRYFPGTD--------------PTVSVLQNTPIHAVKDTLKKFL 2959
chicken SIRPT3-sr2            ------FSAMEKVSKSVVSAHKNEDITLFPRGGVAACITHNYKKPHRAFCFLPLS----LETGLPFHVNGHFALDSARRNLWRD--DNGVGVRSDWNNSLMTALIAPAYVELLIQLKKRYFPGTD--------------PTVSVLQNTPVHAVKDTLKKFL 2954
anole SIRPT3-sr2              ------FSSIDKVSKSVISAHKNKDITLFPRGGVAACITHNYKKPHRAFCFLPLS----LETGLPFHVNGHFALDSARRNLWRD--DNGVGVRSDWNNSLMTALIAPAYVELLIQLKKRYFQGTD--------------PTVSVLQNTSMHAVKDTLKKFL 2953
fugu SIRPT3-sr2               ------FPNMENVSKGLVSAHKNEDITLFPRGGVAACVSHNYKKPHRAFCFLPLS----LETGLPFHVNGHFALDSARRNLWRD--DNGVGVRSDWNNNLMTLLIAPACVELLIQLKRRYFPGPD--------------PTMTILQGTPLHMVKDTLRKYL 2962
tetraodon SIRPT3-sr2          ------FPNIENVSKALVSAHKNEDITLFPRGGVAACVSHNYKKPHRAFCFLPLS----LETGLPFHVNGHFALDSARRNLWRD--DNGVGVRSDWNTNLMTLLIAPACVELLIQLKRRYFPGPD--------------PTMTILQGTPLHAVKDTLRKYL 2959
stickleback SIRPT3-sr2        ------FPDIENVSKSVISAHKNEDITLFPRGGVAACVSHNYKKPHRAFCFLPLS----LETGLPFHVNGHFALDSARRNLWRD--DNGVGVRSDWNNNLMTSLIAPAIVELLIQLKRRSFQGPD--------------PTITVLQGTPLHVVKDTLRKYL 2962
medaka SIRPT3-sr2             ------FSDMETVSKSIISAHKNDDITLFPRGGVAACLSHNYKKPHRAFCFLPLS----LETGLPFHVNGHFALDSARRNLWRD--DNGVGVRSDWNNTLMTSLIAPAYVELLIQLKRRYFPGPD--------------PTMTMLQGTPLHAVKDTLRKYL 2962
zebrafish SIRPT3-sr2          ------FSDMERVSKSVISAHKNEDITLFPRGGVAACTSHNYKKTHRAFCFLPLS----LETGLPFHVNGHFALDSARRNLWRD--DNGVGVRSDWNNNLMKVLIAPACVELLIQLKRRLFPGPD--------------PTMTIFQGTPIHVVRDILRKYL 2962
                                                 .     : *                  ..**:***       ****.*:.* *.: . *:.:         .  . **  ::  ::       :                          *    .:            .
                                      G                                             X      I               C                XX               M                          RL                  R
                                                                                           K               X
                                                                                           R
                                                                                           I
```

Supp. Figure S5. Multiple alignment of the *SIRPT1-sr2*, *SIRPT2-sr2* and *SIRPT3-sr2* amino acid sequences of vertebrate sacsin proteins. Sequences (human, orangutan, dog, horse, mouse, rat, mouse, zebrafinch, chicken, anole, fugu, tetraodon, stickleback, medaka, zebrafish) were aligned by *ClustalW2* using default parameters. Then missense and nonsense mutations were mapped on the generated alignment. Amino acid changes due to mutations known to be pathogenic are highlighted in red (missense mutations) and purple (nonsense mutations) (see Table 1 for reference). Amino acid changes due to missense mutations not clearly associated with disease are highlighted in yellow (see Supp. Table S3 for reference). Amino acid changes that are known to be non-pathogenic are highlighted in green (Supp. Table S3). The following mutations are mapped: p.T458I, p.R474C, p.W492X, p.P536L, p.R1877X, p.T1880I, p.R1907X, p.W1946R, p.R2893X, p.L508M, p.S2828G, p.K2958R, p.T458K, p.T458R.

```
human SIRPT1-sr3        ALGIFTKPSLSAVLKHFKIVVDWYSSKTFSDEDYYQFQHILLEIYGFMHDHLNEGKDSFRALKFPWVWTGKKFCPLAQAVIKPIHDLDLQPYLHNVPKTMA-KFHQLFKVCGSIEELTSDHISMVIQKIYLKSDQ-DLSEQESKQNLHLMLNIIRWLYSNQIPAS 1374
orangutan SIRPT1-sr3    ALGIFTKPSISAVLKHFKIVVDWYTSKTFSDEDYYQFQHILLEIYGFMHDHLNEGKDSFKALKFPWVWTGKKFCPLAQAVIKPIHDLDLQPYLHNVPKTMA-KFHQLFKVCGSIEELTSDHISMVIQKIYLKSDQ-DLSEQESKQNLHLMLNIIRWLYSNQIPAS 1374
dog SIRPT1-sr3          ALGIFTKPSISAVLKHFKIVVDWYTSKTFSDEDYYQFQHILLEIYGFMHDHLSEGKDAFRALKFPWVWTGKKFCPLAQAVIKPFHDLDLQPYLHSVPKTMA-KFHQLFKVCGSIEELTSDHISMVIQKVYLKSDQ-DLSDQESKQNLHLMLNIIRWLYSNQIPAS 1372
horse SIRPT1-sr3        ALGIVTKPSISAVLKHFKIVVDWYTSKTFSDEDYYQFQHILLEIYGFMHDHLNEGKDAFRALKFPWVWTGKKFCPLSQAVIKPFHDLDLQPYLHSVPKTMA-KFHQLFKVCGSIEELTSDHISMVIQKVYLKSDQ-DLSEQESKQNLHLMLNIIRWLYSNQIPAS 1371
mouse SIRPT1-sr3        ALSIFTKPTINAVLKHFKTVVDWYTSKTFSDEDYYQFQHILLEIYGFMHDHLSEGKDSFKALKFPWVWTGKNFCPLAQAVIKPTHDLDLQPYLYNVPKTMA-KFHQLFKACGSIEELTSDHISMVIQKVYLKSDQ-ELSEEESKQNLHLMLNIMRWLYSNQIPAS 1376
rat SIRPT1-sr3          TLGIFTKPTISAVLKHFKIVVDWYTSKTFSDEDYYQFQHILLEIYGFMHDHLSEGKDSFKALKYPWVWTGKNFCPLAQAVIKPIHDLDLQPYLYNVPKTMA-KFHQLFKACGSIEELTSDHISMVIQKVYLKSDQ-ELSEEESKQNLHLMLNIMRWLYSNQIPAS 1375
zebrafinch SIRPT1-sr3   ALGIPTKPSIKAVLKHFKVVVDWHGSKTFSDEDYYQFQHILLEIYGFMHDHLEEGKEAFKSLKFPWVWTGKTFCSLAQAVIKSVPDLDLQPYLHYVPKTMA-KFHQLFKCCGSIEELTPDHVSMVIQKIYLKSEQ-SLSEQESKQSLHIMLSIIRWLYSNQIPAS 1371
chicken SIRPT1-sr3      ALGISTKPSIKAVLKHFKVVVDWHSSKTFSDEDYYQFQHILLEIYGFMHDHLEEGKEAFKALKFPWVWTGKTFCSLTQAVIKTVPDLDLQPYLHYVPKTMA-KFHQLFKCCGSIEQLTPDHVSMVIQKIYLKSEQ-SLSEQESKQNLHIMLSIIRWLYSNQIPAS 1366
anole SIRPT1-sr3        ALGISIKPSIKSVLKHFKVVVDWHSSKKFSDEDYYQFQHILLEIYGFMHDHLDEGKEAFKTLKFPWVWTGKTFCSLSQAVIKSVHDLDLQPYLHNVPKTMA-KFHQLFKCCGSIEQLTPDHVSMVIQKIYLRSEQ-PLTEDESKQNLHIMLNIIRWLYRCQIPAN 1366
fugu SIRPT1-sr3         ALKLSLEPQADQVVQHLRAVIDWHKSKAFTTEDWYQFQQILFEIYGFMQTHIQDAKEAMKSLPFDWVWTGKTFSSPGRTVLKPLPDLDLQPYLYSLPKTIR-KFNKLFKSCGSIEEVVPSQVLEVINTIQQRCTG-ELTKDDSKHNILLLLNILRWLHNNQIPVD 1375
tetraodon SIRPT1-sr3    ALKLNVEPQVDQVIQHLKAVIEWHKSQAFTTEDWYQFQQILFEMYGFMQTHLQDAKEAMKSLPFDWVWTGKTFSSPDRTVLKPLPDLDLQPYLYSLPKTIR-KFNKLFKSCGSIEEVDPSQVLEVVNTIQQRCTG-EMTKDDSKHNILLLINILRWLYNNQIPVA 1372
stickleback SIRPT1-sr3  ALKLTTEPQVDQVLQQLKAVNDWHKSQAFTTEDWYQFQQILFEIYGFMQAHLEDAREAMKSLPFDWVWTGKTFSSPGHTALKPLPDLDLQPYLYTLPKTIR-KFHKLFKFCGSVEEVVSSHVFEVISTIKQRCDG-EITKEESKHDILLLVNILRWLNNNQIEVD 1375
medaka SIRPT1-sr3       SLKLNIEPQVDQVLQHLKAVNDWHKSQAFTTEDWYQFQQILFEIYSFMQAHLEDAREAMKSLSFDWVWTGKTFSSPSQTVLKPLADLDLQPYLYSLPKTMR-KFQKLFKFCGSVEEVVPNHVFDVIATIRQRCEE-EMTKEESKHDILLLINILRWLYNTQTPVD 1375
zebrafish SIRPT1-sr3    ALKLSLEPQVDQVLQHLKAVNDWHQSQAFTTEDWYQFQQILCEIYEFMQAHLEDAREALKSLSFDWVWTGKTFASPGCTVLKPIPDLDLQPYLYSLPKTIA-KFNKLFKFCGSIEEVVPSHVFEVVKTIRQRCEE-EMTKQESKRNVLLLINILRWLYSSQIPVD 1375
human SIRPT2-sr3        ------KPTVDLVINQLKEVAKSVDDGITLYQENITNACYKYLHEALMQNEITKMSIIDKLKPFSFILVENAYVDSEKVSFHLN--FEAAPYLYQLPNKYKNNFRELFETVGVRQSCTVEDFALVLESIDQERGTKQITEENFQLCRRIISEGIWSLIREKKQEF 2443
orangutan SIRPT2-sr3    ------KPTVDLVINQLKEVAKSVHDGITLYQENITNACYKYLHEALMQNEITKISIIDKLKPFSFILVENAYVDSEKVSFHLN--FEAAPYLYQLPNKYKNNFRELFESVGVRQSFTVEDFALVLESIDQERGTKQITEENFQLCRRIISEGIWSLIREKKQEF 2443
dog SIRPT2-sr3          ------KPTVDLVVNQLKEVAKSVDDGVTLYQENITNACYKYLHDAMMQNEMIKISIIEKLKPFSFILVENAYVDSEKVSFHLN--FEAAPYLYQLPNKYKNNFRELFESVGVRQSFTVEDFALVLESIDQEKGTKQITEENFQLCRRIISEGIWSLIREKKQEF 2441
horse SIRPT2-sr3        ------KPTVDLVVNQLKEVARSVDDGVTLYQENITNACYKYLHDAMMQNEVAKMSIVEKLKLFNFILVENAYVDSERVSFHLN--FEAAPYLYQLPNKYKNNFRELFESVGVRQSFTVEDFALVLESIDQEKGTKQITEENFQLCRRIISEGIWSLIREKKQEF 2440
mouse SIRPT2-sr3        ------KPTVDLVINQLKQVAKSVDDGITLYQENITNACYKYLHEAVLQNEMAKATIIEKLKPFCFILVENVYVESEKVSFHLN--FEAAPYLYQLPNKYKNNFRELFESVGVRQSFTVEDFALVLESIDQERGKKQITEENFQLCRRIISEGIWSLIREKRQEF 2445
rat SIRPT2-sr3          ------KPTVDLVINQLKQVAKSVDDGITLYQENITNACYKYLHEAILQNEMAKATIIEKLKPFCFILVENVYVESEKVSFHLN--FEAAPYLYQLPNKYKNNFRELFESVGVRQSFTVEDFALVLESIDQERGKKQITEENFQLCRRIISEGIWSLIREKRQEF 2444
zebrafinch SIRPT2-sr3   ------KPAVNLVINQLEEVAKSFD-GITLYQENITNACYKYLHEAMLESESTKAMIIEQLTNSSFILVENVYADPSKVSFHLN--FEAAPYLYQLPNKYKNSFRELFESVGVRQAFTVEDFAVVLELINQERGTKQLTEDNFQLCRRIISEGIWGLIREKKQEF 2442
chicken SIRPT2-sr3      ------KPAVNLVINQLEEVAKSFD-GITLYQENITNACYKHLHEAMLENESTKAMIIEQLTNCSFILVENVYVDPTKVSFHLN--FEAAPYLYQLPNKYKNSFRELFESMGVRQSFTVEDFALVLELINQERGTKQLTEENFQLCRRIISEGIWSLIREKKQEF 2437
anole SIRPT2-sr3        ------KPTVIMVINQLKVVAKHFD-GITLYQENITNACYKYLHEALLQNGATKIVIVEELKNYSFILVEDGYVDPTRVSFHLN--FEAAPYLHQLPNKYRNSFRELFESVGVHQAFTVEDFAQVLGSVNQETENKSLTEENFQLCRRIISEGIWSLIREKKQEL 2436
fugu SIRPT2-sr3         ------KPSVELVISQLKKLSQSFDG-ITLYQENITNACYKFLHEEMLQDECAKEQIAEELTAFNSILVENTYVSPAKIAFHLN--FDASPHLYQLPNKYRSSCRELFENVGVKSSFSVDDFSGILETVKQGCGRRALSEDNFQLCRRVISEGIWSLIRDKSQEY 2445
tetraodon SIRPT2-sr3    ------TPSVELVISQLKKLSQSFDG-VTLYQENITNACYKFLHEEMLQDERAKQQMAEELTAFNSILVENTYVSPAKIAFHLN--FDASPHLYQLPNKYRNSCRELFESVGVKSSFSVEDFSGILETVKQGCGRRALSEDNFQLCRRVISEGIWSLIRDKSQEH 2442
stickleback SIRPT2-sr3  ------KPSVGLVISQLKKLSQSFDG-VTLYQENITNACYKFLHEEMLQDKKATEQISEELKEFNSILVENTYVNPSKVAFHLN--FDAAPHLYQLPNKYRNSCRELFENVGVQASFTVEDFATVLETVKEECGRRVLTEENFQLCRRIISEGIWSLIRDKNQEY 2445
medaka SIRPT2-sr3       ------KPSVALVISQLKKLSQSFDG-VTLYQENITNACYKYLHEEMFQDENAKDLITEELKAFNSILVENTYVSPSKVAFHLN--FDVAPHLYQLPNKYRNSCRELFENVGVQPNFTVEDFSTVLETVRQECGRRALTEENFQLCRRIISEGIWSLIRDKNQEY 2445
zebrafish SIRPT2-sr3    ------KPTVSLVISQLKELSKSFDG-VTLYQENITNACYKYLHEELLQSNAAKEEIMEELKTFSSILVENTYVEPLKVAFNLN--FDAAPYLYQLPNKYRNSCRELFENVGVHASFTVETFSGVLQLIKNECGRKPLTEENFQLSRRIISEGIWSLIRDKSQEF 2445
human SIRPT3-sr3        MLNVNLDPPLDKVINNCRNICNIT---TLDEEMVKTRAKVLRSIYEFLSAEKREFRFQLRGVAFVMVEDGWKLLKPEEVVINLEYESDFKPYLYKLPLELG-TFHQLFKHLGTEDIISTKQYVEVLSRIFKNSEGKQLDPNEMRTVKRVVSGLFRSLQNDSVKVR 3896
orangutan SIRPT3-sr3    MLNVNLDPPLDKVINNCRNICNIT---TLDEEMVKTRAKVLRSIYEFLSAEKREFRFQLRGVAFVMVEDGWKLLKPEEVVINLEYESDFKPYLYKLPLELG-TFHQLFKHLGTEDIISTKQYAEVLSRIFKNSEGKQLDPNEMRTVKRVVSGLFRSLQNDSVKVR 3896
dog SIRPT3-sr3          MLNVNLDPPLDKVINNCRNICNIT---TLDEEMVKTRAKVLRSIYEFLSAEKREFRFQLRGVAFVMVEDGWKLLKPEEVVINLEYESDFKPYLYKLPLELG-TFHQLFKHLGTEDIISTKQYVEVLSRIFKNSEGKQLDPNEMRTVKRVVSGLFKSLQNDSVKVR 3894
horse SIRPT3-sr3        MLNVNLDPPLDKVINNCRNICNIT---TLDEEMVKTRAKVLRSIYEFLSAEKREFRFQLRGVAFVMVEDGWKLLKPEEVVINLEYESDFKPYLYKLPLELG-TFHQLFKHLGTEDIISTKQYVEVLSRIFKNSEGKQLDPNEMRTVKRVVSGLFRSLQNDSVKVR 3893
mouse SIRPT3-sr3        MLNVNLDPPLDKVINNCRNICNIT---TLDEEMVKTRAKVLRSIYEFLSAEKREFRFQLRGVAFVMVEDGWKLLKPEEVVINLEYEADFKPYLYKLPLELG-TFHQLFKHLGTEDIISTKQYVEVLSRIFKSSEGKQLDPNEMRTVKRVVSGLFKSLQNDSVKVR 3898
rat SIRPT3-sr3          MLNVNLDPPLDKVINNCRNICNIT---TLDEEMVKTRAKVLRSIYEFLSAEKREFRFQLRGVAFVMVEDGWKLLKPEEVVINLEYEADFKPYLYKLPLELG-TFHQLFKHLGTEDIISTKQYVEVLSRIFKNSEGKQLDPNEMRTVKRVVSGLFKSLQNDSVKVR 3897
zebrafinch SIRPT3-sr3   MLNVNLDPPLDKVINNCRNICNIT---TLDEDMVKTRAKVLRSIYEFLSTEKREFRFQLRGVSFVMVEEGWKLLKPEEVVINLEYESDFKPYLYKLPLELG-TFHQLFKHLGTEDIISTKQYVEVLGRIYKSSEGKQLDPNEMRTVKRVVSGLFKSLQNDSVKVR 3895
chicken SIRPT3-sr3      MLSVNLDPPLDKVINNCRNICNIT---TVDEEMVKTRAKILRSIYEFLSTEKREFRFQLRGVSFVMVEEGWKLLKPEEVVINLEYESDFKPYLYKLPLELG-TFHQLFKHLGTEDVISTKQYVEVLGRIFKNSEGKQLDPNEMRTVKRVVSGLFKSLQNDSVKVR 3890
anole SIRPT3-sr3        MLSVNLDPPLDKVINNCRNICNIT---TLDEDMVKTRVKVLRSIYEFLSAEKREFRFQLRGVAFVMVEEGWKLLKPEEVVINLEYESDFKPYLYKLPLELG-TFHQLFKHLGTEDVISTKQYVEVLSRIFKNSEGKQLDPNEMRTVKRVVSGLFKSLQNDSVKVR 3889
fugu SIRPT3-sr3         TLAVTLDPPLEKVISNCKNICNIS---SPDDEMVKTRNKVLRSTYEFLNGDKRDFRYHLRGVAFVIVEDGWKLLKSEEVVINLDNESDFKPYLYKLPLELG-TFHQLFKLLGAEDIVSTKQYTEVLWRIYRNSEGKQLDPNEMRTVKRVVSGFFKSLHNDPVEIR 3896
tetraodon SIRPT3-sr3    LLGVNLDPPLEKVISNCKNICNIS---SPDDEMVKTRNKVLRSTYEFLNGDKRDFRYHLRGVAFVIVEDGWKLLKSEEVVINLDNESDFKPYLYKLPLELG-TFHQLFKLLGTEDVVSTKQYTEVLWRIYKNSEGKQLDPNEMRTVKRVVSGFFKSLHNDPVEIR 3894
stickleback SIRPT3-sr3  MLGVNLDPPLEKVLSNCKNICNIS---NPDDEMVKTRNKVLRSTYEFLNADKRDFRYQLRGVAVVMVEDGWKLLKPEEVVINLENESDFKPYLYKLPLELG-TFHQLFKLLGTEDVVSTKQYVEVLWRIYKSSDGKQLDPNEMRTVKRVVSGLFKSLQNDPVAIR 3896
medaka SIRPT3-sr3       MLGVNLDPPLDKVISNCKNICNIA---NPDDEMVKTRNKVLRSTYEFLTADKRDFSHQLRGIAFVMVEDGWKLLKPEQVVINLDNESDFKPYLYKLPLELG-TFHQLFKLLGTEDVVSTKQYVEVLWRIFRNSEGKQLDPNEMRSVKRVVSGLFKSLQNDPVEIR 3896
zebrafish SIRPT3-sr3    MLNVNVEPPLEKVISNCKNICSVT---NLDDDTVKTRNKVLRSIYEFLNADKNDFRFQLRGVAFVMVEEGWKLLKPEEVVINLDNESDFKPYLYKLPLELG-TFHQLFKLLGTEDIVSTKQYVEVLCRIFRISEGKQLDPNEMRTVKRTVSGLFKSLQNDAVQVR 3897
                               *    *:.: . :           :              .:           .      :             ::     :  *:*: :*     . .:**:  *       .    ::  :        :  :: :     :   :  *
                                                         C A   T                   X                                   S   K K              R AE              X            X       G    X  P
                                                                                                                                                                                           V
```

Supp. Figure S6. Multiple alignment of *SIRPT1-sr3*, *SIRPT2-sr3* and *SIRPT3-sr3* amino acid sequences of vertebrate sacsin proteins. Sequences (human, orangutan, dog, horse, mouse, rat, mouse, zebrafinch, chicken, anole, fugu, tetraodon, stickleback, medaka, zebrafish) were aligned by *ClustalW2* using default parameters. Then missense and nonsense mutations were mapped on the generated alignment. Amino acid changes due to mutations known to be pathogenic are highlighted in red (missense mutations) and purple (nonsense mutations) (see Table 1 for reference). Amino acid changes that are known to be non-pathogenic are highlighted in green (Supp. Table S3). The following mutations are mapped: p.M1311K, p.Q1345X, p.Q1370X, p.L2374S, p.R2426X, p.R3792X, p.Y1245C, p.I1251T, p.D1330E, p.A1373P, p.A1373V, p.T2316A, p.N2380K, p.C2395R, p.V2397A, p.S2434G.

```
human SIRPT1-srX          -------------AEEKLHLLEFVLSDQAYSELLGLELLPLQNGNFVPFSSSVSDQDVIYITSA------EYPRSLFPSLEGRFILDNLKPHLVAALKEAAQTRG---------------RPCTQLQLLNPERFARLIKEVMNTFWPGRE  759
orangutan SIRPT1-srX      -------------AEEKLHLLEFVLSDQAYSELLGLELLPLQNGNFVPFSSSVSDQDVIYITSA------EYPRSLFPSLEGRFILDNLKPHLVAALKEAAQTRG---------------RPCTQLQFLNPERFARLVKEVMNTFWPGRE  759
dog SIRPT1-srX            -------------AQDKLHLLEFVLSDQAYSELLGLELLPLQNGNFVPFSSSVSDQDVIYITSE------DYPRSLFPGLEGRFILDSLKPHLIAALKEAAQTRG---------------RPCTQLQLLNPERFARLIKEVMNTFWPGRE  757
horse SIRPT1-srX          -------------ARERLHLLEFVLSDEAYSELLGLELLPLQNGHFVPFSSSVSDQDVVYITSE------DYPRSLFPGLEGRFILDNLKPHLLAALKEAAQTRG---------------RPCTQLQLLNPERFARLIKEAMNTFWPGRE  756
mouse SIRPT1-srX          -------------AEEKLHLLEFVLSDQAYSELLGLELLPLQSGAFVPFSSSVSDQDVVYITSE------EFPRSLFPGLEARLILENLKPHLLAALKEAAQTRG---------------RPCTQLQLLNPERFARLIKEVMNTFWPGRE  761
rat SIRPT1-srX            -------------AEEKLHLLEFVLSDQAYSELLGLELLPLQSGAFVPFSSSVSDQDVVYITSE------EFPRSLFPGLEARLILENLKPHLLAALKEAAQTRG---------------RPCTQLQLLNPERFARLIKEVMNTFWPGRE  760
zebrafinch SIRPT1-srX     -------------AEEKLNLLEFVLSDGVYSELIGLELLPLQNGSFIPFSSSVSEQDVVYITSE------EYPRSLFPGLEGRLLSDDLKPEVLAALKEAAKSRG---------------RPCTQLQLLNPERFARLVKEVMNSVWPGRD  757
chicken SIRPT1-srX        -------------AEEKLHLLQFVLSDGVYSELIGLELLPLQNGNFISFSSSVSEQDIVYITSE------DFPRSLFPGLEGRLLSDDLKPEVLAALKEAAKSRG---------------RPCTQLQLLNPERFTRLIKEVMNSVWPGRD  751
anole SIRPT1-srX          -------------ADAKLHILDFVLSDGNYNELIGLELLPLQNGSFTLFSSSASDQDAVYITSE------SYPRSLFPGFEGRLLADDVKPHILAALKEAAKSRG---------------RPCTQLQLLNPERFARLIKEVMNALWPRRE  751
fugu SIRPT1-srX           -------------SQEKLLLLEFVLSDANYSDLIGLELLPLQDETFTSFSSPVSEKDAIYITSE------EYPRFLYPGLEGRFILESIKSSVMDSLKKAAKSRG---------------RPCTQLQMLSPERSARLIKDILSSVWPTRD  760
tetraodon SIRPT1-srX      -------------SQEKLSLLEFVLADANYSDLIGLELLPLQDETFASFASPVSDKDSVYMASE------EYPRCLYPGLEARFVLESIKPSVKDGLMKAAKSRG---------------RPCTQLQVLSPERSARLIKDVLSSAWPTRD  757
stickleback SIRPT1-srX    -------------SQEKLLLLEFALSDGNYSDLIGLELLPLQDGTFTTFSSSFSDKDSIYIASE------QYPKSLYPGLEGRFLLESIKPLVMDALKEAAKSRG---------------RPCTQLQMLNSERSARLIKEILSTAWLSRD  760
medaka SIRPT1-srX         -------------AQEKLLLLEFALSDANYSDLIGLELLPLQDKTFATFSSSVSEKDAVYIPSE------DYPRVLYPGLEHRFILESIKPSVMDSLKEAANSRG---------------RPCTQLQELNPERSARLIKEILSTAWTTRE  760
zebrafish SIRPT1-srX      -------------STEKLLLLEFILSDSCYNDLIGLELLPLQDETFAVFSSSVNDKDAVYIASQ------EYPRSLYPGLEGRFVLESIPPHVMTSLKEAAKSRG---------------RPCTQVQVLTPERSARLIKEVLTTAWPSRD  759
human SIRPT3-srX          HCLIDADIPVSYVTPADIRSFLMTFSSPDTNCHIGKLPCRLQQTNLKLFHSLKLLVDYCFKDAEENEIEVEGLPLLITLDSVLQTFDAKRPKFLTTYHELIPSRKDLFMNTLYLKYSNILLNCKVAKVFDISSFADLLSSVLPREYKTK-  3229
orangutan SIRPT3-srX      HCLIDADIPVSYVTPADIRSFLMTFSSPDTNCHIGKLPCRLQQTNLKLFHSLKLLVDYCFKDAEENEIEVEGLPLLITLDSVLQTFDAKRPKFLTTYHELIPSRKDLFMNTLYLKYSNILLNCKVAKVFDISSFADLLSSVLPREYKTK-  3229
dog SIRPT3-srX            HCLIDADIPVSYVTPADVRSFLMTFSSPDTNCHIGKLPCRLQQTNLKLFHSLKLLVDYCFKDAEENEIEVEGLPLLITLDSVLQTFDAKRPKFLTTYHELIPSRKDLFMNTLYLKYSNILLNCKVAKVFDISSFADLLSSVLPREYKTK-  3227
horse SIRPT3-srX          HCLIDADIPVSYVTPADVRSFLMTFSSPDTNCHIGKLPCRLQQTNLKLFHSLKLLVDYCFKDAEENEIEVEGLPLLITLDSVLQTFDAKRPKFLTTYHELIPSRKDLFMNTLFLKYSNILLNCKVAKVFDISSFADLLSSVLPREYKTK-  3226
mouse SIRPT3-srX          HCLVDADIPVSYVTPADVRSFLMTFSSPDTNCHIGKLPCRLQQTNLKLFHSLKLLVDYCFKDAEESEFEVEGLPLLITLDSVLQIFDGKRPKFLTTYHELIPSRKDLFMNTLYLKYSSVLLNCKVAKVFDISSFADLLSSVLPREYKTK-  3131
rat SIRPT3-srX            HCLVDAAIPVSYVTPADVRSFLMTFSSPDTNCHIGKLPCRLQQTNLKLFHSLKLLVDYCFKDAEESEFEVEGLPLLITLDSVLQIFDGKRPKFLTTYHELIPSRKDLFMNTLYLKYSSVLLNCKVAKVFDISSFADLLSSVLPREYKTK-  3230
zebrafinch SIRPT3-srX     HCLVDADIPVTYVTPADVRLFLMTFSFPDSNCHIGKLPCRLQQTNLKLFHSLKLLVDYCFKDAEENEIQIEGLPLLITLDNVLQVFDSKRPKFLTTYHELIPSRKDLFMNTLYLRYNNILLSSEVAKVFDIRSFAELLSSVLPREYKTR-  3228
chicken SIRPT3-srX        HCLVDADIPVTYVTPADVRLFLMTFSFPDSNCHIGKLPCRLQQTNLKLFHSLKLLVDYCFKDAEENEIQIEGLPLLITLDNVLQIFDSKRPKFLTTYHELIPSRKDLFMNTLYLRYNNILLSSDVAKVFDIASFAELLSSVLPREYKTK-  3223
anole SIRPT3-srX          HCLVDADIPVSYVTPADVRSFLLTFSSPDSNCHIGKLPCRLQQTNLKLFHSLKLLVDYCFKDAEENEIEVEGLPLLITLDSVLQVFDSKRPKFLTTYHDLIPSRKDLFMNTLYLKYSNILLKCSVAKVFDISSFADLLSSVLPREYKAR-  3222
fugu SIRPT3-srX           TCLEATGIPVSYVTPKDVRNFLYTFSSPDTTCHIGKLPCRIQHSHYKLLHNLKLLVDYCFKDVDVDEVKIEGLPLLITIDNMLQVFDSKRPKFLTLHHELISSRKELFINTLFMRYSNILLKEGIAKNFDISSFGDLLGSVLPREYQTK-  3231
tetraodon SIRPT3-srX      ACLEAAGIPVSYVTPRDVRNFLYTFSSPDTTCHVGKLPCRIQHSHYKLLHNLKLLVDYCFKDMEVDEVKLEGLPLLITMDNMLQVFDSKRQKFLTVHHELVSSRKDMFINTLFMRYSSILMKQGIAKHFDISSFGDLLGSVLPREYRTK-  3228
stickleback SIRPT3-srX    FCLEDAGIPVSYVTPDDVRHFLHTFLSPDTSCHVGKLPCRIQQSNFKLVHNLKLLVDYCFKDREAGDLTIEGLPLLITMDNMLQVFDSKRPKFLTTHHELISSRKEMFMNTLYIRYSNILLMAGAAKTFDISCFADLLGSVLPREYRTR-  3231
medaka SIRPT3-srX         FCLEDAGIPVSYVTPTDVRNFLQTFSSPDTSCHVGKLPCRLQQSNYRLLHSLKLLVDYCFTDIEEGEVKIEGLPLLITMDGMLQVFDSNRPKFLTTHHELISSRKEMFMNTLYLKYCNVLLKAQVAKNFDISSFGDLLGSVLPREYHTR-  3231
zebrafish SIRPT3-srX      FCLEDAGIPVNYVTPEDVRSFLLTFSSPDTSCQVGKLPCRLQQSNFKLFHGLKLLVDYCFKDVEENDINIEGLPLLMTMDNILQVFDSRHPKFLTTYHELIATRKELFMHTLYIRYSNILKAGVAKTFDINSFGDLLGSVLPRECKAR-  3231
                                       :   :  :   :      .  .:*     :*      . .     *  :          .     * .        :     .     .   :*                  : :      *: . :     :
                                                                                    T                                        SX                                  X         X

human SIRPT1-srX          LIVQWYPFDENRNHPSVSWLKMVWKNLYIHFSED------------LTLFDEMPLIPRTILEEGQTCVELIR--LRIPSLVILDDESEAQLPEFLADIVQKLGGFVLKKLDASIQHP-----LIKKYIHSPLPSAVLQIMEKMPLQKLC  889
orangutan SIRPT1-srX      LIVQWYPFDENRNHPSVSWLKMVWKNLYIHFSED------------LTLFDEMPLIPRTILEEGQTCVELIR--LRIPSLVILDDESEAQLPEFLADVVQKLGGFVLKKLDASIQHP-----LIKKYIHSPLPSAVLQIMEKMPLQKLC  889
dog SIRPT1-srX            LIVQWYPFDEDKNHPSVSWLKMVWKNLYIHFSED------------LTLFDEMPLIPRTTLEEGQTCMELIR--LRIPSLVILDDESEAQLPEFLADIVQKLGGIVLKKLDASIQHP-----LIKKYIHSPLPSAVLQIMEKMPLQKLC  887
horse SIRPT1-srX          LIVQWYPCDKDKRHPSVSWLKMVWKNLYIHFSED------------LTLFDEMPLIPRTALEEGQMCVELIR--LRIPSLVILDDESEAQLPEFLADIVQKLGGIILKKLDASIQHP-----LIKKYINSPLPSAVLQIMEKMPLQKLC  886
mouse SIRPT1-srX          LVVQWYPFSEDKRHPSLSWLKMVWKNLYIHFSED------------LTLFDEMPLIPRTLLNEDQTCVELIR--LRIPSVVILDDETEAQLPEFLADIVQKLGGIVLKRLDTSIQHP-----LVKKYIHSPLPSAILQIMEKIPLQKLC  891
rat SIRPT1-srX            LVVQWYPLSEDKHHPSISWLKMVWKNLYIHFSED------------LTLFDEMPLIPRTPLDADQTCVELIR--LRVPSVVILDDETEAQLPEFLADIVQKLGGIVLKRLDTSLQHP-----LVKKYIHSPLPSAILQIMEKIPLQKLC  890
zebrafinch SIRPT1-srX     VVVQWYPGLEEKKHPSVSWLKMVWKNLYIHFSDD------------LSVFDEMPLIPRTLLEENQTSVELVR--FKNPSPIILEDESETQLPEYLADIIQKIGGVVLKKLDISIQHP-----LIKKYVHPPLPSAVLQIMEKMSLQKLC  887
chicken SIRPT1-srX        MVVQWYPGLEDKNHPSISWLKMVWKNLYMHFSDD------------LSAFDDMPLIPKTLLEENQTSLELVR--FRNPSPVILEDESETQLPDYIADIIEKVGGVVLKKLDVSIQHP-----LIKKYVHPPLPSAVLQIMEKVSLQKLC  881
anole SIRPT1-srX          IVVQWFPLSEDKSQPPVSWLKMVWKNLYLHFSGD------------LSMFDDMPLLPKTLLDEGEEIVELIR--LRTPSPIILDDGLETQLPEFLPDIIEQLGGIVIKKLDPAIQHP-----LLKKYIHAPLPSVVLQIVEKLSLQKLT  881
fugu SIRPT1-srX           FTVEWEPGNRELKHPTISWLRMIWKHLYIHFSDD------------LSTFEDMPLIPLVPLEESMNSVHLLR--LRTPSAIILVEGEETTSSGILLDIMEKLGGTVMKKLDSCLQHP-----LLKNYIHPSSPAVLLQIMDRLSKQRLS  890
tetraodon SIRPT1-srX      FAVEWEPGNRELRHPTLSWLRMVWKHLYIHFAED------------LSTFEDMPLIPLVPLEENMTRVHLLR--LRTPSPIIFADAEEAAPSGNLLDIMEKLGGTVMTKLDSCLQHP-----LLKNYIHPSSPAALLQIMDRLSKQRLA  887
stickleback SIRPT1-srX    FIVRWEPGNKELKHPTVSWLKRIWKHLYIHFADD------------LSTFEDMPLIPLVPLEESMEVAQLLR--LKTPSPVILLDEEESSLPENLVDIMEKLGGVVIKKLDSSLQHP-----QLKNFVHPSSPGLLLQIMDRLPTQRLS  890
medaka SIRPT1-srX         FSVKWEPGNGELKHPTVSWLKMIWRHLYIHFADD------------LYTFEDMPLIPLVPLDESMNNITLLR--LKSPSPIILIDDKQAPFSENLLEVVEKLGGVAMKKMDPCLQHP-----LLKKYIHPCSPSILLQIMGRQSTQRLS  890
zebrafish SIRPT1-srX      FAVQWNPGDQEKKHPSASWLRMIWKHLYINFADD------------LSVFEDLPLIPNVPLADTLDCLELLR--LKSPSPIVLVNEEEGPPSEDLLEVMKKLGCVILKQIDPCLHHP-----LLKNYIHQSSPSTLLQIMDRSTSQRLA  889
human SIRPT3-srX          SCTKWKDN-----FASESWLKNAWHFISESVSVKEDQEETKPTFDIVVDTLKDWALLPGTKFTVSANQLVVPEGDVLLPLSLMHIAVFPNAQSDKVFHALMKAGCIQLALNKICSKDSAFVPLLSCHTANIESPTSILKALHYMVQTSTF  3374
orangutan SIRPT3-srX      SCTKWKDN-----FASESWLKNAWHFISESVSVKEDQEETKPTFDIVVDTLKDWALLPGTKFTVSANQLVVPEGDVLLPLSLMHIAVFPNAQSDKVFHALMKAGCIQLALNKICSKDSAFVPLLSCHTANIESPTSILKALHYMVQTSTF  3374
dog SIRPT3-srX            NCTKWKDN-----FASESWLKNAWHFISESVNVKEDQEETQPIFDIVVETLKDWALLPGTKFTVSANQLVVPEGDVLLPLSLMHIAVFPNAQSDKVFHALMKAGCIQLALNKICSKDSAFVPLLSCHTANIESPTSILKAVHYMVQTSTF  3372
horse SIRPT3-srX          NCTKWKDN-----FASESWLKNTWHFISESVSVKEEQEETKPVFDVVVDTLKDWALLPGTKFTVSANQLVVPEGDVLLPLSLMHIAVFPNAQSDKVFHALMKAGCIQLALNKICSKDSAFVPLLSCHTANIESPASILKALHYMVQTSTF  3371
mouse SIRPT3-srX          NCAKWKDN-----FASESWLKNAWHFISESVSVTDDQEEPKPAFDVIVDILKDWALLPGTKFTVSTSQLVVPEGDVLIPLSLMHIAVFPNAQSDKVFHALMKAGCIQLALNKICSKDSALVPLLSCHTANIDSPASILKAVHYMVQTSTF  3376
rat SIRPT3-srX            NCAKWKDN-----FASESWLKNAWHFISESVSVTDDQEEPKPAFDVIVDILKDWALLPGTKFTVSASQLVVPEGDVLIPLSLMHIAVFPNAQSDKVFHALMKAGCIQLALNKICSKDSALVPLLSCHTANIDSPASILKAVHYMVQTSTF  3375
zebrafinch SIRPT3-srX     NCMKWKEN-----FASESWLKSAWHFISESLNIKEEQEDTQAKFDGVIETLKDWTLLPGVKFTVSANHLVVPESDVLLPLSIMHIAVFPNAQSDKVFHALMKTGCIQLALNKICSKDSALVPLLSGRTANIDNPSSILKAIQYMVQTSTF  3373
chicken SIRPT3-srX        SCMKWKEN-----FASESWLKNAWHFISESIAVKEEQEDIQAKFDGVVETLKDWTLLPGVKFTVSANHLVVPECDVLLPLSIMHIAVFPNAQSDKVFHALMKTGCIQLALNKICSKDSALVPLLSASTANIDNPSSILKAIQYMVQTSSF  3368
anole SIRPT3-srX          GCIRWKEN-----FASESWLKNAWHFISESLHAKDDQDDVKPTFEDVVDTLKDWALIPGAKFTVSSNQLVVPDCDVLLPLSLMHIALFPNSQTDKVFHALMKTGCIQLALNKICSKDNALVPLMSAHTANIDNPSSILRAIHYMVQTSTF  3367
fugu SIRPT3-srX           VPVKWRDT-----FASESWLKNVWNFISENIAIKEDQLERTLGFDTVADILKDWALLPGTKFMAR-DKIVVPEHDVLLPLSLMHLAIFPQGQNDKVFHTLMKSGCIQLALNKISVKENPIMPILAQHTANIENPQSILRAVEYMIQASAF  3375
tetraodon SIRPT3-srX      VPVKWRDT-----FASESWLKNVWNFISENVAAKGDQLEHTPGFDTAMDILKDWALLPGTKFLAK-EKIVVPEHDVLLPLSLMHLAIFPQGQNDKVFHTLMKSGCIQLAVNKISVKENPIMPILAQHTANIENPQSILRAVEYMIQASAF  3372
stickleback SIRPT3-srX    IPVKWRDT-----FASESWLKSVWNFISENIAVKEDQVDIKPSFETLLDILKDWALLPGMKFMAR-DKIVVPEHDVLLPLSLMSIAIFPHGQNDKVFHTLMKAGCIQLAVTKICLKDNPIMPFLAQHTANIENPSSVLKAVEYIIQTATF  3375
medaka SIRPT3-srX         VPVKWRDT-----FANDSWLKNVWNFLSENIAVKEDQVDIKPSFDTMVDILKDWALLPGLKFIAR-DKIVVPDHDMLLPLSLMNIAIFPQGQNDKVFHTLMKAGCIQLAVNKICVKDNQIMHFLTQHTANIENPSSILKATEYMIQMSAF  3375
zebrafish SIRPT3-srX      SSVKWKEN-----FPTESWLKSSWHFISEQLANKDDQAENKLKFETILEILKDWALLPGIKFLVSGNKLVVPEHDVLLPLSLIFAAIFPNGQNDKVFHTLMKGGCFQLAVNKICAKENPMLPLLAQHTANIDNPSSILRALEYMIQISAF  3376
                             .*          .  ***:  *. :   .                  :.: .*:*  :         :     .  *  ::             : . : : *   :     . . :.              :    *  :*:
                                                   R                              F                                                K                          A
```

(continue)

```
human SIRPT1-srX          NQITSLLPTHKDALRKFLASLTDSS-EKEKRIIQELAIFKRINHSSDQGISSYTKLKGCKVLHHTAKLPADLRLSISVIDSSDEATIRLANMLKIEQLKTTSCLKLVLKDIENAFYSHEEVTQLMLWVLENLSSLKNENPN-----VLEW 1033
orangutan SIRPT1-srX      NQITSLLPTHKDALRKFLASLTDSS-EKEKRIIQELAIFKRINHSSDQGISSYTKLKGCKVLHHTAKLPADLRLSISVIDSSDEATIRLANMLKIEQLKTTSCLKLVLKDIENAFYSHEEVTQLMLWVLENLSSLKNENPN-----VLEW 1033
dog SIRPT1-srX            NQIASLLPTHKDALRKFLASLTDSS-EKEKRIIQELPIFKRINHLSDQGISSYTKLKGCKVLHHTAKLAPDLRLSISVIDSSDEATIRLANMLKIEKLKTTHCIKLILRDIENAFYSHDEVTHLMLWILENLSSLKNENPN-----VLDW 1031
horse SIRPT1-srX          NQIASLLPTHKDALRKFLAGLTDSS-EKEKRIIQELTIFKRVNHSSGQGLSSYTKLKGCKVLHHTAKLPPGLRLSVPVIDSSDEATIRLANMLKIETLKTTSCLQLVLKDMENAFYSHEEITHLMLWILENLSSLKNENPN-----VLDW 1030
mouse SIRPT1-srX          NQIASLLPTHKDALRKFLASLTDTS-EKEKRIIQELTIFKRINHSSDQGISSYTKLKGCKVLHHTAKLPTDLRLSISVIDSSDEATIRLANMLKIEKLKTTSCLKFVLKDIGNAFYTQEEVTQLMLWILENLSSLKNENSN-----VLDW 1035
rat SIRPT1-srX            NQIASLLPTHKDALRKFLASLTDTS-EKEKRIIQELTIFKRINHSSDQGISSYTKLKGCKVLDHTAKLPTELRLSVSVIDSSDEATIRLANMLKIEKLKTTSCLKFVLKDIENAFYTQEEVTRLMLWILENLSSLKNENPS-----VLDW 1034
zebrafinch SIRPT1-srX     NQVASFPPTHKDALRAFLASLTDAN-EKEKRIIQELLMFKKVEKSSEESVPVYTGLNGSKVLHHTAKIPPGLRFSVPIIDSSDEATIRLANLLKIEQLKSTDCLKFVIEDIRSDFYSYDETAQIMQWVLENLTFLKNENTD-----VIDW 1031
chicken SIRPT1-srX        NQVSSLPSTHKDALRAFLASLNDVS-EKEGRIIQELLIFKKMEKSSDDGVPVYAGLNGSKVLHHTAKIPPGLRFSVPVIDSSDEATIRLANLLKIEQLKSTDCLKFIIQDIRSCFYSYDETTQIMQWVLENLTFLKNENTD-----VIDW 1025
anole SIRPT1-srX          SQITSFSTSYKNALRSYLASLTDIT-EKEKKVIHELVIFRRISQSLDDDI-AFTSLKSCKVLHHTAKIPPDIKLSISLVDSSDEATIRLVKILKAEQLRSTDCIKFILNDIQSDFYCNEEATKIMLWILENLTFLKNENAE-----VLDW 1024
fugu SIRPT1-srX           SQVTSLSITEKIALRKYLAGLSDVT-EREKHTLLELSIFEKFG-TSCEGTSKFTSLRGARALHHRAKYPPNVKLSINLVGYCDEESLRLIKMLNIEQITTTECLKFIVHDIERGFYTTDEITQIMLWALQNMAFLKNENSS-----VLGW 1033
tetraodon SIRPT1-srX      SQVSSFSLTEKIALRKYLAGLSDVT-EREKHTLLELSVFEKVG-SSCEGSSRFTSLRGARALHHRAKYPPDVKLSINLVGYCDEESIRLIKMLNIEQLTTTECLKFIVHDIERGFYTTEEMAHIMLWALQHMAFLKNENPS-----VLSW 1030
stickleback SIRPT1-srX    GLVSSFSVKEKVALRNFLAGLSDIT-AREKHTLLELSIFEKVG-TCSEGTSAFTSLRGARALHHRAKYPPDVKLSINLVGCCDEESIRLIKMLNVQQLKTTECLKMIIQDIERGFYTTDEVTQVMLWALKHLAFLKNENPT-----VIRW 1033
medaka SIRPT1-srX         SQVSSLSDKEKTALRNYLAGLPDIT-EKEKHTLLDLSIFDKVG-GFREGSPVFTSLRGARALHHRAKYPPDVKLSQSLVACSDEESIRLFKMLNVEQLKTTECLKMIIQDMETGFYTKDETTKIMLWALKHLAFLKNENSC-----VIGW 1033
zebrafish SIRPT1-srX      SQVSSLTVKQRLALRSFLAGLTDVT-EKEKRIIQELPIFDKVGQKSKTDTSPLTLLKGARVLHHTAKHPLDVKLSINVVDCSDEATIRLVKLLNIEQVKSTECLKVIIQDIEKGFYTKEEVTKIMLWALTYLSFLKNENKA-----VISW 1033
human SIRPT3-srX          RAEKLVENDFEALLMYFNCNLNHLMSQDDIKILKSLPCYKSIS----GRYVSIGKFGTCYVLTKSIPSAEVEKWTQSSSSAFLEEKIHLKELYEVIGCVPVDDLEVYLKHLLPKIENLSYDAKLEHLIYLKNRLSSAEELSEIKEQLFEK 3520
orangutan SIRPT3-srX      RTEKLVENDFEALLMYFNCNLNHLMSQDDIKILKSLPCYKSIS----GRYVSIAKFGTCYVLTKSIPSAEVEKWTQSSSSAFLEEKIHLKELYEVIGCVPVDDLEVYLKHLLPKIENLSYDAKLEHLIYLKNRLSSAEELSEIKEQLFEK 3520
dog SIRPT3-srX            RAEKLVENDFEALLMYFNCNLSHLMSQDDIKILKSLPCYKSIS----GRYMSIAKFGTCYVLTKSIPSAEVEKWTQSSLSAFLEEKIHLKELYEVLGCVPVDDLEVYLKHLLPKIENLSYDAKLEHLIYLKNRLSSIEELSEIKEQLFEK 3518
horse SIRPT3-srX          RTEKLVENDFEALLMYFNCNLSHLMSQDDIKILKSLPCYKSIS----GRYMSIAKFGACYVLTKSIPSAEVEKWTQSSPSAFLEEKIHLKELYEVLGCVPVDDLEVYLKHLLPKIENLSYDAKLEHLIYLKNRLSSIEELSEIKEQLFEK 3517
mouse SIRPT3-srX          RTEKLMENDFEALLMYFNCNLSHLMSQDDIKILKSLPCYKSIS----GRYMSIAKFGTCYVLTKSIPSAEVEKWTQSSSSAFLEEKVHLKELYEVLGCVPVDDLEVYLKHLLPKIENLSYDAKLEHLIYLKNRLASIEEPSEIKEQLFEK 3522
rat SIRPT3-srX            RTEKLVENDFEALLMYFNCNLSHLMSQDDLKILKSLPCYKSIS----GRYMSIAKFGTCYVLTRSIPSAEVEKWTQSSSSAFLEEKVHLKELYEVLGCVPVDDLEVYLKHLLPKIENLSYDAKLEHLIYLKNRLASIEELSETKEQLFEK 3521
zebrafinch SIRPT3-srX     KTEKLAESDFEALLMYFNCNLCHLTSQDDIKILKSLPCYKSIS----GRYISIAKYGTCYVLTKSIPSVEVDRWTQSTSSAFLEEKVHLKDLYTMLGCVSVDDLEVYLKHLLPKIESLSYDAKLEHLIYLKNRLTSIEETSEMKEQLFEK 3519
chicken SIRPT3-srX        RTEKLAESDFEALLMYFNCNLCHLTSQDDIKILKSLPCYKSIS----GRYISISKYGTCYVLTKSIPSVEVDRWTQSTSSAFLEEKIHLKDLYTVLGCVSVDDLEVYLKHLLPKIESLSYDAKLEHLIYLKNRLSSIEETSEMKEQLFEK 3514
anole SIRPT3-srX          KTEKLAENDFEALLMYFNCNLNHLTSEDDIKILRSLPCYKSIS----GRYMSISKCGSCYVLTRSIPSAEVEKWTQSASAAFLEEKIHLKDLYTLLGCVPVDDLEVYLKHLLPKIENLSYDAKLEHLIYLKNRLCSIEESSEIKEQLFEK 3513
fugu SIRPT3-srX           KAASLTDKDFEALLLYFNCNLAGLT-QQNAQSLKLLPCFKSIG----GKYVSIANHGANYVLVRNIPTADMDRWAHNGSFAFLADCPQLKELYTFLGCMPIDDLEIYHHHVLPKFESFCYDAKVEHIVYLKERLLSIEEPCDLVDQLYEK 3520
tetraodon SIRPT3-srX      KAASLSDRDFEALLSYFSCNLASLT-QQNAQSLKLLPCFRSVG----GKFMSIANYGASYVLVRNIPTADMDKWAHNGSSAFLADCPQLKELYSFLGCAPIDDLEVYLHHVLPKFESFCYDAKVEHVAYLKERLVSMEEPSDFTDQLYEK 3517
stickleback SIRPT3-srX    KTSNLTEKDFEALLLYFNCNLANLT-KQDAQSLKLLPCFKSVS----GRYISIANNGTNYVLVKSIPTADIDKWAHTAPLGFLADNQQLKDLYTFLGCVPIDDLEIYLQYLLPKFESLSYDAKVEHIVYLKERLLSMEESCEMKNQLYEK 3520
medaka SIRPT3-srX         KTANLTDKDFEALLLYFNCNLVSLT-QQDAQSLKLLPCFKSVS----GRYISIASYGANYVLEKSPPTADIEKWAHTPSSSFLADIPQLKELYTFLGCMPIDDLEIYLHHLLPKFDSLSSDAKVEHIVYLKERLLSMEESCAMKEQLYEK 3520
zebrafish SIRPT3-srX      KTASLTDKDFDALLLYFNCNLSNLA-QDDIQCLKLLPCYKAVS----GRYICISNFGACYVLTKSIPSADIEKWAHNTSSAFLADNPQLKELYGFLGITPIDDLEVYLNHVLPKFDILSHDAKIQHVIYLKERLLAMEEQVSIKDKLFEK 3521
                                       *  : ..*       : : :  *  :  .               .* :         : :          :* .:        .  ::.  . :   :       :::             *:        :
                                                                                E              X                                               R   F   P
                                                                                K

human SIRPT1-srX          LTPLKFIQISQEQMVSAGELFDPDIEVLKDLFCNEEGTYFPPSVFTSP-DILHS--------------LRQIGLKNEASLKEKDVVQVAKKIEALQVGACPDQDVLLKKAKTLLLVLNKNHTLLQSSEGKMTLKKIKWVPACKE 1162
orangutan SIRPT1-srX      LTPLKFIQISQEQMVSAGELFDPDIEVLKDLFCNEEGTYFPPSVFTSP-DILHS--------------LRQIGLKNEASLKEKDFVQVAKKIEALQVGACPDQDVLLKKAKTLLLVLNKNHTLLQSSEGKMTLKKIKWVPACKE 1162
dog SIRPT1-srX            LMPLKFIQISQEQMVSAGELFDPDIEVLKDLFYNEEETCFPPSVFTSP-DILHS--------------LRQIGLKNEASLKENDVVQVAKKIEDLQVSSCPNQDILLKKAKTLLMVLNKNHMLLQSSEGKMTLKKIKWVPACKE 1160
horse SIRPT1-srX          LMPLKFIQISQEQIVSASELFDPDIEILKDLFYDEEETCFPPSVFTSP-DILHS--------------LRQIGLKNEASLKEKDVVQVAKKIEGLQVSSCPNQDVLLKKAKTLLLVLNKNHALLQSAEGKMTLKKIKWVPACKE 1159
mouse SIRPT1-srX          LMPLKFIHMSQGHVVAAGDLFDPDIEVLRDLFYNEEEACFPPTIFTSP-DILHS--------------LRQIGLKNESSLKEKDVVQVARKIEALQVSSCPNQDVLMKKAKTLLLVLNKNQTLLQSSEGKMALKKIKWVPACKE 1164
rat SIRPT1-srX            LMPLKFIHMSQEHVVAASDLFDPDIEVLRDLFYNEEEACFPPAVFTSP-DILHS--------------LRQIGLKNEASLKEKDVVQVAKKIEALQVSSCQNQDVLLKKAKTLLLVLNKNHTLLQSSEGKMALKKIKWVPACKE 1163
zebrafinch SIRPT1-srX     LTPLKFIQISPEKIVSANELFDPEVELLQHLFYAEEESCFPPVIFTS--DILHS--------------LRQIGLKNEANLEENDILRVAKKIESLHADSNTNHDLLLRKARTLLTILNKNHMLLQSSETKATMKKIKWIPASKE 1159
chicken SIRPT1-srX        LAALRFIRISEEKLMTANELFDPEVELLQNLFYSEEEISFPPAIFTSS-DILHS--------------LRQIGLKSEANLEESDIMRVANKIESLHADSNTDCDLLVRKARTLLMILNKNHKLLQSSETKTALKKIKWIPACKE 1154
anole SIRPT1-srX          LAPLKFIQISEGKLVSASELFDPEVEVLQNLFYGEEHCFPPLTFRASSDILHS--------------LRLIGLKSEGSLEEKDILHLAHKIENLRDCTGTNHDALKRKARTLLMILNKSCSLLRSPETKASLKKIKWIPACKE 1154
fugu SIRPT1-srX           LSPIKFIQLPCGKLAKASDLFDPELEILQNLFYMEEKTRFPTSEFTFSSDVLHS--------------LRQLGLRNEVQLNEKDVVTVAKKIEELQHSKDTNEDLVIKKAKMLLQILNRQTKLVKSADAQTALLKLQWVPVCKE 1163
tetraodon SIRPT1-srX      LSPIKFIQLPCGRLVKASDLFDPELEILQNLFYMEEKSRFPTSELASSPDVLHS--------------LRQLGLRNEVQLSERDVVTVAKRIEELQRSQDAKEDLVIKKAKTLLQILNRQTKLVKSADAQSALLKLQWVPVCKE 1160
stickleback SIRPT1-srX    LSALTFIQLPCGKSVKASDLFDSELEILQNLFYMEEKSRFPPGLFKSSADILHS--------------LRQLGLRNEVELTEKDVLKVAMKIEELQSSREPEMELIVKKAKTLLQILNRQTKLVKSADAQMSLLKLKWVPVCKE 1163
medaka SIRPT1-srX         LSTLKFIHMPCGKSVKASDLFDPELEILQNLFYMEEKTRFPTNVFTSSPDILHS--------------LRQLGLKNEVQLNEKDALKVAKKIEELQSSEDPDWDSITKKAKTLLQILNKQTKLIKTADTQASLLKLKWVPVCKE 1163
zebrafish SIRPT1-srX      LSSLKFIHTASEKLHSPTDFFDPELEILQNLFFMEEKVRFPPDDFMSSPDVLHS--------------LRQLGLKNEVQLNEKDVLQVARKIEELQGNSKPEWDPILKKAKTLLTILNKQTKLVKSSEVQTNLQKLKWVPVCKD 1163
human SIRPT3-srX          LESLLIIHDANSRLKQAKHFYDRTVRVFEVMLP--EKLFIPNDFFKKLEQLIKPKNHVTF-MTSWVEFLRNIGLKYILSQ--QQLLQFAKEISVRANTENWSKETLQNTVDILLHHIFQERMDLLSGNFLKELSLIPELCPERA 3659
orangutan SIRPT3-srX      LESLLIIHDANSRLKQAKHFYDRTVRVFEVMLP--EKLFIPNDFFKKLEQLIKPKNHVTF-MTSWVEFLRNIGLKYILSQ--QQLLQFAKEISVRANTENWSKETLQNTVDILLHHIFQERMDLLSGNFLKELSLIPELCPERA 3659
dog SIRPT3-srX            LESLLIIHDANSRLKQAKHFYDRTVRVFEVMLP--EKLFIPKDFFKKLEQLIKPKNQATF-MTSWVEFLRNIGLKYILSQ--QQLLQFAKEISVRANTENWSKETLQNTVDILLHHIFQERMDLLSGNFLKELSLIPELCPERA 3657
horse SIRPT3-srX          LESLLIIYDANSRLKQAKHFYDRTVRVFEVMLP--EKLFIPKDFFKKLEQLIKPKNQVAF-MTSWVEFLRNIGLKYILSQ--QQLLQFAKEISVRATTENWSKETLQNTVDILLHHIFQERMDLLSGNFLKELSLIPELCPERA 3656
mouse SIRPT3-srX          LESLLIIHDANNRLKQAKHFYDRTVRVFEVMLP--EKLFIPKEFFKKLEQVIKPKNQAAF-MTSWVEFLRNIGLKYALSQ--QQLLQFAKEISVRANTENWSKETLQSTVDILLHHIFQERMDLLSGNFLKELSLIPELCPERA 3661
rat SIRPT3-srX            LESLLIIHDANNRLKQAKHFYDRTVRVFEVMLP--EKLFIPKEFFKKLEQVIKPKNQVVF-MTSWVEFLRNIGLKHVLSQ--QQLLQFAKEISVRANTENWSKETLQNTVDILLHHIFQERTDLLSGNFLKELSLIPELCPERA 3660
zebrafinch SIRPT3-srX     LESILIIHDASNRLKPAKYFYDRTVKVFEVMLP--EKFFIPQDFFKKLEQLTKPKNQAAF-LPSWVTFLRHIGLKFIISQ--QQLLQFAKEISMRANTENWSKETLQNTVDVLLHHIFQERADLFSGNFLKELSLIPELCPERA 3658
chicken SIRPT3-srX        LESILIIHDASNRLKPAKYFYDRTVKVFEVMLP--EKLFIPQDFFKKLEQITKPKSQAAF-LPSWVTFLRHIGLKYIISQ--QQLLQFAKEISMRANTENWSKETLQNAVDVLLHHIFQERTDLLSGNFLKELSLIPELCPERA 3653
anole SIRPT3-srX          LESFLIIHDSCNRLKPAKHFYDRTVRVFEVMLP--EKLFIPQDFFKKLEQLTKPKNRAAF-LTSWVAFLRNMGLKYIISQ--QQLLQFAKEISVRANTENWTKDTLQNTVDVLLHHIFQERTDLLSGNFLKELSSIALLCPERA 3652
fugu SIRPT3-srX           LEGLMFIFDYSNRLRPAKFFYDETVKVFQVMLP--SKSFITSDFFKKVEQVSKPKNGALS-LASWITFLRNIGLKHQVSQ--QQVLQFAKEVSIKAQTEGWTKEKVQTTADVLLNHIFNERTDLFQGSFLKELSMIPFLYPERA 3659
tetraodon SIRPT3-srX      LEGLMFIFDCSNRLRPAKFFFDETVRVFQVMLP--AKSFITSDFFKKVEQVSRPKNGIASSLASWITFLRHIGLKHQVSQ--QQVLQFAKEVSIKAQTEGWTKEKVQTAVDVLLSHVFNERTDLFQGSFLKELSMVQFLYPERA 3657
stickleback SIRPT3-srX    LEGLLFIYDYSNRLRPTKFFYDETVKVFKVMLP--TKSFIQSDFFKKVEQVSKPRNGTLL-LTSWITFLRNIGLKHKVSQ--QQVLQFAKEVSIKAQTESWSQEKVQTIVDVLLNHIFNDRTDLLEGTFLKELSMIPELCPERA 3659
medaka SIRPT3-srX         LEGLSFIIHDHSNRLRPVKFFFDQTVKVFEVMLP--SKSFIPSEFFKKIEQLTKPKNGTLL-MTSWIIFLRNIGLKHDVTQ--QQVLQFAKEVSIKAQTEGWSKEKVQNIVEVLLNHIFNDRKDLFQGTFLKELSMIPELCPERA 3659
zebrafish SIRPT3-srX      LEGLSFIYDSTNRLKPAKAFFDQTVKVFEVMLP--QKAFIPNDFFRKIEQVSKPKSGTSY-VPSWTTFLRNIGLKYVVSQ--QQVLQFAKEISIKAHTENWSKEKVQTIVDVLLNHIFNDRTDLFDGTFLKELSMISELCPERA 3660
                          *  : :*     :       ::*  :.::. ::        :    :     :: :.              ** :**:         : :  .* .:.              : :    .  **  :  ..     :         :   :  ::     :
                                                S             X                                                                               M   X       P     S   W  V
```

Supp. Figure S7. Multiple alignment of the *SIRPT1-srX* and *SIRPT3-srX* amino acid sequences of vertebrate sacsin proteins. Sequences (human, orangutan, dog, horse, mouse, rat, mouse, zebrafinch, chicken, anole, fugu, tetraodon, stickleback, medaka, zebrafish) were aligned by *ClustalW2* using default parameters. Then missense and nonsense mutations were mapped on the generated alignment. Amino acid changes due to mutations known to be pathogenic are highlighted in red (missense mutations) and purple (nonsense mutations) (see Table 1 for reference). Amino acid changes due to missense mutations not clearly associated to disease are highlighted in yellow (see Supp. Table S3 for reference). Amino acid changes that are known to be non-pathogenic are highlighted in green (Supp. Table S3). The following mutations are mapped: p.R728X, p.R742X, p.L802P, p.C991R, p.F1054S, p.C1066X, p.R3224X, p.W3248R, p.Y3430X, p.L3481P, p.R3636X, p.L3645P, p.F3653S, p.A694T, p.T727S, p.Q860K, p.Q936E, p.Q936K, p.V995F, p.V3369A, p.I3632M, p.R3658W.

Supp. Table S1. DNA sequences at the exon/intron boundaries of the zebrafish (*Danio rerio*) *sacs* gene

No.	Exon size (bp)	Donor exon/intron[a]	Intron size[b] (bp)	Acceptor intron/exon[a]
1	Not defined	AAGACCCGTG gtgagtttaa	7825	acaattttag GTTGCCATGG
2	151	TGGAAAAATG gtgagttgca	11559	ttgtgtatag GTTCATGATG
3	91	AGAGGTGGAG gtaagacact	5054	tcttttgcag GGAGGTTTGG
4	86	GATTCTAAAA gtaagaacat	113	tgctgtacag GAGCTGATTC
5	112	CAATATCAGG gtaacatgag	119	ttttctacag GAACAGCTTT
6	147	CACATAACAG gtgagaaaaa	3618	tcttttatag ATGTACCTAG
7	1843	AGTATCCCAG gtacaggcta	3063	ttatttctag GTCACTTTAT
8	92	AAAAGCAGAG gtagacaccc	3002	gtactttcag GGAGGCCTTG
9	Not defined			

[a]Exon sequences are indicated by upper case letters and intron sequences by lower case letters.
[b]Introns sizes were determined from sequence analysis of clone CH73-346G24 (GenBank acc. no. FP016239.4).
bp, base pair.

Supp. Table S2. Sequence identity (%) among vertebrate sacsin proteins

**Species**	human (4579 aa)	orangutan (4579 aa)	mouse (4582 aa)	rat (4581 aa)	dog (4578 aa)	horse (4577 aa)	chicken (4573 aa[a])	zebra finch (4578 aa)	anole lizard (4579 aa[a])	fugu (4579 aa)	tetraodon (4578 aa)	stickleback (4579 aa)	medaka (4579 aa)	zebrafish (4578 aa)
human (4579 aa)	100													
orangutan (4579 aa)	99.2 (99)	100												
mouse (4582 aa)	93.5 (93)	93.5 (93)	100											
rat (4581 aa)	93.4 (93)	93.4 (93)	98.1 (98)	100										
dog (4578 aa)	95.5 (95)	95.5 (95)	93.4 (93)	93.3 (93)	100									
horse (4577 aa)	94.6 (94)	94.5 (94)	92.4 (92)	92.4 (92)	95.4 (95)	100								
chicken (4573 aa[a])	84.6 (84)	84.7 (84)	84.2 (84)	84.2 (84)	84.9 (84)	84.3 (84)	100							
zebra finch (4578 aa)	84.8 (84)	84.9 (84)	84.3 (84)	84.3 (84)	85.3 (85)	84.7 (84)	94.7 (94)	100						
anole lizard (4579 aa[a])	83.0 (83)	83.0 (83)	82.8 (82)	82.7 (82)	83.3 (83)	82.9 (82)	85.3 (85)	85.5 (85)	100					
fugu (4579 aa)	68.8 (68)	68.8 (68)	68.6 (68)	68.6 (68)	68.7 (68)	68.7 (68)	69.1 (69)	69.6 (69)	68.7 (68)	100				
tetraodon (4578 aa)	67.8 (67)	67.9 (67)	68.1 (67)	68.0 (67)	68.1 (68)	68.2 (68)	68.3 (68)	68.9 (68)	68.1 (67)	90.9 (90)	100			
stickleback (4579 aa)	70.1 (69)	70.1 (69)	69.8 (69)	69.7 (69)	69.9 (69)	70.1 (69)	70.3 (70)	70.7 (70)	69.9 (69)	84.9 (84)	83.5 (83)	100		
medaka (4579 aa)	69.8 (69)	70.0 (69)	69.9 (69)	69.9 (69)	69.8 (69)	70.0 (69)	69.8 (69)	70.2 (69)	69.6 (69)	83.4 (83)	82.2 (82)	86.4 (86)	100	
zebrafish (4578 aa)	70.2 (70)	70.3 (70)	70.2 (70)	70.2 (70)	70.1 (70)	70.3 (70)	70.8 (70)	71.1 (70)	70.4 (70)	78.0 (78)	77.4 (77)	80.4 (80)	79.6 (79)	100

Pairwise scores were generated using *SIM*. Similar results were obtained using *ClustalW2* (parentheses).
[a]Amino acids from the first coding exon missing (see Supp. Fig. S1).
aa, amino acid.

Supp. Table S3. Human *SACS* gene (missense and nonsense only) polymorphisms mapped in this study

Variation (see SNP database at GenBank and/or SNP annotations at Ensembl)	***Exon***	***Mutation*** Nucleotide position in CDS[a]	***Amino acid change*** Amino acid position in protein[b]	***Clinical association*** (see *Reference*, last column)	***Reference***
rs34482854	7	c.484G>A	p.A162T	non-pathogenic	http://www.ncbi.nlm.nih.gov/snp?term=rs34482854
rs2031640	8	c.696T>A	p.N232K	non-pathogenic	[Vermeer et al., 2009; Guernsey et al., 2010]
	8	c.973G>A	p.G325R	non-pathogenic	[Vermeer et al., 2009]
rs78629788	8	c.1186A>G	p.N396D	unclear	http://www.ncbi.nlm.nih.gov/snp?term=rs78629788
	8	c.1373C>A	p.T458K	unclear	In house database
		c.1373C>G	p.T458R	unclear	In house database
rs76662648	8	c.1522C>A	p.L508M	non-pathogenic	http://www.ncbi.nlm.nih.gov/snp?term=rs76662648
	8	c.1846G>C	p.A616P	non-pathogenic	[Vermeer et al., 2009]
rs17325713	8	c.2080G>A	p.A694T	non-pathogenic	[Vermeer et al., 2009]
rs61760905	9	c.2180C>G	p.T727S	non-pathogenic	http://www.ncbi.nlm.nih.gov/snp?term=rs61760905
rs76194641	10	c.2578C>A	p.Q860K	non-pathogenic	http://www.ncbi.nlm.nih.gov/snp?term=rs76194641
rs61978562	10	c.2806C>A	p.Q936K	non-pathogenic	http://www.ncbi.nlm.nih.gov/snp?term=rs61978562
		c.2806C>G	p.Q936E	non-pathogenic	In house database
	10	c.2983G>T	p.V995F	non-pathogenic	[Vermeer et al., 2009]
rs76470518	10	c.3734A>G	p.Y1245C	non-pathogenic	http://www.ncbi.nlmnih.gov/snp?term=rs76470518
rs76872266	10	c.3752T>C	p.I1251T	non-pathogenic	http://www.ncbi.nlm.nih.gov/snp?term=rs76872266
	10	c.3990T>A	p.D1330E	non-pathogenic	[Guernsey et al., 2010]
rs61326562	10	c.4117G>C	p.A1373P	non-pathogenic	http://www.ncbi.nlm.nih.gov/snp?term=rs61326562
rs61548169	10	c.4118C>T	p.A1373V	non-pathogenic	http://www.ncbi.nlm.nih.gov/snp?term=rs61548169
	10	c.4466A>G	p.N1489S	non-pathogenic	[Vermeer et al., 2009]
rs117929959	10	c.5443T>C	p.C1815R	non-pathogenic	http://www.ncbi.nlm.nih.gov/snp?term=rs117929959
rs35865691	10	c.6051G>C	p.K2017N	non-pathogenic	http://www.ncbi.nlm.nih.gov/snp?term=rs35865691
rs80132141	10	c.6336A>C	p.R2112S	unclear	http://www.ncbi.nlm.nih.gov/snp?term=rs80132141
rs61948369	10	c.6344A>C	p.H2115P	unclear	http://www.ncbi.nlm.nih.gov/snp?term=rs61948369
	10	c.6781C>A	p.L2261I	non-pathogenic	[Vermeer et al., 2009]
rs79291029	10	c.6833T>G	p.V2278G	unclear	http://www.ncbi.nlm.nih.gov/snp?term=rs79291029
	10	c.6946A>G[c]	p.T2316A	non-pathogenic	[Vermeer et al., 2009]
rs61754478	10	c.7140T>A	p.N2380K	non-pathogenic	[Vermeer et al., 2009]
rs113600013	10	c.7183T>C	p.C2395R	non-pathogenic	http://www.ncbi.nlm.nih.gov/snp?term=rs113600013
rs111250527	10	c.7190T>C	p.V2397A	non-pathogenic	http://www.ncbi.nlm.nih.gov/snp?term=rs111250527
rs112638594	10	c.7300A>G	p.S2434G	non-pathogenic	http://www.ncbi.nlm.nih.gov/snp?term=rs112638594
rs78239814	10	c.7384C>T	p.P2462S	unclear	http://www.ncbi.nlm.nih.gov/snp?term=rs78239814
rs111920492	10	c.7528G>A	p.A2510T	non-pathogenic	http://www.ncbi.nlm.nih.gov/snp?term=rs111920492
rs36060617	10	c.7880A>G	p.N2627S	unclear	http://www.ncbi.nlm.nih.gov/snp?term=rs36060617
rs78827970	10	c.8127A>C	p.K2709N	non-pathogenic	http://www.ncbi.nlm.nih.gov/snp?term=rs78827970
rs117846594	10	c.8245A>G	p.I2749V	non-pathogenic	http://www.ncbi.nlm.nih.gov/snp?term=rs117846594
rs111540787	10	c.8339T>G	p.F2780C	non-pathogenic	http://www.ncbi.nlm.nih.gov/snp?term=rs111540787
rs61742502	10	c.8344G>A	p.A2782T	unclear	http://www.ncbi.nlm.nih.gov/snp?term=rs61742502
		c.8344G>C	p.A2782P	unclear	In house database
		c.8344G>T	p.A2782S	unclear	In house database
rs61742500	10	c.8345C>T	p.A2782V	unclear	http://www.ncbi.nlm.nih.gov/snp?term=rs61742500
		c.8345C>A	p.A2782E	unclear	In house database
		c.8345C>G	p.A2782G	unclear	In house database
rs9552930	10	c.8482A>G	p.S2828G	non-pathogenic	http://www.ncbi.nlm.nih.gov/snp?term=rs9552930
rs11839380	10	c.8873A>G	p.K2958R	non-pathogenic	http://www.ncbi.nlm.nih.gov/snp?term=rs11839380
rs17078605	10	c.10106T>C	p.V3369A	non-pathogenic	[Engert et al., 2000; Vermeer et al., 2009]
rs35256065	10	c.10896A>G	p.I3632M	non-pathogenic	http://www.ncbi.nlm.nih.gov/snp?term=rs35256065
rs115155117	10	c.10972C>T	p.R3658W	unclear	http://www.ncbi.nlm.nih.gov/snp?term=rs115155117
rs36061856	10	c.10982C>T	p.A3661V	unclear	http://www.ncbi.nlm.nih.gov/snp?term=rs36061856
rs17078601	10	c.11032C>G	p.P3678A	non-pathogenic	[Vermeer et al., 2009]
	10	c.11780C>T	p.A3927V	unclear	[Guernsey et al., 2010]
rs113609509	10	c.12380A>G	p.N4127S	non-pathogenic	http://www.ncbi.nlm.nih.gov/snp?term=rs113609509
rs35799469	10	c.12649A>G	p.N4217D	unclear	http://www.ncbi.nlm.nih.gov/snp?term=rs35799469
	10	c.12649A>C	p.N4217H	unclear	In house database
rs76919938	10	c.13002G>T	p.L4334F	unclear	http://www.ncbi.nlm.nih.gov/snp?term=rs76919938
	10	c.13522A>C	p.K4508Q	unclear	[Vermeer et al., 2009]
rs34382952	10	c.13717A>C	p.N4573H	unclear	[Vermeer et al., 2009]

This table lists 57 missense mutations described as Single Nucleotide Polymorphisms (SNPs) in humans (January 2012[d]). A total of 8 variants are new and as yet unpublished, and were identified upon a large collaborative clinical-genetic work performed in the laboratory of one of us (Filippo M. Santorelli, in house database) on behalf of SPATAX, the Euro-Mediterranean clinical network on inherited ataxias and spastic paraplegias. Detailed information on the associated clinical and paraclinical features in patients as well as on mutation analyses will be presented elsewhere. Such mutations are either undoubtedly non-pathogenic (35 missense mutations) or not yet clearly associated with disease (22 missense mutations). Unclear were defined gene variants present in SNP databases or also detected in low percentage in healthy controls but dubbed as "probably damaging" following analysis with PolyPhen-2 (Polymorphism Phenotyping v2; http://genetics.bwh.harvard.edu/pph2/), a tool which predicts possible impact on the structure and function using physical and comparative considerations.

[a]Based on the following NCBI Reference Sequence: GenBank acc. no. NM_014363.4. DNA mutation numbering system in use is based on cDNA sequence (with a 'c.' symbol before the number). Nucleotide numbering reflects cDNA numbering with +1 corresponding to the A of the ATG translation initiation codon in the reference sequence, with the initiation codon being codon 1 (www.hgvs.org).

[b]Based on the following NCBI Reference Sequence: GenBank acc. no. NP_055178.3. Amino acid change numbering system in use is based on protein sequence (with a 'p.' symbol before the letter) (www.hgvs.org).

[c]SNP not validated.

[d]New validated SNPs from dBSNP are reported in Appendix II; new SNPs from NHLBI Exome Sequencing Project are reported in Appendix III.

SNP, Single Nucleotide Polymorphism; CDS, coding sequence.

Supp. Table S4. Detailed positional information and distribution of the mutations within the various domains along human sacsin

Domain	*Domain length (amino acids)*	*Missense mutations (pathogenic)*	*Nonsense mutations (pathogenic)*	*Missense mutations (non-pathogenic)*	*Missense mutations (unclear)*	*Missense mutations (uncertain; pathogenic in double mutants)*[a]	*Total mutations*
Interdomain	11	-	-	-	-	-	-
Ubiquitin-like	72	-	-	-	-	-	-
SIRPT1-sr1	256	6 (6/0/0/0)[b]	2 (1/1/0/0)	3 (3/0/0/0)	-	-	11 (10/1/0/0)
Interdomain	60	-	-	-	1 (0/0/1/0)	-	1 (0/0/1/0)
SIRPT1-sr2	158	3 (3/0/0/0)	1 (1/0/0/0)	1 (1/0/0/0)	2 (2/0/0/0)	1[c] (1/0/0/0)	8 (8/0/0/0)
Interdomain	86	1 (1/0/0/0)	-	1 (0/0/0/1)	-	-	2 (1/0/0/1)
SIRPT1-srX	519	3 (3/0/0/0)	3 (2/0/0/1)	6 (0/3/1/2)	-	-	12 (5/3/1/3)
Interdomain	49	-	-	-	-	-	-
SIRPT1-sr3	163	1 (0/1/0/0)	2 (1/0/0/1)	5 (1/1/3/0)	-	-	8 (2/2/3/1)
Interdomain	69	2 (2/0/0/0)	-	-	-	-	2 (2/0/0/0)
SIRPT2-sr1	304	5 (5/0/0/0)	3 (2/1/0/0)	1 (0/0/1/0)	-	-	9 (7/1/1/0)
Interdomain	78	-	-	1 (0/0/0/1)	-	-	1 (0/0/0/1)
SIRPT2-sr2	143	2 (2/0/0/0)	1 (0/1/0/0)	-	-	-	3 (2/1/0/0)
Interdomain	318	-	2 (1/1/0/0)	2 (1/1/0/0)	3 (2/1/0/0)	-	7 (4/3/0/0)
SIRPT2-sr3	157	1 (1/0/0/0)	1 (1/0/0/0)	5 (2/0/2/1)	-	-	7 (4/0/2/1)
Interdomain	68	-	1 (1/0/0/0)	1 (1/0/0/0)	1 (1/0/0/0)	-	3 (3/0/0/0)
SIRPT3-sr1	257	2 (2/0/0/0)	1 (1/0/0/0)	2 (1/1/0/0)	1 (1/0/0/0)	-	6 (5/1/0/0)
Interdomain	57	-	-	1 (1/0/0/0)	6 (6/0/0/0)	1[c] (1/0/0/0)	8 (8/0/0/0)
SIRPT3-sr2	135	-	1 (1/0/0/0)	2 (1/0/0/1)	-	-	3 (2/0/0/1)
Interdomain	120	-	-	-	-	-	-
SIRPT3-srX	579	4 (3/1/0/0)	3 (3/0/0/0)	2 (0/2/0/0)	1 (1/0/0/0)	2[d] (2/0/0/0)	12 (9/3/0/0)
XPCB	76	-	1 (0/0/0/1)	1 (1/0/0/0)	1 (0/0/0/1)	-	3 (1/0/0/2)
SIRPT3-sr3	161	-	1 (1/0/0/0)	-	-	-	1 (1/0/0/0)
Interdomain	404	1 (1/0/0/0)	3 (2/1/0/0)	1 (0/0/1/0)	3 (1/0/2/0)	-	8 (4/1/3/0)
DnaJ	60	3 (2/1/0/0)	1 (1/0/0/0)	-	1 (1/0/0/0)	-	5 (4/1/0/0)
Interdomain	90	-	1 (1/0/0/0)	-	-	-	1 (1/0/0/0)
HEPN	117	3 (3/0/0/0)	-	-	1 (1/0/0/0)	-	4 (4/0/0/0)
Interdomain	12	-	-	-	1 (0/0/1/0)	-	1 (0/0/1/0)
Sacsin	4579	37 (34/3/0/0)	28 (20/5/0/3)	35 (13/8/8/6)	22 (16/1/4/1)	4 (4/0/0/0)	126 (87/17/12/10)

When mapped along the sacsin sequence (see Supp. Fig. S2), the vast majority of the natural mutations (111 out of 126) found in human sacsin fell in domains among those defined, namely *SIRPTs*, DnaJ and HEPN domain (see also Supp. Fig. S3). In particular, 42 mutations were in *SIRPT1*, 27 in *SIRPT2*, 33 in *SIRPT3*, 5 in DnaJ and 4 in HEPN, whereas 15 mutations were in the regions external to these domains. The mutations were pathogenic missense mutations (37 mutations), pathogenic nonsense (protein truncating) mutations (28 mutations), non-pathogenic missense mutations (35 mutations), unclear missense mutations (22 mutations) and unclear double missense mutations (i.e. the allele contained a double missense mutation that resulted in a pathogenic state) (4 mutations). Moreover, 46 natural pathogenic mutations (27 missense and 19 nonsense mutations) were within the borders of the newly identified *SIRPT* sub-repeats, that occurred in triplicate (*sr1*, *sr2*, *sr3*) and duplicate (*srX*) in the sacsin protein. In the same regions, 27 non-pathogenic missense mutations (among SNPs) and 7 missense mutations not obviously associated with disease (4 unclear missense mutations among SNPs and 3 among the pathogenic double missense mutations) could also be located. The distribution of all the various categories of mutations considered in this study within the different sacsin domains is schematically represented in this table.

[a]For details, see: Baets et al., 2010.

[b]The schematic notation (number1/number2/number3/number4) indicates the number of positions along the alignment respectively occupied by identical (*), conserved (:), semi-conserved (.) and non-conserved ( ) amino acids (as assessed by ClustalW) in a given domain.

[c]p.[L556P;P2798Q]+[L556;P2798Q]. Based on the NCBI Reference Sequence: GenBank acc. no. NP_055178.3. Amino acid change numbering system in use is based on protein sequence (with a 'p.' symbol before the letter) (www.hgvs.org).

[d]p.[R3636Q;P3652T]+[R3636Q;P3652T]. Based on the NCBI Reference Sequence: GenBank acc. no. NP_055178.3. Amino acid change numbering system in use is based on protein sequence (with a 'p.' symbol before the letter) (www.hgvs.org).

SNPs, Single Nucleotide Polymorphisms.

Supp. Table S5. Detailed positional information and distribution of the missense mutations in the aligned *SIRPT sr1, sr2, sr3* and *srX* domains

Aligned Domains	*Missense mutations (pathogenic)*	*Missense mutations (non-pathogenic)*	*Missense mutations (unclear)*	*Total mutations*
SIRPT1-sr1	6 (5/1/0/0)[a]	3 (0/0/0/3)	-	11 (5/1/0/5)
SIRPT2-sr1	5 (2/0/0/3)	1 (0/0/0/1)	-	9 (2/0/0/7)
SIRPT3-sr1	2 (2/0/0/0)	2 (0/1/0/1)	1 (1/0/0/0)	6 (3/1/0/2)
**Sub-total *sr1***	13 (9/1/0/3)	6 (0/1/0/5)	1 (1/0/0/0)	26 (10/2/0/14)
SIRPT1-sr2	3 (3/0/0/0)	1 (0/0/0/1)	2 (2/0/0/0)	8 (6/0/0/2)
SIRPT2-sr2	2 (1/0/0/1)	-	-	3 (1/0/0/2)
SIRPT3-sr2	-	2 (0/0/1/1)	-	3 (1/0/1/1)
**Sub-total *sr2***	5 (4/0/0/1)	3 (0/0/1/2)	2 (2/0/0/0)	14 (8/0/1/5)
SIRPT1-sr3	1 (0/0/0/1)	5 (0/0/1/4)	-	8 (0/0/1/7)
SIRPT2-sr3	1 (0/1/0/0)	5 (0/0/0/5)	-	7 (0/1/0/6)
SIRPT3-sr3	-	-	-	1 (0/0/1/0)
**Sub-total *sr3***	2 (0/1/0/1)	10 (0/0/1/9)	-	16 (0/1/2/13)
SIRPT1-srX	3 (1/1/0/1)	6 (0/2/0/4)	-	12 (2/3/0/7)
SIRPT3-srX	4 (1/2/0/1)	2 (0/1/0/1)	1 (0/1/0/0)	12 (1/4/0/7)
**Sub-total *srX***	7 (2/3/0/2)	8 (0/3/0/5)	1 (0/1/0/0)	24 (3/7/0/14)
**Total**	27 (15/5/0/7)	27 (0/4/2/21)	4 (3/1/0/0)	80 (21/10/3/46)

The multiple alignments of the *SIRPT sr1, sr2, sr3* and *srX* domains from the human and other vertebrate sacsin are represented in Supp. Fig. S4, Supp. Fig. S5, Supp. Fig. S6 and Supp. Fig. S7, respectively. When mapped on our alignments, it was evident that the majority (20 out of 27) of the natural pathogenic missense mutations occurred in human sacsin on amino acid residue positions that were invariably conserved a) in all vertebrate sacsin proteins and b) in each of the aligned *SIRPT* sub-repeat. Conversely, only 6 out of 27 non-pathogenic missense mutations (SNPs) could be placed in conserved positions across the *SIRPT* domains. The distribution of these mutations within and across the different sacsin domains is schematically represented in this table.

[a]The schematic notation (number1/number2/number3/number4) indicates the number of positions along the alignment respectively occupied by identical (*), conserved (:), semi-conserved (.) and non-conserved ( ) amino acids (as assessed by ClustalW) across domains.

SNPs, Single Nucleotide Polymorphisms.

Supp. Table S6. Composite SPAX score assigned to ARSACS patients selected for the presence on both alleles of either a frameshift mutation or a stop mutation or a macrodeletion

Origin	*SACS mutation* *Allele 1*[a]	*Allele 2*[a]	*Reference*	*Composite SPAX Score = Total score corrected for years disease duration/100*
Aragona-Spain/Croatia	c.832C>T (p.Q278X)	c.9670C>T (p.R3224X)	[Gazulla et al., 2012]	1.63
Japan	c.1184_1193delGTAACAGTGT (p.C395WfsX12)	c.2060delA (p.D687VfsX26)	[Ouyang et al., 2006]	1.60
The Netherlands	c.1475G>A (p.W492X)	c.961C>T (p.R321X)	[Vermeer et al., 2008; Vermeer et al., 2009]	1.72
The Netherlands	c.2182C>T (p.R728X)	c.2182C>T (p.R728X)	[Vermeer et al., 2008; Vermeer et al., 2009]	1.56
UK	c.2224C>T (p.R742X)	Δ (1.5 Mb macrodeletion; 6 genes are deleted)	[Terracciano et al., 2010]	1.52
Aragona-Spain	c.3198T>A (p.C1066X)	c.3198T>A (p.C1066X)	[Gazulla et al., 2012]	1.70
Italy	c.4033_4034insC (p.Q1345fsX4)	c.4033_4034insC (p.Q1345fsX4)	[Criscuolo et al., 2004]	1.84
Japan	c.4033C>T (p.Q1345X)	c.4033C>T (p.Q1345X)	[Okawa et al., 2006]	1.66
Italy	c.4108C>T (p.Q1370X)	c.6837_6838insA (p.K2279fsX11)	[Grieco et al., 2004]	1.84
The Netherlands (Turkish descent)	c.4957G>T (p.E1653X)	c.4957G>T (p.E1653X)	[Vermeer et al., 2008; Vermeer et al., 2009]	not possible to calculate
The Netherlands	c.5125C>T (p.Q1709X)	c.12160C>T (p.Q4054X)	[Vermeer et al., 2008; Vermeer et al., 2009]	not possible to calculate
Italy	c.5629C>T (p.R1877X)	c.5629C>T (p.R1877X)	[Anesi et al., 2011]	not possible to calculate
Algeria	c.5719C>T (p.R1907X)	c.5719C>T (p.R1907X)	In house database	1.56
Japan	c.5990_5991delCT (p.D1996fsX3)	c.5990_5991delCT (p.D1996fsX3)	[Shimazaki et al., 2007]	1.52
Japan	c.6355C>T (p.R2119X)	c.6355C>T (p.R2119X)	[Hara et al., 2007]	1.52
Algeria	c.6409C>T (p.Q2137X)	c.6409C>T (p.Q2137X)	[H'mida-Ben Brahim et al., 2011]	not possible to calculate
Italy	c.7250_7254delCAGAA (p.T2417fsX12)	c.7250_7254delCAGAA (p.T2417fsX12)	[Grieco et al., 2004]	1.82
Quebec	c.7504C>T (p.R2502X)	c.8844delT (p.P2948fsX4)	[Engert et al., 2000]	not possible to calculate
France	c.8289T>G (p.Y2763X)	c.8320A>T (p.R2774X)	In house database	not possible to calculate
Spain	c.8677A>T (p.R2893X)	c.11303insG (p.T3768fsX1)	In house database	1.50
Japan	c.8793delA (p.K2931fsX21)	c.8793delA (p.K2931fsX21)	[Hara et al., 2005]	not possible to calculate
Tunisia	c.10290C>G (p.Y3430X)	c.10290C>G (p.Y3430X)	[H'mida-Ben Brahim et al., 2011]	not possible to calculate
The Netherlands	c.10906C>T (p.R3636X)	c.961C>T (p.R321X)	[Vermeer et al., 2008; Vermeer et al., 2009]	1.54
Italy	c.11185C>T (p.Q3729X)	c.11185C>T (p.Q3729X)	[Masciullo et al., 2008]	1.48
Belgium (Hungarian descent)	c.11265_11266delAT (p.I3755MfsX7)	c.172del? (Ex3-5del)	[Baets et al., 2010]	not possible to calculate
The Netherlands	c.12160C>T (p.Q4054X)	c.12160C>T (p.Q4054X)	[Vermeer et al., 2008; Vermeer et al., 2009]	1.54
The Netherlands	c.12160C>T (p.Q4054X)	c.6000_6004delAAGAA (p.R2002fsX24)	[Vermeer et al., 2008; Vermeer et al., 2009]	1.68
The Netherlands	c.12160C>T (p.Q4054X)	c.2094-2 A>G[b] (possible exon skipping)	[Vermeer et al., 2008; Vermeer et al., 2009]	1.56
The Netherlands	c.2185+1delG[b] (possible exon skipping)	c.2185+1delG[b] (possible exon skipping)	[Vermeer et al., 2008; Vermeer et al., 2009]	1.64
Italy	c.12428_12429insA (p.Y4143X)	c.12428_12429insA (p.Y4143X)	[Prodi et al., 2012]	1.76
Tunisia	c.12851_12854delAGAG (p.E4284fsX23)	c.12851_12854delAGAG (p.E4284fsX23)	[Bouhlal et al., 2009]	not possible to calculate
Japan	c.12973C>T (p.R4325X)	c.12973C>T (p.R4325X)	[Takiyama et al., 2006; Yamamoto et al., 2006]	not possible to calculate
Italy	c.13132C>T (p.R4378X)	c.13132C>T (p.R4378X)	[Anesi et al., 2011]	not possible to calculate
UK	c.13237C>T (p.Q4413X)	Δ (1.5 Mb macrodeletion; 6 genes are deleted)	[Terracciano et al., 2010]	1.72

This table lists 34 ARSACS patients selected for the presence on both alleles of either a frameshift mutation or a stop mutation or a macrodeletion. A total of 3 patients are new and as yet unpublished, and were identified upon a large collaborative clinical-genetic work performed in the laboratory of one of us (Filippo M. Santorelli, in house database) on behalf of SPATAX, the Euro-Mediterranean clinical network on inherited ataxias and spastic paraplegias. Detailed information on the associated clinical and paraclinical features in patients as well as on mutation analyses will be presented elsewhere.

[a]Numbering based on the following NCBI Reference Sequences: GenBank acc. no. NM_014363.4, for nucleotide, and GenBank acc. n. NP_055178.3, for protein. DNA mutation numbering system in use is based on cDNA sequence (with a 'c.' symbol before the number). Nucleotide numbering reflects cDNA numbering with +1 corresponding to the A of the ATG translation initiation codon in the reference sequence, with the initiation codon being codon 1. Amino acid change numbering system in use is based on protein sequence (with a 'p.' symbol before the letter) (www.hgvs.org). Original nucleotide and amino acid positions and changes can be found in the original papers (see *Reference*).

[b]Splice site mutation.

del, base deletion (microdeletion); Δ, gene deletion (macrodeletion); Ex, exon; fs, frameshift; ins, insertion.

Appendix I. Variants not mapped in this study

Origin	*Exon*	*Nucleotide position (NM_014363.4)*	*Amino acid position (NP_055178.3)*	*Reference*
Belgium	4	c.216delT	p.C72fsX4	[Baets et al., 2010]
Japan	7	c.482delA	p.N161fsX14	[Kamada et al., 2008]
Italy	7	c.600_604+1delAACAGG	p.I200fsX6	[Terracciano et al., 2009]
China	8	c.1229delT	p.L410X	[Shen et al., 2012]
UK	9	c.2076delG	p.T692fsX15	[Pyle et al., 2012]
Tunisia	10	c.3328_3329insA	p.I1110fsX1	[El Euch-Fayache et al., 2003]
Belgium	10	c.3421_3422insAC	p.L1141fsX9	[Baets et al., 2010]
Tunisia	10	c.3585delT	p.I1195fsX11	[El Euch-Fayache et al., 2003]
UK	10	c.3965_3966delAC	p.G1322VfsX21	[Pyle et al., 2012]
Japan	10	c.5201_5202delAG	p.K1733fsX3	[Yamamoto et al., 2005]
China	10	c.5840C>G	p.P1947R	[Shen et al., 2012]
Serbia	10	c.6093_6095delTTC	p.S2032del	[Baets et al., 2010]
Japan	10	c.6172delT	p.F2457fsX19	[Yamamoto et al., 2005]
Belgium	10	c.7374delT	p.L2458fsX16	[Baets et al., 2010]
The Netherlands	10	c.8401_8403delCAA	p.Q2801del	[Vermeer et al., 2009]
Belgium	10	c.9331delA	p.N3111IfsX16	[Manto et al., unpublished data]
Turkey	10	c.10298delC	p.T3433fsX25	[Richter et al., 2004]
Belgium	10	c.11234_11235delTT	p.L3745RfsX1	[Baets et al., 2010]
Italy	10	c.11265_11266delAT	p.N3754fsX7	[Masciullo et al., 2012]
Italy	10	c.11598delC	p.G3866GfsX2	[Masciullo et al., 2012]
Italy	10	c.11777A>G	p.D3926G	[Masciullo et al., 2012]
Turkey	10	c.11829_11832delAGTT	p.L3943fsX3	[Richter et al., 2004]
Tunisia	10	c.13390G>T	p.D4464Y	[Bouhlal et al., unpublished data]

This table represents the recent update of additional pathogenic variants (small deletions, insertions) leading to frameshift in the SACS gene and described in ARSACS patients of different geographic origins. Positions follow the guidelines for mutation nomenclature with numbering +1 in cDNA corresponding to the A of the ATG translation initiation codon in the reference sequence, with the initiation codon being codon 1 (www.hgvs.org).
del, base deletion; fs, frameshift; ins, insertion.

Appendix II. New validated SNPs in dBSNP

Variation<br><br>(see SNP database at GenBank and/or SNP annotations at Ensembl)	***Exon***	***Mutation***<br><br>Nucleotide position in CDS (NM_014363.4)	***Amino acid change***<br><br>Amino acid position in protein (NP_055178.3)	***Clinical association***<br><br>(see *Reference*, last column)	***Reference***
rs148286091	6	c.1066T>G	p.I356L	non-pathogenic	http://www.ncbi.nlm.nih.gov/snp?term=rs148286091
rs145213666	7	c.1378G>A	p.L460F	unclear	http://www.ncbi.nlm.nih.gov/snp?term=rs145213666
rs146852400	8	c.2146G>A	p.H716Y	non-pathogenic	http://www.ncbi.nlm.nih.gov/snp?term=rs146852400
rs143677534	8	c.2234C>T	p.R745H	unclear	http://www.ncbi.nlm.nih.gov/snp?term=rs143677534
rs139993038	8	c.2926G>T	p.R976S	unclear	http://www.ncbi.nlm.nih.gov/snp?term=rs139993038
rs144267558	8	c.3427G>T	p.Q1143K	non-pathogenic	http://www.ncbi.nlm.nih.gov/snp?term=rs144267558
rs146451611	8	c.4076A>G	p.M1359T	non-pathogenic	http://www.ncbi.nlm.nih.gov/snp?term=rs146451611
rs147317123	10	c.10274T>C	p.K3425R	non-pathogenic	http://www.ncbi.nlm.nih.gov/snp?term=rs147317123

This table represents the recent update of additional SNPs in the SACS gene. Such mutations are either undoubtedly non-pathogenic or not yet clearly associated with disease. Unclear were defined gene variants present in SNP databases or also detected in low percentage in healthy controls but dubbed as “probably damaging” following analysis with PolyPhen-2 (Polymorphism Phenotyping v2; http://genetics.bwh.harvard.edu/pph2/), a tool which predicts possible impact on the structure and function using physical and comparative considerations. Positions follow the guidelines for mutation nomenclature with numbering +1 in cDNA corresponding to the A of the ATG translation initiation codon in the reference sequence, with the initiation codon being codon 1 (www.hgvs.org).
SNP, Single Nucleotide Polymorphism; CDS, coding sequence.

Appendix III. SNPs in Exome projects

Variation<br><br>(see SNP database at GenBank and/or SNP annotations at Ensembl)	***Exon***	***Mutation***<br><br>Nucleotide position in CDS (NM_014363.4)	***Amino acid change***<br><br>Amino acid position in protein (NP_055178.3)	***Clinical association***
rs146610589	3	c.443A>G	p.M148T	non-pathogenic
	3	c.446G>A	p.A149V	non-pathogenic
rs146784878	4	c.655G>T	p.Q219K	unclear
	4	c.659G>A	p.T220I	non-pathogenic
rs116907814	5	c.810A>C	p.F270L	non-pathogenic
	5	c.956T>A	p.Y319F	non-pathogenic
	5	c.981C>A	p.E327D	unclear
rs149444716	6	c.1067A>G	p.I356T	non-pathogenic
	6	c.1081T>C	p.K361E	non-pathogenic
	6	c.1094C>T	p.S365N	unclear
	6	c.1097T>C	p.N366S	non-pathogenic
rs137924716	6	c.1123C>T	p.V375I	non-pathogenic
	6	c.1129T>C	p.I377V	non-pathogenic
	6	c.1144C>T	p.E382K	non-pathogenic
	6	c.1272C>G	p.M424I	non-pathogenic
rs142024534	6	c.1273G>T	p.P425T	non-pathogenic
rs138413501	6	c.1278T>A	p.L426F	unclear
rs138842240	6	c.1369T>C	p.S457G	non-pathogenic
rs149951538	7	c.1387G>A	p.H463N	unclear
rs141487769	7	c.1444G>C	p.L482N	non-pathogenic
	7	c.1463G>A	p.P488L	unclear
	7	c.1519T>C	p.T507A	non-pathogenic
rs147821633	7	c.1544C>T	p.R515H	unclear
	7	c.1562C>G	p.S521T	non-pathogenic
	7	c.1592A>G	p.I531T	non-pathogenic
	7	c.1613G>A	p.A538V	non-pathogenic
rs140507581	7	c.1640G>C	p.P547R	unclear
	7	c.1762T>C	p.K588E	non-pathogenic
	8	c.1880G>A	p.T627M	unclear
rs149638449	8	c.1885C>T	p.A629T	non-pathogenic
	8	c.1907C>T	p.R636Q	unclear
	8	c.2023T>A	p.N675Y	non-pathogenic
rs146395198	8	c.2024T>C	p.N675S	non-pathogenic
rs138971880	8	c.2059C>T	p.D687N	unclear
	8	c.2096G>C	p.S699C	non-pathogenic
	8	c.2123A>T	p.F708Y	non-pathogenic
rs141006271	8	c.2140T>C	p.K714E	unclear
rs141553858	8	c.2294T>C	p.Y765C	unclear
rs147837803	8	c.2329A>G	p.S777P	unclear
rs142131182	8	c.2425C>G	p.E809Q	non-pathogenic
	8	c.2476C>T	p.V826I	non-pathogenic

	8	c.2492T>C	p.E831G	non-pathogenic
rs143433500	8	c.2497C>T	p.E833K	unclear
rs151172635	8	c.2534T>C	p.Q845R	non-pathogenic
	8	c.2548A>G	p.F850L	non-pathogenic
rs142037771	8	c.2564A>G	p.L855S	unclear
rs142284018	8	c.2602T>C	p.I868V	non-pathogenic
	8	c.2611G>A	p.P871S	non-pathogenic
	8	c.2669T>C	p.N890S	non-pathogenic
	8	c.2681G>A	p.S894L	unclear
rs144362131	8	c.2776T>C	p.I926V	non-pathogenic
	8	c.2785G>A	p.R929C	unclear
	8	c.2810C>A	p.G937V	non-pathogenic
	8	c.2890T>C	p.I964V	non-pathogenic
rs150429953	8	c.2936T>C	p.N979S	non-pathogenic
	8	c.2996A>G	p.I999T	unclear
	8	c.3017T>C	p.H1006R	non-pathogenic
rs142300853	8	c.3073T>A	p.N1025Y	unclear
rs150981983	8	c.3074T>A	p.N1025I	unclear
	8	c.3082G>C	p.P1028A	non-pathogenic
rs143530284	8	C.3120G>C	p.I1040M	unclear
	8	c.3200T>C	p.N1067S	non-pathogenic
	8	c.3242G>A	p.P1081L	non-pathogenic
	8	c.3265G>C	p.Q1089E	unclear
rs139805032	8	c.3391G>A	p.L1131F	unclear
	8	c.3419G>T	p.T1140K	non-pathogenic
rs140028127	8	c.3443T>G	p.K1148T	unclear
rs150694997	8	c.3514C>T	p.V1172I	unclear
	8	c.3545G>A	p.A1182V	non-pathogenic
	8	c.3551G>C	p.P1184R	non-pathogenic
	8	c.3550G>A	p.P1184S	non-pathogenic
rs148565480	8	c.3617T>C	p.H1206R	non-pathogenic
rs144713081	8	c.3778G>C	p.H1260D	unclear
rs139012356	8	c.4064T>C	p.N1355S	unclear
rs138245586	8	c.4255T>C	p.I1419V	unclear
	8	c.4453C>T	p.A1485T	unclear
rs148255791	8	c.4508A>G	p.M1503T	non-pathogenic
	8	c.4513T>C	p.I1505V	non-pathogenic
rs144653411	8	c.4522T>G	p.N1508H	unclear
	8	c.4628T>C	p.E1543G	non-pathogenic
	8	c.4649A>G	p.V1550A	non-pathogenic
	8	c.4654T>G	p.K1552Q	unclear
	8	c.4663T>C	p.K1555E	unclear
	8	c.4669C>G	p.G1557R	unclear
	8	c.4757T>C	p.N1586S	unclear
rs140953505	8	c.4894T>G	p.T1632P	non-pathogenic
rs143961484	8	c.4900C>G	p.E1634Q	unclear

	8	c.4936G>C	p.L1646M	unclear
rs148824690	8	c.4955T>C	p.Q1652R	unclear
rs139387396	8	c.4985G>A	p.T1662M	unclear
	8	c.5147T>C	p.K1716R	unclear
	8	c.5315C>A	p.R1772I	unclear
	8	c.5336G>A	p.S1779L	unclear
rs147517201	8	c.5368C>T	p.A1790T	non-pathogenic
rs144715822	8	c.5391C>G	p.K1797N	unclear
	8	c.5461A>G	p.C1821R	unclear
rs147273896	8	c.5513C>T	p.G1838E	unclear
	8	c.5522A>G	p.L1841P	unclear
	8	c.5545C>T	p.V1849I	non-pathogenic
rs140678034	8	c.5583C>G	p.W1861C	unclear
rs138586923	8	c.5592T>A	p.K1864N	unclear
	8	c.5617A>C	p.Y1873N	unclear
	8	c.5732G>A	p.T1911M	unclear
rs144822691	8	c.5744T>C	p.H1915R	unclear
	8	c.5848C>T	p.D1950N	unclear
	8	c.5849T>C	p.D1950G	non-pathogenic
	8	c.5915C>A	p.G1972V	unclear
	8	c.5932C>T	p.V1978I	unclear
rs140058391	8	c.5992T>C	p.I1998V	non-pathogenic
rs149018756	8	c.6084T>A	p.E2028D	non-pathogenic
	8	c.6178C>G	p.V2060L	unclear
rs140946485	8	c.6241G>C	p.L2081V	unclear
	8	c.6266G>A	p.S2089L	non-pathogenic
rs150018812	8	c.6278C>T	p.R2093H	unclear
	9	c.6377A>G	p.I2126T	non-pathogenic
	9	c.6389C>G	p.R2130T	unclear
	9	c.6409G>C	p.Q2137E	non-pathogenic
rs138732180	9	c.6457T>C	p.M2153V	unclear
rs146031135	9	c.6460C>T	p.A2154T	unclear
	9	c.6485T>C	p.D2162G	unclear
	9	c.6518A>G	p.I2173T	non-pathogenic
rs142530357	9	c.6542G>A	p.A2181V	unclear
rs138040321	10	c.6570A>C	p.S2190R	unclear
rs149278134	10	c.6577C>T	p.D2193N	unclear
rs138379074	10	c.6640G>A	p.R2214C	non-pathogenic
	10	c.6742C>A	p.A2248S	non-pathogenic
rs150098233	10	c.6776G>A	p.P2259L	unclear
	10	c.6892T>G	p.K2298Q	non-pathogenic
rs147949881	10	c.6952C>T	p.A2318T	unclear
rs146909241	10	c.6953G>A	p.A2318V	unclear
rs143414642	10	c.7003T>C	p.M2335V	non-pathogenic
rs141091294	10	c.7049T>C	p.E2350G	unclear
rs142869943	10	c.7165C>T	p.V2389M	non-pathogenic

	10	c.7189C>G	p.V2397L	unclear
	10	c.7288C>T	p.E2430K	unclear
	10	c.7325T>C	p.E2442G	unclear
	10	c.7426C>T	p.V2476I	unclear
	10	c.7509G>T	p.H2503Q	unclear
rs150783263	10	c.7892T>C	p.H2631R	unclear
rs141197941	10	c.7930T>C	p.I2644V	non-pathogenic
	10	c.7988G>A	p.P2663L	unclear
	10	c.8012T>C	p.D2671G	unclear
	10	c.8062T>C	p.T2688A	non-pathogenic
rs137945310	10	c.8203C>T	p.A2735T	unclear
	10	c.8222A>G	p.L2741P	unclear
rs146898231	10	c.8243G>T	p.S2748Y	unclear
	10	c.8254T>C	p.I2752V	non-pathogenic
	10	c.8281C>T	p.V2761M	unclear
	10	c.8395C>G	p.V2799L	non-pathogenic
	10	c.8417A>G	p.M2806T	non-pathogenic
	10	c.8450G>A	p.T2817M	unclear
	10	c.8491T>C	p.K2831V	non-pathogenic
rs142358701	10	c.8509T>C	p.I2837V	non-pathogenic
rs146554323	10	c.8784C>A	p.Q2928H	unclear
	10	c.8915G>T	p.P2972Q	unclear
	10	c.8972C>T	p.R2991H	unclear
	10	c.9031T>C	p.I3011V	non-pathogenic
rs144616826	10	c.9082C>A	p.D3028Y	unclear
	10	c.9238A>G	p.Y3080H	unclear
rs149290718	10	c.9251A>G	p.I3084T	non-pathogenic
rs139579036	10	c.9275T>A	p.Y3092F	unclear
	10	c.9382G>C	p.L3128V	unclear
	10	c.9404A>G	p.L3135S	unclear
	10	c.9476A>G	p.L3159P	unclear
rs143557803	10	c.9550G>A	p.R3184C	unclear
rs137905181	10	c.9562A>G	p.F3188L	unclear
	10	c.9637T>C	p.S3213G	unclear
	10	c.9656G>C	p.S3219C	non-pathogenic
	10	c.9668G>A	p.P3223L	unclear
	10	c.9788T>C	p.E3263G	non-pathogenic
rs147363191	10	c.9799T>C	p.T3267A	non-pathogenic
	10	c.10076G>A	p.T3359I	non-pathogenic
	10	c.10075T>C	p.T3359A	non-pathogenic
	10	c.10238T>C	p.Y3413C	unclear
rs144179865	10	c.10291C>G	p.V3431L	unclear
	10	c.10298G>A	p.T3433I	non-pathogenic
	10	c.10348A>G	p.S3450P	unclear
rs148659448	10	c.10375A>C	p.L3459V	unclear

	10	c.10383T>G	p.E3461D	non-pathogenic
	10	c.10394A>C	p.V3465G	non-pathogenic
	10	c.10398A>C	p.I3466M	non-pathogenic
	10	c.10576T>C	p.I3526V	non-pathogenic
	10	c.10630T>C	p.T3544A	unclear
	10	c.10724T>C	p.H3575R	non-pathogenic
	10	c.10747C>T	p.V3583M	unclear
rs147379730	10	c.10818T>A	p.E3606D	unclear
	10	c.10819T>C	p.I3607V	non-pathogenic
rs150900362	10	c.10901T>A	p.Q3634L	unclear
rs141591052	10	c.10911C>T	p.M3637I	non-pathogenic
rs148349355	10	c.10925C>T	p.G3642E	unclear
	10	c.10989G>T	p.F3663L	non-pathogenic
rs138609508	10	c.11048T>C	p.N3683S	non-pathogenic
	10	c.11050C>T	p.G3684R	unclear
rs148925505	10	c.11066G>A	p.P3689L	unclear
	10	c.11123G>A	p.P3708L	unclear
rs145725116	10	c.11135G>A	p.T3712I	unclear
	10	c.11149T>C	p.K3717E	non-pathogenic
rs149745343	10	c.11194G>C	p.Q3732Q	non-pathogenic
rs140905170	10	c.11228G>C	p.P3743R	unclear
	10	c.11249T>C	p.N3750S	non-pathogenic
rs151012679	10	c.11279G>A	p.T3760M	unclear
rs145188410	10	c.11327A>G	p.I3776T	non-pathogenic
rs147369196	10	c.11363C>T	p.R3788H	unclear
rs143907401	10	c.11519T>C	p.K3840R	unclear
rs149427669	10	c.11576C>T	p.R3859H	unclear
	10	c.11585T>C	p.K3862R	non-pathogenic
	10	c.11656T>C	p.R3886G	non-pathogenic
	10	c.11717G>A	p.A3906V	non-pathogenic
	10	c.11887T>C	p.M3963V	unclear
rs138442606	10	c.11915C>A	p.R3972L	unclear
rs149139875	10	c.12046T>C	p.I4016V	non-pathogenic
rs147634443	10	c.12103T>C	p.I4035V	non-pathogenic
rs147013767	10	c.12148A>G	p.F4050L	unclear
	10	c.12260T>C	p.Y4087C	unclear
rs139538222	10	c.12437G>A	p.S4146L	unclear
	10	c.12533G>A	p.P4178L	unclear
	10	c.12589A>G	p.Y4197H	unclear
	10	c.12677T>A	p.Q4226L	unclear
	10	c.12701T>C	p.Y4234C	unclear
rs146148720	10	c.12705T>A	p.K4235N	unclear
	10	c.12854C>T	p.S4285N	non-pathogenic
	10	c.12862T>A	p.T4288S	non-pathogenic
	10	c.12913C>T	p.E4305K	non-pathogenic

rs138760047	10	c.12937C>T	p.V4313M	unclear
	10	c.12950G>A	p.A4317V	unclear
rs150225486	10	c.12968G>A	p.S4323L	unclear
	10	c.12982T>A	p.I4328F	unclear
	10	c.13036C>T	p.D4346N	unclear
	10	c.13343C>T	p.R4448H	unclear
rs138328181	10	c.13538C>T	p.S4513N	non-pathogenic
	10	c.13694G>T	p.A4565D	unclear

This table represents the recent update of additional SNPs in the *SACS* gene from exome project under the multicenter National Heart Lung and Blood Institute GO Exome Sequencing Project (NHLBI Exome Sequencing Project) initiative (at http://evs.gs.washington.edu/EVS/). Although these variants have low frequencies (<0.001% total alleles) and need to be validated, we defined them as either undoubtedly non-pathogenic or not yet clearly associated with disease (that is, unclear). Unclear were defined gene variants dubbed as “probably damaging” following analysis with PolyPhen-2 (Polymorphism Phenotyping v2; http://genetics.bwh.harvard.edu/pph2/), a tool which predicts possible impact on the structure and function using physical and comparative considerations. Positions follow the guidelines for mutation nomenclature with numbering +1 in cDNA corresponding to the A of the ATG translation initiation codon in the reference sequence, with the initiation codon being codon 1 (www.hgvs.org).
SNP, Single Nucleotide Polymorphism; CDS, coding sequence.
